# An Efficient Method for Contraction of Property-Oriented Basis Sets: A Considerable Reduction of the pecJ-1 and pecJ-2 Basis Sets for the Calculations of Spin–Spin Coupling Constants Involving H, C, N and F Nuclei

**DOI:** 10.3390/ijms27104650

**Published:** 2026-05-21

**Authors:** Irina L. Rusakova, Yuriy Yu. Rusakov

**Affiliations:** A. E. Favorsky Irkutsk Institute of Chemistry, Siberian Branch of the Russian Academy of Sciences, Favorsky St. 1, 664033 Irkutsk, Russia; rusakov82@mail.ru

**Keywords:** pecJ-1, pecJ-2, basis set, spin–spin coupling constant, property–energy consistent method, correlated ab initio methods

## Abstract

In this paper, it is suggested that the property–energy consistent (PEC) method represents an efficient and reliable method for generating accurate contraction coefficients for the property-oriented basis sets. Due to its peculiarities, the PEC method allows the implementation of rather succinct contraction schemes, without a noticeable loss of accuracy, giving in result very compact property-oriented basis sets that are capable of providing the same or even better accuracy than that reached with considerably larger basis sets of the same kind. This idea has been demonstrated on the example of the recontraction of previously introduced spin–spin coupling constant (SSCC)-oriented pecJ-*n* (*n* = 1, 2) basis sets for H, C, N, and F atoms, whose exponents were optimized by the PEC algorithm, but the contraction coefficients were defined using the usual self-consistent field calculations of the molecular energies of the simplest hydrides. In this work, the original pecJ-*n* basis sets were recontacted by means of the PEC method, resulting in compact segmented–contracted basis sets being smaller in size than their previous analogies by four and seven functions for hydrogen and the 2nd-period atoms, respectively. High-quality SOPPA(CCSD) calculations of 436 SSCCs of various types involving H, C, N, and F nuclei showed the supremacy of the newly contracted pecJ-*n* (*n* = 1, 2) basis sets over their original versions and most of the other well-known SSCC-oriented basis sets.

## 1. Introduction

In performing quantum chemical calculations of linear and nonlinear response properties with highly correlated ab initio methods, the problem of choosing the basis set possessing desired features has always been an utterly important issue. In this, the first and foremost aspect requiring special consideration is the balance between the size of the basis set and the accuracy attainable with it in the boundaries of a chosen correlated ab initio method: the fewer basis set functions are necessary for achieving a given level of accuracy, the better. For instance, the formal scaling factors of the three most accurate models of the coupled clusters (CC) theory [1,2,3,4], the CC singles and doubles (CCSD) [5], an iterative CC approach including non-iterative triples, the CC3 [6,7], and the CC singles, doubles, and triples (CCSDT) [8,9], are *N*^6^, *N*^7^, and *N*^8^ [1,10], respectively, with *N* being the number of basis set functions. At the same time, one of the most popular polarization propagator (PP) methods, the second-order polarization propagator approach (SOPPA) [11], with its modifications, the SOPPA(CC2) [12] and SOPPA(CCSD) [11,13], also imposes a great computational challenge, because the formal scaling factors for these models are, accordingly, *N*^5^, *N*^5^ and *N*^6^ [12]. For the sake of comparison, the scaling factor of the density functional theory (DFT) method [14,15] is only *N*^3^ [10] or can be even less, depending on the computer realization [16]. Currently, unlike the DFT method, highly correlated ab initio methods can hardly treat large- or even medium-sized molecules described with moderate-sized but sufficient basis sets, nor provide reasonably effective computational means of calculating small molecules in large-sized basis sets. Therefore, for the computationally costly high-quality ab initio correlated calculations, the size of the basis set is the main limiting factor that is responsible for their feasibility. For example, let us take benzene that is described with the well-known cc-pVDZ basis set [17], and conceive to add only one s-function to the basis set on each atom. In result, the number of elementary algorithmic operations within the methods with the scaling factors of *N*^5^, *N*^6^, *N*^7^ and *N*^8^ would increase 1.6, 1.8, 2.0 and 2.2 times, respectively. Thus, for the ab initio correlated methods, a diminution of the basis sets used in such computations just by one function already means a lot, and, if even the smallest reduction is possible without compromising the accuracy, this can be regarded as a considerable benefit.

The NMR indirect spin–spin coupling constant (SSCC) represents a molecular property which naturally requires highly correlated ab initio approaches. Of course, the linear response DFT can be applied to calculate SSCCs [18,19]; however, notwithstanding all its attraction due to favorable scaling characteristics, this method has several drawbacks undermining the reliability of the results. In particular, DFT does not allow the building of systematic hierarchical models with successive improvement accounting for the electron correlation effects to get the exact solution eventually [20]. In fact, the results obtained with the DFT method are strongly dependent on the exchange–correlation functional used [21,22,23]. Another pronounced problem is that the DFT calculation sometimes results in triplet instability when calculating the contributions to SSCCs of triplet types [24]. The onset of this computational phenomenon can easily be overlooked, if not paying attention to the Hessian matrix, which should be positively defined for stable solutions. On top of that, it is frequently the case that when the DFT method provides the results, they are noticeably inferior in accuracy relative to those of highly correlated ab initio approaches for molecules with a complex electronic structure, bearing strong correlation effects. For example, one of the most challenging cases for the DFT method is that of the SSCCs involving a fluorine nucleus. In this regard, the DFT values, in general, appear to be noticeably more deviating from the experiment than those obtained within the SOPPA, SOPPA(CC2), and SOPPA(CCSD) methods [22,25,26,27]. Meanwhile, for some extremely hard cases like dinitrogen difluoride (N_2_F_2_) or cyanogen fluoride (FCN), even the CCSD approximation is not enough and the inclusion of triple excitations is required [28,29], for example, in the framework of CC3 or CCSDT models.

The most suitable basis sets for the SSCCs’ calculations within highly correlated ab initio approaches are those which are optimized specifically for the calculation SSCCs; they are called the J-oriented basis sets. As compared to the usual energy-optimized basis sets, the J-oriented basis sets are indeed much more effective due to the smaller amount of basis functions needed for achieving the desired level of accuracy. In this regard, many types of J-oriented basis sets were proposed. To name a few, there are several well-known families of this kind that are readily accessible through the Basis Set Exchange (BSE) library [30,31]. These include the aug-cc-pVTZ-J [11,32,33,34,35], the 6-31G-J and 6-311G-J with their augmented and polarized versions [36], the (aug)pcJ-*n* (*n* = 0–4) [37,38], the ccJ-pVXZ (X = D, T, Q, 5) [39], and the acv*n*z-J (*n* = 2, 3, 4) [40]. We have also introduced a series of J-oriented basis sets recently, namely, the pecJ-*n* (*n* = 1, 2) [41,42,43], for a number of selected atoms. In particular, the basis sets for H, C, N, and F atoms [41] were added to the BSE library as well.

Generally, a reasonable question arises of whether is it possible to reduce the sizes of the existing J-oriented basis sets intended for the high-quality correlated ab initio calculations even more, without spoiling the accuracy of the results. This is a particularly important question, since the SSCCs are highly sensitive to the smallest changes in almost all basis set shells, and any attempts to reduce the size of one shell or another may prove fatal for the whole basis set [37]. In general, this is due to the fact that the SSCC is a very complex property, which consists of four distinct contributions, the Fermi contact (FC), the spin dipolar (SD), the paramagnetic spin–orbit (PSO), and the diamagnetic spin–orbit (DSO) terms. The FC, SD, and PSO contributions are expressed in the form of linear response functions, implying the summation over the excited states of the electron system. Each of the three has its own peculiarities and is sensitive to distinct types of basis set functions [44,45]. Thus, the FC depends on the electron density at the nuclear position and, for proper description, requires an enlarged number of tight s-type functions. The SD contribution is sensitive to the modifications in the space of the p-, d- and f-type functions with larger exponents. The description of the PSO contribution drastically improves when adding tight p-functions. The DSO contribution is only marginally sensitive to basis set changes.

We suppose that it is possible indeed to make a painless reduction of the J-oriented basis sets. The solution lies in applying a tighter contraction scheme, though performed in such an intricate way that the accuracy of the results is retained at the same level or even becomes better.

We have mentioned above that we have recently proposed the J-oriented basis sets named as pecJ-*n* (*n* = 1, 2) for a number of selected atoms. These were generated using the so-called property–energy consistent (PEC) method, introduced by us in 2021 [41]. This method represents a unique approach that allows the optimization of property-oriented basis sets based on the consistent optimization of exponents with respect to the property target function under the condition that the energy must be minimized. In particular, it also can be applied to the optimization of contraction coefficients, and in some works devoted to generating PEC-type basis sets for NMR shielding constants [46,47,48] and geometry optimization [49,50,51], we have successfully done so. Analyzing the efficiency of the PEC method in application to the contraction problem, we have come to an idea that this method could potentially be very promising in creating more compact property-oriented basis sets than those obtained by applying the usual means of contraction, namely, those which are based on the use of atomic or molecular orbital coefficients, obtained from the self-consistent field (SCF) or ab initio correlated energy calculations.

In this work, we aimed at solidifying this idea, showing the excellent performance of the PEC method as a method of contraction on the example of our very first J-oriented basis sets of the PEC-type, the pecJ-*n* (*n* = 1, 2) basis sets for H, C, N, and F atoms, which were proposed together with the PEC method [41]. The exponents for the original pecJ-*n* basis sets were generated with the PEC method, but the contraction coefficients were initially obtained by the usual way, namely, from the SCF calculation of the molecular energy of the simplest hydrides. Now, we have created new versions of these basis sets, keeping the exponents intact but applying a deeper contraction scheme with contraction coefficients optimized within the PEC method. Thus, newly contracted pecJ-*n* (*n* = 1, 2) basis sets are considerably smaller in size than their previous analogies, namely, by four and seven functions for both levels (*n* = 1, 2), for hydrogen and the 2nd-period atoms, respectively. Additionally, the accuracy that has been reached with these basis sets is even better than that provided by their older versions, their uncontracted versions and the majority of the other well-known J-oriented basis sets.

It is important to note that such considerable contraction of the pecJ-*n* (*n* = 1, 2) basis set sizes has become possible, in particular, due to the fact that we have carried out the contraction of the p- and d-shells for hydrogen and the 2nd-period atoms, respectively. This was not a trivial act. Most of the J-oriented basis sets, as one can conjecture, are not contracted in the first polarization shell, lest they lose their flexibility in the description of various contributions to the SSCCs, especially the ones of the triplet type (FC and SD). Overall, the pronounced sensitivity of the SSCCs to the number of functions in the first polarization shell is very well known [34,43]. Thus, if the contraction of the basis sets, including the space of the first polarization shell, is not carried out in a proper way, a failure of the whole basis set may ensue.

We have also tried different schemes of contraction, including the general [52] and the segmented [53] ones. From the beginning, it was hard to say which one would turn out to be the most efficient. As a result, both variants were generated and compared with each other, while the best one was proposed as the final version of the pecJ-*n* (*n* = 1, 2) basis sets.

The accuracy of the new pecJ-*n* (*n* = 1, 2) basis sets for H, C, N, and F has been tested on high-quality SOPPA(CCSD) calculations of a wide variety of SSCCs of different types, including ^1^H-^1^H, ^13^C-^1^H, ^13^C-^13^C, ^19^F-^13^C, ^19^F-^1^H, ^19^F-^19^F, ^15^N-^1^H, ^15^N-^13^C, ^19^F-^15^N, and ^15^N-^15^N. The performance of the final versions of the pecJ-*n* (*n* = 1, 2) basis sets was also subjected to a very rigorous trial, including the comparison of the calculated SSCCs with the gas phase NMR experiment. For that, high-precision CCSDT calculations of the SSCCs involving H, C, N, and F atoms of the smallest symmetric molecules were carried out with the pecJ-*n* (*n* = 1, 2) basis sets, and the vibrational corrections were taken into account at the CCSD level of theory. A good agreement with the experiment has been achieved using both the pecJ-1 and pecJ-2 basis sets.

## 2. Results and Discussion

### 2.1. New Contraction of the pecJ-n (n = 1, 2) Basis Sets for H, C, N, and F

Our goal in this work is to demonstrate that the application of the PEC method to the optimization of the contraction coefficients allows us to use the schemes of essentially deep contraction, resulting in rather compact basis sets that provide an accuracy of results which is comparable to or even better than that achieved with the noticeably larger basis sets of shallower contraction. For that purpose, we turned our attention to our first J-oriented pecJ-*n* (*n* = 1, 2) basis sets for H, C, N, and F atoms [41], seeking their improvement by increasing the contraction depth (*D*) while preserving or, maybe, even enhancing the original accuracy.

The contraction depth *D* is measured in this work within the percentage scale in accordance with the following formula:

(1) $$D=\frac{\left(N_{uc}-N_{c}\right)}{N_{uc}}\times 100\%,$$

The numbers $N_{uc}$ and $N_{c}$ in expression (1) designate the numbers of uncontracted (*uc*) and contracted (*c*) basis set functions.

The exponents for the original pecJ-*n* (*n* = 1, 2) basis sets for H, C, N, and F atoms were generated within the PEC method in our previous work [41], but the contraction coefficients were obtained by the usual way, namely, from the SCF energy minimization of the simplest hydrides of the mentioned elements. Actually, since the mentioned work was our first work on the PEC method, not much attention was paid to subtler issues such as the contraction schemes. Thus, the original pecJ-*n* (*n* = 1, 2) basis sets have a rather small contraction depth, being approximately 15–23% for hydrogen and only 9–16% for the 2nd-period atoms, depending on the cardinal number *n*.

In this work, the original pecJ-*n* (*n* = 1, 2) basis sets were fully uncontracted and the exponents of each shell were rearranged to form more succinct functional spaces for each shell. We have considered two possibilities of the rearrangement, including the segmented and general contraction schemes. The segmented contraction scheme is the older method, and it assumes that a given set of primitive Gaussian-type orbitals (PGTOs) is partitioned into smaller sets of functions that are combined into contracted Gaussian-type orbitals (CGTOs) by determining suitable coefficients. No one PGTO is allowed to enter more than one CGTO at once. At the same time, the general contraction scheme takes the latter restriction off: any PGTO can enter as many CGTOs as needed.

To unambiguously distinguish between the old and new versions of the pecJ-*n* (*n* = 1, 2) basis sets in this paper, we will adhere the following notations throughout the text: the notation pecJ-*n*-old (*n* = 1, 2) stands for the old version of the pecJ-*n* (*n* = 1, 2) basis set, and the notations pecJ-*n*-new(gen) and pecJ-*n*-new(seg) stand for the newly contracted versions of the pecJ-*n* (*n* = 1, 2) basis sets that embody the general and segmented schemes of contraction, respectively. The structures of the old and new versions of the pecJ-*n* (*n* = 1, 2) basis sets considered in this paper are given in Table 1.

It should be noted that we have considered different variants of contracted basis set compositions for H, C, N, and F, of the general and segmented types alike. In the case of nonhydrogen atoms, we have chosen only one scheme of each type, namely, the general one and the segmented one, that gives the least error at the end of the PEC optimization. In the case of hydrogen, we have selected the segmented version only, the pecJ-*n*-new(seg), as the optimization of the contraction coefficients for all possible general contraction schemes for hydrogen atoms ended up with unsatisfactorily high errors. Table 1 contains the compositions of the selected schemes, while those that provided larger errors were omitted from consideration due to their irrelevance. It should also be noted that the pecJ-*n*-old (*n* = 1, 2) basis sets were contracted in accordance with the segmented scheme, though the mentioning of this fact is omitted from their names herewith.

It can be seen from Table 1 that the compositions of the newly contracted pecJ-*n* (*n* = 1, 2) basis sets contain a much smaller number of CGTOs as compared to the old versions. Namely, the contracted functional spaces of the s- and p- (for H) and s- and d-shells (for C, N, and F) of the pecJ-*n*-new(gen/seg) basis sets are considerably smaller than those of the corresponding old versions. In particular, the new basis set compositions for hydrogen include one less s- and one less p-function than their old forms, which gives the reduction of the basis set size as four functions in total. At the same time, for the 2nd-period atoms, the new compositions of the pecJ-*n* basis sets are two s- and one d-function less than the corresponding old analogues, resulting in the reduction of the total size by seven functions. Thus, the contraction depth for the newly contracted basis sets is considerably larger than those of the corresponding old versions, being 46–27% for hydrogen and 43–29% for the 2nd-period atoms, depending on the cardinal number *n*.

The starting guess sets of the contraction coefficients were formed from the orbital atomic coefficients obtained from the SCF energy minimization for each atom. Then, the property–energy consistent (PEC) method was applied to optimize the contraction coefficients for the new compositions of the pecJ-*n* basis sets presented in Table 1, keeping the exponents unchanged. Notwithstanding that we have meticulously described the essence of the PEC method in the original paper [41], its root conception is worth mentioning here for the sake of convenience. The PEC approach embodies the constrained optimization of the basis set parameters (exponents or/and contraction coefficients) in relation to a certain molecular property, performed under the condition that the minimum of the total energy is achieved. Thus, the PEC algorithm randomly generates the parameters around the starting set via Monte Carlo simulations and the generated arrays are verified for whether they give a value of the property under interest within a desired range or not. Among the sets that provide the property in the desired range, only one set is selected, namely, that one which gives the lowest energy.

The current problem consists of the optimization of contraction coefficients. The target property or the objective function (as it is formally called in mathematics) is represented by the mean absolute error evaluated for the selected SSCCs calculated with the basis sets with varying contraction coefficients in relation to their so-called ideal or target values, calculated with a very large basis set that is close to the complete basis set (CBS) limit. In the present work, the ideal values were calculated with the ccJ-pV5Z [39] basis set. This is indeed a very large basis set which contains 66 and 107 contracted basis set functions for hydrogen and 2nd-period atoms, respectively. It amply describes the s-, p-, d-, and f-angular spaces, and even provides one function for the g-space for hydrogen atoms and two g- and one h-function for the 2nd-period atoms. Generally, the ccJ-pV5Z can be considered as a basis set that is close to the CBS limit.

Thus, briefly, our goal consisted of obtaining such contraction coefficients which would minimize the mean absolute deviation of the calculated SSCCs from their target values under the energetic constraint. Mathematically, the problem under consideration can be expressed as follows:

(2) $$\tilde{∆}=\frac{1}{N}\sum_{i=1}^{N}\left|{\tilde{J}}_{i}-J_{i}^{\mathrm{ideal}}\right|\to \min$$

(3) $$\sum_{j=1}^{M}{\tilde{E}}_{j}\to \min$$

In Equations (2) and (3), $\tilde{∆}$ represents the objective function which is to be minimized, and *N* is the total number of SSCCs participating in the PEC optimization. The SSCCs ${\left\{{\tilde{J}}_{i}\right\}}_{i=\overset{-}{1,N}}$ are calculated with the basis set with the contraction coefficients being under variation, while the SSCCs ${\left\{J_{i}^{\mathrm{ideal}}\right\}}_{i=\overset{-}{1,N}}$ represent the ideal values of the SSCCs. The ${\left\{{\tilde{E}}_{j}\right\}}_{j=\overset{-}{1,M}}$ are the energies of the fitting molecules, totaling in number to *M*.

In more detail, the fitting molecules that were used for the optimizations of the contraction coefficients of the pecJ-*n* (*n* = 1, 2)-new(gen/seg) basis sets included a variety of symmetric molecular systems, small enough to perform thousands of calculations during the optimization cycles. These are H_2_ and CH_4_ for hydrogen; C_2_H_2_, C_2_H_4_, and C_2_H_6_ for carbon; N_2_, N_2_H_2_, and N_2_H_4_ for nitrogen; and FCCF, CH_2_F_2_, and H_2_C-CF_2_ for fluorine. For each molecule that was used in the optimization of the contraction coefficients for the basis set for a certain element, the corresponding homonuclear SSCCs were being calculated during the optimization. Thus, the ^1^*J*(^1^H,^1^H) of H_2_ and ^2^*J*(^1^H,^1^H) of CH_4_ were considered in the case of the hydrogen basis sets; the ^1^*J*(^13^C,^13^C) and ^1^*J*(^15^N,^15^N) were considered for all fitting molecules for the carbon and nitrogen basis sets, respectively; and the ^3^*J*(^19^F,^19^F) of FCCF and ^2^*J*(^19^F,^19^F) of both H_2_C-CF_2_ and CH_2_F_2_ were considered for the fluorine basis sets.

The fact that we have used several molecules in the optimization also represents an advantage over the old contraction approach. As pertaining to the previous contraction, only one simplest hydride of the considered element has been considered. Such an approach may cause discrepancies in the accuracy of the results obtained for different molecular systems within the same method under the same conditions. At the same time, using several fitting molecules increases the robustness of the generated basis sets in relation to the diversity of electronic systems.

During the PEC-optimization of the contraction coefficients for the pecJ-*n*-new(gen/seg) basis sets, we have used the SOPPA(CCSD) method for the SSCCs’ calculation. This method was also used in the original paper [41] when performing the PEC optimization of the exponents for the pecJ-*n*-old basis sets. The SOPPA(CCSD) [13] represents an improvement over the original SOPPA method, replacing all first-order Møller–Plesset (MP) doubles and second-order MP singles correlation coefficients with the corresponding CCSD amplitudes that are used to calculate the resolvent matrix in the SOPPA approach. This makes it imperative to iteratively solve the system of the coupled cluster equations on the CCSD amplitudes, which results in increasing the computational cost of the SOPPA(CCSD) model relative to the classical SOPPA by an order of magnitude in terms of the number of basis set functions *N*, making its scaling factor *N*^6^. Thus, the SOPPA(CCSD) is a rather computer-expensive ab initio approach, which makes it rather cumbersome to use it in the optimization process when thousands of calculations of SSCCs are required. However, the SOPPA(CCSD) is theoretically superior to the SOPPA or SOPPA(CC2) [11,12,13], giving in general very accurate and reliable results, even for exceptionally challenging systems [40,54,55]. Therefore, in spite of its high computational cost, it is the precision and stability of the results which have determined our decision in preference of the SOPPA(CCSD) method. Taking into consideration that for the pecJ-*n*-new(gen/seg) (*n* = 1, 2) basis sets, the exponents and contraction coefficients have both been optimized at the SOPPA(CCSD) level, these basis sets can be regarded as the basis sets that are intended, in the first place, for the high-quality ab initio wavefunction-based correlated calculations of SSCCs.

It is an always-present peculiarity of our approach that we perform the optimization of the basis sets’ parameters for different elements sequentially, and we did so this time. When carrying out the basis set optimization for a series of elements, it is logical to start with the optimization for the most proliferated element that would enter the fitting molecules prepared for the other elements, then followed by the second most abundant element, and so on. Thus, the first basis sets whose contraction coefficients were subjected to the PEC-optimization were the basis sets for hydrogen. Then, in all fitting molecules prepared for the carbon and nitrogen basis sets, the newly contracted hydrogen pecJ-*n*-new(seg) basis sets were set on all hydrogen atoms and were kept fixed during the optimization. Lastly, the contraction coefficients for the fluorine basis sets were optimized, under the condition that the newly contracted hydrogen and carbon basis sets were set on all eponymous atoms and retained unchanged during the optimization.

All the newly contracted basis sets are presented in the Supplementary Material (see pages S3–S18).

It is important to underscore here that the pecJ-*n*-new(gen/seg) (*n* = 1, 2) basis sets contain a much smaller number of CGTOs as compared to their old versions, and this is essentially due to the contraction of the first polarization shell. Indeed, in the pecJ-*n*-old (*n* = 1, 2) basis sets, the p- (for H) and d-shells (for C, N, and F), which constitute the first polarization shell in each case, were not contracted at all (see their contraction schemes in Table 1). Generally, the reduction of the first polarization shell is especially dangerous for the J-oriented basis sets, for that can result in a dramatic loss of flexibility of the basis set in describing, in the first place, the triplet second-order contributions to the SSCCs, such as FC or SD. Indeed, the quantum chemical expressions for the second-order triplet contributions to SSCCs represent the linear response functions involving the summation over the triplet excited states [56]. The polarization functions of the first polarization shell in the highest degree are responsible for the correct description of the distortion of the molecular orbitals, capturing the response of the excited triplet wavefunctions to the magnetic field. Factually, the reduction of the first polarization shell could have destroyed the flexibility of the J-oriented basis set in important regions, but the PEC method, which has been applied to the optimization of the contraction coefficients, allowed the avoidance of this dangerous situation, which will be demonstrated in the subsequent sections of this paper.

### 2.2. Testing the Newly Contracted pecJ-n (n = 1, 2) Basis Sets for H, C, N, and F Against Theoretical Data

To estimate the performance of the pecJ-*n*-new(gen/seg) basis sets with respect to their older versions, their uncontracted versions, and the other J-oriented basis sets, we have conducted several tests. The first test consisted of the comparison of the theoretical accuracies that can be reached with the pecJ-*n*-new(gen/seg), pecJ-*n*-old, and the uncontracted pecJ-*n*(uc) (*n* = 1, 2) basis sets when performing the SOPPA(CCSD) calculations of the SSCCs involving ^1^H, ^13^C, ^19^F, and ^15^N nuclei. By “theoretical accuracy” we imply that it is assessed with respect to the corresponding reference theoretical data, representing the result of the SOPPA(CCSD)/ccJ-pV5Z calculation.

**Figure 1:**
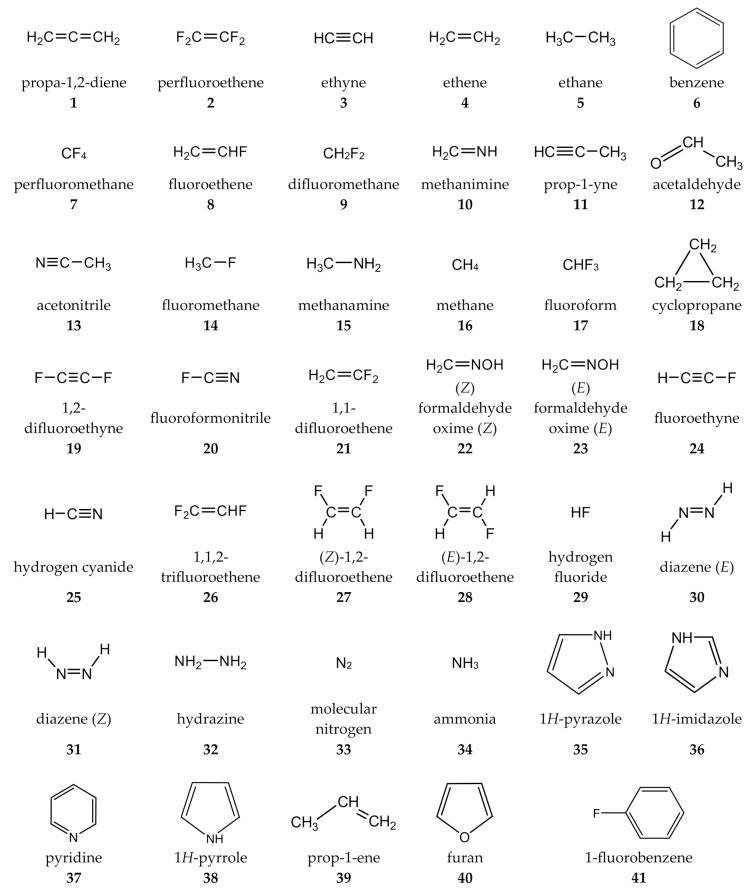
Benchmark molecules of set 1.

The calculations were carried out for a set of small- and medium-sized molecules, having versatile and sometimes computationally challenging electronic structures. These molecules, totaling in number to 41 and providing 436 SSCCs of various types (^1^H-^1^H, ^13^C-^1^H, ^13^C-^13^C, ^19^F-^13^C, ^19^F-^1^H, ^19^F-^19^F, ^15^N-^1^H, ^15^N-^13^C, ^19^F-^15^N, and ^15^N-^15^N), are gathered in set 1, which is shown in Figure 1.

**Figure 2:**
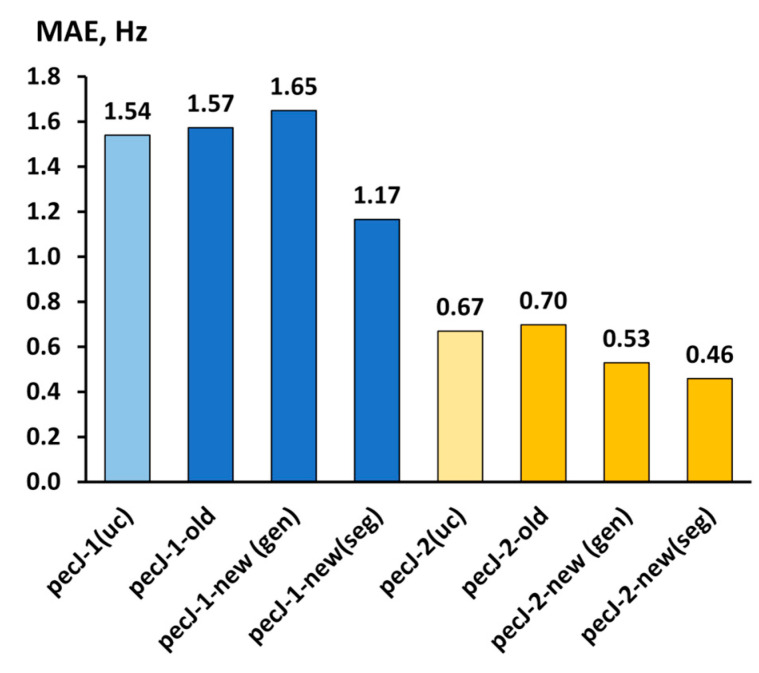
MAEs (in Hz) for all the theoretical SSCCs of the molecules of set 1 obtained at the SOPPA(CCSD) level with the old, new, and the fully uncontracted versions of the pecJ-*n* (*n* = 1, 2) basis sets for H, C, N, and F, evaluated against the ccJ-pV5Z results.

For each basis set version, the mean absolute error (MAE) against the ccJ-pV5Z data has been calculated. These MAEs are presented in Figure 2. The values of the SSCCs of the molecules of set 1 calculated with the ccJ-pV5Z and various versions of the pecJ-*n* basis sets are given in Tables S3–S9 of the Supplementary Material.

As can be seen from Figure 2, the PEC algorithm, as applied to the optimization of the contraction coefficients for the pecJ-*n* (*n* = 1, 2) basis set, does perform very well. Indeed, it has not only allowed the avoidance of the wrecking of the basis sets’ flexibility, but also propelled the accuracy to become even better than it was in the older or even in the fully uncontracted versions. Generally, the MAE of the results obtained with the pecJ-1-new(gen) is only slightly larger than that of the results obtained with the pecJ-1-old. At the same time, the SSCCs calculated with the pecJ-1-new(seg) turned out to be significantly better in accuracy than those calculated with the pecJ-1-old (cf. the MAE of 1.17 Hz for the former vs. the MAE of 1.57 Hz for the latter).

The results obtained with the pecJ-2-new(gen) and pecJ-2-new(seg) both represent the improvement over the results obtained with the pecJ-2-old basis set, implying a noticeable decrease of the MAE by 0.17 Hz for the pecJ-2-new(gen) and by 0.24 Hz for the pecJ-2-new(seg), relative to the MAE provided by the pecJ-2-old.

Provided that for both cardinal numbers *n*, the segmented contraction scheme performed considerably better than the general one and even showed superiority over the uncontracted variant, we have chosen the segmented contracted pecJ-*n*-new(seg) basis sets to represent the final versions of the pecJ-*n* basis sets. Thus, from this moment on, they will be referred to as the pecJ-*n*-new. The pecJ-*n*-new are given both in Appendix A at the end of the paper and in the Supplementary Material File.

It is remarkable that the final pecJ-*n*-new basis sets were more accurate than their uncontracted analogies. This reflects the fact that our original pecJ-*n* basis sets were not tailored in an entirely perfect way. To be more precise, the important feature of the original pecJ-*n* basis sets consists of the fact that their exponents were optimized on a very limited number of selected SSCCs for each atom, particularly using only one homonuclear one-bond SSCC of a single fitting molecule for each case. The PEC-based contraction procedure that was applied in this work has alleviated this disadvantage by involving several homonuclear SSCCs of a number of fitting molecules, with a varying number of intervening chemical bonds between the coupled nuclei. This endowed the newly contracted basis sets with more robustness towards a variety of electronic systems relative to that which was inherent in their uncontracted forms. In result, the MAEs evaluated over a large number of the SSCCs of different types for a great variety of electronic systems were noticeably lower for the data obtained with the newly contracted basis sets than for the values obtained with the uncontracted basis sets.

We have also distinguished the MAEs evaluated over different types of SSCCs. These actually vary considerably, depending on the SSCC type. The results are gathered in Table 2, which contains the MAEs for eight SSCC species, for which it was possible to make statistically solid estimations. The corresponding ranges of variation of the selected types of SSCCs of set 1 are also given in the second column of Table 2.

As follows from Figure 2, an improvement of the newly segmented-contracted basis sets over their old versions is apparent, and yet another question could be asked: how well do the pecJ-*n*-new (*n* = 1, 2) basis sets work in comparison with the other well-known J-oriented basis sets of the same zeta-level? To answer this question, we have considered the performance of the pecJ-*n*-new (*n* = 1, 2) basis sets against the performance of several of the most popular J-oriented basis sets of double- and triple-zeta quality, including the pcJ-*n* (*n* = 1, 2) [37], ccJ-pVXZ (X = D, T) [39], and the aug-cc-pVTZ-J [11,33], in the SOPPA(CCSD) calculations of all SSCCs in the molecules of set 1.

Actually, each of the J-oriented basis sets to be compared with ours in this testing has its own peculiarities, but the common feature that is shared by all of them consists of the principle of their contraction. Namely, the contraction coefficients for the pcJ-*n* (*n* = 1, 2), ccJ-pVXZ (X = D, T), and the aug-cc-pVTZ-J were obtained based on the atomic/molecular orbital coefficients resulting from the energy minimization problem of atoms/simple molecules. Most importantly, none of the three basis set series have the first polarization shell contracted. The mentioned characteristics define the main distinction of the contraction principle of the pecJ-*n*-new basis sets from those applied to the other J-oriented basis sets.

**Figure 3:**
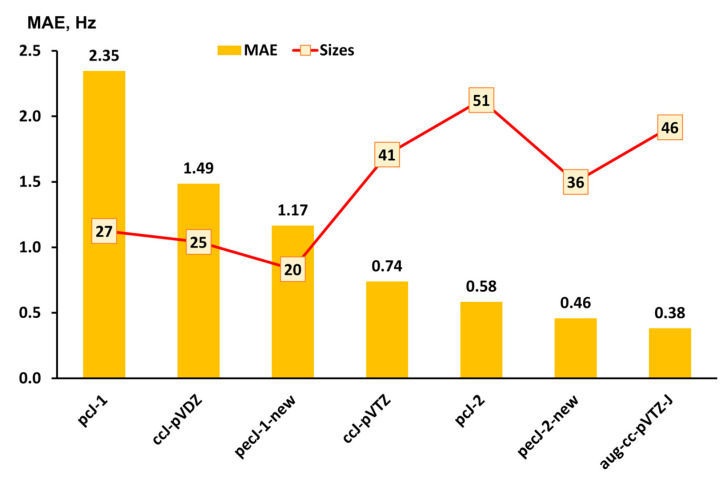
The MAEs for all theoretical SSCCs of the molecules of set 1 obtained at the SOPPA(CCSD) level with the pcJ-*n* (*n* = 1, 2), ccJ-pVXZ (X = D, T), pecJ-*n*-new (*n* = 1, 2) and aug-cc-pVTZ-J basis sets, evaluated against the ccJ-pV5Z results.

Like in the previous test, the performance of the considered basis sets was estimated on the same set of molecules using the same SOPPA(CCSD)/ccJ-pV5Z values as the reference data. The evaluated MAEs are given in Figure 3. The red line with yellow squares in Figure 3 shows the total number of functions included in a given basis set. The values of the SSCCs in the molecules of set 1 calculated with the pcJ-*n* (*n* = 1, 2), ccJ-pVXZ (X = D, T), and aug-cc-pVTZ-J are presented in Tables S10–S14, respectively, in the Supplementary Material.

As can be seen from Figure 3, the pecJ-1-new basis set shows a considerable superiority in accuracy as compared to the other double-zeta basis sets of commensurable sizes (cf. MAEs of 2.35, 1.49, and 1.17 Hz for the pcJ-1, ccJ-pVDZ, and pecJ-1-new basis sets, respectively). At the same time, the pecJ-1-new basis set contains accordingly seven and five functions fewer than the pcJ-1 and ccJ-pVDZ basis sets do.

Among the basis sets of triple-zeta quality, one can see that the pecJ-2-new basis set provides the second-best accuracy after the aug-cc-pVTZ-J basis set, resulting in a MAE of only 0.46 Hz. In fact, the pecJ-2-new is the smallest basis set among all the other triple-zeta basis sets considered in this test. Additionally, the difference between the pecJ-2-new basis set and the other triple-zeta-quality J-oriented basis sets is a matter of 5–15 functions, depending on the basis set.

Although the pecJ-*n*-new (*n* = 1, 2) basis sets were created at the SOPPA(CCSD) level, they were also checked within the DFT approach. In this test, all 436 SSCCs of the molecules in set 1 were calculated within the DFT method in combination with the PBE0 exchange–correlation functional [57,58,59] using the pcJ-*n* (*n* = 1, 2), ccJ-pVXZ (X = D, T), pecJ-*n*-new (*n* = 1, 2), and aug-cc-pVTZ-J basis sets, against the reference values of the corresponding SSCCs calculated at the same level of theory, the DFT(PBE0), with the ccJ-pV5Z basis set. The corresponding MAEs provided by the pecJ-1-new and pecJ-2-new basis sets are 1.29 and 0.47 Hz, respectively. The MAEs obtained with the other first-level basis sets, the ccJ-pVDZ and pcJ-1, are 1.73 and 1.43 Hz, respectively, while the MAEs for the second-level basis sets, the ccJ-pVTZ, pcJ-2, and aug-cc-pVTZ-J, accordingly, are 0.73, 0.56, and 0.42 Hz. As can be concluded from these figures, the tendencies of the basis set errors within the DFT(PBE0) and SOPPA(CCSD) methods are almost the same.

Considering these results and the results of our previous works dealing with the other molecular properties, the question of whether or not the PEC method is good for the optimization of the contraction coefficients for property-oriented basis sets is no more. This method perfectly suits such kinds of problems, especially when one wants to considerably reduce the size of a given property-oriented basis set on account of the first polarization shell.

### 2.3. Testing the Newly Contracted pecJ-n (n = 1, 2) Basis Sets for H, C, N, and F Against Experiment

The final judgment of any theoretical achievement is the comparison of the theoretical results with an experiment. Therefore, in this section, we have selected a series of small but challenging symmetric molecules with available gas phase experimental NMR data, and performed the calculations of the SSCCs in these molecules with the pecJ-*n*-new (*n* = 1, 2) basis sets within the CCSDT scheme, taking into account the vibrational corrections within the CCSD scheme. The series of the selected molecules (designated here as set 2) included the following systems: CH_4_, C_2_H_2_, C_2_H_4_, C_2_H_6_, CH_3_F, and CH_3_NH_2_. These molecules cover most of the possible types of SSCCs between H, C, N and F nuclei, and their gas phase experimental values are readily accessible [60,61,62,63,64]. It is worth mentioning that it was the compactness of the pecJ-*n*-new (*n* = 1, 2) basis sets that made it feasible for us to carry out the calculations of the SSCCs within the CCSDT scheme, which represents an extraordinary computationally demanding CC approximation with a formal scaling factor of *N*^8^.

The zero-point vibrational corrections (ZPVCs) were calculated within the CCSD scheme, within the second-order vibrational perturbation theory (VPT2) [65], employing the smallest of the two basis sets, the pecJ-1-new.

The calculated SSCCs (breaking down the total values into four different contributions) are presented in Table 3.

Table 3 demonstrates good agreement between the theoretical data obtained with both the pecJ-*n*-new (*n* = 1, 2) basis sets with the experiment, with the small deviations being within the range of only a few Hz. In this way, the pecJ-*n*-new (*n* = 1, 2) basis sets provide a physical opportunity to perform extensive high-quality calculations of SSCCs using modern ab initio correlated quantum chemistry methods with sufficiently good accuracy. This is primarily due to their moderate sizes, considerably inferior to typical J-oriented basis sets of the same zeta-quality.

## 3. Materials and Methods

The geometry optimization of all molecules in set 1 or that of the fitting molecules participating in the PEC optimization was carried out in the gas phase at the CCSD [5] level of theory using the pecG-2 basis set [49], within the Gaussian 09 (Rev. C.01) program [66]. The geometry optimization of all molecules in set 2 was performed in the gas phase at the CCSD(T) [67] level of theory using the pecG-2 basis set in the CFOUR program [68]. All equilibrium geometries are given in Tables S1 and S2 of the Supplementary Material.

The calculations of the SSCCs at the SOPPA(CCSD) level of theory were carried out in the Dalton program (release Dalton2022.0-dev) [69], while their calculations at the CCSDT level were performed in the MRCC module [70,71] as implemented in the CFOUR program. The vibrationally averaged values of the SSCCs were obtained at the CCSD level of theory within the VPT2 approach in the CFOUR program. The values of the calculated SSCCs of the molecules of set 1 are presented in Tables S3–S14 of the Supplementary Material, while those of the molecules of set 2 are reported in the main text.

For the basis set optimization, we have used the PEC algorithm, coded within the Python 3.097 media, and, during the optimization of the contraction coefficients, the calculations of the SSCCs were being performed at the SOPPA(CCSD) level in the Dalton program.

## 4. Conclusions

The PEC method has been shown to represent an efficient method for the contraction of property-oriented basis sets. As an example, old versions of the J-oriented pecJ-*n* (*n* = 1, 2) basis sets for H, C, N, and F atoms (published in 2021) were considered, whose contraction coefficients were found by the usual means in the original work, i.e., from the SCF calculation of the molecular energy of the simplest hydrides. In this work, the original pecJ-*n* basis sets were recontacted by means of the PEC method, providing the possibility to reach a greater contraction depth. Indeed, the newly contracted basis sets, the pecJ-*n*-new, include a smaller number of basis set functions as compared to their old versions, namely, four and seven less for hydrogen and the 2nd-period atoms, respectively, for both cardinal numbers *n* = 1, 2. Such considerable contraction has become possible due to the contraction of the first polarization shell, which represents a rather unusual and portentous maneuver that was not applied when generating the previous versions of the pecJ-*n* basis sets. Thus, new segmented and generally contracted versions of the pecJ-1 and pecJ-2 basis sets were obtained.

The performances of all versions of the pecJ-*n* (*n* = 1, 2) basis sets, including the originally contracted ones, fully uncontracted ones, and the newly contracted ones, were compared with each other and with the performance of the other popular J-oriented basis sets, namely, the pcJ-*n* (*n* = 1, 2), ccJ-pVXZ (X = D, T), and aug-cc-pVTZ-J basis sets, as assessed against the theoretical reference data obtained with the ccJ-pV5Z basis set. The examination was carried out on the SSCCs of different types of 41 molecules, including ^1^H-^1^H, ^13^C-^1^H, ^13^C-^13^C, ^19^F-^13^C, ^19^F-^1^H, ^19^F-^19^F, ^15^N-^1^H, ^15^N-^13^C, ^19^F-^15^N, and ^15^N-^15^N SSCCs (totaling in number to 436), calculated at the SOPPA(CCSD) level. This showed that the new segmented versions of the pecJ-*n* basis sets perform considerably better than the generally contracted versions do, leaving behind not only the original contracted pecJ-*n* and the pcJ-*n* and ccJ-pVXZ basis sets of the corresponding zeta-quality but also their fully uncontracted analogies. The MAEs for the SSCCs obtained with the pecJ-1 and pecJ-2 basis sets against the theoretical reference values are equal to 1.17 and 0.46 Hz, respectively. Thus, the segmented contracted versions of the pecJ-*n* (*n* = 1, 2) basis sets were recommended as the final ones.

The last test for the newly contracted pecJ-*n*-new (*n* = 1, 2) basis sets has been conducted using the gas phase experimental values as the reference, and this can be regarded as an austere verification of the aptness of the newly proposed basis sets. In this regard, the calculations of several types of SSCCs involving the H, C, N and F nuclei of six small symmetric molecules have been performed within the CCSDT method, taking into account the vibrational corrections within the CCSD scheme. The obtained theoretical results were of sufficiently high accuracy, providing a good agreement with experiment.

Thus, the PEC method has been proven to be suitable for defining accurate contraction coefficients of property-oriented basis sets, especially when one uses several fitting molecules (the more the better) and very precise target values in the PEC optimization.

## Figures and Tables

**Table 1:** Structures of the old and new versions of the pecJ-*n* (*n* = 1, 2) basis sets.

Element	Basis Set	Structure	Contraction Scheme	$N_{uc}$ $/N_{c}$	Contraction Depth, %
H	pecJ-1-old	[7s2p|5s2p]	7s → (3,1,1,1,1) 2p →(1,1)	13/11	15
	pecJ-2-old	[8s3p1d|6s3p1d]	8s → (3,1,1,1,1,1) 3p →(1,1,1) 1d →(1)	22/20	9
	pecJ-1-new(seg)	[7s2p|4s1p]	7s →(3,2,1,1) 2p →(1)	13/7	46
	pecJ-2-new(seg)	[8s3p1d|5s2p1d]	8s →(3,2,1,1,1) 3p →(2,1) 1d →(1)	22/16	27
C, N, F	pecJ-1-old	[10s5p2d|8s3p2d]	10s →(3,1,1,1,1,1,1,1) 5p →(3,1,1) 2d →(1,1)	35/27	23
	pecJ-2-old	[11s6p3d1f|9s4p3d1f]	11s →(3,1,1,1,1,1,1,1,1) 6p →(3,1,1,1) 3d →(1,1,1) 1f →(1)	51/43	16
	pecJ-1-new(gen), pecJ-1-new(seg)	[10s5p2d|6s3p1d]	General: 10s →(6,6,1,1,1,1) 5p →(3,1,1) 2d →(1)	35/20	43
			Segmented: 10s →(3,2,2,1,1,1) 5p →(3,1,1) 2d →(1)		
	pecJ-2-new(gen), pecJ-2-new(seg),	[11s6p3d1f|7s4p2d1f]	General: 11s →(6,6,1,1,1,1,1) 6p →(3,1,1,1) 3d →(2,1) 1f →(1)	51/36	29
			Segmented: 11s →(3,2,2,1,1,1,1) 6p →(3,1,1,1) 3d →(2,1) 1f →(1)		

**Table 2:** The MAEs for different types of SSCCs (in Hz) in the molecules of set 1 calculated at the SOPPA(CCSD) level with the pecJ-*n*-new (*n* = 1, 2) basis sets against the reference theoretical values obtained with the ccJ-pV5Z basis set.

Type of SSCC	Range of SSCCs	Number of SSCCs	MAE for pecJ-1-New	MAE for pecJ-2-New
*J*(^1^H,^1^H)	−20 to +38	96	0.63	0.19
*J*(^13^C,^1^H)	−13 to 281	148	0.88	0.41
*J*(^13^C,^13^C)	−7 to 414	54	0.84	0.44
*J*(^19^F,^13^C)	−405 to 66	29	3.80	0.94
*J*(^19^F,^1^H)	−5 to 550	20	3.28	1.35
*J*(^19^F,^19^F)	−134 to 322	13	4.47	1.94
*J*(^15^N,^1^H)	−107 to 4	39	0.57	0.23
*J*(^15^N,^13^C)	−19 to 3	25	0.46	0.17

**Table 3:** The SSCCs (in Hz) of different types in the molecules of set 2 calculated at the CCSDT level of theory with the pecJ-*n*-new (*n* = 1, 2) basis sets, taking into account ZPVCs, against the reference experimental gas phase data.

Molecule	Type of SSCC	*n* ^1^	*J*_FC_^2^	*J*_SD_^3^	*J*_PSO_^4^	*J*_DSO_^5^	*J_eq_* ^6^	Δ*J_vib_* ^7^	*J_tot_* ^8^	Exp. ^9^
CH_4_	^1^*J*(^13^C,^1^H)	1	118.16	0.03	1.54	0.28	120.01	4.63	124.64	125.31 **^9a^**
		2	117.78	−0.01	1.55	0.26	119.58		124.21	
C_2_H_2_	^1^*J*(^13^C,^13^C)	1	170.53	8.53	6.99	0.02	186.07	−9.81	176.26	174.78 **^9b^**
		2	170.08	8.46	6.82	0.02	185.38		175.57	
	^1^*J*(^13^C, ^1^H)	1	242.52	0.41	−0.49	0.36	242.80	2.96	245.76	247.56 **^9b^**
		2	242.71	0.31	−0.75	0.31	242.58		245.54	
	^2^*J*(^13^C,^1^H)	1	46.52	0.64	5.36	−1.34	51.18	−3.42	47.76	50.14 **^9b^**
		2	48.07	0.98	5.61	−1.35	53.31		49.89	
	^3^*J*(^1^H,^1^H)	1	9.21	0.55	2.4	−3.59	8.57	−0.09	8.48	9.62 **^9b^**
		2	8.41	0.56	2.82	−3.61	8.18		8.09	
C_2_H_4_	^1^*J*(^13^C,^13^C)	1	74.14	2.86	−9.11	0.07	67.96	1.06	69.02	67.92 **^9c^**
		2	73.80	2.69	−9.04	0.07	67.52		68.58	
	^1^*J*(^13^C,^1^H)	1	149.09	0.12	0.59	0.48	150.28	4.2	154.48	156.03 **^9c^**
		2	149.35	0.06	0.46	0.44	150.31		154.51	
	^2^*J*(^13^C,^1^H)	1	−0.69	−0.03	−1.12	−0.69	−2.53	−1.22	−3.75	−2.55 **^9c^**
		2	−0.23	0.08	−1.02	−0.69	−1.86		−3.08	
	^2^*J*(^1^H,^1^H)	1	0.71	0.22	3.68	−3.83	0.78	−0.01	0.77	2.53 **^9c^**
		2	1.05	0.33	4.09	−3.88	1.59		1.58	
	^3^*J_cis_*(^1^H,^1^H)	1	11.71	−0.04	0.59	−1.06	11.20	0.64	11.84	11.81 **^9c^**
		2	11.59	−0.05	0.74	−1.08	11.20		11.84	
	^3^*J_trans_*(^1^H,^1^H)	1	18.36	0.25	2.73	−3.57	17.77	1.74	19.51	19.18 **^9c^**
		2	17.96	0.22	3.11	−3.59	17.70		19.44	
C_2_H_6_	^1^*J*(^13^C,^13^C)	1	32.14	1.02	0.14	0.11	33.41	1.92	35.33	35.00 **^9c^**
		2	31.56	1.03	0.18	0.11	32.88		34.80	
	^1^*J*(^13^C,^1^H)	1	117.93	−0.01	1.28	0.52	119.72	4.21	123.93	124.98 **^9c^**
		2	117.96	−0.05	1.23	0.50	117.96		123.85	
	^2^*J*(^13^C,^1^H)	1	−4.8	0.06	0.32	−0.35	−4.77	−0.65	−5.42	−4.79 **^9c^**
		2	−4.52	0.07	0.40	−0.35	−4.40		−5.05	
	^3^*J*(^1^H,^1^H)	1	7.66	0.06	1.43	−1.66	7.49	0.76	8.25	8.08 **^9c^**
		2	7.63	0.05	1.61	−1.68	7.61		8.37	
CH_3_F	^1^*J*(^19^F,^13^C)	1	−217.24	19.20	35.03	0.43	−162.58	−2.59	−165.17	−163.00 **^9d^**
		2	−220.27	19.65	34.88	0.39	−165.35		−167.94	
	^1^*J*(^13^C,^1^H)	1	139.73	0.01	0.05	0.67	140.46	5.15	145.61	147.25 **^9d^**
		2	139.22	−0.01	−0.11	0.66	139.76		144.91	
	^2^*J*(^19^F,^1^H)	1	40.86	−2.84	12.48	−1.88	48.62	−1.52	47.10	46.47 **^9d^**
		2	42.00	−3.00	12.64	−1.93	49.71		48.19	
CH_3_NH_2_	^1^*J*(^15^N,^1^H)	1	−61.66	−0.07	−2.96	−0.22	−64.91	0.45	−64.46	−65.41 **^9e^**
		2	−60.42	−0.13	−2.75	−0.20	−63.50		−63.05	
	^1^*J*(^13^C,^1^H)	1	124.42	−0.04	0.84	0.58	125.8	4.67	130.47	132.51 **^9e^**
		2	124.71	−0.07	0.75	0.56	125.95		130.62	
	^1^*J*(^15^N,^13^C)	1	−3.87	−0.73	−0.43	−0.05	−5.08	0.14	−4.94	−5.45 **^9e^**
		2	−3.41	−0.71	−0.47	−0.05	−4.64		−4.50	
	^3^*J*(^1^H,^1^H)	1	6.81	0.05	1.84	−2.14	6.56	0.35	6.91	7.01 **^9e^**
		2	6.70	0.05	2.10	−2.16	6.69		7.04	

**^1^** Numbers *n* = 1, 2 designate the cardinal numbers of the pecJ-*n*-new basis sets; **^2^** *J*_FC_ is the FC contribution; **^3^** *J*_SD_ is the SD contribution; **^4^** *J*_PSO_ is the PSO contribution; **^5^** *J*_DSO_ is the DSO contribution; **^6^** *J_eq_* represents the total SSCC calculated on equilibrium geometry, *J_eq_* = *J*_FC_ + *J*_SD_ + *J*_PSO_ + *J*_DSO_; **^7^** Δ*J_vib_* is the ZPVC to SSCC; **^8^** *J_tot_* represents the final theoretical value, *J_tot_* = *J_eq_* + Δ*J_vib_*; **^9^** experimental values were taken from the following works: **^9a^** ref. [60]; **^9b^** ref. [61]; **^9c^** ref. [62]; **^9d^** ref. [63]; **^9e^** ref. [64].

## Data Availability

The original contributions presented in this study are included in the article/Supplementary Material. Further inquiries can be directed to the corresponding authors.

## Supplementary Materials

The following supporting information can be downloaded at: https://www.mdpi.com/article/10.3390/ijms27104650/s1.

## Appendix A

In this section, the pecJ-*n*-new(seg) (*n* = 1, 2) basis sets for H, C, N, and F atoms are given in the Dalton format. These represent the final recommended versions; therefore, in order to avoid confusing them with the older versions, their names should be mentioned as the pecJ-*n* (*n* = 1, 2) basis sets with indication of their version (the version of 2026).

**The pecJ-1 for H**

$ H

a 1

$ s functions

   7  4  0

   5.07308E+03  2.2082E-03  0.00000000  0.00000000  0.00000000

   2.16644E+02  4.6279E-02  0.00000000  0.00000000  0.00000000

   2.50008E+01  4.9109E-01  0.00000000  0.00000000  0.00000000

   5.00205E+00  0.00000000  3.4393E-02  0.00000000  0.00000000

   1.29556E+00  0.00000000  1.3877E-01  0.00000000  0.00000000

   4.00307E-01  0.00000000  0.00000000  1.00000000  0.00000000

   1.33227E-01  0.00000000  0.00000000  0.00000000  1.00000000

$ p functions

   2  1  0

   1.48544E+00  9.6135E-02

   4.06750E-01  1.9980E-01

**The pecJ-1 for C**

$ C

a 6

$ s functions

   10  6  0

   3.551632E+04  7.6818E-03  0.00000000  0.00000000  0.00000000  0.00000000  0.00000000

   5.744652E+03  6.7994E-02  0.00000000  0.00000000  0.00000000  0.00000000  0.00000000

   9.954157E+02  5.1820E-01  0.00000000  0.00000000  0.00000000  0.00000000  0.00000000

   2.315943E+02  0.00000000  2.6261E-01  0.00000000  0.00000000  0.00000000  0.00000000

   6.620654E+01  0.00000000  9.9015E-01  0.00000000  0.00000000  0.00000000  0.00000000

   2.157261E+01  0.00000000  0.00000000  2.6227E-01  0.00000000  0.00000000  0.00000000

   7.648720E+00  0.00000000  0.00000000  4.4212E-01  0.00000000  0.00000000  0.00000000

   2.844272E+00  0.00000000  0.00000000  0.00000000  1.00000000  0.00000000  0.00000000

   6.482868E-01  0.00000000  0.00000000  0.00000000  0.00000000  1.00000000  0.00000000

   2.179306E-01  0.00000000  0.00000000  0.00000000  0.00000000  0.00000000  1.00000000

$ p functions

   5  3  0

   3.053233E+01  4.2463E-03  0.00000000  0.00000000

   6.903835E+00  3.0380E-02  0.00000000  0.00000000

   1.882246E+00  1.2368E-01  0.00000000  0.00000000

   5.969291E-01  0.00000000  1.00000000  0.00000000

   1.734469E-01  0.00000000  0.00000000  1.00000000

$ d functions

   2  1  0

   1.234913E+00  2.0744E-01

   3.853405E-01  2.5437E-01

**The pecJ-1 for N**

$ N

a 7

$ s functions

   10  6  0

   1.649023E+05  4.1857E-05  0.00000000  0.00000000  0.00000000  0.00000000  0.00000000

   9.215658E+03  5.9857E-04  0.00000000  0.00000000  0.00000000  0.00000000  0.00000000

   1.455967E+03  3.7819E-03  0.00000000  0.00000000  0.00000000  0.00000000  0.00000000

   3.238329E+02  0.00000000  2.4503E-02  0.00000000  0.00000000  0.00000000  0.00000000

   8.920075E+01  0.00000000  9.6591E-02  0.00000000  0.00000000  0.00000000  0.00000000

   2.841262E+01  0.00000000  0.00000000  2.6578E-01  0.00000000  0.00000000  0.00000000

   1.001470E+01  0.00000000  0.00000000  4.3032E-01  0.00000000  0.00000000  0.00000000

   3.764414E+00  0.00000000  0.00000000  0.00000000  1.00000000  0.00000000  0.00000000

   7.224090E-01  0.00000000  0.00000000  0.00000000  0.00000000  1.00000000  0.00000000

   2.026552E-01  0.00000000  0.00000000  0.00000000  0.00000000  0.00000000  1.00000000

$ p functions

   5  3  0

   3.123160E+01  9.4162E-03  0.00000000  0.00000000

   6.893201E+00  4.9086E-02  0.00000000  0.00000000

   1.978571E+00  1.4805E-01  0.00000000  0.00000000

   6.571405E-01  0.00000000  1.00000000  0.00000000

   1.946419E-01  0.00000000  0.00000000  1.00000000

$ d functions

   2  1  0

   1.411561E+00  2.4444E-01

   3.724804E-01  2.1075E-01

**The pecJ-1 for F**

$ F

a 9

$ s functions

   10  6  0

   2.282510E+05  1.9333E-03  0.00000000  0.00000000  0.00000000  0.00000000  0.00000000

   1.395200E+04  8.3095E-02  0.00000000  0.00000000  0.00000000  0.00000000  0.00000000

   2.238199E+03  7.5509E-01  0.00000000  0.00000000  0.00000000  0.00000000  0.00000000

   5.097673E+02  0.00000000  2.2165E-02  0.00000000  0.00000000  0.00000000  0.00000000

   1.430299E+02  0.00000000  8.5661E-02  0.00000000  0.00000000  0.00000000  0.00000000

   4.604822E+01  0.00000000  0.00000000  3.1930E-01  0.00000000  0.00000000  0.00000000

   1.633767E+01  0.00000000  0.00000000  5.0025E-01  0.00000000  0.00000000  0.00000000

   6.221429E+00  0.00000000  0.00000000  0.00000000  1.00000000  0.00000000  0.00000000

   1.267895E+00  0.00000000  0.00000000  0.00000000  0.00000000  1.00000000  0.00000000 

   3.612004E-01   0.00000000  0.00000000  0.00000000  0.00000000  0.00000000  1.00000000

$ p functions

   5  3  0

   9.142507E+01  6.1012E-03  0.00000000  0.00000000 

   1.727983E+01  7.5734E-02  0.00000000  0.00000000

   4.618007E+00  2.9003E-01  0.00000000  0.00000000

   1.355099E+00  0.00000000  1.00000000  0.00000000

   3.034276E-01  0.00000000  0.00000000  1.00000000

$ d functions

   2  1  0

   2.207200E+00  2.5247E-01

   4.610628E-01  1.4322E-01

**The pecJ-2 for H**

$ H

a 1

$ s functions

   8  5  0

   3.26942E+04  1.1521E-03  0.00000000  0.00000000  0.00000000  0.00000000

   3.09626E+03  2.7651E-02  0.00000000  0.00000000  0.00000000  0.00000000

   1.76078E+02  6.3149E-01  0.00000000  0.00000000  0.00000000  0.00000000

   2.25414E+01  0.00000000  7.7960E-02  0.00000000  0.00000000  0.00000000

   4.68174E+00  0.00000000  4.0720E-01  0.00000000  0.00000000  0.00000000

   1.25964E+00  0.00000000  0.00000000  1.00000000  0.00000000  0.00000000

   3.88875E-01  0.00000000  0.00000000  0.00000000  1.00000000  0.00000000 

   1.26668E-01  0.00000000  0.00000000  0.00000000  0.00000000  1.00000000

$ p functions

   3  2  0

   2.35117E+00  3.5097E-02  0.00000000

   7.73294E-01  1.8498E-01  0.00000000

   2.79310E-01  0.00000000  1.00000000

$ d functions

   1  1  0

   1.07027E+00  1.00000000

**The pecJ-2 for C**

$ C

a 6

$ s functions

   11  7  0

   1.620383E+05  2.0755E-03  0.00000000  0.00000000  0.00000000  0.00000000  0.00000000  0.00000000

   8.803354E+03  4.4843E-02  0.00000000  0.00000000  0.00000000  0.00000000  0.00000000  0.00000000

   1.333357E+03  3.5203E-01  0.00000000  0.00000000  0.00000000  0.00000000  0.00000000  0.00000000

   3.008000E+02  0.00000000  1.8823E-01  0.00000000  0.00000000  0.00000000  0.00000000  0.00000000

   8.511667E+01  0.00000000  7.3105E-01  0.00000000  0.00000000  0.00000000  0.00000000  0.00000000

   2.752030E+01  0.00000000  0.00000000  2.1511E-01  0.00000000  0.00000000  0.00000000  0.00000000

   9.731687E+00  0.00000000  0.00000000  4.1388E-01  0.00000000  0.00000000  0.00000000  0.00000000

   3.570774E+00  0.00000000  0.00000000  0.00000000  1.00000000  0.00000000  0.00000000  0.00000000

   8.102360E-01  0.00000000  0.00000000  0.00000000  0.00000000  1.00000000  0.00000000  0.00000000 

   3.593953E-01  0.00000000  0.00000000  0.00000000  0.00000000  0.00000000  1.00000000  0.00000000 

   1.356139E-01  0.00000000  0.00000000  0.00000000  0.00000000  0.00000000  0.00000000  1.00000000

$ p functions

   6  4  0

   9.495948E+01  8.0674E-03  0.00000000  0.00000000  0.00000000 

   1.778687E+01  9.0636E-02  0.00000000  0.00000000  0.00000000 

   4.534333E+00  5.0283E-01  0.00000000  0.00000000  0.00000000 

   1.336460E+00  0.00000000  1.00000000  0.00000000  0.00000000 

   4.353544E-01  0.00000000  0.00000000  1.00000000  0.00000000 

   1.348775E-01  0.00000000  0.00000000  0.00000000  1.00000000

$ d functions

   3  2  0

   6.136738E+00  3.7772E-02  0.00000000 

   1.157001E+00  3.3463E-01  0.00000000 

   3.542131E-01  0.00000000  1.00000000

$ f functions

   1  1  0

   7.838496E-01  1.00000000

**The pecJ-2 for N**

$ N

a 7

$ s functions

   11  7  0

   2.079517E+05  2.0364E-03  0.00000000  0.00000000  0.00000000  0.00000000  0.00000000  0.00000000 

   1.292159E+04  4.5289E-02  0.00000000  0.00000000  0.00000000  0.00000000  0.00000000  0.00000000 

   1.817985E+03  3.8615E-01  0.00000000  0.00000000  0.00000000  0.00000000  0.00000000  0.00000000 

   4.082074E+02  0.00000000  1.9107E-01  0.00000000  0.00000000  0.00000000  0.00000000  0.00000000 

   1.167577E+02  0.00000000  7.3266E-01  0.00000000  0.00000000  0.00000000  0.00000000  0.00000000 

   3.805405E+01  0.00000000  0.00000000  2.1867E-01  0.00000000  0.00000000  0.00000000  0.00000000 

   1.337707E+01  0.00000000  0.00000000  4.3083E-01  0.00000000  0.00000000  0.00000000  0.00000000 

   4.904271E+00  0.00000000  0.00000000  0.00000000  1.00000000  0.00000000  0.00000000  0.00000000 

   1.419406E+00  0.00000000  0.00000000  0.00000000  0.00000000  1.00000000  0.00000000  0.00000000 

   5.160150E-01  0.00000000  0.00000000  0.00000000  0.00000000  0.00000000  1.00000000  0.00000000 

   1.537757E-01  0.00000000  0.00000000  0.00000000  0.00000000  0.00000000  0.00000000  1.00000000

$ p functions

   6  4  0

   5.465743E+01  3.0877E-02  0.00000000  0.00000000  0.00000000 

   1.370955E+01  1.8895E-01  0.00000000  0.00000000  0.00000000 

   4.095889E+00  7.8794E-01  0.00000000  0.00000000  0.00000000 

   1.373762E+00  0.00000000  1.00000000  0.00000000  0.00000000 

   4.870015E-01  0.00000000  0.00000000  1.00000000  0.00000000 

   1.595081E-01  0.00000000  0.00000000  0.00000000  1.00000000

$ d functions

   3  2  0

   4.573625E+00  6.4754E-03  0.00000000 

   1.031398E+00  5.3587E-02  0.00000000 

   2.690828E-01  0.00000000  1.00000000

$ f functions

   1  1  0

   1.051532E+00  1.00000000

**The pecJ-2 for F**

$ F

a 9

$ s functions

   11  7  0

   3.224350E+05  2.3702E-03  0.00000000  0.00000000  0.00000000  0.00000000  0.00000000  0.00000000 

   1.950000E+04  4.7838E-02  0.00000000  0.00000000  0.00000000  0.00000000  0.00000000  0.00000000 

   2.923000E+03  3.7620E-01  0.00000000  0.00000000  0.00000000  0.00000000  0.00000000  0.00000000 

   6.645000E+02  0.00000000  1.9949E-01  0.00000000  0.00000000  0.00000000  0.00000000  0.00000000 

   1.875000E+02  0.00000000  7.8477E-01  0.00000000  0.00000000  0.00000000  0.00000000  0.00000000 

   6.062000E+01  0.00000000  0.00000000  2.4217E-01  0.00000000  0.00000000  0.00000000  0.00000000 

   2.142000E+01  0.00000000  0.00000000  4.6199E-01  0.00000000  0.00000000  0.00000000  0.00000000 

   7.950000E+00  0.00000000  0.00000000  0.00000000  1.00000000  0.00000000  0.00000000  0.00000000 

   2.257000E+00  0.00000000  0.00000000  0.00000000  0.00000000  1.00000000  0.00000000  0.00000000 

   8.815000E-01  0.00000000  0.00000000  0.00000000  0.00000000  0.00000000  1.00000000  0.00000000 

   3.041000E-01  0.00000000  0.00000000  0.00000000  0.00000000  0.00000000  0.00000000  1.00000000

$ p functions

   6  4  0

   2.846860E+02  9.4644E-04  0.00000000  0.00000000  0.00000000 

   4.388000E+01  1.5278E-02  0.00000000  0.00000000  0.00000000 

   9.926000E+00  9.8425E-02  0.00000000  0.00000000  0.00000000 

   2.930000E+00  0.00000000  1.00000000  0.00000000  0.00000000 

   9.132000E-01  0.00000000  0.00000000  1.00000000  0.00000000 

   2.672000E-01  0.00000000  0.00000000  0.00000000  1.00000000

$ d functions

   3  2  0

   4.106940E+00  1.2685E-02  0.00000000 

   1.193130E+00  1.1768E-01  0.00000000 

   1.988930E-01  0.00000000  1.00000000

$ f functions

   1  1  0

   1.891980E+00  1.00000000
