# Supplementary material for: An Efficient Method for Contraction of Property-Oriented Basis Sets: A Considerable Reduction of the pecJ-1 and pecJ-2 Basis Sets for the Calculations of Spin–Spin Coupling Constants Involving H, C, N and F Nuclei
Source: Int J Mol Sci. 2026 May 21;27(10):4650. doi: 10.3390/ijms27104650 (PMC13206858; doi:10.3390/ijms27104650)
Supplement: Supplementary file 1 [file ijms-27-04650-s001.zip › ijms-4333370-supplementary.pdf]

Supplementary Material for Article:

An efficient method for contraction of the property-oriented basis sets: A considerable reduction of the pecJ-1 and pecJ-2 basis sets for the calculations of spin-spin coupling constants involving H, C, N and F nuclei

Irina L. Rusakova [1,*], Yuriy Yu. Rusakov [1]

[1] A. E. Favorsky Irkutsk Institute of Chemistry, Siberian Branch of the Russian Academy of Sciences, Favorsky St. 1, 664033 Irkutsk, Russia; rusakov82@mail.ru
* Correspondence: i-rusakova@bk.ru

Contents

The pecJ-*n*-new(gen/seg) (*n* = 1, 2) basis sets for H, C, N, F atoms in Dalton format:

The pecJ-1-new(seg) for H S3

The pecJ-1-new(seg) for C S3

The pecJ-1-new(seg) for N S4

The pecJ-1-new(seg) for F S5

The pecJ-1-new(gen) for C S6

The pecJ-1-new(gen) for N S7

The pecJ-1-new(gen) for F S9

The pecJ-2-new(seg) for H S10

The pecJ-2-new(seg) for C S10

The pecJ-2-new(seg) for N S12

The pecJ-2-new(seg) for F S13

The pecJ-2-new(gen) for C S14

The pecJ-2-new(gen) for N S16

The pecJ-2-new(gen) for F S17

Table S1. Cartesian coordinates for equilibrium geometries of molecules in set 1, calculated in the gas phase at the CCSD/pecG-2 level of theory S18

Table S2. Z-matrices for equilibrium geometries of molecules in set 2, calculated in the gas phase at the CCSD(T)/pecG-2 level of theory S24

Table S3. Reference values of SSCC in molecules of set 1 calculated at the SOPPA(CCSD) level with the ccJ-pV5Z basis set... S26

Table S4. Symmetry independent values of SSCC (in Hz) in molecules of set 1 calculated at the SOPPA(CCSD) level with the pecJ-1-old basis set. S37

Table S5. Symmetry independent values of SSCC (in Hz) in molecules of set 1 calculated at the SOPPA(CCSD) level with the pecJ-2-old basis set. S47

Table S6. Symmetry independent values of SSCC (in Hz) in molecules of set 1 calculated at the SOPPA(CCSD) level with the pecJ-1-new(gen) basis set. S57

Table S7. Symmetry independent values of SSCC (in Hz) in molecules of set 1 calculated at the SOPPA(CCSD) level with the pecJ-1-new(seg) basis set. S67

Table S8. Symmetry independent values of SSCC (in Hz) in molecules of set 1 calculated at the SOPPA(CCSD) level with the pecJ-2-new(gen) basis set. S77

Table S9. Symmetry independent values of SSCC (in Hz) in molecules of set 1 calculated at the SOPPA(CCSD) level with the pecJ-2-new(seg) basis set... S87

Table S10. Symmetry independent values of SSCC (in Hz) in molecules of set 1 calculated at the SOPPA(CCSD) level with the pcJ-1 basis set. S97

Table S11. Symmetry independent values of SSCC (in Hz) in molecules of set 1 calculated at the SOPPA(CCSD) level with the pcJ-2 basis set. S107

Table S12. Symmetry independent values of SSCC (in Hz) in molecules of set 1 calculated at the SOPPA(CCSD) level with the ccJ-pVDZ basis set. S117

Table S13. Symmetry independent values of SSCC (in Hz) in molecules of set 1 calculated at the SOPPA(CCSD) level with the ccJ-pVTZ basis set. S127

Table S14. Symmetry independent values of SSCC (in Hz) in molecules of set 1 calculated at the SOPPA(CCSD) level with the aug-cc-pVTZ-J basis set. S137

The pecJ-1-new(seg) for H

$ H

a 1

$ s functions

7 4 0

5.07308E+03 2.2082E-03 0.00000000 0.00000000 0.00000000

2.16644E+02 4.6279E-02 0.00000000 0.00000000 0.00000000

2.50008E+01 4.9109E-01 0.00000000 0.00000000 0.00000000

5.00205E+00 0.00000000 3.4393E-02 0.00000000 0.00000000

1.29556E+00 0.00000000 1.3877E-01 0.00000000 0.00000000

4.00307E-01 0.00000000 0.00000000 1.00000000 0.00000000

1.33227E-01 0.00000000 0.00000000 0.00000000 1.00000000

$ p functions

2 1 0

1.48544E+00 9.6135E-02

4.06750E-01 1.9980E-01

The pecJ-1-new(seg) for C

$ C

a 6

$ s functions

10 6 0

3.551632E+04 7.6818E-03 0.00000000 0.00000000 0.00000000 0.00000000 0.00000000

5.744652E+03 6.7994E-02 0.00000000 0.00000000 0.00000000 0.00000000 0.00000000

9.954157E+02 5.1820E-01 0.00000000 0.00000000 0.00000000 0.00000000 0.00000000

2.315943E+02 0.00000000 2.6261E-01 0.00000000 0.00000000 0.00000000 0.00000000

6.620654E+01 0.00000000 9.9015E-01 0.00000000 0.00000000 0.00000000 0.00000000

2.157261E+01 0.00000000 0.00000000 2.6227E-01 0.00000000 0.00000000 0.00000000

7.648720E+00 0.00000000 0.00000000 4.4212E-01 0.00000000 0.00000000 0.00000000

2.844272E+00 0.00000000 0.00000000 0.00000000 1.00000000 0.00000000 0.00000000

6.482868E-01 0.00000000 0.00000000 0.00000000 0.00000000 1.00000000 0.00000000

2.179306E-01 0.00000000 0.00000000 0.00000000 0.00000000 0.00000000 1.00000000

$ p functions

5 3 0

3.053233E+01 4.2463E-03 0.00000000 0.00000000

6.903835E+00 3.0380E-02 0.00000000 0.00000000

1.882246E+00 1.2368E-01 0.00000000 0.00000000

5.969291E-01 0.00000000 1.00000000 0.00000000

1.734469E-01 0.00000000 0.00000000 1.00000000

$ d functions

2 1 0

1.234913E+00 2.0744E-01

3.853405E-01 2.5437E-01

The pecJ-1-new(seg) for N

$ N

a 7

$ s functions

10 6 0

1.649023E+05 4.1857E-05 0.00000000 0.00000000 0.00000000 0.00000000 0.00000000

9.215658E+03 5.9857E-04 0.00000000 0.00000000 0.00000000 0.00000000 0.00000000

1.455967E+03 3.7819E-03 0.00000000 0.00000000 0.00000000 0.00000000 0.00000000

3.238329E+02 0.00000000 2.4503E-02 0.00000000 0.00000000 0.00000000 0.00000000

8.920075E+01 0.00000000 9.6591E-02 0.00000000 0.00000000 0.00000000 0.00000000

2.841262E+01 0.00000000 0.00000000 2.6578E-01 0.00000000 0.00000000 0.00000000

1.001470E+01 0.00000000 0.00000000 4.3032E-01 0.00000000 0.00000000 0.00000000

3.764414E+00 0.00000000 0.00000000 0.00000000 1.00000000 0.00000000 0.00000000

7.224090E-01 0.00000000 0.00000000 0.00000000 0.00000000 1.00000000 0.00000000

2.026552E-01 0.00000000 0.00000000 0.00000000 0.00000000 0.00000000 1.00000000

$ p functions

5 3 0

3.123160E+01 9.4162E-03 0.00000000 0.00000000

6.893201E+00 4.9086E-02 0.00000000 0.00000000

1.978571E+00 1.4805E-01 0.00000000 0.00000000

6.571405E-01 0.00000000 1.00000000 0.00000000

1.946419E-01 0.00000000 0.00000000 1.00000000

$ d functions

2 1 0

1.411561E+00 2.4444E-01

3.724804E-01 2.1075E-01

The pecJ-1-new(seg) for F

$ F

a 9

$ s functions

10 6 0

2.282510E+05 1.9333E-03 0.00000000 0.00000000 0.00000000 0.00000000 0.00000000

1.395200E+04 8.3095E-02 0.00000000 0.00000000 0.00000000 0.00000000 0.00000000

2.238199E+03 7.5509E-01 0.00000000 0.00000000 0.00000000 0.00000000 0.00000000

5.097673E+02 0.00000000 2.2165E-02 0.00000000 0.00000000 0.00000000 0.00000000

1.430299E+02 0.00000000 8.5661E-02 0.00000000 0.00000000 0.00000000 0.00000000

4.604822E+01 0.00000000 0.00000000 3.1930E-01 0.00000000 0.00000000 0.00000000

1.633767E+01 0.00000000 0.00000000 5.0025E-01 0.00000000 0.00000000 0.00000000

6.221429E+00 0.00000000 0.00000000 0.00000000 1.00000000 0.00000000 0.00000000

1.267895E+00 0.00000000 0.00000000 0.00000000 0.00000000 1.00000000 0.00000000

3.612004E-01 0.00000000 0.00000000 0.00000000 0.00000000 0.00000000 1.00000000

$ p functions

5 3 0

9.142507E+01 6.1012E-03 0.00000000 0.00000000

1.727983E+01 7.5734E-02 0.00000000 0.00000000

4.618007E+00 2.9003E-01 0.00000000 0.00000000

1.355099E+00 0.00000000 1.00000000 0.00000000

3.034276E-01 0.00000000 0.00000000 1.00000000

$ d functions

2 1 0

2.207200E+00 2.5247E-01

4.610628E-01 1.4322E-01

The pecJ-1-new(gen) for C

$ C

a 6

$ s functions

10 6 0

3.551632E+04 7.5259E-05 1.3515E-04 0.00000000 0.00000000 0.00000000 0.00000000

5.744652E+03 6.7755E-04 1.2612E-03 0.00000000 0.00000000 0.00000000 0.00000000

9.954157E+02 5.2226E-03 9.3217E-03 0.00000000 0.00000000 0.00000000 0.00000000

2.315943E+02 2.6163E-02 4.7582E-02 0.00000000 0.00000000 0.00000000 0.00000000

6.620654E+01 9.7106E-02 1.9116E-01 0.00000000 0.00000000 0.00000000 0.00000000

2.157261E+01 2.5680E-01 5.9942E-01 0.00000000 0.00000000 0.00000000 0.00000000

7.648720E+00 0.00000000 0.00000000 1.00000000 0.00000000 0.00000000 0.00000000

2.844272E+00 0.00000000 0.00000000 0.00000000 1.00000000 0.00000000 0.00000000

6.482868E-01 0.00000000 0.00000000 0.00000000 0.00000000 1.00000000 0.00000000

2.179306E-01 0.00000000 0.00000000 0.00000000 0.00000000 0.00000000 1.00000000

$ p functions

5 3 0

3.053233E+01 4.2788E-03 0.00000000 0.00000000

6.903835E+00 3.0806E-02 0.00000000 0.00000000

1.882246E+00 1.2540E-01 0.00000000 0.00000000

5.969291E-01 0.00000000 1.00000000 0.00000000

1.734469E-01 0.00000000 0.00000000 1.00000000

$ d functions

2 1 0

1.234913E+00 2.0596E-01

3.853405E-01 2.5440E-01

The pecJ-1-new(gen) for N

$ N

a 7

$ s functions

10 6 0

1.649023E+05 2.7036E-05 1.1118E-05 0.00000000 0.00000000 0.00000000 0.00000000

9.215658E+03 7.1056E-04 1.5490E-04 0.00000000 0.00000000 0.00000000 0.00000000

1.455967E+03 4.9896E-03 1.0793E-03 0.00000000 0.00000000 0.00000000 0.00000000

3.238329E+02 2.1163E-02 5.1202E-03 0.00000000 0.00000000 0.00000000 0.00000000

8.920075E+01 9.5238E-02 2.1435E-02 0.00000000 0.00000000 0.00000000 0.00000000

2.841262E+01 1.7514E-01 4.8838E-02 0.00000000 0.00000000 0.00000000 0.00000000

1.001470E+01 0.00000000 0.00000000 1.00000000 0.00000000 0.00000000 0.00000000

3.764414E+00 0.00000000 0.00000000 0.00000000 1.00000000 0.00000000 0.00000000

7.224090E-01 0.00000000 0.00000000 0.00000000 0.00000000 1.00000000 0.00000000

2.026552E-01 0.00000000 0.00000000 0.00000000 0.00000000 0.00000000 1.00000000

$ p functions

5 3 0

3.123160E+01 8.5892E-03 0.00000000 0.00000000

6.893201E+00 4.5140E-02 0.00000000 0.00000000

1.978571E+00 1.3690E-01 0.00000000 0.00000000

6.571405E-01 0.00000000 1.00000000 0.00000000

1.946419E-01 0.00000000 0.00000000 1.00000000

$ d functions

2 1 0

1.411561E+00 2.1500E-01

3.724804E-01 2.0914E-01

The pecJ-1-new(gen) for F

$ F

a 9

$ s functions

10 6 0

2.282510E+05 3.8036E-05 6.2481E-05 0.00000000 0.00000000 0.00000000 0.00000000

1.395200E+04 6.9384E-04 1.5722E-03 0.00000000 0.00000000 0.00000000 0.00000000

2.238199E+03 5.3433E-03 1.2287E-02 0.00000000 0.00000000 0.00000000 0.00000000

5.097673E+02 2.7261E-02 6.3211E-02 0.00000000 0.00000000 0.00000000 0.00000000

1.430299E+02 1.0503E-01 2.4341E-01 0.00000000 0.00000000 0.00000000 0.00000000

4.604822E+01 2.8384E-01 6.6695E-01 0.00000000 0.00000000 0.00000000 0.00000000

1.633767E+01 0.00000000 0.00000000 1.00000000 0.00000000 0.00000000 0.00000000

6.221429E+00 0.00000000 0.00000000 0.00000000 1.00000000 0.00000000 0.00000000

1.267895E+00 0.00000000 0.00000000 0.00000000 0.00000000 1.00000000 0.00000000

3.612004E-01 0.00000000 0.00000000 0.00000000 0.00000000 0.00000000 1.00000000

$ p functions

5 3 0

9.142507E+01 5.6241E-03 0.00000000 0.00000000

1.727983E+01 5.5694E-02 0.00000000 0.00000000

4.618007E+00 2.1965E-01 0.00000000 0.00000000

1.355099E+00 0.00000000 1.00000000 0.00000000

3.034276E-01 0.00000000 0.00000000 1.00000000

```
$ d functions
  2   1   0
 2.207200E+00  1.0381E-01
 4.610628E-01  3.1812E-01
```

The pecJ-2-new(seg) for H

```
$ H
a 1
$ s functions
  8   5   0
  3.26942E+04  1.1521E-03  0.00000000  0.00000000  0.00000000  0.00000000
  3.09626E+03  2.7651E-02  0.00000000  0.00000000  0.00000000  0.00000000
  1.76078E+02  6.3149E-01  0.00000000  0.00000000  0.00000000  0.00000000
  2.25414E+01  0.00000000  7.7960E-02  0.00000000  0.00000000  0.00000000
  4.68174E+00  0.00000000  4.0720E-01  0.00000000  0.00000000  0.00000000
  1.25964E+00  0.00000000  0.00000000  1.00000000  0.00000000  0.00000000
  3.88875E-01  0.00000000  0.00000000  0.00000000  1.00000000  0.00000000
  1.26668E-01  0.00000000  0.00000000  0.00000000  0.00000000  1.00000000
$ p functions
  3   2   0
  2.35117E+00  3.5097E-02  0.00000000
  7.73294E-01  1.8498E-01  0.00000000
  2.79310E-01  0.00000000  1.00000000
$ d functions
  1   1   0
  1.07027E+00  1.00000000
```

The pecJ-2-new(seg) for C

```
$ C
a 6
```

$ s functions

11 7 0

1.620383E+05 2.0755E-03 0.00000000 0.00000000 0.00000000 0.00000000 0.00000000 0.00000000

8.803354E+03 4.4843E-02 0.00000000 0.00000000 0.00000000 0.00000000 0.00000000 0.00000000

1.333357E+03 3.5203E-01 0.00000000 0.00000000 0.00000000 0.00000000 0.00000000 0.00000000

3.008000E+02 0.00000000 1.8823E-01 0.00000000 0.00000000 0.00000000 0.00000000 0.00000000

8.511667E+01 0.00000000 7.3105E-01 0.00000000 0.00000000 0.00000000 0.00000000 0.00000000

2.752030E+01 0.00000000 0.00000000 2.1511E-01 0.00000000 0.00000000 0.00000000 0.00000000

9.731687E+00 0.00000000 0.00000000 4.1388E-01 0.00000000 0.00000000 0.00000000 0.00000000

3.570774E+00 0.00000000 0.00000000 0.00000000 1.00000000 0.00000000 0.00000000 0.00000000

8.102360E-01 0.00000000 0.00000000 0.00000000 0.00000000 1.00000000 0.00000000 0.00000000

3.593953E-01 0.00000000 0.00000000 0.00000000 0.00000000 0.00000000 1.00000000 0.00000000

1.356139E-01 0.00000000 0.00000000 0.00000000 0.00000000 0.00000000 0.00000000 1.00000000

$ p functions

6 4 0

9.495948E+01 8.0674E-03 0.00000000 0.00000000 0.00000000

1.778687E+01 9.0636E-02 0.00000000 0.00000000 0.00000000

4.534333E+00 5.0283E-01 0.00000000 0.00000000 0.00000000

1.336460E+00 0.00000000 1.00000000 0.00000000 0.00000000

4.353544E-01 0.00000000 0.00000000 1.00000000 0.00000000

1.348775E-01 0.00000000 0.00000000 0.00000000 1.00000000

$ d functions

3 2 0

6.136738E+00 3.7772E-02 0.00000000

1.157001E+00 3.3463E-01 0.00000000

3.542131E-01 0.00000000 1.00000000

$ f functions

1 1 0

7.838496E-01 1.00000000

The pecJ-2-new(seg) for N

$ N

a 7

$ s functions

11 7 0

2.079517E+05 2.0364E-03 0.00000000 0.00000000 0.00000000 0.00000000 0.00000000 0.00000000

1.292159E+04 4.5289E-02 0.00000000 0.00000000 0.00000000 0.00000000 0.00000000 0.00000000

1.817985E+03 3.8615E-01 0.00000000 0.00000000 0.00000000 0.00000000 0.00000000 0.00000000

4.082074E+02 0.00000000 1.9107E-01 0.00000000 0.00000000 0.00000000 0.00000000 0.00000000

1.167577E+02 0.00000000 7.3266E-01 0.00000000 0.00000000 0.00000000 0.00000000 0.00000000

3.805405E+01 0.00000000 0.00000000 2.1867E-01 0.00000000 0.00000000 0.00000000 0.00000000

1.337707E+01 0.00000000 0.00000000 4.3083E-01 0.00000000 0.00000000 0.00000000 0.00000000

4.904271E+00 0.00000000 0.00000000 0.00000000 1.00000000 0.00000000 0.00000000 0.00000000

1.419406E+00 0.00000000 0.00000000 0.00000000 0.00000000 1.00000000 0.00000000 0.00000000

5.160150E-01 0.00000000 0.00000000 0.00000000 0.00000000 0.00000000 1.00000000 0.00000000

1.537757E-01 0.00000000 0.00000000 0.00000000 0.00000000 0.00000000 0.00000000 1.00000000

$ p functions

6 4 0

5.465743E+01 3.0877E-02 0.00000000 0.00000000 0.00000000

1.370955E+01 1.8895E-01 0.00000000 0.00000000 0.00000000

4.095889E+00 7.8794E-01 0.00000000 0.00000000 0.00000000

1.373762E+00 0.00000000 1.00000000 0.00000000 0.00000000

4.870015E-01 0.00000000 0.00000000 1.00000000 0.00000000

1.595081E-01 0.00000000 0.00000000 0.00000000 1.00000000

$ d functions

3 2 0

4.573625E+00 6.4754E-03 0.00000000

1.031398E+00 5.3587E-02 0.00000000

2.690828E-01 0.00000000 1.00000000

$ f functions

1 1 0

1.051532E+00 1.00000000

The pecJ-2-new(seg) for F

$ F

a 9

$ s functions

11 7 0

3.224350E+05 2.3702E-03 0.00000000 0.00000000 0.00000000 0.00000000 0.00000000 0.00000000

1.950000E+04 4.7838E-02 0.00000000 0.00000000 0.00000000 0.00000000 0.00000000 0.00000000

2.923000E+03 3.7620E-01 0.00000000 0.00000000 0.00000000 0.00000000 0.00000000 0.00000000

6.645000E+02 0.00000000 1.9949E-01 0.00000000 0.00000000 0.00000000 0.00000000 0.00000000

1.875000E+02 0.00000000 7.8477E-01 0.00000000 0.00000000 0.00000000 0.00000000 0.00000000

6.062000E+01 0.00000000 0.00000000 2.4217E-01 0.00000000 0.00000000 0.00000000 0.00000000

2.142000E+01 0.00000000 0.00000000 4.6199E-01 0.00000000 0.00000000 0.00000000 0.00000000

7.950000E+00 0.00000000 0.00000000 0.00000000 1.00000000 0.00000000 0.00000000 0.00000000

2.257000E+00 0.00000000 0.00000000 0.00000000 0.00000000 1.00000000 0.00000000 0.00000000

8.815000E-01 0.00000000 0.00000000 0.00000000 0.00000000 0.00000000 1.00000000 0.00000000

3.041000E-01 0.00000000 0.00000000 0.00000000 0.00000000 0.00000000 0.00000000 1.00000000

$ p functions

6 4 0

2.846860E+02 9.4644E-04 0.00000000 0.00000000 0.00000000

4.388000E+01 1.5278E-02 0.00000000 0.00000000 0.00000000

9.926000E+00 9.8425E-02 0.00000000 0.00000000 0.00000000

2.930000E+00 0.00000000 1.00000000 0.00000000 0.00000000

9.132000E-01 0.00000000 0.00000000 1.00000000 0.00000000

2.672000E-01 0.00000000 0.00000000 0.00000000 1.00000000

$ d functions

3 2 0

4.106940E+00 1.2685E-02 0.00000000

1.193130E+00 1.1768E-01 0.00000000

1.988930E-01 0.00000000 1.00000000

$ f functions

1 1 0

1.891980E+00 1.00000000

The pecJ-2-new(gen) for C

$ C

a 6

$ s functions

11 7 0

1.620383E+05 2.0585E-05 3.7351E-05 0.00000000 0.00000000 0.00000000
0.00000000 0.00000000

8.803354E+03 4.7759E-04 8.3949E-04 0.00000000 0.00000000 0.00000000
0.00000000 0.00000000

1.333357E+03 3.7712E-03 6.8374E-03 0.00000000 0.00000000 0.00000000
0.00000000 0.00000000

3.008000E+02 1.9410E-02 3.5273E-02 0.00000000 0.00000000 0.00000000
0.00000000 0.00000000

8.511667E+01 7.5608E-02 1.3739E-01 0.00000000 0.00000000 0.00000000
0.00000000 0.00000000

2.752030E+01 2.1712E-01 4.2691E-01 0.00000000 0.00000000 0.00000000
0.00000000 0.00000000

9.731687E+00 0.00000000 0.00000000 1.00000000 0.00000000 0.00000000
0.00000000 0.00000000

3.570774E+00 0.00000000 0.00000000 0.00000000 1.00000000 0.00000000
0.00000000 0.00000000

8.102360E-01 0.00000000 0.00000000 0.00000000 0.00000000 1.00000000
0.00000000 0.00000000

3.593953E-01 0.00000000 0.00000000 0.00000000 0.00000000 0.00000000
1.00000000 0.00000000

1.356139E-01 0.00000000 0.00000000 0.00000000 0.00000000 0.00000000
0.00000000 1.00000000

$ p functions

6 4 0

9.495948E+01 8.0467E-03 0.00000000 0.00000000 0.00000000

1.778687E+01 8.8351E-02 0.00000000 0.00000000 0.00000000

4.534333E+00 4.9395E-01 0.00000000 0.00000000 0.00000000

1.336460E+00 0.00000000 1.00000000 0.00000000 0.00000000

4.353544E-01 0.00000000 0.00000000 1.00000000 0.00000000

1.348775E-01 0.00000000 0.00000000 0.00000000 1.00000000

$ d functions

3 2 0

6.136738E+00 4.7462E-02 0.00000000

1.157001E+00 2.6265E-01 0.00000000

3.542131E-01 0.00000000 1.00000000

$ f functions

1 1 0

7.838496E-01 1.00000000

The pecJ-2-new(gen) for N

$ N

a 7

$ s functions

11 7 0

2.079517E+05 1.9623E-05 4.2375E-06 0.00000000 0.00000000 0.00000000 0.00000000 0.00000000

1.292159E+04 4.3632E-04 8.8564E-05 0.00000000 0.00000000 0.00000000 0.00000000 0.00000000

1.817985E+03 3.9310E-03 7.6001E-04 0.00000000 0.00000000 0.00000000 0.00000000 0.00000000

4.082074E+02 1.9464E-02 3.7771E-03 0.00000000 0.00000000 0.00000000 0.00000000 0.00000000

1.167577E+02 7.5115E-02 1.4619E-02 0.00000000 0.00000000 0.00000000 0.00000000 0.00000000

3.805405E+01 2.1225E-01 4.1943E-02 0.00000000 0.00000000 0.00000000 0.00000000 0.00000000

1.337707E+01 0.00000000 0.00000000 1.00000000 0.00000000 0.00000000 0.00000000 0.00000000

4.904271E+00 0.00000000 0.00000000 0.00000000 1.00000000 0.00000000 0.00000000 0.00000000

1.419406E+00 0.00000000 0.00000000 0.00000000 0.00000000 1.00000000 0.00000000 0.00000000

5.160150E-01 0.00000000 0.00000000 0.00000000 0.00000000 0.00000000 1.00000000 0.00000000

1.537757E-01 0.00000000 0.00000000 0.00000000 0.00000000 0.00000000 0.00000000 1.00000000

$ p functions

6 4 0

5.465743E+01 3.1471E-03 0.00000000 0.00000000 0.00000000

1.370955E+01 1.9627E-02 0.00000000 0.00000000 0.00000000

4.095889E+00 7.9341E-02 0.00000000 0.00000000 0.00000000

1.373762E+00 0.00000000 1.00000000 0.00000000 0.00000000

4.870015E-01 0.00000000 0.00000000 1.00000000 0.00000000

1.595081E-01 0.00000000 0.00000000 0.00000000 1.00000000

$ d functions

3 2 0

4.573625E+00 4.9514E-03 0.00000000

1.031398E+00 6.7184E-02 0.00000000

2.690828E-01 0.00000000 1.00000000

$ f functions

1 1 0

1.051532E+00 1.00000000

The pecJ-2-new(gen) for F

$ F

a 9

$ s functions

11 7 0

3.224350E+05 2.2572E-05 5.2897E-05 0.00000000 0.00000000 0.00000000 0.00000000 0.00000000

1.950000E+04 4.9690E-04 1.0867E-03 0.00000000 0.00000000 0.00000000 0.00000000 0.00000000

2.923000E+03 4.0277E-03 8.5501E-03 0.00000000 0.00000000 0.00000000 0.00000000 0.00000000

6.645000E+02 2.0674E-02 4.3732E-02 0.00000000 0.00000000 0.00000000 0.00000000 0.00000000

1.875000E+02 7.9953E-02 1.7436E-01 0.00000000 0.00000000 0.00000000 0.00000000 0.00000000

6.062000E+01 2.2724E-01 5.2405E-01 0.00000000 0.00000000 0.00000000 0.00000000 0.00000000

2.142000E+01 0.00000000 0.00000000 1.00000000 0.00000000 0.00000000 0.00000000 0.00000000

7.950000E+00 0.00000000 0.00000000 0.00000000 1.00000000 0.00000000 0.00000000 0.00000000

2.257000E+00 0.00000000 0.00000000 0.00000000 0.00000000 1.00000000 0.00000000 0.00000000

8.815000E-01 0.00000000 0.00000000 0.00000000 0.00000000 0.00000000 1.00000000 0.00000000

3.041000E-01 0.00000000 0.00000000 0.00000000 0.00000000 0.00000000 0.00000000 1.00000000

$ p functions

6 4 0

2.846860E+02 9.3100E-04 0.00000000 0.00000000 0.00000000

4.388000E+01 1.5563E-02 0.00000000 0.00000000 0.00000000

9.926000E+00 1.0156E-01 0.00000000 0.00000000 0.00000000

2.930000E+00 0.00000000 1.00000000 0.00000000 0.00000000

9.132000E-01 0.00000000 0.00000000 1.00000000 0.00000000

2.672000E-01 0.00000000 0.00000000 0.00000000 1.00000000

$ d functions

3 2 0

4.106940E+00 1.3143E-02 0.00000000

1.193130E+00 1.0041E-01 0.00000000

1.988930E-01 0.00000000 1.00000000

$ f functions

1 1 0

1.891980E+00 1.00000000

Table S1. Cartesian coordinates (given in Å) for equilibrium geometries of molecules in **set 1**, calculated in the gas phase at the CCSD/pecG-2 level of theory.

Molecule name	Formula	#	Cartesian coordinates (x, y, z), in Å
propa-1,2-diene	$H_2C{=}C{=}CH_2$	**1**	C 0.000000 0.000000 -1.300423<br>C 0.000000 0.000000 0.000000

			C 0.000000 0.000000 1.300423<br>H 0.000000 -0.922935 -1.855447<br>H -0.000000 0.922935 -1.855447<br>H -0.922935 0.000000 1.855447<br>H 0.922935 0.000000 1.855447
perfluoroethene	$F_2C{=}CF_2$	**2**	C 0.000000 0.000000 0.656544<br>C 0.000000 0.000000 -0.656544<br>F 0.000000 -1.090667 1.378253<br>F 0.000000 -1.090667 -1.378253<br>F 0.000000 1.090667 1.378253<br>F 0.000000 1.090667 -1.378253
ethyne	$HC{\equiv}CH$	**3**	C 0.000000 0.000000 0.598101<br>C 0.000000 0.000000 -0.598101<br>H 0.000000 0.000000 1.656659<br>H 0.000000 0.000000 -1.656659
ethene	$H_2C{=}CH_2$	**4**	C 0.000000 0.000000 0.661783<br>H 0.000000 0.917844 1.225029<br>H -0.000000 -0.917844 1.225029<br>C 0.000000 0.000000 -0.661783<br>H -0.000000 -0.917844 -1.225029<br>H 0.000000 0.917844 -1.225029
ethane	$H_3C{-}CH_3$	**5**	C 0.000000 0.000000 0.760041<br>C 0.000000 0.000000 -0.760041<br>H -0.000000 1.012281 1.151707<br>H -0.876661 -0.506140 1.151707<br>H 0.876661 -0.506140 1.151707<br>H -0.000000 -1.012281 -1.151707<br>H -0.876661 0.506140 -1.151707<br>H 0.876661 0.506140 -1.151707
benzene	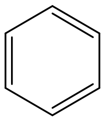	**6**	C 0.000000 1.385127 0.000000<br>C 1.199555 0.692564 0.000000<br>C -1.199555 0.692564 0.000000<br>C 1.199555 -0.692564 0.000000<br>C -1.199555 -0.692564 0.000000<br>C 0.000000 -1.385127 0.000000<br>H 0.000000 2.462523 0.000000<br>H 2.132607 1.231261 0.000000<br>H -2.132607 1.231261 0.000000<br>H 2.132607 -1.231261 0.000000<br>H -2.132607 -1.231261 0.000000<br>H 0.000000 -2.462523 0.000000
perfluoromethane	CF_4	**7**	C -0.000002 -0.000002 0.041745<br>F -0.000955 0.000165 1.353146<br>F 1.236719 -0.000108 -0.394476<br>F -0.617925 -1.070780 -0.395706<br>F -0.617837 1.070723 -0.395967
fluoroethene	$H_2C{=}CHF$	**8**	C -1.179485 0.148798 -0.000000<br>C -0.000000 -0.431741 0.000000<br>F 1.137666 0.270277 -0.000000<br>H -2.063126 -0.460160 0.000000<br>H -1.276185 1.218349 -0.000000<br>H 0.177220 -1.493022 0.000000

difluoromethane	CH_2F_2	**9**	C -0.000000 0.000000 0.501151<br>F 0.000000 -1.092441 -0.289025<br>F 0.000000 1.092441 -0.289025<br>H 0.903877 0.000000 1.097771<br>H -0.903877 -0.000000 1.097771
methanimine	$H_2C{=}NH$	**10**	C -0.056062 -0.582947 0.000000<br>N -0.056062 0.680399 0.000000<br>H -1.003046 -1.103739 0.000000<br>H 0.839375 -1.196244 0.000000<br>H 0.892483 1.034868 0.000000
prop-1-yne	$HC{\equiv}C{-}CH_3$	**11**	C 0.000000 0.000000 1.416878<br>C 0.000000 0.000000 0.219120<br>C 0.000000 0.000000 -1.238315<br>H 0.000000 0.000000 2.474506<br>H 0.000000 1.015424 -1.620199<br>H 0.879383 -0.507712 -1.620199<br>H -0.879383 -0.507712 -1.620199
acetaldehyde	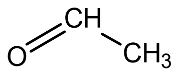	**12**	C 0.000000 0.459915 0.000000<br>C -0.927794 -0.713120 0.000000<br>O 1.196360 0.379972 0.000000<br>H -0.489716 1.445472 0.000000<br>H -0.370601 -1.640849 0.000000<br>H -1.571901 -0.662583 0.874050<br>H -1.571901 -0.662583 -0.874050
acetonitrile	$N{\equiv}C{-}CH_3$	**13**	C 0.000000 0.000000 -1.175537<br>C 0.000000 0.000000 0.280590<br>N 0.000000 0.000000 1.429050<br>H 0.000000 1.018400 -1.544557<br>H 0.881960 -0.509200 -1.544557<br>H -0.881960 -0.509200 -1.544557
fluoromethane	$H_3C{-}F$	**14**	C 0.000000 0.000000 -0.629873<br>F 0.000000 0.000000 0.746820<br>H 0.000000 1.025424 -0.980713<br>H 0.888043 -0.512712 -0.980713<br>H -0.888043 -0.512712 -0.980713
methanamine	$H_3C{-}NH_2$	**15**	C -0.049945 -0.702319 0.000000<br>N -0.049945 0.754013 0.000000<br>H 0.439869 1.104104 0.804592<br>H 0.439869 1.104104 -0.804592<br>H -0.585425 -1.057065 -0.873643<br>H 0.940396 -1.158249 0.000000<br>H -0.585425 -1.057065 0.873643
methane	CH_4	**16**	C 0.000000 0.000000 0.000000<br>H 0.625347 0.625347 0.625347<br>H -0.625347 -0.625347 0.625347<br>H -0.625347 0.625347 -0.625347<br>H 0.625347 -0.625347 -0.625347
fluoroform	CHF_3	**17**	C 0.000000 0.000000 0.336530<br>H 0.000000 0.000000 1.417189<br>F -0.000000 1.242176 -0.127273<br>F -1.075756 -0.621088 -0.127273

			F 1.075756 -0.621088 -0.127273
cyclopropane	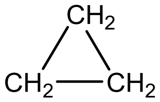	**18**	C 0.000000 0.864052 0.000000<br>C 0.748291 -0.432026 0.000000<br>C -0.748291 -0.432026 0.000000<br>H 0.000000 1.443165 -0.905357<br>H 0.000000 1.443165 0.905357<br>H 1.249818 -0.721583 0.905357<br>H 1.249818 -0.721583 -0.905357<br>H -1.249818 -0.721583 -0.905357<br>H -1.249818 -0.721583 0.905357
1,2-difluoroethyne	F−C≡C−F	**19**	C 0.000000 0.000000 0.589665<br>C 0.000000 0.000000 -0.589665<br>F 0.000000 0.000000 1.870179<br>F 0.000000 0.000000 -1.870179
fluoroformonitrile	F−C≡N	**20**	C 0.000000 0.000000 -0.150358<br>F 0.000000 0.000000 1.110457<br>N 0.000000 0.000000 -1.298853
1,1-difluoroethene	$H_2C{=}CF_2$	**21**	C 0.000000 0.000000 1.374985<br>C 0.000000 0.000000 0.064575<br>F 0.000000 -1.071729 -0.691406<br>F 0.000000 1.071729 -0.691406<br>H 0.000000 -0.930524 1.903971<br>H 0.000000 0.930524 1.903971
formaldehyde oxime (*Z*)	$H_2C{=}NOH$ (*Z*)	**22**	C -1.108056 0.051744 0.000000<br>N 0.000000 -0.554688 0.000000<br>O 1.123141 0.229526 0.000000<br>H -1.997491 -0.549655 0.000000<br>H -1.192331 1.132841 0.000000<br>H 0.853026 1.152958 0.000000
formaldehyde oxime (*E*)	$H_2C{=}NOH$ (*E*)	**23**	C 1.129613 -0.030560 0.000000<br>N 0.000000 0.533596 0.000000<br>O -1.024575 -0.399663 0.000000<br>H 1.990821 0.610470 0.000000<br>H 1.238396 -1.104440 0.000000<br>H -1.810300 0.139459 0.000000
fluoroethyne	H−C≡C−F	**24**	C 0.000000 0.000000 -1.284000<br>C 0.000000 0.000000 -0.094819<br>F 0.000000 0.000000 1.179165<br>H 0.000000 0.000000 -2.339575
hydrogen cyanide	H−C≡N	**25**	C 0.000000 0.000000 -0.497569<br>N 0.000000 0.000000 0.649148<br>H 0.000000 0.000000 -1.558622
1,1,2-trifluoroethene	$F_2C{=}CHF$	**26**	C -0.697161 0.685937 0.000000<br>C 0.000000 -0.428511 0.000000<br>F -0.553965 -1.616153 0.000000<br>F -0.088086 1.869960 0.000000<br>F 1.303187 -0.503165 0.000000<br>H -1.767253 0.699675 0.000000
(*Z*)-1,2-difluoroethene	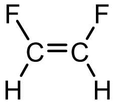	**27**	C 0.000000 0.658113 0.572580<br>C 0.000000 -0.658113 0.572580<br>F 0.000000 1.376463 -0.546035

			F 0.000000 -1.376463 -0.546035<br>H 0.000000 1.233231 1.478837<br>H 0.000000 -1.233231 1.478837
(*E*)-1,2-difluoroethene	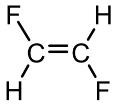	**28**	C 0.330081 0.568915 0.000000<br>C -0.330081 -0.568915 0.000000<br>F -0.330081 1.728889 0.000000<br>F 0.330081 -1.728889 0.000000<br>H 1.399763 0.659121 0.000000<br>H -1.399763 -0.659121 0.000000
hydrogen fluoride	HF	**29**	F 0.000000 0.000000 0.091281<br>H 0.000000 0.000000 -0.821527
diazene (*E*)	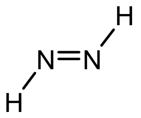	**30**	N 0.000000 0.617820 0.000000<br>N 0.000000 -0.617820 0.000000<br>H 0.978113 0.912762 0.000000<br>H -0.978113 -0.912762 0.000000
diazene (*Z*)	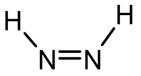	**31**	N 0.000000 -0.617776 -0.118814<br>N 0.000000 0.617776 -0.118814<br>H 0.000000 -1.005077 0.831699<br>H 0.000000 1.005077 0.831699
hydrazine	NH_2—NH_2	**32**	N 0.000000 -0.731521 0.000000<br>N 0.000000 0.731521 0.000000<br>H -0.575404 -0.984536 0.789022<br>H -0.575404 -0.984536 -0.789022<br>H 0.575404 0.984536 0.789022<br>H 0.575404 0.984536 -0.789022
molecular nitrogen	N_2	**33**	N 0.000000 0.000000 0.545999<br>N 0.000000 0.000000 -0.545999
ammonia	NH_3	**34**	N 0.000000 0.000000 0.113268<br>H 0.000000 0.932620 -0.264293<br>H -0.807673 -0.466310 -0.264293<br>H 0.807673 -0.466310 -0.264293
1*H*-pyrazole	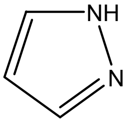	**35**	C -0.660673 -0.991538 0.000000<br>C 0.740838 -0.882760 0.000000<br>C -1.100154 0.304017 0.000000<br>N 1.135540 0.375864 0.000000<br>N 0.000000 1.077644 0.000000<br>H -1.257310 -1.880482 0.000000<br>H 1.470076 -1.668762 0.000000<br>H -2.085838 0.723502 0.000000<br>H 0.044230 2.072874 0.000000
1*H*-imidazole	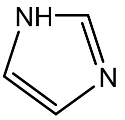	**36**	C 0.632207 0.975656 0.000000<br>C 1.110762 -0.294919 0.000000<br>C -1.079418 -0.280951 0.000000<br>N -0.740582 0.975728 0.000000<br>N 0.000000 -1.096287 0.000000<br>H 1.191108 1.889732 0.000000<br>H 2.104572 -0.691648 0.000000<br>H -2.082022 -0.660114 0.000000<br>H -0.010887 -2.092765 0.000000

pyridine	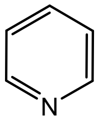	**37**	C 0.000000 0.000000 1.372461<br>C 0.000000 -1.188632 0.667521<br>C 0.000000 1.188632 0.667521<br>C 0.000000 -1.132248 -0.715288<br>C 0.000000 1.132248 -0.715288<br>N 0.000000 0.000000 -1.408981<br>H 0.000000 0.000000 2.449566<br>H 0.000000 -2.139614 1.171275<br>H 0.000000 2.139614 1.171275<br>H 0.000000 -2.041392 -1.295406<br>H 0.000000 2.041392 -1.295406
1*H*-pyrrole	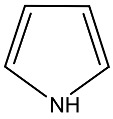	**38**	N 0.000000 0.000000 1.113072<br>C 0.000000 0.709577 -0.974560<br>C 0.000000 -0.709577 -0.974560<br>C 0.000000 1.115090 0.328512<br>C 0.000000 -1.115090 0.328512<br>H 0.000000 1.353674 -1.831733<br>H 0.000000 -1.353674 -1.831733<br>H 0.000000 2.096429 0.758028<br>H 0.000000 -2.096429 0.758028<br>H 0.000000 0.000000 2.108486
prop-1-ene	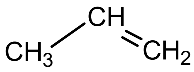	**39**	C 1.282663 0.145278 0.000000<br>C 0.000000 0.474110 0.000000<br>C -1.131065 -0.500216 0.000000<br>H 2.056330 0.893712 0.000000<br>H 1.596096 -0.885790 0.000000<br>H -0.270003 1.520715 0.000000<br>H -1.762064 -0.360456 0.874238<br>H -1.762064 -0.360456 -0.874238<br>H -0.767879 -1.522764 0.000000
furan	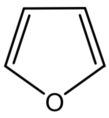	**40**	C 0.000000 -0.715469 -0.950987<br>C 0.000000 0.715469 -0.950987<br>C 0.000000 -1.083784 0.344086<br>C 0.000000 1.083784 0.344086<br>O 0.000000 0.000000 1.151802<br>H 0.000000 -1.368707 -1.800012<br>H 0.000000 1.368707 -1.800012<br>H 0.000000 -2.035410 0.834213<br>H 0.000000 2.035410 0.834213
1-fluorobenzene	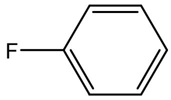	**41**	F 0.000000 0.000000 -2.261756<br>C 0.000000 0.000000 -0.922616<br>C 0.000000 1.207303 -0.259170<br>C 0.000000 -1.207303 -0.259170<br>C 0.000000 -1.197479 1.125274<br>C 0.000000 1.197479 1.125274<br>C 0.000000 0.000000 1.820867<br>H 0.000000 2.123485 -0.822445<br>H 0.000000 -2.123485 -0.822445<br>H 0.000000 -2.132086 1.660347<br>H 0.000000 2.132086 1.660347<br>H 0.000000 0.000000 2.897243

Table S2. Z-matrices (distances in Å, angles in arc degrees) for equilibrium geometries of molecules in **set 2**, calculated in the gas phase at the CCSD(T)/pecG-2 level of theory.

Molecule name	Formula	#	Z-matrix
methane	CH_4	**16**	C<br>H 1 R1<br>H 1 R1 2 TDA<br>H 1 R1 2 TDA 3 D120<br>H 1 R1 2 TDA 4 D120<br><br>R1 = 1.084456335441068<br>TDA = 109.471220634490692<br>D120 = 120.000000000000014
ethyne	C_2H_2	**3**	C<br>C 1 cc2<br>X 1 xc3 2 xcc3<br>H 1 hc 3 hcx4 2 dih4<br>X 2 xc5 1 xcc5 3 dih5<br>H 2 hc 5 hcx6 1 dih6<br><br>cc2 = 1.202456436003752<br>xc3 = 1.000000000000000<br>xcc3 = 90.000000000000000<br>hc = 1.060283089535931<br>hcx4 = 90.000000000000000<br>dih4 = 180.000000000000000<br>xc5 = 1.000000000000000<br>xcc5 = 90.000000000000000<br>dih5 = 0.000000000000000<br>hcx6 = 90.000000000000000<br>dih6 = 180.000000000000000
ethene	C_2H_4	**4**	C<br>C 1 cc2<br>H 1 hc1 2 hcc3<br>H 1 hc2 2 hcc4 3 dih4<br>H 2 hc3 1 hcc5 3 dih5<br>H 2 hc4 1 hcc6 4 dih6<br><br>cc2 = 1.329416768328072<br>hc1 = 1.078534910698578<br>hcc3 = 121.484290755569717<br>hc2 = 1.078534910698578<br>hcc4 = 121.484290755569717<br>dih4 = 180.000000000000000<br>hc3 = 1.078534910698578<br>hcc5 = 121.484290755569717<br>dih5 = 180.000000000000000<br>hc4 = 1.078534910698578<br>hcc6 = 121.484290755569717

			dih6 = 180.000000000000000
ethane	C_2H_6	**5**	C<br>C 1 RCC<br>H 1 RCH 2 ACCH<br>H 1 RCH 2 ACCH 3 dih1<br>H 1 RCH 2 ACCH 3 dih2<br>H 2 RCH 1 ACCH 3 dih3<br>H 2 RCH 1 ACCH 6 dih1<br>H 2 RCH 1 ACCH 6 dih2<br><br>RCC = 1.522859795544686<br>RCH = 1.086947636619504<br>ACCH = 111.130123308838236<br>dih1 = 120.000000000000199<br>dih2 = -120.000000000000199<br>dih3 = 180.000000000000000
fluoromethane	CH_3F	**14**	C<br>F 1 R1<br>H 1 R2 2 TDA<br>H 1 R2 2 TDA 3 D120<br>H 1 R2 2 TDA 4 D120<br><br>R1 = 1.382246858548905<br>R2 = 1.085348636855558<br>TDA = 108.833524883838351<br>D120 = 120.000000000000071
difluoromethane	CH_2F_2	**9**	C<br>H 1 RCH<br>H 1 RCH 2 HCH<br>F 1 RCL 2 CCL 3 Dih1<br>F 1 RCL 3 CCL 2 Dih2<br><br>RCH = 1.084740022906903<br>HCH = 113.336003478303297<br>RCL = 1.353563637907476<br>CCL = 108.784850227424641<br>Dih1 = 121.142810901920157<br>Dih2 = 121.142810901920186
acetonitrile	CH_3CN	**13**	C<br>C 1 cc2<br>X 2 xc3 1 xcc3<br>N 2 nc4 3 ncx4 1 dih4<br>H 1 hc5 2 hcc5 3 dih5<br>H 1 hc5 2 hcc5 3 dih6<br>H 1 hc5 2 hcc5 3 dih7<br><br>cc2 = 1.458019766881534<br>xc3 = 1.000000000000000<br>xcc3 = 90.000000000000000<br>nc4 = 1.155889158820919<br>ncx4 = 90.000000000000000

			dih4 = 180.000000000000000<br>hc5 = 1.084902112750216<br>hcc5 = 109.912174661669553<br>dih5 = 0.000000000000000<br>dih6 = -119.999999999999986<br>dih7 = 119.999999999999986
methanamine	CH_3NH_2	**15**	C<br>N 1 nc2<br>H 1 hc3 2 hcn3<br>H 1 hc4 2 hcn4 3 dih4<br>H 1 hc3 2 hcn3 3 dih5<br>H 2 hn6 1 hnc6 3 dih6<br>H 2 hn6 1 hnc6 3 dih7<br><br>nc2 = 1.460967830294795<br>hc3 = 1.085892872089478<br>hcn3 = 109.007144147067820<br>hc4 = 1.091888215133912<br>hcn4 = 114.766653264617119<br>dih4 = 121.526090811322462<br>dih5 = -116.947818395873767<br>hn6 = 1.007450240163833<br>hnc6 = 110.098639514378547<br>dih6 = -179.871909347760777<br>dih7 = -63.180273733136552

Table S3. Reference symmetry independent values of SSCC (in Hz) in molecules of set 1 calculated at the SOPPA(CCSD) level with the ccJ-pV5Z basis set.

#	Molecule	Type of SSCC [1]	SSCC value
1	$H_2C{=}C{=}CH_2$<br>propa-1,2-diene (**1**)	$^1J(^{13}C,^{13}C)$	104.2978
2		$^2J(^{13}C,^{13}C)$	8.4616
3		$^2J(^{13}C,^1H)$	-5.4190
4		$^1J(^{13}C,^1H)$	166.9576
5		$^3J(^{13}C,^1H)$	7.7481
6		$^2J(^1H,^1H)$	-13.8843
7		$^4J(^1H,^1H)$	-8.4965
8	$F_2C{=}CF_2$<br>Perfluoroethene (**2**)	$^1J(^{13}C,^{13}C)$	199.6821
9		$^1J(^{13}C,^{19}F)$	-265.1876
10		$^2J(^{13}C,^{19}F)$	50.3079
11		$^2J(^{19}F,^{19}F)$	115.7032
12		$^3J_{cis}(^{19}F,^{19}F)$	77.0144
13		$^3J_{trans}(^{19}F,^{19}F)$	-114.5148
14	$HC{\equiv}CH$<br>ethyne (**3**)	$^1J(^{13}C,^{13}C)$	190.3749
15		$^1J(^{13}C,^1H)$	252.0253
16		$^2J(^{13}C,^1H)$	52.0810
17		$^3J(^1H,^1H)$	10.8211

18	$H_2C{=}CH_2$ ethene (**4**)	$^{1}J(^{13}C,^{13}C)$	71.2116
19		$^{1}J(^{13}C,^{1}H)$	154.4322
20		$^{2}J(^{13}C,^{1}H)$	-2.8877
21		$^{3}J_{cis}(^{1}H,^{1}H)$	12.2720
22		$^{2}J(^{1}H,^{1}H)$	0.4726
23		$^{3}J_{trans}(^{1}H,^{1}H)$	18.4140
24	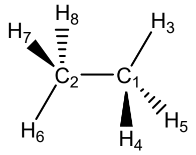 ethane (**5**)	$^{1}J(^{13}C,^{13}C)$	35.0676
25		$^{1}J(^{13}C,^{1}H)$	122.0646
26		$^{2}J(^{13}C,^{1}H)$	-4.8716
27		$^{3}J_{trans}(^{1}H_3,^{1}H_6)$	15.8931
28		$^{2}J(^{1}H,^{1}H)$	-13.8980
29		$^{3}J_{gauche}(^{1}H_4,^{1}H_6)$	3.8126
30	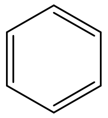 benzene (**6**)	$^{3}J(^{13}C,^{13}C)$	11.0857
31		$^{1}J(^{13}C,^{13}C)$	59.3638
32		$^{2}J(^{13}C,^{13}C)$	-3.3357
33		$^{1}J(^{13}C,^{1}H)$	155.8996
34		$^{4}J(^{13}C,^{1}H)$	-1.6513
35		$^{2}J(^{13}C,^{1}H)$	0.1232
36		$^{3}J(^{13}C,^{1}H)$	7.7453
37		$^{3}J(^{1}H,^{1}H)$	8.1029
38		$^{4}J(^{1}H,^{1}H)$	0.8913
39		$^{5}J(^{1}H,^{1}H)$	1.1347
40	CF_4 perfluoromethane (**7**)	$^{1}J(^{19}F,^{13}C)$	-253.8939
41		$^{2}J(^{19}F,^{19}F)$	35.7720
42	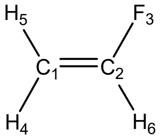 fluoroethene (**8**)	$^{1}J(^{13}C,^{13}C)$	88.1847
43		$^{2}J(^{19}F,^{13}C)$	11.3271
44		$^{1}J(^{19}F,^{13}C)$	-258.5006
45		$^{1}J(^{13}C_1,^{1}H_4)$	159.6631
46		$^{2}J(^{13}C_2,^{1}H_4)$	7.3474
47		$^{3}J_{trans}(^{19}F_3,^{1}H_4)$	42.0785
48		$^{1}J(^{13}C_1,^{1}H_5)$	157.3382
49		$^{2}J(^{13}C_2,^{1}H_5)$	-9.7978
50		$^{3}J_{cis}(^{19}F_3,^{1}H_5)$	14.3913
51		$^{2}J(^{1}H,^{1}H)$	-4.5296
52		$^{2}J(^{13}C_1,^{1}H_6)$	13.3026
53		$^{1}J(^{13}C_2,^{1}H_6)$	194.0521
54		$^{2}J(^{19}F,^{1}H)$	83.3604
55		$^{3}J_{cis}(^{1}H_6,^{1}H_4)$	5.9487
56		$^{3}J_{trans}(^{1}H_6,^{1}H_5)$	12.7470
57	CH_2F_2 difluoromethane (**9**)	$^{1}J(^{19}F,^{13}C)$	-225.1834
58		$^{2}J(^{19}F,^{19}F)$	321.5740
59		$^{1}J(^{13}C,^{1}H)$	175.1877
60		$^{2}J(^{19}F,^{1}H)$	50.1121
61		$^{2}J(^{1}H,^{1}H)$	1.0184
62		$^{1}J(^{15}N,^{13}C)$	-4.0256

63		$^{1}J(^{13}C_1,^{1}H_3)$	171.6253
64		$^{2}J(^{15}N_2,^{1}H_3)$	-9.4091
65		$^{1}J(^{13}C_1,^{1}H_4)$	156.4978
66	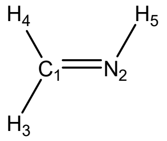	$^{2}J(^{15}N_2,^{1}H_4)$	4.1007
67		$^{2}J(^{1}H,^{1}H)$	16.8349
68		$^{2}J(^{13}C,^{1}H)$	-13.2333
69	methanimine (**10**)	$^{1}J(^{15}N,^{1}H)$	-51.3919
70		$^{3}J_{trans}(^{1}H_5,^{1}H_3)$	24.5334
71		$^{3}J_{cis}(^{1}H_5,^{1}H_4)$	18.2590
72		$^{1}J(^{13}C_1,^{13}C_2)$	191.4558
73		$^{2}J(^{13}C,^{13}C)$	13.0135
74		$^{1}J(^{13}C_2,^{13}C_3)$	71.0499
75		$^{1}J(^{13}C_1,^{1}H_4)$	250.8325
76	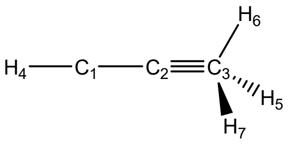	$^{2}J(^{13}C_2,^{1}H_4)$	52.0378
77		$^{3}J(^{13}C_3,^{1}H_4)$	4.1120
78		$^{3}J(^{13}C_1,^{1}H_6)$	3.7491
79	prop-1-yne (**11**)	$^{2}J(^{13}C_2,^{1}H_6)$	-11.5442
80		$^{1}J(^{13}C_3,^{1}H_6)$	128.3717
81		$^{4}J(^{1}H,^{1}H)$	-3.5447
82		$^{2}J(^{1}H,^{1}H)$	-17.2195
83		$^{1}J(^{13}C,^{13}C)$	41.9032
84		$^{1}J(^{13}C_1,^{1}H_4)$	166.4939
85		$^{2}J(^{13}C_2,^{1}H_4)$	26.4436
86		$^{2}J(^{13}C_1,^{1}H_5)$	-8.3028
87	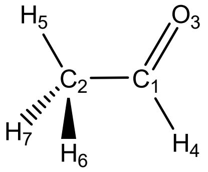	$^{1}J(^{13}C_2,^{1}H_5)$	131.4773
88		$^{3}J_{trans}(^{1}H_5,^{1}H_4)$	8.2476
89		$^{2}J(^{13}C_1,^{1}H_6)$	-6.2554
90	acetaldehyde (**12**)	$^{1}J(^{13}C_2,^{1}H_6)$	120.3929
91		$^{3}J_{gauche}(^{1}H_6,^{1}H_4)$	0.3703
92		$^{2}J(^{1}H_6,^{1}H_5)$	-13.2517
93		$^{2}J(^{1}H_6,^{1}H_7)$	-20.0105
94		$^{1}J(^{13}C,^{13}C)$	62.7865
95		$^{2}J(^{15}N,^{13}C)$	2.7988
96	$N{\equiv}C{-}CH_3$	$^{1}J(^{15}N,^{13}C)$	-17.6879
97	acetonitrile (**13**)	$^{1}J(^{13}C,^{1}H)$	131.6532
98		$^{2}J(^{13}C,^{1}H)$	-10.7691
99		$^{3}J(^{15}N,^{1}H)$	-1.5819
100		$^{2}J(^{1}H,^{1}H)$	-17.2448
101		$^{1}J(^{19}F,^{13}C)$	-160.2124
102	$H_3C{-}F$	$^{1}J(^{13}C,^{1}H)$	143.8290
103	fluoromethane (**14**)	$^{2}J(^{19}F,^{1}H)$	48.3234
104		$^{2}J(^{1}H,^{1}H)$	-10.8042
105		$^{1}J(^{15}N,^{13}C)$	-5.8387
106		$^{2}J(^{13}C,^{1}H)$	-3.5005
107		$^{1}J(^{15}N,^{1}H)$	-65.8590

108	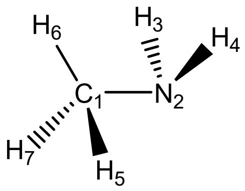 methanamine (**15**)	$^{2}J(^{1}H_{3},^{1}H_{4})$	-10.4962
109		$^{1}J(^{13}C_{1},^{1}H_{5})$	129.7384
110		$^{2}J(^{15}N_{2},^{1}H_{5})$	-1.4937
111		$^{3}J(^{1}H_{5},^{1}H_{4})$	2.7766
112		$^{3}J(^{1}H_{5},^{1}H_{3})$	15.7201
113		$^{2}J(^{1}H_{7},^{1}H_{5})$	-15.5989
114		$^{1}J(^{13}C_{1},^{1}H_{6})$	127.1083
115		$^{2}J(^{15}N_{2},^{1}H_{6})$	0.9909
116		$^{3}J(^{1}H_{6},^{1}H_{4})$	2.2091
117		$^{2}J(^{1}H_{6},^{1}H_{5})$	-11.4293
118	CH_4 methane (**16**)	$^{1}J(^{13}C,^{1}H)$	122.2492
119		$^{2}J(^{1}H,^{1}H)$	-13.6506
120	CHF_3 fluoroform (**17**)	$^{1}J(^{13}C,^{1}H)$	225.4331
121		$^{1}J(^{19}F,^{13}C)$	-260.2719
122		$^{2}J(^{19}F,^{1}H)$	74.8329
123		$^{2}J(^{19}F,^{19}F)$	129.3441
124	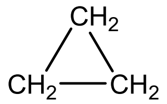 cyclopropane (**18**)	$^{1}J(^{13}C,^{13}C)$	13.3984
125		$^{1}J(^{13}C,^{1}H)$	157.0148
126		$^{2}J(^{13}C,^{1}H)$	-2.8971
127		$^{2}J(^{1}H,^{1}H)$	-5.8414
128		$^{3}J_{cis}(^{1}H,^{1}H)$	9.1431
129		$^{3}J_{trans}(^{1}H,^{1}H)$	5.1098
130	F−C≡C−F 1,2-difluoroethyne (**19**)	$^{1}J(^{13}C,^{13}C)$	413.9765
131		$^{1}J(^{19}F,^{13}C)$	-276.4495
132		$^{2}J(^{19}F,^{13}C)$	40.1731
133		$^{3}J(^{19}F,^{19}F)$	-7.2499
134	F−C≡N fluoroformonitrile (**20**)	$^{1}J(^{19}F,^{13}C)$	-405.0512
135		$^{1}J(^{15}N,^{13}C)$	-3.5361
136		$^{2}J(^{19}F,^{15}N)$	52.3182
137	$H_2C{=}CF_2$ 1,1-difluoroethene (**21**)	$^{1}J(^{13}C,^{13}C)$	118.2347
138		$^{2}J(^{19}F,^{13}C)$	27.8836
139		$^{1}J(^{19}F,^{13}C)$	-284.9060
140		$^{2}J(^{19}F,^{19}F)$	24.0860
141		$^{1}J(^{13}C,^{1}H)$	164.5183
142		$^{2}J(^{13}C,^{1}H)$	-1.6924
143		$^{3}J_{cis}(^{19}F,^{1}H)$	-1.0442
144		$^{3}J_{trans}(^{19}F,^{1}H)$	28.3367
145		$^{2}J(^{1}H,^{1}H)$	-6.2723
146	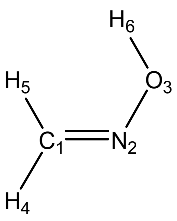 formaldehyde oxime (*Z*) (**22**)	$^{1}J(^{15}N,^{13}C)$	-2.8097
147		$^{1}J(^{13}C_{1},^{1}H_{4})$	184.3752
148		$^{2}J(^{15}N_{2},^{1}H_{4})$	-12.4344
149		$^{1}J(^{13}C_{1},^{1}H_{5})$	153.8403
150		$^{2}J(^{15}N_{2},^{1}H_{5})$	2.9713
151		$^{2}J(^{1}H,^{1}H)$	6.4306
152		$^{3}J(^{13}C,^{1}H)$	4.1099

153		$^{2}J(^{15}N_2,^{1}H_6)$	1.4126
154		$^{4}J(^{1}H_6,^{1}H_4)$	-1.2276
155		$^{4}J(^{1}H_6,^{1}H_5)$	1.0767
156	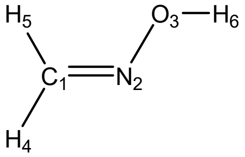 formaldehyde oxime (*E*) (**23**)	$^{1}J(^{15}N,^{13}C)$	-5.8505
157		$^{1}J(^{13}C_1,^{1}H_4)$	177.4637
158		$^{2}J(^{15}N_2,^{1}H_4)$	-13.2225
159		$^{1}J(^{13}C_1,^{1}H_5)$	163.2842
160		$^{2}J(^{15}N_2,^{1}H_5)$	2.9719
161		$^{2}J(^{1}H,^{1}H)$	8.0869
162		$^{3}J(^{13}C,^{1}H)$	10.8440
163		$^{2}J(^{15}N_2,^{1}H_6)$	-1.8958
164		$^{4}J(^{1}H_6,^{1}H_4)$	1.4257
165		$^{4}J(^{1}H_6,^{1}H_5)$	-0.3411
166	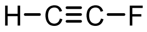 fluoroethyne (**24**)	$^{1}J(^{13}C,^{13}C)$	276.5630
167		$^{2}J(^{19}F,^{13}C)$	23.0434
168		$^{1}J(^{19}F,^{13}C)$	-291.3098
169		$^{1}J(^{13}C,^{1}H)$	280.5430
170		$^{2}J(^{13}C,^{1}H)$	66.9935
171		$^{3}J(^{19}F,^{1}H)$	11.8905
172	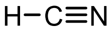 hydrogen cyanide (**25**)	$^{1}J(^{15}N,^{13}C)$	-18.5021
173		$^{1}J(^{13}C,^{1}H)$	263.6928
174		$^{2}J(^{15}N,^{1}H)$	-8.5363
175	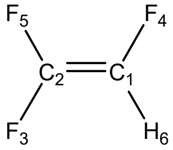 1,1,2-trifluoroethene (**26**)	$^{1}J(^{13}C,^{13}C)$	146.5692
176		$^{2}J(^{19}F_3,^{13}C_1)$	66.0569
177		$^{1}J(^{19}F_3,^{13}C_2)$	-271.0691
178		$^{1}J(^{19}F_4,^{13}C_1)$	-231.7360
179		$^{2}J(^{19}F_4,^{13}C_2)$	36.3721
180		$^{3}J_{trans}(^{19}F_3,^{19}F_4)$	-120.9432
181		$^{2}J(^{19}F_5,^{13}C_1)$	19.8532
182		$^{1}J(^{19}F_5,^{13}C_2)$	-283.8055
183		$^{2}J(^{19}F,^{19}F)$	73.6582
184		$^{3}J_{cis}(^{19}F_4,^{19}F_5)$	38.0697
185		$^{1}J(^{13}C,^{1}H)$	205.1002
186		$^{2}J(^{13}C,^{1}H)$	14.5802
187		$^{3}J_{cis}(^{19}F_3,^{1}H_6)$	-5.1069
188		$^{2}J(^{19}F,^{1}H)$	73.0866
189		$^{3}J_{trans}(^{19}F_5,^{1}H_6)$	8.5551
190	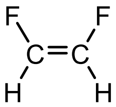 (*Z*)-1,2-difluoroethene (**27**)	$^{1}J(^{13}C,^{13}C)$	101.7526
191		$^{1}J(^{19}F,^{13}C)$	-251.9295
192		$^{2}J(^{19}F,^{13}C)$	8.7429
193		$^{3}J(^{19}F,^{19}F)$	-13.5464
194		$^{1}J(^{13}C,^{1}H)$	198.3466
195		$^{2}J(^{13}C,^{1}H)$	23.3694
196		$^{2}J(^{19}F,^{1}H)$	73.1945
197		$^{3}J(^{19}F,^{1}H)$	14.7564

198		$^{3}J(^{1}H,^{1}H)$	3.4644
199	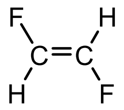 (*E*)-1,2-difluoroethene (**28**)	$^{1}J(^{13}C,^{13}C)$	116.0859
200		$^{1}J(^{19}F,^{13}C)$	-238.9994
201		$^{2}J(^{19}F,^{13}C)$	49.9793
202		$^{3}J(^{19}F,^{19}F)$	-134.0178
203		$^{2}J(^{13}C,^{1}H)$	4.6491
204		$^{1}J(^{13}C,^{1}H)$	196.4156
205		$^{3}J(^{19}F,^{1}H)$	0.9937
206		$^{2}J(^{19}F,^{1}H)$	76.9227
207		$^{3}J(^{1}H,^{1}H)$	9.9143
208	HF hydrogen fluoride (**29**)	$^{1}J(^{19}F,^{1}H)$	549.5992
209	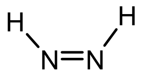 diazene (*Z*) (**31**)	$^{1}J(^{15}N,^{15}N)$	-21.0960
210		$^{1}J(^{15}N,^{1}H)$	-36.6394
211		$^{2}J(^{15}N,^{1}H)$	1.6059
212		$^{3}J(^{1}H,^{1}H)$	37.8110
213	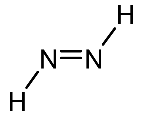 diazene (*E*) (**30**)	$^{1}J(^{15}N,^{15}N)$	-21.7396
214		$^{2}J(^{15}N,^{1}H)$	0.0452
215		$^{1}J(^{15}N,^{1}H)$	-46.6834
216		$^{3}J(^{1}H,^{1}H)$	37.4152
217	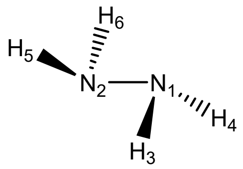 hydrazine (**32**)	$^{1}J(^{15}N,^{15}N)$	0.9880
218		$^{1}J(^{15}N,^{1}H)$	-59.8936
219		$^{2}J(^{15}N,^{1}H)$	-1.6209
220		$^{2}J(^{1}H,^{1}H)$	-15.0264
221		$^{3}J(^{1}H_5,^{1}H_3)$	1.5743
222		$^{3}J(^{1}H_4,^{1}H_5)$	13.4936
223	N_2 molecular nitrogen (**33**)	$^{1}J(^{15}N,^{15}N)$	-2.7975
224	NH_3 ammonia (**34**)	$^{1}J(^{15}N,^{1}H)$	-62.4496
225		$^{2}J(^{1}H,^{1}H)$	-10.9202
226	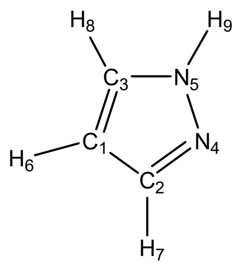 1*H*-pyrazole (**35**)	$^{1}J(^{13}C_1,^{13}C_2)$	55.0479
227		$^{1}J(^{13}C_3,^{13}C_1)$	69.7061
228		$^{2}J(^{13}C_3,^{13}C_2)$	1.8552
229		$^{2}J(^{15}N_4,^{13}C_1)$	3.0284
230		$^{1}J(^{15}N_4,^{13}C_2)$	-2.1517
231		$^{2}J(^{15}N_4,^{13}C_3)$	1.0101
232		$^{2}J(^{15}N_5,^{13}C_1)$	-5.7688
233		$^{2}J(^{15}N_5,^{13}C_2)$	-0.7830
234		$^{1}J(^{15}N_5,^{13}C_3)$	-15.4133
235		$^{1}J(^{15}N,^{15}N)$	-11.9913
236		$^{1}J(^{13}C_1,^{1}H_6)$	173.1490
237		$^{2}J(^{13}C_2,^{1}H_6)$	5.4070
238		$^{2}J(^{13}C3,^{1}H_6)$	7.9071
239		$^{3}J(^{15}N_4,^{1}H_6)$	-1.1411
240		$^{3}J(^{15}N_5,^{1}H_6)$	-5.8015
241		$^{2}J(^{13}C_1,^{1}H_7)$	10.7999

242		$^{1}J(^{13}C_{2},^{1}H_{7})$	182.5737
243		$^{3}J(^{13}C_{3},^{1}H_{7})$	4.7448
244		$^{2}J(^{15}N_{4},^{1}H_{7})$	-12.0885
245		$^{3}J(^{15}N_{5},^{1}H_{7})$	-8.8595
246		$^{3}J(^{1}H_{7},^{1}H_{6})$	1.8882
247		$^{2}J(^{13}C_{1},^{1}H_{8})$	7.0957
248		$^{3}J(^{13}C_{2},^{1}H_{8})$	7.8821
249		$^{1}J(^{13}C_{3},^{1}H_{8})$	181.2382
250		$^{3}J(^{15}N_{4},^{1}H_{8})$	0.2483
251		$^{2}J(^{15}N_{5},^{1}H_{8})$	-4.6284
252		$^{3}J(^{1}H_{8},^{1}H_{6})$	3.0612
253		$^{4}J(^{1}H_{8},^{1}H_{7})$	0.2365
254		$^{3}J(^{13}C_{1},^{1}H_{9})$	5.5310
255		$^{3}J(^{13}C_{2},^{1}H_{9})$	10.2935
256		$^{2}J(^{13}C_{3},^{1}H_{9})$	9.2868
257		$^{2}J(^{15}N_{4},^{1}H_{9})$	-8.6637
258		$^{1}J(^{15}N,^{1}H)$	-107.3958
259		$^{4}J(^{1}H_{9},^{1}H_{6})$	2.0907
260		$^{4}J(^{1}H_{9},^{1}H_{7})$	1.9927
261		$^{3}J(^{1}H_{9},^{1}H_{8})$	1.7474
262		$^{1}J(^{13}C,^{13}C)$	71.7312
263		$^{2}J(^{13}C_{1},^{13}C_{3})$	-4.3076
264		$^{2}J(^{13}C_{3},^{13}C_{2})$	9.0472
265		$^{1}J(^{15}N_{4},^{13}C_{1})$	1.3041
266		$^{2}J(^{15}N_{4},^{13}C_{2})$	2.5471
267		$^{1}J(^{15}N_{4},^{13}C_{3})$	-2.6210
268		$^{2}J(^{15}N_{5},^{13}C_{1})$	-6.2860
269		$^{1}J(^{15}N_{5},^{13}C_{2})$	-16.7991
270		$^{1}J(^{15}N_{5},^{13}C_{3})$	-13.2342
271		$^{2}J(^{15}N,^{15}N)$	-1.5988
272		$^{1}J(^{13}C_{1},^{1}H_{6})$	186.4255
273		$^{2}J(^{13}C_{2},^{1}H_{6})$	15.8468
274	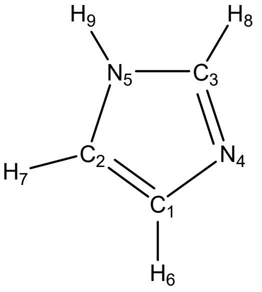	$^{3}J(^{13}C_{3},^{1}H_{6})$	11.5477
275		$^{2}J(^{15}N_{4},^{1}H_{6})$	-9.8654
276		$^{3}J(^{15}N_{5},^{1}H_{6})$	-3.6146
277		$^{2}J(^{13}C_{1},^{1}H_{7})$	8.4732
278	1*H*-imidazole (**36**)	$^{1}J(^{13}C_{2},^{1}H_{7})$	185.0685
279		$^{3}J(^{13}C_{3},^{1}H_{7})$	6.8326
280		$^{3}J(^{15}N_{4},^{1}H_{7})$	-1.1507
281		$^{2}J(^{15}N_{5},^{1}H_{7})$	-4.7047
282		$^{3}J(^{1}H_{7},^{1}H_{6})$	2.1182
283		$^{3}J(^{13}C_{1},^{1}H_{8})$	11.3551
284		$^{3}J(C_{2},^{1}H_{8})$	3.3855
285		$^{1}J(C_{3},^{1}H_{8})$	202.6697
286		$^{2}J(^{15}N_{4},^{1}H_{8})$	-11.2849

287		$^{2}J(^{15}N_{5},^{1}H_{8})$	-8.9869
288		$^{4}J(^{1}H_{8},^{1}H_{6})$	0.3654
289		$^{4}J(^{1}H8,^{1}H_{7})$	1.4269
290		$^{3}J(^{13}C_{1},^{1}H_{9})$	7.8860
291		$^{2}J(^{13}C_{2},^{1}H_{9})$	4.5681
292		$^{2}J(^{13}C_{3},^{1}H_{9})$	4.8237
293		$^{3}J(^{15}N_{4},^{1}H_{9})$	0.1002
294		$^{1}J(^{15}N,^{1}H)$	-97.2237
295		$^{4}J(^{1}H_{9},^{1}H_{6})$	1.8245
296		$^{3}J(^{1}H_{9},^{1}H_{7})$	2.1666
297		$^{3}J(^{1}H_{9},^{1}H_{8})$	1.0517
298		$^{1}J(^{13}C_{1},^{13}C_{2})$	57.3635
299		$^{2}J(^{13}C_{2},^{13}C_{3})$	-4.0230
300		$^{2}J(^{13}C_{1},^{13}C_{4})$	-3.5590
301		$^{1}J(^{13}C_{2},^{13}C_{4})$	58.1669
302		$^{3}J(^{13}C_{3},^{13}C_{4})$	15.2805
303		$^{2}J(^{13}C_{5},^{13}C_{4})$	-7.1388
304		$^{3}J(^{15}N_{6},^{13}C_{1})$	-4.6469
305		$^{2}J(^{15}N_{6},^{13}C_{2})$	3.0134
306		$^{1}J(^{15}N,^{13}C)$	-1.7094
307		$^{1}J(^{13}C_{1},^{1}H_{7})$	156.8267
308		$^{2}J(^{13}C_{2},^{1}H_{7})$	-0.1883
309		$^{3}J(^{13}C_{4},^{1}H_{7})$	6.8339
310		$^{4}J(^{15}N,^{1}H)$	0.5107
311		$^{2}J(^{13}C_{1},^{1}H_{8})$	-0.1314
312	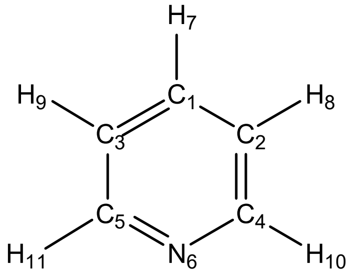 pyridine (**37**)	$^{1}J(^{13}C_{2},^{1}H_{8})$	159.6222
313		$^{3}J(^{13}C_{3},^{1}H_{8})$	6.6899
314		$^{2}J(^{13}C_{4},^{1}H_{8})$	2.1496
315		$^{4}J(^{13}C_{5},^{1}H_{8})$	-1.2316
316		$^{3}J(^{15}N,^{1}H)$	-1.6388
317		$^{3}J(^{1}H_{7},^{1}H_{8})$	8.0857
318		$^{4}J(^{1}H_{8},^{1}H_{9})$	0.9037
319		$^{3}J(^{13}C_{1},^{1}H_{10})$	6.6658
320		$^{2}J(^{13}C_{2},^{1}H_{10})$	7.6884
321		$^{4}J(^{13}C_{3},^{1}H_{10})$	-2.0167
322		$^{1}J(^{13}C_{4},^{1}H_{10})$	173.9992
323		$^{3}J(^{13}C_{5},^{1}H_{10})$	11.3255
324		$^{2}J(^{15}N,^{1}H)$	-9.9175
325		$^{4}J(^{1}H_{7},^{1}H_{10})$	1.4079
326		$^{3}J(^{1}H_{8},^{1}H_{10})$	5.4743
327		$^{5}J(^{1}H,^{1}H)$	1.3807
328		$^{4}J(^{1}H_{10},^{1}H_{11})$	-0.5692
329		$^{2}J(^{15}N,^{13}C)$	-4.4172
330		$^{1}J(^{13}C_{2},^{13}C_{3})$	55.1481
331		$^{1}J(^{15}N,^{13}C)$	-15.9625

332		$^{1}J(^{13}C_5,^{13}C_3)$	71.2349
333		$^{2}J(^{13}C_2,^{13}C_5)$	1.1686
334		$^{2}J(^{13}C_4,^{13}C_5)$	7.5009
335		$^{3}J(^{15}N,^{1}H)$	-5.5308
336		$^{1}J(^{13}C_3,^{1}H_7)$	167.9389
337		$^{2}J(^{13}C_2,^{1}H_7)$	3.5779
338		$^{2}J(^{13}C_5,^{1}H_7)$	7.3266
339	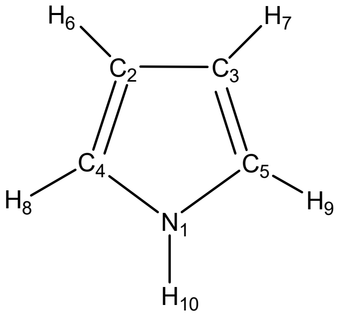	$^{3}J(^{13}C_4,^{1}H_7)$	7.3634
340		$^{3}J(^{1}H_6,^{1}H_7)$	3.6688
341		$^{2}J(^{15}N,^{1}H)$	-4.1038
342		$^{2}J(^{13}C_3,^{1}H_9)$	6.4922
343		$^{3}J(^{13}C_2,^{1}H_9)$	7.6333
344		$^{1}J(^{13}C_5,^{1}H_9)$	180.4356
345		$^{3}J(^{13}C_4,^{1}H_9)$	6.0298
346	1*H*-pyrrole (**38**)	$^{3}J(^{1}H_7,^{1}H_9)$	3.6492
347		$^{4}J(^{1}H_6,^{1}H_9)$	1.0575
348		$^{4}J(^{1}H_8,^{1}H_9)$	2.1887
349		$^{1}J(^{15}N,^{1}H)$	-97.0426
350		$^{3}J(^{13}C_3,^{1}H_{10})$	7.1424
351		$^{2}J(^{13}C_5,^{1}H_{10})$	3.7450
352		$^{4}J(^{1}H_7,^{1}H_{10})$	2.5756
353		$^{3}J(^{1}H_9,^{1}H_{10})$	2.8532
354		$^{1}J(^{13}C_2,^{13}C_1)$	73.7637
355		$^{2}J(^{13}C,^{13}C)$	0.0279
356		$^{1}J(^{13}C_2,^{13}C_3)$	43.9392
357		$^{1}J(^{13}C_1,^{1}H_4)$	155.5607
358		$^{2}J(^{13}C_2,^{1}H_4)$	-1.6556
359		$^{3}J(^{13}C_3,^{1}H_4)$	11.9406
360		$^{1}J(^{13}C_1,^{1}H_5)$	151.6664
361		$^{2}J(^{13}C_2,^{1}H_5)$	-3.2263
362		$^{3}J(^{13}C_3,^{1}H_5)$	7.7179
363		$^{2}J(^{1}H_4,^{1}H_5)$	0.2401
364	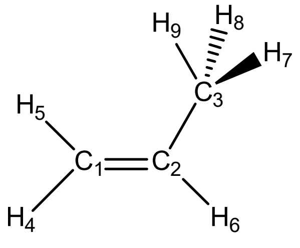	$^{2}J(^{13}C_1,^{1}H_6)$	-0.4766
365		$^{1}J(^{13}C_2,^{1}H_6)$	149.3861
366		$^{2}J(^{13}C_3,^{1}H_6)$	4.1774
367		$^{3}J(^{1}H_4,^{1}H_6)$	10.9208
368	prop-1-ene (**39**)	$^{3}J(^{1}H_5,^{1}H_6)$	16.3636
369		$^{3}J(^{13}C_1,^{1}H_7)$	6.2200
370		$^{2}J(^{13}C_2,^{1}H_7)$	-8.0167
371		$^{1}J(^{13}C_3,^{1}H_7)$	122.7888
372		$^{4}J(^{1}H_4,^{1}H_7)$	-2.6146
373		$^{4}J(^{1}H_5,^{1}H_7)$	-2.8528
374		$^{3}J(^{1}H_6,^{1}H_7)$	3.8907
375		$^{2}J(^{1}H_7,^{1}H_8)$	-18.9752
376		$^{3}J(^{13}C_1,^{1}H_9)$	6.4541

377		$^{2}J(^{13}C_{2},^{1}H_{9})$	-5.9946
378		$^{1}J(^{13}C_{3},^{1}H_{9})$	123.8785
379		$^{4}J(^{1}H_{4},^{1}H_{9})$	-0.2816
380		$^{4}J(^{1}H_{5},^{1}H_{9})$	-0.5821
381		$^{3}J(^{1}H_{6},^{1}H_{9})$	12.1522
382		$^{2}J(^{1}H_{7},^{1}H_{9})$	-13.9647
383	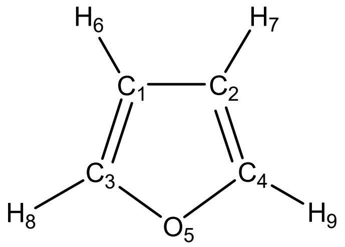 furan (**40**)	$^{1}J(^{13}C_{1},^{13}C_{2})$	52.8754
384		$^{1}J(^{13}C_{1},^{13}C_{3})$	74.6173
385		$^{2}J(^{13}C_{2},^{13}C_{3})$	0.0940
386		$^{2}J(^{13}C_{3},^{13}C_{4})$	4.0661
387		$^{1}J(^{13}C_{1},^{1}H_{6})$	171.7745
388		$^{2}J(^{13}C_{2},^{1}H_{6})$	3.3553
389		$^{2}J(^{13}C_{3},^{1}H_{6})$	9.2310
390		$^{3}J(^{13}C_{4},^{1}H_{6})$	6.7635
391		$^{3}J(^{1}H_{6},^{1}H_{7})$	3.2889
392		$^{2}J(^{13}C_{1},^{1}H_{8})$	12.3928
393		$^{3}J(^{13}C_{2},^{1}H_{8})$	6.3156
394		$^{1}J(^{13}C_{3},^{1}H_{8})$	197.6437
395		$^{3}J(^{13}C_{4},^{1}H_{8})$	6.7415
396		$^{3}J(^{1}H_{6},^{1}H_{8})$	2.7925
397		$^{4}J(^{1}H_{7},^{1}H_{8})$	0.4458
398		$^{4}J(^{1}H_{8},^{1}H_{9})$	1.7507
399	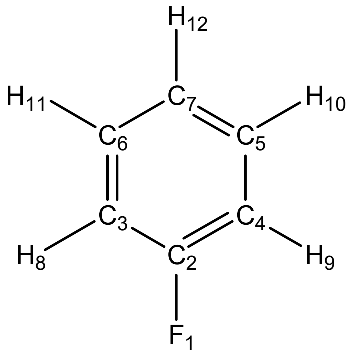 1-fluorobenzene (**41**)	$^{1}J(^{19}F,^{13}C)$	-246.6680
400		$^{2}J(^{19}F,^{13}C)$	22.1139
401		$^{1}J(^{13}C_{2},^{13}C_{3})$	74.1870
402		$^{2}J(^{13}C_{3},^{13}C_{4})$	2.9889
403		$^{3}J(^{19}F,^{13}C)$	5.3798
404		$^{2}J(^{13}C_{2},^{13}C_{6})$	-0.3770
405		$^{1}J(^{13}C_{3},^{13}C_{6})$	60.6181
406		$^{3}J(^{13}C_{4},^{13}C_{6})$	7.8267
407		$^{2}J(^{13}C_{5},^{13}C_{6})$	-2.3281
408		$^{4}J(^{19}F,^{13}C)$	5.4509
409		$^{3}J(^{13}C_{7},^{13}C_{2})$	11.6264
410		$^{2}J(^{13}C_{3},^{13}C_{7})$	-3.8281
411		$^{1}J(^{13}C_{6},^{13}C_{7})$	59.8362
412		$^{3}J(^{19}F,^{1}H)$	6.7193
413		$^{2}J(^{13}C_{2},^{1}H_{8})$	-6.2188
414		$^{1}J(^{13}C_{3},^{1}H_{8})$	159.9446
415		$^{3}J(^{13}C_{4},^{1}H_{8})$	4.4418
416		$^{2}J(^{13}C_{6},^{1}H_{8})$	-1.2771
417		$^{4}J(^{13}C_{5},^{1}H_{8})$	-1.1773
418		$^{3}J(^{13}C_{7},^{1}H_{8})$	7.8312
419		$^{4}J(^{1}H_{8},^{1}H_{9})$	2.1917
420		$^{4}J(^{19}F,^{1}H)$	4.9781
421		$^{3}J(^{13}C_{2},^{1}H_{11})$	10.7791

422		$^{2}J(^{13}C_3,^{1}H_{11})$	0.0198
423		$^{4}J(^{13}C_4,^{1}H_{11})$	-1.8554
424		$^{1}J(^{13}C_6,^{1}H_{11})$	157.2111
425		$^{3}J(^{13}C_5,^{1}H_{11})$	9.0125
426		$^{2}J(^{13}C_7,^{1}H_{11})$	-0.2987
427		$^{3}J(^{1}H_8,^{1}H_{11})$	8.8420
428		$^{5}J(^{1}H_9,^{1}H_{11})$	0.9510
429		$^{4}J(^{1}H_{10},^{1}H_{11})$	1.2517
430		$^{5}J(^{19}F,^{1}H)$	-1.1287
431		$^{4}J(^{13}C_2,^{1}H_{12})$	-2.2251
432		$^{3}J(^{13}C_3,^{1}H_{12})$	8.2623
433		$^{2}J(^{13}C_6,^{1}H_{12})$	0.6142
434		$^{1}J(^{13}C_7,^{1}H_{12})$	158.8712
435		$^{4}J(^{1}H_8,^{1}H_{12})$	0.6437
436		$^{3}\mathrm{J}(^{1}H_{11},^{1}H_{12})$	8.0656

[1] Atoms are given numbers only when it is necessary, to distinguish between the eponymous types of SSCCs.

Table S4. Symmetry independent values of SSCC (in Hz) in molecules of set 1 calculated at the SOPPA(CCSD) level with the pecJ-1-old basis set.

#	Molecule	Type of SSCC [1]	SSCC value
1	$H_2C{=}C{=}CH_2$ propa-1,2-diene (**1**)	$^{1}J(^{13}C,^{13}C)$	103.0619
2		$^{2}J(^{13}C,^{13}C)$	7.8628
3		$^{2}J(^{13}C,^{1}H)$	-6.9071
4		$^{1}J(^{13}C,^{1}H)$	163.5071
5		$^{3}J(^{13}C,^{1}H)$	8.1596
6		$^{2}J(^{1}H,^{1}H)$	-14.7811
7		$^{4}J(^{1}H,^{1}H)$	-9.3629
8	$F_2C{=}CF_2$ Perfluoroethene (**2**)	$^{1}J(^{13}C,^{13}C)$	195.0125
9		$^{1}J(^{13}C,^{19}F)$	-260.8703
10		$^{2}J(^{13}C,^{19}F)$	50.6640
11		$^{2}J(^{19}F,^{19}F)$	133.3532
12		$^{3}J_{cis}(^{19}F,^{19}F)$	79.9855
13		$^{3}J_{trans}(^{19}F,^{19}F)$	-118.0644
14	$HC{\equiv}CH$ ethyne (**3**)	$^{1}J(^{13}C,^{13}C)$	190.2688
15		$^{1}J(^{13}C,^{1}H)$	246.8020
16		$^{2}J(^{13}C,^{1}H)$	48.9559
17		$^{3}J(^{1}H,^{1}H)$	11.0714
18	$H_2C{=}CH_2$ ethene (**4**)	$^{1}J(^{13}C,^{13}C)$	71.4433
19		$^{1}J(^{13}C,^{1}H)$	151.2861
20		$^{2}J(^{13}C,^{1}H)$	-3.9790
21		$^{3}J_{cis}(^{1}H,^{1}H)$	11.8488
22		$^{2}J(^{1}H,^{1}H)$	-1.0902
23		$^{3}J_{trans}(^{1}H,^{1}H)$	18.1738
24	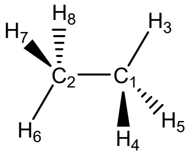 ethane (**5**)	$^{1}J(^{13}C,^{13}C)$	35.2041
25		$^{1}J(^{13}C,^{1}H)$	119.7402
26		$^{2}J(^{13}C,^{1}H)$	-5.3045
27		$^{3}J_{trans}(^{1}H_3,^{1}H_6)$	15.3505
28		$^{2}J(^{1}H,^{1}H)$	-14.8011
29		$^{3}J_{gauche}(^{1}H_4,^{1}H_6)$	3.6841
30	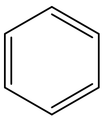 benzene (**6**)	$^{3}J(^{13}C,^{13}C)$	11.1635
31		$^{1}J(^{13}C,^{13}C)$	59.1404
32		$^{2}J(^{13}C,^{13}C)$	-3.8462
33		$^{1}J(^{13}C,^{1}H)$	151.3035
34		$^{4}J(^{13}C,^{1}H)$	-1.9498
35		$^{2}J(^{13}C,^{1}H)$	-0.8131
36		$^{3}J(^{13}C,^{1}H)$	7.8617
37		$^{3}J(^{1}H,^{1}H)$	7.9092
38		$^{4}J(^{1}H,^{1}H)$	0.4728
39		$^{5}J(^{1}H,^{1}H)$	1.0548
40	CF_4 perfluoromethane (**7**)	$^{1}J(^{19}F,^{13}C)$	-253.8965
41		$^{2}J(^{19}F,^{19}F)$	45.2809

42	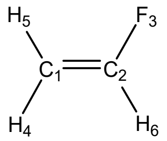 fluoroethene (**8**)	$^{1}J(^{13}C,^{13}C)$	87.8417
43		$^{2}J(^{19}F,^{13}C)$	12.2679
44		$^{1}J(^{19}F,^{13}C)$	-249.5648
45		$^{1}J(^{13}C_1,^{1}H_4)$	156.3512
46		$^{2}J(^{13}C_2,^{1}H_4)$	5.8536
47		$^{3}J_{trans}(^{19}F_3,^{1}H_4)$	40.1387
48		$^{1}J(^{13}C_1,^{1}H_5)$	155.0797
49		$^{2}J(^{13}C_2,^{1}H_5)$	-10.8404
50		$^{3}J_{cis}(^{19}F_3,^{1}H_5)$	13.0377
51		$^{2}J(^{1}H,^{1}H)$	-5.8526
52		$^{2}J(^{13}C_1,^{1}H_6)$	11.8093
53		$^{1}J(^{13}C_2,^{1}H_6)$	189.9695
54		$^{2}J(^{19}F,^{1}H)$	82.1674
55		$^{3}J_{cis}(^{1}H_6,^{1}H_4)$	5.9741
56		$^{3}J_{trans}(^{1}H_6,^{1}H_5)$	12.6118
57	CH_2F_2 difluoromethane (**9**)	$^{1}J(^{19}F,^{13}C)$	-219.5315
58		$^{2}J(^{19}F,^{19}F)$	336.9739
59		$^{1}J(^{13}C,^{1}H)$	172.1671
60		$^{2}J(^{19}F,^{1}H)$	48.7801
61		$^{2}J(^{1}H,^{1}H)$	-0.7443
62	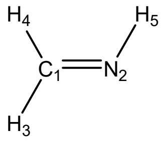 methanimine (**10**)	$^{1}J(^{15}N,^{13}C)$	-4.2836
63		$^{1}J(^{13}C_1,^{1}H_3)$	168.1406
64		$^{2}J(^{15}N_2,^{1}H_3)$	-8.8677
65		$^{1}J(^{13}C_1,^{1}H_4)$	152.2996
66		$^{2}J(^{15}N_2,^{1}H_4)$	3.6177
67		$^{2}J(^{1}H,^{1}H)$	15.0406
68		$^{2}J(^{13}C,^{1}H)$	-14.2136
69		$^{1}J(^{15}N,^{1}H)$	-49.0099
70		$^{3}J_{trans}(^{1}H_5,^{1}H_3)$	24.1694
71		$^{3}J_{cis}(^{1}H_5,^{1}H_4)$	18.0504
72	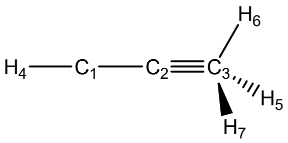 prop-1-yne (**11**)	$^{1}J(^{13}C_1,^{13}C_2)$	190.2140
73		$^{2}J(^{13}C,^{13}C)$	12.7319
74		$^{1}J(^{13}C_2,^{13}C_3)$	70.7525
75		$^{1}J(^{13}C_1,^{1}H_4)$	245.6790
76		$^{2}J(^{13}C_2,^{1}H_4)$	48.6487
77		$^{3}J(^{13}C_3,^{1}H_4)$	4.3441
78		$^{3}J(^{13}C_1,^{1}H_6)$	3.7758
79		$^{2}J(^{13}C_2,^{1}H_6)$	-12.3790
80		$^{1}J(^{13}C_3,^{1}H_6)$	125.4915
81		$^{4}J(^{1}H,^{1}H)$	-3.9759
82		$^{2}J(^{1}H,^{1}H)$	-17.8790
83		$^{1}J(^{13}C,^{13}C)$	42.2508
84		$^{1}J(^{13}C_1,^{1}H_4)$	160.6426
85		$^{2}J(^{13}C_2,^{1}H_4)$	26.4266
86		$^{2}J(^{13}C_1,^{1}H_5)$	-8.5010

87	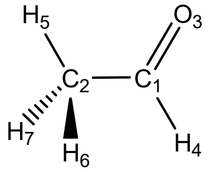 acetaldehyde (**12**)	$^{1}J(^{13}C_2,^{1}H_5)$	129.2372
88		$^{3}J_{trans}(^{1}H_5,^{1}H_4)$	7.7133
89		$^{2}J(^{13}C_1,^{1}H_6)$	-6.8661
90		$^{1}J(^{13}C_2,^{1}H_6)$	117.6731
91		$^{3}J_{gauche}(^{1}H_6,^{1}H_4)$	0.3319
92		$^{2}J(^{1}H_6,^{1}H_5)$	-14.0062
93		$^{2}J(^{1}H_6,^{1}H_7)$	-20.4960
94	$N{\equiv}C{-}CH_3$ acetonitrile (**13**)	$^{1}J(^{13}C,^{13}C)$	62.0964
95		$^{2}J(^{15}N,^{13}C)$	2.3914
96		$^{1}J(^{15}N,^{13}C)$	-15.5856
97		$^{1}J(^{13}C,^{1}H)$	128.8630
98		$^{2}J(^{13}C,^{1}H)$	-11.4384
99		$^{3}J(^{15}N,^{1}H)$	-1.3382
100		$^{2}J(^{1}H,^{1}H)$	-17.9944
101	$H_3C{-}F$ fluoromethane (**14**)	$^{1}J(^{19}F,^{13}C)$	-155.5363
102		$^{1}J(^{13}C,^{1}H)$	141.3025
103		$^{2}J(^{19}F,^{1}H)$	47.1785
104		$^{2}J(^{1}H,^{1}H)$	-11.7780
105	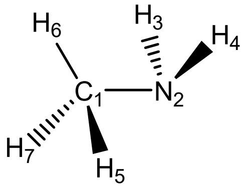 methanamine (**15**)	$^{1}J(^{15}N,^{13}C)$	-6.2352
106		$^{2}J(^{13}C,^{1}H)$	-4.1012
107		$^{1}J(^{15}N,^{1}H)$	-63.9598
108		$^{2}J(^{1}H_3,^{1}H_4)$	-12.2898
109		$^{1}J(^{13}C_1,^{1}H_5)$	127.1265
110		$^{2}J(^{15}N_2,^{1}H_5)$	-1.3319
111		$^{3}J(^{1}H_5,^{1}H_4)$	2.6499
112		$^{3}J(^{1}H_5,^{1}H_3)$	15.2485
113		$^{2}J(^{1}H_7,^{1}H_5)$	-16.3240
114		$^{1}J(^{13}C_1,^{1}H_6)$	123.9184
115		$^{2}J(^{15}N_2,^{1}H_6)$	1.1936
116		$^{3}J(^{1}H_6,^{1}H_4)$	2.0943
117		$^{2}J(^{1}H_6,^{1}H_5)$	-12.1143
118	CH_4 methane (**16**)	$^{1}J(^{13}C,^{1}H)$	120.0472
119		$^{2}J(^{1}H,^{1}H)$	-14.6221
120	CHF_3 fluoroform (**17**)	$^{1}J(^{13}C,^{1}H)$	220.9980
121		$^{1}J(^{19}F,^{13}C)$	-256.2392
122		$^{2}J(^{19}F,^{1}H)$	72.1365
123		$^{2}J(^{19}F,^{19}F)$	144.4275
124	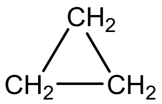 cyclopropane (**18**)	$^{1}J(^{13}C,^{13}C)$	14.0763
125		$^{1}J(^{13}C,^{1}H)$	153.5440
126		$^{2}J(^{13}C,^{1}H)$	-3.2325
127		$^{2}J(^{1}H,^{1}H)$	-6.8783
128		$^{3}J_{cis}(^{1}H,^{1}H)$	8.8290
129		$^{3}J_{trans}(^{1}H,^{1}H)$	4.8578
130	$F{-}C{\equiv}C{-}F$ 1,2-difluoroethyne (**19**)	$^{1}J(^{13}C,^{13}C)$	404.5293
131		$^{1}J(^{19}F,^{13}C)$	-268.7655

132		$^{2}J(^{19}F,^{13}C)$	44.1173
133		$^{3}J(^{19}F,^{19}F)$	-13.4889
134	F−C≡N<br>fluoroformonitrile (**20**)	$^{1}J(^{19}F,^{13}C)$	-391.7917
135		$^{1}J(^{15}N,^{13}C)$	-3.3377
136		$^{2}J(^{19}F,^{15}N)$	49.6430
137	$H_2C{=}CF_2$<br>1,1-difluoroethene (**21**)	$^{1}J(^{13}C,^{13}C)$	116.8873
138		$^{2}J(^{19}F,^{13}C)$	28.0179
139		$^{1}J(^{19}F,^{13}C)$	-276.3287
140		$^{2}J(^{19}F,^{19}F)$	43.1589
141		$^{1}J(^{13}C,^{1}H)$	161.9983
142		$^{2}J(^{13}C,^{1}H)$	-2.9791
143		$^{3}J_{cis}(^{19}F,^{1}H)$	-1.7058
144		$^{3}J_{trans}(^{19}F,^{1}H)$	26.7233
145		$^{2}J(^{1}H,^{1}H)$	-7.6040
146	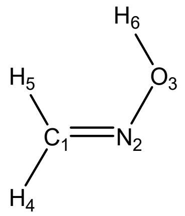<br>formaldehyde oxime (*Z*) (**22**)	$^{1}J(^{15}N,^{13}C)$	-3.2078
147		$^{1}J(^{13}C_1,^{1}H_4)$	180.1690
148		$^{2}J(^{15}N_2,^{1}H_4)$	-11.1708
149		$^{1}J(^{13}C_1,^{1}H_5)$	150.6331
150		$^{2}J(^{15}N_2,^{1}H_5)$	2.8943
151		$^{2}J(^{1}H,^{1}H)$	5.2094
152		$^{3}J(^{13}C,^{1}H)$	4.2501
153		$^{2}J(^{15}N_2,^{1}H_6)$	1.5150
154		$^{4}J(^{1}H_6,^{1}H_4)$	-1.6494
155		$^{4}J(^{1}H_6,^{1}H_5)$	1.7813
156	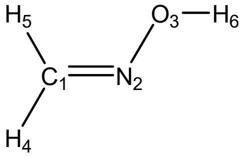<br>formaldehyde oxime (*E*) (**23**)	$^{1}J(^{15}N,^{13}C)$	-6.3541
157		$^{1}J(^{13}C_1,^{1}H_4)$	173.6475
158		$^{2}J(^{15}N_2,^{1}H_4)$	-11.9621
159		$^{1}J(^{13}C_1,^{1}H_5)$	159.6430
160		$^{2}J(^{15}N_2,^{1}H_5)$	2.8531
161		$^{2}J(^{1}H,^{1}H)$	6.9112
162		$^{3}J(^{13}C,^{1}H)$	10.8986
163		$^{2}J(^{15}N_2,^{1}H_6)$	-1.9474
164		$^{4}J(^{1}H_6,^{1}H_4)$	0.7386
165		$^{4}J(^{1}H_6,^{1}H_5)$	-1.4808
166	H−C≡C−F<br>fluoroethyne (**24**)	$^{1}J(^{13}C,^{13}C)$	273.2212
167		$^{2}J(^{19}F,^{13}C)$	26.5068
168		$^{1}J(^{19}F,^{13}C)$	-278.7960
169		$^{1}J(^{13}C,^{1}H)$	276.1838
170		$^{2}J(^{13}C,^{1}H)$	63.4222
171		$^{3}J(^{19}F,^{1}H)$	9.6789
172	H−C≡N<br>hydrogen cyanide (**25**)	$^{1}J(^{15}N,^{13}C)$	-17.1356
173		$^{1}J(^{13}C,^{1}H)$	256.6963
174		$^{2}J(^{15}N,^{1}H)$	-8.3103
175		$^{1}J(^{13}C,^{13}C)$	144.0263
176		$^{2}J(^{19}F_3,^{13}C_1)$	65.5275

177	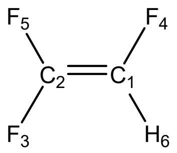 1,1,2-trifluoroethene (**26**)	$^{1}J(^{19}F_3,^{13}C_2)$	-264.1930
178		$^{1}J(^{19}F_4,^{13}C_1)$	-226.7864
179		$^{2}J(^{19}F_4,^{13}C_2)$	37.6602
180		$^{3}J_{trans}(^{19}F_3,^{19}F_4)$	-123.4231
181		$^{2}J(^{19}F_5,^{13}C_1)$	20.1450
182		$^{1}J(^{19}F_5,^{13}C_2)$	-277.1044
183		$^{2}J(^{19}F,^{19}F)$	91.3191
184		$^{3}J_{cis}(^{19}F_4,^{19}F_5)$	40.5779
185		$^{1}J(^{13}C,^{1}H)$	202.0828
186		$^{2}J(^{13}C,^{1}H)$	12.9245
187		$^{3}J_{cis}(^{19}F_3,^{1}H_6)$	-5.8448
188		$^{2}J(^{19}F,^{1}H)$	71.9422
189		$^{3}J_{trans}(^{19}F_5,^{1}H_6)$	7.8406
190	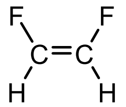 (*Z*)-1,2-difluoroethene (**27**)	$^{1}J(^{13}C,^{13}C)$	100.9962
191		$^{1}J(^{19}F,^{13}C)$	-244.4641
192		$^{2}J(^{19}F,^{13}C)$	9.7700
193		$^{3}J(^{19}F,^{19}F)$	-9.2036
194		$^{1}J(^{13}C,^{1}H)$	194.2648
195		$^{2}J(^{13}C,^{1}H)$	21.4530
196		$^{2}J(^{19}F,^{1}H)$	72.1032
197		$^{3}J(^{19}F,^{1}H)$	13.9329
198		$^{3}J(^{1}H,^{1}H)$	3.6085
199	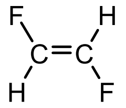 (*E*)-1,2-difluoroethene (**28**)	$^{1}J(^{13}C,^{13}C)$	114.6005
200		$^{1}J(^{19}F,^{13}C)$	-232.3326
201		$^{2}J(^{19}F,^{13}C)$	50.1346
202		$^{3}J(^{19}F,^{19}F)$	-134.5262
203		$^{2}J(^{13}C,^{1}H)$	3.3086
204		$^{1}J(^{13}C,^{1}H)$	193.4501
205		$^{3}J(^{19}F,^{1}H)$	-0.0752
206		$^{2}J(^{19}F,^{1}H)$	75.8283
207		$^{3}J(^{1}H,^{1}H)$	9.8541
208	HF hydrogen fluoride (**29**)	$^{1}J(^{19}F,^{1}H)$	545.9233
209	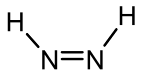 diazene (*Z*) (**31**)	$^{1}J(^{15}N,^{15}N)$	-19.7853
210		$^{1}J(^{15}N,^{1}H)$	-34.2511
211		$^{2}J(^{15}N,^{1}H)$	1.2896
212		$^{3}J(^{1}H,^{1}H)$	38.3649
213	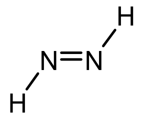 diazene (*E*) (**30**)	$^{1}J(^{15}N,^{15}N)$	-19.8451
214		$^{2}J(^{15}N,^{1}H)$	-0.1676
215		$^{1}J(^{15}N,^{1}H)$	-44.1338
216		$^{3}J(^{1}H,^{1}H)$	37.9707
217		$^{1}J(^{15}N,^{15}N)$	1.5453
218		$^{1}J(^{15}N,^{1}H)$	-57.7402
219		$^{2}J(^{15}N,^{1}H)$	-1.4534
220		$^{2}J(^{1}H,^{1}H)$	-16.9412

221	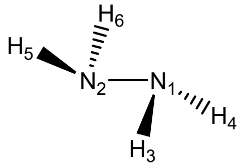 hydrazine (**32**)	$^{3}J(^{1}H_5,^{1}H_3)$	1.4799
222		$^{3}J(^{1}H_4,^{1}H_5)$	13.1576
223	N_2 molecular nitrogen (**33**)	$^{1}J(^{15}N,^{15}N)$	-3.36973
224	NH_3 ammonia (**34**)	$^{1}J(^{15}N,^{1}H)$	-60.2057
225		$^{2}J(^{1}H,^{1}H)$	-12.9138
226	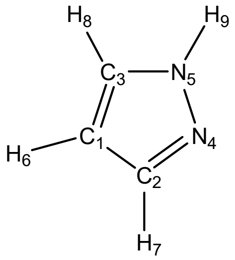 1*H*-pyrazole (**35**)	$^{1}J(^{13}C_1,^{13}C_2)$	54.7371
227		$^{1}J(^{13}C_3,^{13}C_1)$	69.6943
228		$^{2}J(^{13}C_3,^{13}C_2)$	1.4681
229		$^{2}J(^{15}N_4,^{13}C_1)$	2.705495
230		$^{1}J(^{15}N_4,^{13}C_2)$	-2.40124
231		$^{2}J(^{15}N_4,^{13}C_3)$	0.829029
232		$^{2}J(^{15}N_5,^{13}C_1)$	-5.70851
233		$^{2}J(^{15}N_5,^{13}C_2)$	-0.73883
234		$^{1}J(^{15}N_5,^{13}C_3)$	-15.9332
235		$^{1}J(^{15}N,^{15}N)$	-11.3199
236		$^{1}J(^{13}C_1,^{1}H_6)$	167.8782
237		$^{2}J(^{13}C_2,^{1}H_6)$	4.5152
238		$^{2}J(^{13}C3,^{1}H_6)$	6.8068
239		$^{3}J(^{15}N_4,^{1}H_6)$	-1.02892
240		$^{3}J(^{15}N_5,^{1}H_6)$	-5.70136
241		$^{2}J(^{13}C_1,^{1}H_7)$	9.6706
242		$^{1}J(^{13}C_2,^{1}H_7)$	177.123
243		$^{3}J(^{13}C_3,^{1}H_7)$	4.7292
244		$^{2}J(^{15}N_4,^{1}H_7)$	-11.317
245		$^{3}J(^{15}N_5,^{1}H_7)$	-8.72079
246		$^{3}J(^{1}H_7,^{1}H_6)$	1.7368
247		$^{2}J(^{13}C_1,^{1}H_8)$	6.0318
248		$^{3}J(^{13}C_2,^{1}H_8)$	7.9094
249		$^{1}J(^{13}C_3,^{1}H_8)$	175.7567
250		$^{3}J(^{15}N_4,^{1}H_8)$	0.422229
251		$^{2}J(^{15}N_5,^{1}H_8)$	-4.25919
252		$^{3}J(^{1}H_8,^{1}H_6)$	2.8945
253		$^{4}J(^{1}H_8,^{1}H_7)$	-0.1973
254		$^{3}J(^{13}C_1,^{1}H_9)$	5.3363
255		$^{3}J(^{13}C_2,^{1}H_9)$	10.1138
256		$^{2}J(^{13}C_3,^{1}H_9)$	8.5999
257		$^{2}J(^{15}N_4,^{1}H_9)$	-8.35846
258		$^{1}J(^{15}N,^{1}H)$	-105.544
259		$^{4}J(^{1}H_9,^{1}H_6)$	1.6579
260		$^{4}J(^{1}H_9,^{1}H_7)$	1.5389
261		$^{3}J(^{1}H_9,^{1}H_8)$	1.5425

262		$^{1}J(^{13}C,^{13}C)$	71.1588
263		$^{2}J(^{13}C_1,^{13}C_3)$	-4.4463
264		$^{2}J(^{13}C_3,^{13}C_2)$	9.116
265		$^{1}J(^{15}N_4,^{13}C_1)$	0.884297
266		$^{2}J(^{15}N_4,^{13}C_2)$	2.272745
267		$^{1}J(^{15}N_4,^{13}C_3)$	-2.81814
268		$^{2}J(^{15}N_5,^{13}C_1)$	-6.17184
269		$^{1}J(^{15}N_5,^{13}C_2)$	-17.6354
270		$^{1}J(^{15}N_5,^{13}C_3)$	-13.717
271		$^{2}J(^{15}N,^{15}N)$	-1.33628
272		$^{1}J(^{13}C_1,^{1}H_6)$	180.8185
273		$^{2}J(^{13}C_2,^{1}H_6)$	14.288
274		$^{3}J(^{13}C_3,^{1}H_6)$	11.3784
275		$^{2}J(^{15}N_4,^{1}H_6)$	-9.2342
276		$^{3}J(^{15}N_5,^{1}H_6)$	-3.51488
277		$^{2}J(^{13}C_1,^{1}H_7)$	7.2679
278		$^{1}J(^{13}C_2,^{1}H_7)$	179.5198
279	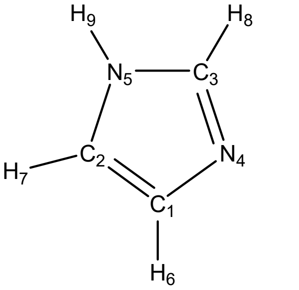	$^{3}J(^{13}C_3,^{1}H_7)$	6.5167
280		$^{3}J(^{15}N_4,^{1}H_7)$	-1.05403
281		$^{2}J(^{15}N_5,^{1}H_7)$	-4.37155
282		$^{3}J(^{1}H_7,^{1}H_6)$	2.0624
283	1*H*-imidazole (**36**)	$^{3}J(^{13}C_1,^{1}H_8)$	11.3211
284		$^{3}J(C_2,^{1}H_8)$	3.1224
285		$^{1}J(C_3,^{1}H_8)$	196.6591
286		$^{2}J(^{15}N_4,^{1}H_8)$	-10.4358
287		$^{2}J(^{15}N_5,^{1}H_8)$	-8.49986
288		$^{4}J(^{1}H_8,^{1}H_6)$	-0.0779
289		$^{4}J(^{1}H8,^{1}H_7)$	1.0978
290		$^{3}J(^{13}C_1,^{1}H_9)$	7.7023
291		$^{2}J(^{13}C_2,^{1}H_9)$	4.0682
292		$^{2}J(^{13}C_3,^{1}H_9)$	4.2235
293		$^{3}J(^{15}N_4,^{1}H_9)$	0.252636
294		$^{1}J(^{15}N,^{1}H)$	-95.3036
295		$^{4}J(^{1}H_9,^{1}H_6)$	1.4019
296		$^{3}J(^{1}H_9,^{1}H_7)$	1.9104
297		$^{3}J(^{1}H_9,^{1}H_8)$	0.8892
298		$^{1}J(^{13}C_1,^{13}C_2)$	57.057
299		$^{2}J(^{13}C_2,^{13}C_3)$	-4.4496
300		$^{2}J(^{13}C_1,^{13}C_4)$	-4.0117
301	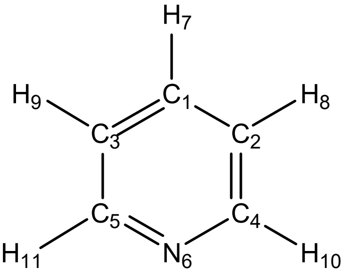	$^{1}J(^{13}C_2,^{13}C_4)$	57.6849
302		$^{3}J(^{13}C_3,^{13}C_4)$	15.0962
303		$^{2}J(^{13}C_5,^{13}C_4)$	-7.4737
304		$^{3}J(^{15}N_6,^{13}C_1)$	-4.41489
305	pyridine (**37**)	$^{2}J(^{15}N_6,^{13}C_2)$	2.782366
306		$^{1}J(^{15}N,^{13}C)$	-2.00875

307		$^{1}J(^{13}C_1,^{1}H_7)$	152.4035
308		$^{2}J(^{13}C_2,^{1}H_7)$	-1.0851
309		$^{3}J(^{13}C_4,^{1}H_7)$	6.8979
310		$^{4}J(^{15}N,^{1}H)$	0.511725
311		$^{2}J(^{13}C_1,^{1}H_8)$	-0.9548
312		$^{1}J(^{13}C_2,^{1}H_8)$	154.8262
313		$^{3}J(^{13}C_3,^{1}H_8)$	6.7067
314		$^{2}J(^{13}C_4,^{1}H_8)$	1.2248
315		$^{4}J(^{13}C_5,^{1}H_8)$	-1.5121
316		$^{3}J(^{15}N,^{1}H)$	-1.57151
317		$^{3}J(^{1}H_7,^{1}H_8)$	7.8893
318		$^{4}J(^{1}H_8,^{1}H_9)$	0.4829
319		$^{3}J(^{13}C_1,^{1}H_{10})$	6.7144
320		$^{2}J(^{13}C_2,^{1}H_{10})$	6.5833
321		$^{4}J(^{13}C_3,^{1}H_{10})$	-2.3229
322		$^{1}J(^{13}C_4,^{1}H_{10})$	169.0686
323		$^{3}J(^{13}C_5,^{1}H_{10})$	11.3376
324		$^{2}J(^{15}N,^{1}H)$	-9.36508
325		$^{4}J(^{1}H_7,^{1}H_{10})$	1.0054
326		$^{3}J(^{1}H_8,^{1}H_{10})$	5.378
327		$^{5}J(^{1}H,^{1}H)$	1.2683
328		$^{4}J(^{1}H_{10},^{1}H_{11})$	-0.9616
329		$^{2}J(^{15}N,^{13}C)$	-4.36832
330		$^{1}J(^{13}C_2,^{13}C_3)$	55.3033
331		$^{1}J(^{15}N,^{13}C)$	-16.628
332		$^{1}J(^{13}C_5,^{13}C_3)$	70.9736
333		$^{2}J(^{13}C_2,^{13}C_5)$	0.7469
334		$^{2}J(^{13}C_4,^{13}C_5)$	7.6265
335		$^{3}J(^{15}N,^{1}H)$	-5.44171
336		$^{1}J(^{13}C_3,^{1}H_7)$	162.7033
337		$^{2}J(^{13}C_2,^{1}H_7)$	2.6629
338		$^{2}J(^{13}C_5,^{1}H_7)$	6.0647
339		$^{3}J(^{13}C_4,^{1}H_7)$	7.3289
340	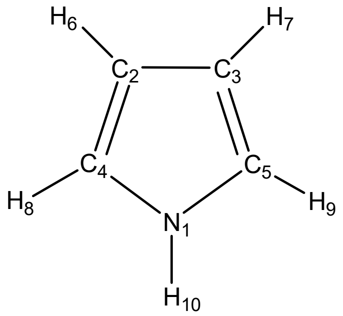	$^{3}J(^{1}H_6,^{1}H_7)$	3.4324
341		$^{2}J(^{15}N,^{1}H)$	-3.7455
342		$^{2}J(^{13}C_3,^{1}H_9)$	5.2908
343		$^{3}J(^{13}C_2,^{1}H_9)$	7.7474
344	1*H*-pyrrole (**38**)	$^{1}J(^{13}C_5,^{1}H_9)$	174.9338
345		$^{3}J(^{13}C_4,^{1}H_9)$	5.7318
346		$^{3}J(^{1}H_7,^{1}H_9)$	3.4876
347		$^{4}J(^{1}H_6,^{1}H_9)$	0.5862
348		$^{4}J(^{1}H_8,^{1}H_9)$	1.8461
349		$^{1}J(^{15}N,^{1}H)$	-95.1841
350		$^{3}J(^{13}C_3,^{1}H_{10})$	7.0071
351		$^{2}J(^{13}C_5,^{1}H_{10})$	3.1759

352		$^{4}J(^{1}H_{7},^{1}H_{10})$	2.1366
353		$^{3}J(^{1}H_{9},^{1}H_{10})$	2.5983
354		$^{1}J(^{13}C_{2},^{13}C_{1})$	73.6164
355		$^{2}J(^{13}C,^{13}C)$	-0.3526
356		$^{1}J(^{13}C_{2},^{13}C_{3})$	44.1686
357		$^{1}J(^{13}C_{1},^{1}H_{4})$	152.2812
358		$^{2}J(^{13}C_{2},^{1}H_{4})$	-2.9061
359		$^{3}J(^{13}C_{3},^{1}H_{4})$	11.9036
360		$^{1}J(^{13}C_{1},^{1}H_{5})$	148.8582
361		$^{2}J(^{13}C_{2},^{1}H_{5})$	-4.2234
362		$^{3}J(^{13}C_{3},^{1}H_{5})$	7.6946
363		$^{2}J(^{1}H_{4},^{1}H_{5})$	-1.2764
364		$^{2}J(^{13}C_{1},^{1}H_{6})$	-1.6042
365		$^{1}J(^{13}C_{2},^{1}H_{6})$	145.7659
366		$^{2}J(^{13}C_{3},^{1}H_{6})$	3.6223
367		$^{3}J(^{1}H_{4},^{1}H_{6})$	10.7156
368	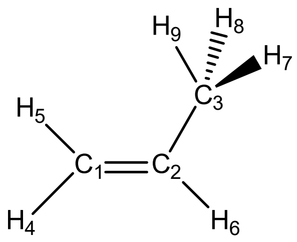	$^{3}J(^{1}H_{5},^{1}H_{6})$	16.1248
369		$^{3}J(^{13}C_{1},^{1}H_{7})$	6.2769
370		$^{2}J(^{13}C_{2},^{1}H_{7})$	-8.7433
371	prop-1-ene (**39**)	$^{1}J(^{13}C_{3},^{1}H_{7})$	120.1865
372		$^{4}J(^{1}H_{4},^{1}H_{7})$	-3.0273
373		$^{4}J(^{1}H_{5},^{1}H_{7})$	-3.2741
374		$^{3}J(^{1}H_{6},^{1}H_{7})$	3.9706
375		$^{2}J(^{1}H_{7},^{1}H_{8})$	-19.6038
376		$^{3}J(^{13}C_{1},^{1}H_{9})$	6.3047
377		$^{2}J(^{13}C_{2},^{1}H_{9})$	-6.3822
378		$^{1}J(^{13}C_{3},^{1}H_{9})$	121.4761
379		$^{4}J(^{1}H_{4},^{1}H_{9})$	-0.5624
380		$^{4}J(^{1}H_{5},^{1}H_{9})$	-0.5096
381		$^{3}J(^{1}H_{6},^{1}H_{9})$	11.714
382		$^{2}J(^{1}H_{7},^{1}H_{9})$	-14.7596
383		$^{1}J(^{13}C_{1},^{13}C_{2})$	53.2837
384		$^{1}J(^{13}C_{1},^{13}C_{3})$	73.6737
385		$^{2}J(^{13}C_{2},^{13}C_{3})$	-0.1439
386		$^{2}J(^{13}C_{3},^{13}C_{4})$	4.0504
387		$^{1}J(^{13}C_{1},^{1}H_{6})$	166.5859
388		$^{2}J(^{13}C_{2},^{1}H_{6})$	2.5841
389	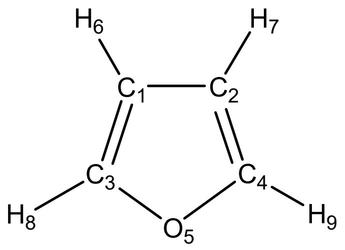	$^{2}J(^{13}C_{3},^{1}H_{6})$	7.6931
390		$^{3}J(^{13}C_{4},^{1}H_{6})$	6.7817
391		$^{3}J(^{1}H_{6},^{1}H_{7})$	3.0242
392		$^{2}J(^{13}C_{1},^{1}H_{8})$	11.0587
393	furan (**40**)	$^{3}J(^{13}C_{2},^{1}H_{8})$	6.3429
394		$^{1}J(^{13}C_{3},^{1}H_{8})$	191.8834
395		$^{3}J(^{13}C_{4},^{1}H_{8})$	6.5293
396		$^{3}J(^{1}H_{6},^{1}H_{8})$	2.7315

397		$^{4}J(^{1}H_{7},^{1}H_{8})$	-0.018
398		$^{4}J(^{1}H_{8},^{1}H_{9})$	1.1771
399		$^{1}J(^{19}F,^{13}C)$	-237.939
400		$^{2}J(^{19}F,^{13}C)$	21.7392
401		$^{1}J(^{13}C_{2},^{13}C_{3})$	73.1863
402		$^{2}J(^{13}C_{3},^{13}C_{4})$	2.4207
403		$^{3}J(^{19}F,^{13}C)$	5.1982
404		$^{2}J(^{13}C_{2},^{13}C_{6})$	-0.9948
405		$^{1}J(^{13}C_{3},^{13}C_{6})$	60.0641
406		$^{3}J(^{13}C_{4},^{13}C_{6})$	7.9857
407		$^{2}J(^{13}C_{5},^{13}C_{6})$	-2.8259
408		$^{4}J(^{19}F,^{13}C)$	5.367
409		$^{3}J(^{13}C_{7},^{13}C_{2})$	11.706
410		$^{2}J(^{13}C_{3},^{13}C_{7})$	-4.2647
411		$^{1}J(^{13}C_{6},^{13}C_{7})$	59.4301
412		$^{3}J(^{19}F,^{1}H)$	6.4563
413	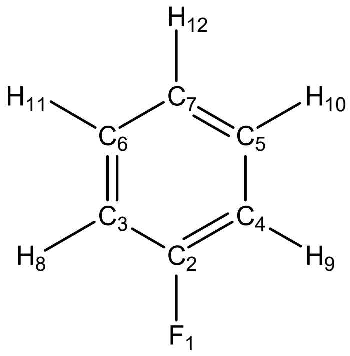 1-fluorobenzene (**41**)	$^{2}J(^{13}C_{2},^{1}H_{8})$	-6.945
414		$^{1}J(^{13}C_{3},^{1}H_{8})$	156.1754
415		$^{3}J(^{13}C_{4},^{1}H_{8})$	4.586
416		$^{2}J(^{13}C_{6},^{1}H_{8})$	-2.1031
417		$^{4}J(^{13}C_{5},^{1}H_{8})$	-1.4657
418		$^{3}J(^{13}C_{7},^{1}H_{8})$	7.9477
419		$^{4}J(^{1}H_{8},^{1}H_{9})$	1.7064
420		$^{4}J(^{19}F,^{1}H)$	4.8671
421		$^{3}J(^{13}C_{2},^{1}H_{11})$	10.858
422		$^{2}J(^{13}C_{3},^{1}H_{11})$	-0.9268
423		$^{4}J(^{13}C_{4},^{1}H_{11})$	-2.1383
424		$^{1}J(^{13}C_{6},^{1}H_{11})$	152.949
425		$^{3}J(^{13}C_{5},^{1}H_{11})$	9.1072
426		$^{2}J(^{13}C_{7},^{1}H_{11})$	-1.2265
427		$^{3}J(^{1}H_{8},^{1}H_{11})$	8.634
428		$^{5}J(^{1}H_{9},^{1}H_{11})$	0.8705
429		$^{4}J(^{1}H_{10},^{1}H_{11})$	0.8377
430		$^{5}J(^{19}F,^{1}H)$	-1.4528
431		$^{4}J(^{13}C_{2},^{1}H_{12})$	-2.5119
432		$^{3}J(^{13}C_{3},^{1}H_{12})$	8.3315
433		$^{2}J(^{13}C_{6},^{1}H_{12})$	-0.3539
434		$^{1}J(^{13}C_{7},^{1}H_{12})$	154.5041
435		$^{4}J(^{1}H_{8},^{1}H_{12})$	0.2483
436		$^{3}\mathrm{J}(^{1}H_{11},^{1}H_{12})$	7.8924

Table S5. Symmetry independent values of SSCC (in Hz) in molecules of set 1 calculated at the SOPPA(CCSD) level with the pecJ-2-old basis set.

#	Molecule	Type of SSCC [1]	SSCC value
1	$H_2C{=}C{=}CH_2$ propa-1,2-diene (**1**)	$^{1}J(^{13}C,^{13}C)$	103.9685
2		$^{2}J(^{13}C,^{13}C)$	8.2630
3		$^{2}J(^{13}C,^{1}H)$	-5.8530
4		$^{1}J(^{13}C,^{1}H)$	165.1396
5		$^{3}J(^{13}C,^{1}H)$	7.9221
6		$^{2}J(^{1}H,^{1}H)$	-13.8874
7		$^{4}J(^{1}H,^{1}H)$	-8.7102
8	$F_2C{=}CF_2$ Perfluoroethene (**2**)	$^{1}J(^{13}C,^{13}C)$	196.9734
9		$^{1}J(^{13}C,^{19}F)$	-263.4578
10		$^{2}J(^{13}C,^{19}F)$	49.5831
11		$^{2}J(^{19}F,^{19}F)$	114.2923
12		$^{3}J_{cis}(^{19}F,^{19}F)$	79.6225
13		$^{3}J_{trans}(^{19}F,^{19}F)$	-116.9129
14	$HC{\equiv}CH$ ethyne (**3**)	$^{1}J(^{13}C,^{13}C)$	190.3232
15		$^{1}J(^{13}C,^{1}H)$	248.9984
16		$^{2}J(^{13}C,^{1}H)$	51.2978
17		$^{3}J(^{1}H,^{1}H)$	10.8961
18	$H_2C{=}CH_2$ ethene (**4**)	$^{1}J(^{13}C,^{13}C)$	71.4680
19		$^{1}J(^{13}C,^{1}H)$	152.7677
20		$^{2}J(^{13}C,^{1}H)$	-3.2551
21		$^{3}J_{cis}(^{1}H,^{1}H)$	12.1374
22		$^{2}J(^{1}H,^{1}H)$	0.1449
23		$^{3}J_{trans}(^{1}H,^{1}H)$	18.3179
24	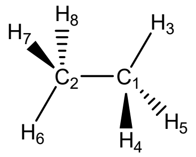 ethane (**5**)	$^{1}J(^{13}C,^{13}C)$	35.1450
25		$^{1}J(^{13}C,^{1}H)$	120.9616
26		$^{2}J(^{13}C,^{1}H)$	-4.9840
27		$^{3}J_{trans}(^{1}H_3,^{1}H_6)$	15.6623
28		$^{2}J(^{1}H,^{1}H)$	-14.0644
29		$^{3}J_{gauche}(^{1}H_4,^{1}H_6)$	3.7417
30	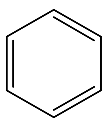 benzene (**6**)	$^{3}J(^{13}C,^{13}C)$	11.1020
31		$^{1}J(^{13}C,^{13}C)$	59.4015
32		$^{2}J(^{13}C,^{13}C)$	-3.5071
33		$^{1}J(^{13}C,^{1}H)$	153.7687
34		$^{4}J(^{13}C,^{1}H)$	-1.7404
35		$^{2}J(^{13}C,^{1}H)$	-0.1627
36		$^{3}J(^{13}C,^{1}H)$	7.7598
37		$^{3}J(^{1}H,^{1}H)$	8.0023
38		$^{4}J(^{1}H,^{1}H)$	0.7798
39		$^{5}J(^{1}H,^{1}H)$	1.1173
40	CF_4 perfluoromethane (**7**)	$^{1}J(^{19}F,^{13}C)$	-252.8380
41		$^{2}J(^{19}F,^{19}F)$	31.8737

42		$^{1}J(^{13}C,^{13}C)$	88.1765
43		$^{2}J(^{19}F,^{13}C)$	11.8545
44		$^{1}J(^{19}F,^{13}C)$	-255.9390
45		$^{1}J(^{13}C_1,^{1}H_4)$	157.5087
46		$^{2}J(^{13}C_2,^{1}H_4)$	6.6107
47		$^{3}J_{trans}(^{19}F_3,^{1}H_4)$	39.4172
48		$^{1}J(^{13}C_1,^{1}H_5)$	155.8763
49	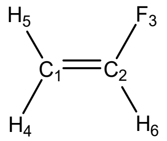	$^{2}J(^{13}C_2,^{1}H_5)$	-10.0118
50		$^{3}J_{cis}(^{19}F_3,^{1}H_5)$	12.9380
51	fluoroethene (**8**)	$^{2}J(^{1}H,^{1}H)$	-4.6593
52		$^{2}J(^{13}C_1,^{1}H_6)$	12.8746
53		$^{1}J(^{13}C_2,^{1}H_6)$	191.0820
54		$^{2}J(^{19}F,^{1}H)$	81.2749
55		$^{3}J_{cis}(^{1}H_6,^{1}H_4)$	5.9507
56		$^{3}J_{trans}(^{1}H_6,^{1}H_5)$	12.7674
57		$^{1}J(^{19}F,^{13}C)$	-222.7948
58		$^{2}J(^{19}F,^{19}F)$	316.2622
59	CH_2F_2 difluoromethane (**9**)	$^{1}J(^{13}C,^{1}H)$	173.1049
60		$^{2}J(^{19}F,^{1}H)$	49.8318
61		$^{2}J(^{1}H,^{1}H)$	0.8458
62		$^{1}J(^{15}N,^{13}C)$	-4.0795
63		$^{1}J(^{13}C_1,^{1}H_3)$	169.9340
64		$^{2}J(^{15}N_2,^{1}H_3)$	-9.3080
65		$^{1}J(^{13}C_1,^{1}H_4)$	154.3657
66	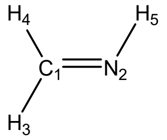	$^{2}J(^{15}N_2,^{1}H_4)$	3.8542
67		$^{2}J(^{1}H,^{1}H)$	16.4618
68	methanimine (**10**)	$^{2}J(^{13}C,^{1}H)$	-13.5455
69		$^{1}J(^{15}N,^{1}H)$	-50.1746
70		$^{3}J_{trans}(^{1}H_5,^{1}H_3)$	24.3741
71		$^{3}J_{cis}(^{1}H_5,^{1}H_4)$	18.1734
72		$^{1}J(^{13}C_1,^{13}C_2)$	191.0651
73		$^{2}J(^{13}C,^{13}C)$	12.9786
74		$^{1}J(^{13}C_2,^{13}C_3)$	70.8411
75		$^{1}J(^{13}C_1,^{1}H_4)$	247.8393
76		$^{2}J(^{13}C_2,^{1}H_4)$	51.1922
77	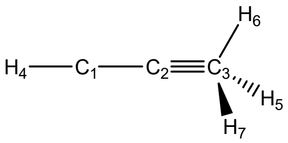	$^{3}J(^{13}C_3,^{1}H_4)$	4.1792
78		$^{3}J(^{13}C_1,^{1}H_6)$	3.7704
79	prop-1-yne (**11**)	$^{2}J(^{13}C_2,^{1}H_6)$	-11.7429
80		$^{1}J(^{13}C_3,^{1}H_6)$	127.1220
81		$^{4}J(^{1}H,^{1}H)$	-3.6647
82		$^{2}J(^{1}H,^{1}H)$	-17.3144
83		$^{1}J(^{13}C,^{13}C)$	42.0580
84		$^{1}J(^{13}C_1,^{1}H_4)$	163.9921
85		$^{2}J(^{13}C_2,^{1}H_4)$	26.5039
86		$^{2}J(^{13}C_1,^{1}H_5)$	-8.3197

87	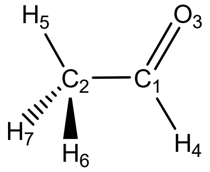<br>acetaldehyde (**12**)	$^{1}J(^{13}C_{2},^{1}H_{5})$	130.2795
88		$^{3}J_{trans}(^{1}H_{5},^{1}H_{4})$	8.1114
89		$^{2}J(^{13}C_{1},^{1}H_{6})$	-6.4244
90		$^{1}J(^{13}C_{2},^{1}H_{6})$	119.1103
91		$^{3}J_{gauche}(^{1}H_{6},^{1}H_{4})$	0.3313
92		$^{2}J(^{1}H_{6},^{1}H_{5})$	-13.3741
93		$^{2}J(^{1}H_{6},^{1}H_{7})$	-20.0224
94	N≡C−CH_3<br>acetonitrile (**13**)	$^{1}J(^{13}C,^{13}C)$	62.4814
95		$^{2}J(^{15}N,^{13}C)$	2.7317
96		$^{1}J(^{15}N,^{13}C)$	-17.8599
97		$^{1}J(^{13}C,^{1}H)$	130.3962
98		$^{2}J(^{13}C,^{1}H)$	-10.9447
99		$^{3}J(^{15}N,^{1}H)$	-1.5214
100		$^{2}J(^{1}H,^{1}H)$	-17.3744
101	H_3C−F<br>fluoromethane (**14**)	$^{1}J(^{19}F,^{13}C)$	-159.5839
102		$^{1}J(^{13}C,^{1}H)$	142.2859
103		$^{2}J(^{19}F,^{1}H)$	48.5904
104		$^{2}J(^{1}H,^{1}H)$	-10.8588
105	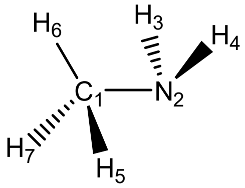<br>methanamine (**15**)	$^{1}J(^{15}N,^{13}C)$	-5.8519
106		$^{2}J(^{13}C,^{1}H)$	-3.6575
107		$^{1}J(^{15}N,^{1}H)$	-64.6582
108		$^{2}J(^{1}H_{3},^{1}H_{4})$	-10.8725
109		$^{1}J(^{13}C_{1},^{1}H_{5})$	128.5070
110		$^{2}J(^{15}N_{2},^{1}H_{5})$	-1.4865
111		$^{3}J(^{1}H_{5},^{1}H_{4})$	2.7147
112		$^{3}J(^{1}H_{5},^{1}H_{3})$	15.5091
113		$^{2}J(^{1}H_{7},^{1}H_{5})$	-15.6777
114		$^{1}J(^{13}C_{1},^{1}H_{6})$	125.5935
115		$^{2}J(^{15}N_{2},^{1}H_{6})$	0.9762
116		$^{3}J(^{1}H_{6},^{1}H_{4})$	2.1576
117		$^{2}J(^{1}H_{6},^{1}H_{5})$	-11.5300
118	CH_4<br>methane (**16**)	$^{1}J(^{13}C,^{1}H)$	121.1305
119		$^{2}J(^{1}H,^{1}H)$	-13.8486
120	CHF_3<br>fluoroform (**17**)	$^{1}J(^{13}C,^{1}H)$	222.1448
121		$^{1}J(^{19}F,^{13}C)$	-257.5743
122		$^{2}J(^{19}F,^{1}H)$	73.7973
123		$^{2}J(^{19}F,^{19}F)$	124.6838
124	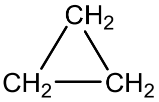<br>cyclopropane (**18**)	$^{1}J(^{13}C,^{13}C)$	13.7051
125		$^{1}J(^{13}C,^{1}H)$	155.4642
126		$^{2}J(^{13}C,^{1}H)$	-2.9522
127		$^{2}J(^{1}H,^{1}H)$	-6.0506
128		$^{3}J_{cis}(^{1}H,^{1}H)$	9.0157
129		$^{3}J_{trans}(^{1}H,^{1}H)$	5.0759
130	F−C≡C−F<br>1,2-difluoroethyne (**19**)	$^{1}J(^{13}C,^{13}C)$	407.6446
131		$^{1}J(^{19}F,^{13}C)$	-279.4004

132		$^{2}J(^{19}F,^{13}C)$	39.0842
133		$^{3}J(^{19}F,^{19}F)$	-6.4344
134	F−C≡N<br>fluoroformonitrile (**20**)	$^{1}J(^{19}F,^{13}C)$	-402.4457
135		$^{1}J(^{15}N,^{13}C)$	-4.6619
136		$^{2}J(^{19}F,^{15}N)$	50.5626
137	$H_2C{=}CF_2$<br>1,1-difluoroethene (**21**)	$^{1}J(^{13}C,^{13}C)$	117.4825
138		$^{2}J(^{19}F,^{13}C)$	27.4732
139		$^{1}J(^{19}F,^{13}C)$	-282.5771
140		$^{2}J(^{19}F,^{19}F)$	18.2069
141		$^{1}J(^{13}C,^{1}H)$	162.6218
142		$^{2}J(^{13}C,^{1}H)$	-2.0645
143		$^{3}J_{cis}(^{19}F,^{1}H)$	-1.5745
144		$^{3}J_{trans}(^{19}F,^{1}H)$	26.7907
145		$^{2}J(^{1}H,^{1}H)$	-6.3499
146	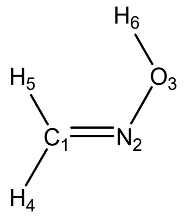<br>formaldehyde oxime (*Z*) (**22**)	$^{1}J(^{15}N,^{13}C)$	-2.9204
147		$^{1}J(^{13}C_1,^{1}H_4)$	182.2179
148		$^{2}J(^{15}N_2,^{1}H_4)$	-11.9863
149		$^{1}J(^{13}C_1,^{1}H_5)$	152.0596
150		$^{2}J(^{15}N_2,^{1}H_5)$	2.9298
151		$^{2}J(^{1}H,^{1}H)$	6.3657
152		$^{3}J(^{13}C,^{1}H)$	4.1101
153		$^{2}J(^{15}N_2,^{1}H_6)$	1.3963
154		$^{4}J(^{1}H_6,^{1}H_4)$	-1.3510
155		$^{4}J(^{1}H_6,^{1}H_5)$	1.3625
156	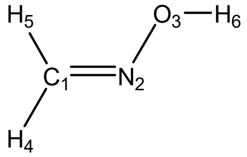<br>formaldehyde oxime (*E*) (**23**)	$^{1}J(^{15}N,^{13}C)$	-5.9917
157		$^{1}J(^{13}C_1,^{1}H_4)$	175.4718
158		$^{2}J(^{15}N_2,^{1}H_4)$	-12.7808
159		$^{1}J(^{13}C_1,^{1}H_5)$	161.3006
160		$^{2}J(^{15}N_2,^{1}H_5)$	2.9350
161		$^{2}J(^{1}H,^{1}H)$	8.0440
162		$^{3}J(^{13}C,^{1}H)$	10.7681
163		$^{2}J(^{15}N_2,^{1}H_6)$	-1.9242
164		$^{4}J(^{1}H_6,^{1}H_4)$	1.1633
165		$^{4}J(^{1}H_6,^{1}H_5)$	-0.8353
166	H−C≡C−F<br>fluoroethyne (**24**)	$^{1}J(^{13}C,^{13}C)$	274.3462
167		$^{2}J(^{19}F,^{13}C)$	23.0367
168		$^{1}J(^{19}F,^{13}C)$	-292.2140
169		$^{1}J(^{13}C,^{1}H)$	277.3661
170		$^{2}J(^{13}C,^{1}H)$	65.7896
171		$^{3}J(^{19}F,^{1}H)$	11.2728
172	H−C≡N<br>hydrogen cyanide (**25**)	$^{1}J(^{15}N,^{13}C)$	-18.4858
173		$^{1}J(^{13}C,^{1}H)$	260.1216
174		$^{2}J(^{15}N,^{1}H)$	-8.3297
175		$^{1}J(^{13}C,^{13}C)$	145.2693
176		$^{2}J(^{19}F_3,^{13}C_1)$	64.3672

177		$^{1}J(^{19}F_3,^{13}C_2)$	-269.2743
178		$^{1}J(^{19}F_4,^{13}C_1)$	-229.8882
179		$^{2}J(^{19}F_4,^{13}C_2)$	36.3480
180		$^{3}J_{trans}(^{19}F_3,^{19}F_4)$	-123.6974
181		$^{2}J(^{19}F_5,^{13}C_1)$	19.9573
182	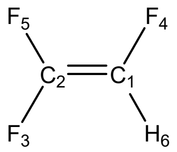	$^{1}J(^{19}F_5,^{13}C_2)$	-281.4419
183		$^{2}J(^{19}F,^{19}F)$	69.7083
184		$^{3}J_{cis}(^{19}F_4,^{19}F_5)$	40.9330
185	1,1,2-trifluoroethene (**26**)	$^{1}J(^{13}C,^{1}H)$	202.0137
186		$^{2}J(^{13}C,^{1}H)$	14.1691
187		$^{3}J_{cis}(^{19}F_3,^{1}H_6)$	-5.4256
188		$^{2}J(^{19}F,^{1}H)$	71.9936
189		$^{3}J_{trans}(^{19}F_5,^{1}H_6)$	7.8584
190		$^{1}J(^{13}C,^{13}C)$	101.8006
191		$^{1}J(^{19}F,^{13}C)$	-249.3040
192		$^{2}J(^{19}F,^{13}C)$	9.5158
193	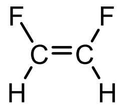	$^{3}J(^{19}F,^{19}F)$	-10.9982
194		$^{1}J(^{13}C,^{1}H)$	194.9690
195	(*Z*)-1,2-difluoroethene (**27**)	$^{2}J(^{13}C,^{1}H)$	22.6226
196		$^{2}J(^{19}F,^{1}H)$	72.0037
197		$^{3}J(^{19}F,^{1}H)$	13.4784
198		$^{3}J(^{1}H,^{1}H)$	3.4663
199		$^{1}J(^{13}C,^{13}C)$	115.3613
200		$^{1}J(^{19}F,^{13}C)$	-237.4103
201		$^{2}J(^{19}F,^{13}C)$	49.0835
202	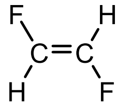	$^{3}J(^{19}F,^{19}F)$	-137.4823
203		$^{2}J(^{13}C,^{1}H)$	4.4366
204	(*E*)-1,2-difluoroethene (**28**)	$^{1}J(^{13}C,^{1}H)$	193.7091
205		$^{3}J(^{19}F,^{1}H)$	0.3977
206		$^{2}J(^{19}F,^{1}H)$	75.3951
207		$^{3}J(^{1}H,^{1}H)$	9.9621
208	HF<br>hydrogen fluoride (**29**)	$^{1}J(^{19}F,^{1}H)$	533.4235
209		$^{1}J(^{15}N,^{15}N)$	-20.5298
210	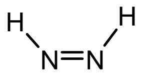	$^{1}J(^{15}N,^{1}H)$	-35.7527
211	diazene (*Z*) (**31**)	$^{2}J(^{15}N,^{1}H)$	1.4075
212		$^{3}J(^{1}H,^{1}H)$	38.0140
213		$^{1}J(^{15}N,^{15}N)$	-20.8699
214	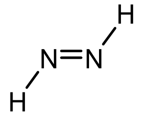	$^{2}J(^{15}N,^{1}H)$	-0.1791
215		$^{1}J(^{15}N,^{1}H)$	-45.6520
216	diazene (*E*) (**30**)	$^{3}J(^{1}H,^{1}H)$	37.4189
217		$^{1}J(^{15}N,^{15}N)$	1.0211
218		$^{1}J(^{15}N,^{1}H)$	-58.6910
219		$^{2}J(^{15}N,^{1}H)$	-1.6332
220		$^{2}J(^{1}H,^{1}H)$	-15.4268

221	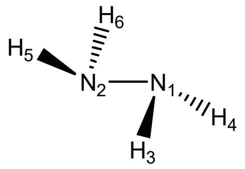	${}^{3}J({}^{1}H_5,{}^{1}H_3)$	1.5231
222	hydrazine (**32**)	${}^{3}J({}^{1}H_4,{}^{1}H_5)$	13.3160
223	N_2 molecular nitrogen (**33**)	${}^{1}J({}^{15}N,{}^{15}N)$	-2.786296118
224	NH_3	${}^{1}J({}^{15}N,{}^{1}H)$	-61.22158444
225	ammonia (**34**)	${}^{2}J({}^{1}H,{}^{1}H)$	-11.3909
226	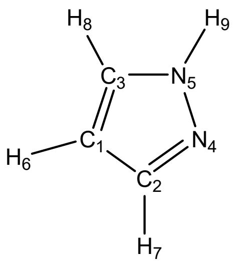 1*H*-pyrazole (**35**)	${}^{1}J({}^{13}C_1,{}^{13}C_2)$	54.9937
227		${}^{1}J({}^{13}C_3,{}^{13}C_1)$	69.7961
228		${}^{2}J({}^{13}C_3,{}^{13}C_2)$	1.7306
229		${}^{2}J({}^{15}N_4,{}^{13}C_1)$	2.897531948
230		${}^{1}J({}^{15}N_4,{}^{13}C_2)$	-2.278215355
231		${}^{2}J({}^{15}N_4,{}^{13}C_3)$	0.919366014
232		${}^{2}J({}^{15}N_5,{}^{13}C_1)$	-5.72576777
233		${}^{2}J({}^{15}N_5,{}^{13}C_2)$	-0.778389077
234		${}^{1}J({}^{15}N_5,{}^{13}C_3)$	-15.5370612
235		${}^{1}J({}^{15}N,{}^{15}N)$	-11.73609176
236		${}^{1}J({}^{13}C_1,{}^{1}H_6)$	170.6495
237		${}^{2}J({}^{13}C_2,{}^{1}H_6)$	5.0887
238		${}^{2}J({}^{13}C3,{}^{1}H_6)$	7.5301
239		${}^{3}J({}^{15}N_4,{}^{1}H_6)$	-1.102986721
240		${}^{3}J({}^{15}N_5,{}^{1}H_6)$	-5.740075877
241		${}^{2}J({}^{13}C_1,{}^{1}H_7)$	10.4566
242		${}^{1}J({}^{13}C_2,{}^{1}H_7)$	180.108
243		${}^{3}J({}^{13}C_3,{}^{1}H_7)$	4.7215
244		${}^{2}J({}^{15}N_4,{}^{1}H_7)$	-11.812184
245		${}^{3}J({}^{15}N_5,{}^{1}H_7)$	-8.753335161
246		${}^{3}J({}^{1}H_7,{}^{1}H_6)$	1.8358
247		${}^{2}J({}^{13}C_1,{}^{1}H_8)$	6.7839
248		${}^{3}J({}^{13}C_2,{}^{1}H_8)$	7.8864
249		${}^{1}J({}^{13}C_3,{}^{1}H_8)$	178.5961
250		${}^{3}J({}^{15}N_4,{}^{1}H_8)$	0.286863518
251		${}^{2}J({}^{15}N_5,{}^{1}H_8)$	-4.45753644
252		${}^{3}J({}^{1}H_8,{}^{1}H_6)$	3.0069
253		${}^{4}J({}^{1}H_8,{}^{1}H_7)$	0.1338
254		${}^{3}J({}^{13}C_1,{}^{1}H_9)$	5.4377
255		${}^{3}J({}^{13}C_2,{}^{1}H_9)$	10.2097
256		${}^{2}J({}^{13}C_3,{}^{1}H_9)$	9.0541
257		${}^{2}J({}^{15}N_4,{}^{1}H_9)$	-8.546709262
258		${}^{1}J({}^{15}N,{}^{1}H)$	-106.1270172
259		${}^{4}J({}^{1}H_9,{}^{1}H_6)$	1.9657
260		${}^{4}J({}^{1}H_9,{}^{1}H_7)$	1.8561
261		${}^{3}J({}^{1}H_9,{}^{1}H_8)$	1.678

262		$^{1}J(^{13}C,^{13}C)$	71.6295
263		$^{2}J(^{13}C_1,^{13}C_3)$	-4.3669
264		$^{2}J(^{13}C_3,^{13}C_2)$	9.1107
265		$^{1}J(^{15}N_4,^{13}C_1)$	1.137354233
266		$^{2}J(^{15}N_4,^{13}C_2)$	2.456645866
267		$^{1}J(^{15}N_4,^{13}C_3)$	-2.709562699
268		$^{2}J(^{15}N_5,^{13}C_1)$	-6.248855329
269		$^{1}J(^{15}N_5,^{13}C_2)$	-17.00785043
270		$^{1}J(^{15}N_5,^{13}C_3)$	-13.31958516
271		$^{2}J(^{15}N,^{15}N)$	-1.515540445
272		$^{1}J(^{13}C_1,^{1}H_6)$	183.8456
273		$^{2}J(^{13}C_2,^{1}H_6)$	15.3446
274		$^{3}J(^{13}C_3,^{1}H_6)$	11.4808
275		$^{2}J(^{15}N_4,^{1}H_6)$	-9.693181406
276		$^{3}J(^{15}N_5,^{1}H_6)$	-3.552338258
277	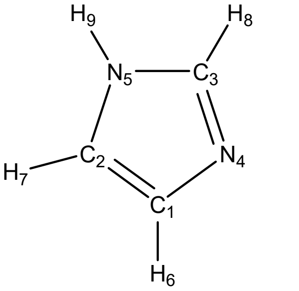	$^{2}J(^{13}C_1,^{1}H_7)$	8.0755
278		$^{1}J(^{13}C_2,^{1}H_7)$	182.2845
279		$^{3}J(^{13}C_3,^{1}H_7)$	6.7453
280		$^{3}J(^{15}N_4,^{1}H_7)$	-1.121362819
281		$^{2}J(^{15}N_5,^{1}H_7)$	-4.578313696
282		$^{3}J(^{1}H_7,^{1}H_6)$	2.0986
283	1*H*-imidazole (**36**)	$^{3}J(^{13}C_1,^{1}H_8)$	11.3185
284		$^{3}J(C_2,^{1}H_8)$	3.2918
285		$^{1}J(C_3,^{1}H_8)$	199.8653
286		$^{2}J(^{15}N_4,^{1}H_8)$	-11.06507637
287		$^{2}J(^{15}N_5,^{1}H_8)$	-8.808042629
288		$^{4}J(^{1}H_8,^{1}H_6)$	0.253
289		$^{4}J(^{1}H8,^{1}H_7)$	1.3426
290		$^{3}J(^{13}C_1,^{1}H_9)$	7.81
291		$^{2}J(^{13}C_2,^{1}H_9)$	4.3989
292		$^{2}J(^{13}C_3,^{1}H_9)$	4.5825
293		$^{3}J(^{15}N_4,^{1}H_9)$	0.121899461
294		$^{1}J(^{15}N,^{1}H)$	-95.95563645
295		$^{4}J(^{1}H_9,^{1}H_6)$	1.7029
296		$^{3}J(^{1}H_9,^{1}H_7)$	2.0797
297		$^{3}J(^{1}H_9,^{1}H_8)$	0.9921
298		$^{1}J(^{13}C_1,^{13}C_2)$	57.3784
299		$^{2}J(^{13}C_2,^{13}C_3)$	-4.1349
300		$^{2}J(^{13}C_1,^{13}C_4)$	-3.7181
301	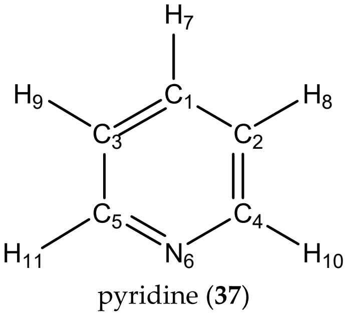	$^{1}J(^{13}C_2,^{13}C_4)$	58.0494
302		$^{3}J(^{13}C_3,^{13}C_4)$	15.1941
303		$^{2}J(^{13}C_5,^{13}C_4)$	-7.249
304		$^{3}J(^{15}N_6,^{13}C_1)$	-4.55166134
305	pyridine (**37**)	$^{2}J(^{15}N_6,^{13}C_2)$	2.950135283
306		$^{1}J(^{15}N,^{13}C)$	-1.83676817

307		$^{1}J(^{13}C_1,^{1}H_7)$	154.7762
308		$^{2}J(^{13}C_2,^{1}H_7)$	-0.4375
309		$^{3}J(^{13}C_4,^{1}H_7)$	6.8361
310		$^{4}J(^{15}N,^{1}H)$	0.522947284
311		$^{2}J(^{13}C_1,^{1}H_8)$	-0.3871
312		$^{1}J(^{13}C_2,^{1}H_8)$	157.3695
313		$^{3}J(^{13}C_3,^{1}H_8)$	6.6803
314		$^{2}J(^{13}C_4,^{1}H_8)$	1.8322
315		$^{4}J(^{13}C_5,^{1}H_8)$	-1.313
316		$^{3}J(^{15}N,^{1}H)$	-1.618218849
317		$^{3}J(^{1}H_7,^{1}H_8)$	7.99
318		$^{4}J(^{1}H_8,^{1}H_9)$	0.7773
319		$^{3}J(^{13}C_1,^{1}H_{10})$	6.6617
320		$^{2}J(^{13}C_2,^{1}H_{10})$	7.3565
321		$^{4}J(^{13}C_3,^{1}H_{10})$	-2.1072
322		$^{1}J(^{13}C_4,^{1}H_{10})$	171.8174
323		$^{3}J(^{13}C_5,^{1}H_{10})$	11.3168
324		$^{2}J(^{15}N,^{1}H)$	-9.8108726
325		$^{4}J(^{1}H_7,^{1}H_{10})$	1.3065
326		$^{3}J(^{1}H_8,^{1}H_{10})$	5.4024
327		$^{5}J(^{1}H,^{1}H)$	1.356
328		$^{4}J(^{1}H_{10},^{1}H_{11})$	-0.6919
329		$^{2}J(^{15}N,^{13}C)$	-4.394973541
330		$^{1}J(^{13}C_2,^{13}C_3)$	55.3168
331		$^{1}J(^{15}N,^{13}C)$	-16.13168929
332		$^{1}J(^{13}C_5,^{13}C_3)$	71.3031
333		$^{2}J(^{13}C_2,^{13}C_5)$	1.0442
334		$^{2}J(^{13}C_4,^{13}C_5)$	7.5966
335		$^{3}J(^{15}N,^{1}H)$	-5.462470545
336	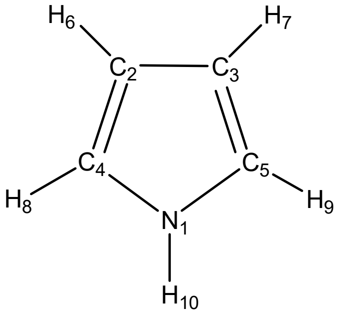	$^{1}J(^{13}C_3,^{1}H_7)$	165.4559
337		$^{2}J(^{13}C_2,^{1}H_7)$	3.2865
338		$^{2}J(^{13}C_5,^{1}H_7)$	6.894
339		$^{3}J(^{13}C_4,^{1}H_7)$	7.3304
340		$^{3}J(^{1}H_6,^{1}H_7)$	3.5811
341		$^{2}J(^{15}N,^{1}H)$	-3.964047023
342		$^{2}J(^{13}C_3,^{1}H_9)$	6.1413
343		$^{3}J(^{13}C_2,^{1}H_9)$	7.6363
344	1*H*-pyrrole (**38**)	$^{1}J(^{13}C_5,^{1}H_9)$	177.7432
345		$^{3}J(^{13}C_4,^{1}H_9)$	5.9218
346		$^{3}J(^{1}H_7,^{1}H_9)$	3.5956
347		$^{4}J(^{1}H_6,^{1}H_9)$	0.9524
348		$^{4}J(^{1}H_8,^{1}H_9)$	2.1135
349		$^{1}J(^{15}N,^{1}H)$	-95.79824727
350		$^{3}J(^{13}C_3,^{1}H_{10})$	7.0636
351		$^{2}J(^{13}C_5,^{1}H_{10})$	3.5508

352		$^{4}J(^{1}H_{7},^{1}H_{10})$	2.4629
353		$^{3}J(^{1}H_{9},^{1}H_{10})$	2.7723
354		$^{1}J(^{13}C_{2},^{13}C_{1})$	73.9311
355		$^{2}J(^{13}C,^{13}C)$	-0.0802
356		$^{1}J(^{13}C_{2},^{13}C_{3})$	44.053
357		$^{1}J(^{13}C_{1},^{1}H_{4})$	153.8169
358		$^{2}J(^{13}C_{2},^{1}H_{4})$	-2.052
359		$^{3}J(^{13}C_{3},^{1}H_{4})$	11.8996
360		$^{1}J(^{13}C_{1},^{1}H_{5})$	150.1173
361		$^{2}J(^{13}C_{2},^{1}H_{5})$	-3.5428
362		$^{3}J(^{13}C_{3},^{1}H_{5})$	7.6688
363		$^{2}J(^{1}H_{4},^{1}H_{5})$	-0.0479
364		$^{2}J(^{13}C_{1},^{1}H_{6})$	-0.8864
365		$^{1}J(^{13}C_{2},^{1}H_{6})$	147.681
366		$^{2}J(^{13}C_{3},^{1}H_{6})$	3.9888
367	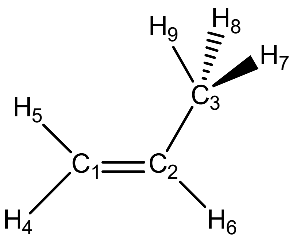	$^{3}J(^{1}H_{4},^{1}H_{6})$	10.8242
368		$^{3}J(^{1}H_{5},^{1}H_{6})$	16.3325
369		$^{3}J(^{13}C_{1},^{1}H_{7})$	6.2673
370		$^{2}J(^{13}C_{2},^{1}H_{7})$	-8.2259
371	prop-1-ene (**39**)	$^{1}J(^{13}C_{3},^{1}H_{7})$	121.576
372		$^{4}J(^{1}H_{4},^{1}H_{7})$	-2.7118
373		$^{4}J(^{1}H_{5},^{1}H_{7})$	-2.9619
374		$^{3}J(^{1}H_{6},^{1}H_{7})$	3.8851
375		$^{2}J(^{1}H_{7},^{1}H_{8})$	-19.0368
376		$^{3}J(^{13}C_{1},^{1}H_{9})$	6.3461
377		$^{2}J(^{13}C_{2},^{1}H_{9})$	-6.097
378		$^{1}J(^{13}C_{3},^{1}H_{9})$	122.7361
379		$^{4}J(^{1}H_{4},^{1}H_{9})$	-0.3665
380		$^{4}J(^{1}H_{5},^{1}H_{9})$	-0.5643
381		$^{3}J(^{1}H_{6},^{1}H_{9})$	12.0323
382		$^{2}J(^{1}H_{7},^{1}H_{9})$	-14.1003
383		$^{1}J(^{13}C_{1},^{13}C_{2})$	53.0844
384		$^{1}J(^{13}C_{1},^{13}C_{3})$	74.5108
385		$^{2}J(^{13}C_{2},^{13}C_{3})$	0.0045
386		$^{2}J(^{13}C_{3},^{13}C_{4})$	4.0926
387		$^{1}J(^{13}C_{1},^{1}H_{6})$	169.2731
388		$^{2}J(^{13}C_{2},^{1}H_{6})$	3.1233
389	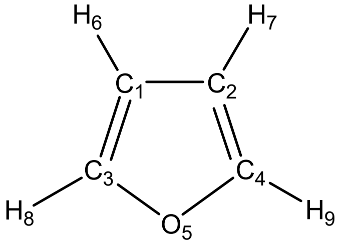	$^{2}J(^{13}C_{3},^{1}H_{6})$	8.6927
390		$^{3}J(^{13}C_{4},^{1}H_{6})$	6.775
391		$^{3}J(^{1}H_{6},^{1}H_{7})$	3.2011
392	furan (**40**)	$^{2}J(^{13}C_{1},^{1}H_{8})$	12.0195
393		$^{3}J(^{13}C_{2},^{1}H_{8})$	6.3001
394		$^{1}J(^{13}C_{3},^{1}H_{8})$	194.8705
395		$^{3}J(^{13}C_{4},^{1}H_{8})$	6.6517
396		$^{3}J(^{1}H_{6},^{1}H_{8})$	2.7697

397		$^{4}J(^{1}H_{7},^{1}H_{8})$	0.3265
398		$^{4}J(^{1}H_{8},^{1}H_{9})$	1.5615
399		$^{1}J(^{19}F,^{13}C)$	-244.4374
400		$^{2}J(^{19}F,^{13}C)$	21.6
401		$^{1}J(^{13}C_{2},^{13}C_{3})$	73.8035
402		$^{2}J(^{13}C_{3},^{13}C_{4})$	2.853
403		$^{3}J(^{19}F,^{13}C)$	5.004
404		$^{2}J(^{13}C_{2},^{13}C_{6})$	-0.6025
405		$^{1}J(^{13}C_{3},^{13}C_{6})$	60.4608
406		$^{3}J(^{13}C_{4},^{13}C_{6})$	7.8625
407		$^{2}J(^{13}C_{5},^{13}C_{6})$	-2.4791
408		$^{4}J(^{19}F,^{13}C)$	5.6373
409		$^{3}J(^{13}C_{7},^{13}C_{2})$	11.6025
410		$^{2}J(^{13}C_{3},^{13}C_{7})$	-3.9598
411		$^{1}J(^{13}C_{6},^{13}C_{7})$	59.8006
412		$^{3}J(^{19}F,^{1}H)$	6.1788
413		$^{2}J(^{13}C_{2},^{1}H_{8})$	-6.317
414		$^{1}J(^{13}C_{3},^{1}H_{8})$	157.9853
415		$^{3}J(^{13}C_{4},^{1}H_{8})$	4.4962
416		$^{2}J(^{13}C_{6},^{1}H_{8})$	-1.4985
417	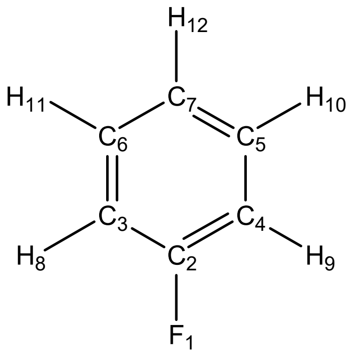	$^{4}J(^{13}C_{5},^{1}H_{8})$	-1.2659
418		$^{3}J(^{13}C_{7},^{1}H_{8})$	7.8358
419		$^{4}J(^{1}H_{8},^{1}H_{9})$	2.035
420		$^{4}J(^{19}F,^{1}H)$	4.8338
421		$^{3}J(^{13}C_{2},^{1}H_{11})$	10.6953
422	1-fluorobenzene (**41**)	$^{2}J(^{13}C_{3},^{1}H_{11})$	-0.2977
423		$^{4}J(^{13}C_{4},^{1}H_{11})$	-1.9355
424		$^{1}J(^{13}C_{6},^{1}H_{11})$	155.1138
425		$^{3}J(^{13}C_{5},^{1}H_{11})$	9.0081
426		$^{2}J(^{13}C_{7},^{1}H_{11})$	-0.5697
427		$^{3}J(^{1}H_{8},^{1}H_{11})$	8.717
428		$^{5}J(^{1}H_{9},^{1}H_{11})$	0.9351
429		$^{4}J(^{1}H_{10},^{1}H_{11})$	1.1386
430		$^{5}J(^{19}F,^{1}H)$	-1.2742
431		$^{4}J(^{13}C_{2},^{1}H_{12})$	-2.289
432		$^{3}J(^{13}C_{3},^{1}H_{12})$	8.2493
433		$^{2}J(^{13}C_{6},^{1}H_{12})$	0.3145
434		$^{1}J(^{13}C_{7},^{1}H_{12})$	156.7981
435		$^{4}J(^{1}H_{8},^{1}H_{12})$	0.5459
436		$^{3}\mathrm{J}(^{1}H_{11},^{1}H_{12})$	7.9731

Table S6. Symmetry independent values of SSCC (in Hz) in molecules of set 1 calculated at the SOPPA(CCSD) level with the pecJ-1-new(gen) basis set.

#	Molecule	Type of SSCC [1]	SSCC value
1	$H_2C{=}C{=}CH_2$ propa-1,2-diene (**1**)	$^1J(^{13}C,^{13}C)$	102.9629
2		$^2J(^{13}C,^{13}C)$	7.7582
3		$^2J(^{13}C,^{1}H)$	-6.9742
4		$^1J(^{13}C,^{1}H)$	166.7133
5		$^3J(^{13}C,^{1}H)$	8.2944
6		$^2J(^{1}H,^{1}H)$	-15.0444
7		$^4J(^{1}H,^{1}H)$	-9.8178
8	$F_2C{=}CF_2$ Perfluoroethene (**2**)	$^1J(^{13}C,^{13}C)$	193.8484
9		$^1J(^{13}C,^{19}F)$	-256.4269
10		$^2J(^{13}C,^{19}F)$	48.0723
11		$^2J(^{19}F,^{19}F)$	124.6105
12		$^3J_{cis}(^{19}F,^{19}F)$	84.4958
13		$^3J_{trans}(^{19}F,^{19}F)$	-116.5283
14	$HC{\equiv}CH$ ethyne (**3**)	$^1J(^{13}C,^{13}C)$	190.1833
15		$^1J(^{13}C,^{1}H)$	251.7990
16		$^2J(^{13}C,^{1}H)$	49.4022
17		$^3J(^{1}H,^{1}H)$	11.5353
18	$H_2C{=}CH_2$ ethene (**4**)	$^1J(^{13}C,^{13}C)$	71.1240
19		$^1J(^{13}C,^{1}H)$	153.9810
20		$^2J(^{13}C,^{1}H)$	-4.0733
21		$^3J_{cis}(^{1}H,^{1}H)$	12.4765
22		$^2J(^{1}H,^{1}H)$	-0.9651
23		$^3J_{trans}(^{1}H,^{1}H)$	18.8562
24	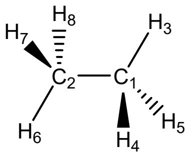 ethane (**5**)	$^1J(^{13}C,^{13}C)$	34.8029
25		$^1J(^{13}C,^{1}H)$	121.9032
26		$^2J(^{13}C,^{1}H)$	-5.4044
27		$^3J_{trans}(^{1}H_3,^{1}H_6)$	15.6939
28		$^2J(^{1}H,^{1}H)$	-15.2792
29		$^3J_{gauche}(^{1}H_4,^{1}H_6)$	3.7167
30	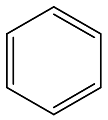 benzene (**6**)	$^3J(^{13}C,^{13}C)$	11.0585
31		$^1J(^{13}C,^{13}C)$	58.9649
32		$^2J(^{13}C,^{13}C)$	-3.8906
33		$^1J(^{13}C,^{1}H)$	154.3892
34		$^4J(^{13}C,^{1}H)$	-2.0275
35		$^2J(^{13}C,^{1}H)$	-0.8426
36		$^3J(^{13}C,^{1}H)$	8.0413
37		$^3J(^{1}H,^{1}H)$	8.1977
38		$^4J(^{1}H,^{1}H)$	0.4049
39		$^5J(^{1}H,^{1}H)$	1.0970
40	CF_4 perfluoromethane (**7**)	$^1J(^{19}F,^{13}C)$	-247.0858
41		$^2J(^{19}F,^{19}F)$	30.1196

42		$^{1}J(^{13}C,^{13}C)$	87.3961
43		$^{2}J(^{19}F, ^{13}C)$	11.6312
44		$^{1}J(^{19}F,^{13}C)$	-242.1496
45		$^{1}J(^{13}C_1,^{1}H_4)$	158.8237
46		$^{2}J(^{13}C_2,^{1}H_4)$	5.7891
47	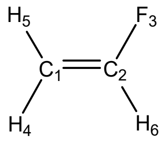	$^{3}J_{trans}(^{19}F_3,^{1}H_4)$	37.3334
48		$^{1}J(^{13}C_1,^{1}H_5)$	158.0858
49		$^{2}J(^{13}C_2,^{1}H_5)$	-11.0983
50		$^{3}J_{cis}(^{19}F_3,^{1}H_5)$	11.8701
51	fluoroethene (**8**)	$^{2}J(^{1}H,^{1}H)$	-5.8223
52		$^{2}J(^{13}C_1,^{1}H_6)$	11.9582
53		$^{1}J(^{13}C_2,^{1}H_6)$	193.2412
54		$^{2}J(^{19}F,^{1}H)$	78.4852
55		$^{3}J_{cis}(^{1}H_6,^{1}H_4)$	6.2860
56		$^{3}J_{trans}(^{1}H_6,^{1}H_5)$	13.1970
57		$^{1}J(^{19}F,^{13}C)$	-210.4535
58	CH_2F_2	$^{2}J(^{19}F,^{19}F)$	321.6382
59	difluoromethane (**9**)	$^{1}J(^{13}C,^{1}H)$	175.5227
60		$^{2}J(^{19}F,^{1}H)$	48.5611
61		$^{2}J(^{1}H,^{1}H)$	-0.3864
62		$^{1}J(^{15}N,^{13}C)$	-4.5890
63		$^{1}J(^{13}C_1,^{1}H_3)$	171.5418
64		$^{2}J(^{15}N_2,^{1}H_3)$	-10.4815
65	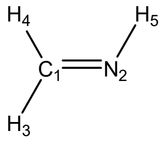	$^{1}J(^{13}C_1,^{1}H_4)$	154.4241
66		$^{2}J(^{15}N_2,^{1}H_4)$	3.9461
67		$^{2}J(^{1}H,^{1}H)$	15.7440
68	methanimine (**10**)	$^{2}J(^{13}C,^{1}H)$	-13.9379
69		$^{1}J(^{15}N,^{1}H)$	-54.0660
70		$^{3}J_{trans}(^{1}H_5,^{1}H_3)$	24.8866
71		$^{3}J_{cis}(^{1}H_5,^{1}H_4)$	18.7111
72		$^{1}J(^{13}C_1,^{13}C_2)$	190.3080
73		$^{2}J(^{13}C,^{13}C)$	12.6798
74		$^{1}J(^{13}C_2,^{13}C_3)$	70.5904
75		$^{1}J(^{13}C_1,^{1}H_4)$	250.6649
76	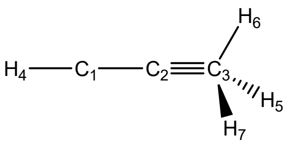	$^{2}J(^{13}C_2,^{1}H_4)$	48.9125
77		$^{3}J(^{13}C_3,^{1}H_4)$	4.4868
78		$^{3}J(^{13}C_1,^{1}H_6)$	3.7314
79	prop-1-yne (**11**)	$^{2}J(^{13}C_2,^{1}H_6)$	-12.4637
80		$^{1}J(^{13}C_3,^{1}H_6)$	127.8878
81		$^{4}J(^{1}H,^{1}H)$	-4.1353
82		$^{2}J(^{1}H,^{1}H)$	-18.5285
83		$^{1}J(^{13}C,^{13}C)$	41.7563
84		$^{1}J(^{13}C_1,^{1}H_4)$	163.5164
85		$^{2}J(^{13}C_2,^{1}H_4)$	26.7519
86		$^{2}J(^{13}C_1,^{1}H_5)$	-8.6298

87	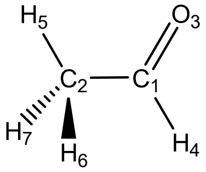 acetaldehyde (**12**)	$^{1}J(^{13}C_{2},^{1}H_{5})$	131.4339
88		$^{3}J_{trans}(^{1}H_{5},^{1}H_{4})$	8.0201
89		$^{2}J(^{13}C_{1},^{1}H_{6})$	-6.8614
90		$^{1}J(^{13}C_{2},^{1}H_{6})$	119.8226
91		$^{3}J_{gauche}(^{1}H_{6},^{1}H_{4})$	0.2867
92		$^{2}J(^{1}H_{6},^{1}H_{5})$	-14.6350
93		$^{2}J(^{1}H_{6},^{1}H_{7})$	-21.1252
94	N≡C−CH_3 acetonitrile (**13**)	$^{1}J(^{13}C,^{13}C)$	61.5337
95		$^{2}J(^{15}N,^{13}C)$	2.6737
96		$^{1}J(^{15}N,^{13}C)$	-16.0611
97		$^{1}J(^{13}C,^{1}H)$	131.2867
98		$^{2}J(^{13}C,^{1}H)$	-11.4635
99		$^{3}J(^{15}N,^{1}H)$	-1.4318
100		$^{2}J(^{1}H,^{1}H)$	-18.6840
101	H_3C−F fluoromethane (**14**)	$^{1}J(^{19}F,^{13}C)$	-148.2300
102		$^{1}J(^{13}C,^{1}H)$	143.9703
103		$^{2}J(^{19}F,^{1}H)$	47.2390
104		$^{2}J(^{1}H,^{1}H)$	-11.9488
105	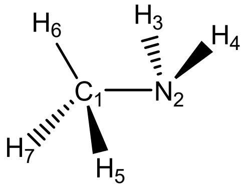 methanamine (**15**)	$^{1}J(^{15}N,^{13}C)$	-6.5060
106		$^{2}J(^{13}C,^{1}H)$	-3.9919
107		$^{1}J(^{15}N,^{1}H)$	-71.4285
108		$^{2}J(^{1}H_{3},^{1}H_{4})$	-11.9990
109		$^{1}J(^{13}C_{1},^{1}H_{5})$	129.6174
110		$^{2}J(^{15}N_{2},^{1}H_{5})$	-1.5586
111		$^{3}J(^{1}H_{5},^{1}H_{4})$	2.6265
112		$^{3}J(^{1}H_{5},^{1}H_{3})$	15.5778
113		$^{2}J(^{1}H_{7},^{1}H_{5})$	-16.8003
114		$^{1}J(^{13}C_{1},^{1}H_{6})$	125.8782
115		$^{2}J(^{15}N_{2},^{1}H_{6})$	1.2869
116		$^{3}J(^{1}H_{6},^{1}H_{4})$	2.0781
117		$^{2}J(^{1}H_{6},^{1}H_{5})$	-12.4496
118	CH_4 methane (**16**)	$^{1}J(^{13}C,^{1}H)$	122.1788
119		$^{2}J(^{1}H,^{1}H)$	-15.1387
120	CHF_3 fluoroform (**17**)	$^{1}J(^{13}C,^{1}H)$	225.4329
121		$^{1}J(^{19}F,^{13}C)$	-246.5827
122		$^{2}J(^{19}F,^{1}H)$	70.6515
123		$^{2}J(^{19}F,^{19}F)$	128.9339
124	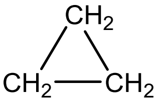 cyclopropane (**18**)	$^{1}J(^{13}C,^{13}C)$	13.6835
125		$^{1}J(^{13}C,^{1}H)$	156.2660
126		$^{2}J(^{13}C,^{1}H)$	-3.3251
127		$^{2}J(^{1}H,^{1}H)$	-7.0308
128		$^{3}J_{cis}(^{1}H,^{1}H)$	9.0904
129		$^{3}J_{trans}(^{1}H,^{1}H)$	4.9277
130	F−C≡C−F 1,2-difluoroethyne (**19**)	$^{1}J(^{13}C,^{13}C)$	405.0362
131		$^{1}J(^{19}F,^{13}C)$	-265.5036

132		$^{2}J(^{19}F,^{13}C)$	41.6643
133		$^{3}J(^{19}F,^{19}F)$	-14.3536
134	F−C≡N<br>fluoroformonitrile (**20**)	$^{1}J(^{19}F,^{13}C)$	-380.1792
135		$^{1}J(^{15}N,^{13}C)$	-1.6907
136		$^{2}J(^{19}F,^{15}N)$	52.3731
137	$H_2C{=}CF_2$<br>1,1-difluoroethene (**21**)	$^{1}J(^{13}C,^{13}C)$	116.1690
138		$^{2}J(^{19}F,^{13}C)$	25.8715
139		$^{1}J(^{19}F,^{13}C)$	-269.4140
140		$^{2}J(^{19}F,^{19}F)$	32.2418
141		$^{1}J(^{13}C,^{1}H)$	164.9397
142		$^{2}J(^{13}C,^{1}H)$	-3.1446
143		$^{3}J_{cis}(^{19}F,^{1}H)$	-2.1808
144		$^{3}J_{trans}(^{19}F,^{1}H)$	24.7717
145		$^{2}J(^{1}H,^{1}H)$	-7.5745
146	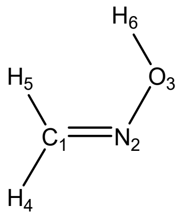<br>formaldehyde oxime (*Z*) (**22**)	$^{1}J(^{15}N,^{13}C)$	-3.0612
147		$^{1}J(^{13}C_1,^{1}H_4)$	183.6663
148		$^{2}J(^{15}N_2,^{1}H_4)$	-12.8617
149		$^{1}J(^{13}C_1,^{1}H_5)$	152.9317
150		$^{2}J(^{15}N_2,^{1}H_5)$	3.0809
151		$^{2}J(^{1}H,^{1}H)$	5.5790
152		$^{3}J(^{13}C,^{1}H)$	4.2396
153		$^{2}J(^{15}N_2,^{1}H_6)$	1.4722
154		$^{4}J(^{1}H_6,^{1}H_4)$	-1.7120
155		$^{4}J(^{1}H_6,^{1}H_5)$	1.8655
156	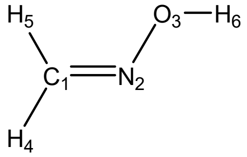<br>formaldehyde oxime (*E*) (**23**)	$^{1}J(^{15}N,^{13}C)$	-6.6861
157		$^{1}J(^{13}C_1,^{1}H_4)$	176.6929
158		$^{2}J(^{15}N_2,^{1}H_4)$	-13.8004
159		$^{1}J(^{13}C_1,^{1}H_5)$	162.3157
160		$^{2}J(^{15}N_2,^{1}H_5)$	3.0774
161		$^{2}J(^{1}H,^{1}H)$	7.2621
162		$^{3}J(^{13}C,^{1}H)$	10.9209
163		$^{2}J(^{15}N_2,^{1}H_6)$	-2.4701
164		$^{4}J(^{1}H_6,^{1}H_4)$	0.7699
165		$^{4}J(^{1}H_6,^{1}H_5)$	-1.5328
166	H−C≡C−F<br>fluoroethyne (**24**)	$^{1}J(^{13}C,^{13}C)$	273.2713
167		$^{2}J(^{19}F,^{13}C)$	25.3812
168		$^{1}J(^{19}F,^{13}C)$	-273.0684
169		$^{1}J(^{13}C,^{1}H)$	281.7540
170		$^{2}J(^{13}C,^{1}H)$	63.6258
171		$^{3}J(^{19}F,^{1}H)$	9.1336
172	H−C≡N<br>hydrogen cyanide (**25**)	$^{1}J(^{15}N,^{13}C)$	-17.3271
173		$^{1}J(^{13}C,^{1}H)$	260.8049
174		$^{2}J(^{15}N,^{1}H)$	-8.9134
175		$^{1}J(^{13}C,^{13}C)$	143.1239
176		$^{2}J(^{19}F_3,^{13}C_1)$	61.6309

177	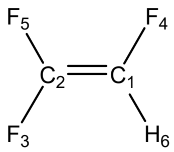 1,1,2-trifluoroethene (**26**)	${}^{1}J({}^{19}F_3,{}^{13}C_2)$	-258.1971
178		${}^{1}J({}^{19}F_4,{}^{13}C_1)$	-221.4434
179		${}^{2}J({}^{19}F_4,{}^{13}C_2)$	36.6298
180		${}^{3}J_{trans}({}^{19}F_3,{}^{19}F_4)$	-121.5026
181		${}^{2}J({}^{19}F_5,{}^{13}C_1)$	18.8380
182		${}^{1}J({}^{19}F_5,{}^{13}C_2)$	-271.3637
183		${}^{2}J({}^{19}F,{}^{19}F)$	82.3079
184		${}^{3}J_{cis}({}^{19}F_4,{}^{19}F_5)$	44.4481
185		${}^{1}J({}^{13}C,{}^{1}H)$	205.9592
186		${}^{2}J({}^{13}C,{}^{1}H)$	12.9895
187		${}^{3}J_{cis}({}^{19}F_3,{}^{1}H_6)$	-5.9909
188		${}^{2}J({}^{19}F,{}^{1}H)$	68.6549
189		${}^{3}J_{trans}({}^{19}F_5,{}^{1}H_6)$	7.0439
190	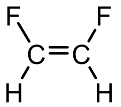 (*Z*)-1,2-difluoroethene (**27**)	${}^{1}J({}^{13}C,{}^{13}C)$	100.4622
191		${}^{1}J({}^{19}F,{}^{13}C)$	-238.1184
192		${}^{2}J({}^{19}F,{}^{13}C)$	9.8360
193		${}^{3}J({}^{19}F,{}^{19}F)$	-6.6435
194		${}^{1}J({}^{13}C,{}^{1}H)$	197.4499
195		${}^{2}J({}^{13}C,{}^{1}H)$	21.5904
196		${}^{2}J({}^{19}F,{}^{1}H)$	68.6528
197		${}^{3}J({}^{19}F,{}^{1}H)$	12.7909
198		${}^{3}J({}^{1}H,{}^{1}H)$	3.7970
199	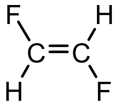 (*E*)-1,2-difluoroethene (**28**)	${}^{1}J({}^{13}C,{}^{13}C)$	113.9197
200		${}^{1}J({}^{19}F,{}^{13}C)$	-225.8784
201		${}^{2}J({}^{19}F,{}^{13}C)$	47.7999
202		${}^{3}J({}^{19}F,{}^{19}F)$	-133.2034
203		${}^{2}J({}^{13}C,{}^{1}H)$	3.2840
204		${}^{1}J({}^{13}C,{}^{1}H)$	197.1529
205		${}^{3}J({}^{19}F,{}^{1}H)$	-0.5443
206		${}^{2}J({}^{19}F,{}^{1}H)$	72.5884
207		${}^{3}J({}^{1}H,{}^{1}H)$	10.4173
208	HF hydrogen fluoride (**29**)	${}^{1}J({}^{19}F,{}^{1}H)$	496.4790
209	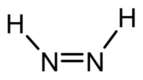 diazene (*Z*) (**31**)	${}^{1}J({}^{15}N,{}^{15}N)$	-22.1434
210		${}^{1}J({}^{15}N,{}^{1}H)$	-37.1092
211		${}^{2}J({}^{15}N,{}^{1}H)$	0.8961
212		${}^{3}J({}^{1}H,{}^{1}H)$	39.8444
213	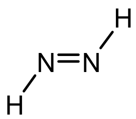 diazene (*E*) (**30**)	${}^{1}J({}^{15}N,{}^{15}N)$	-21.6989
214		${}^{2}J({}^{15}N,{}^{1}H)$	-1.2723
215		${}^{1}J({}^{15}N,{}^{1}H)$	-48.8605
216		${}^{3}J({}^{1}H,{}^{1}H)$	39.1178
217		${}^{1}J({}^{15}N,{}^{15}N)$	1.4923
218		${}^{1}J({}^{15}N,{}^{1}H)$	-64.1932
219		${}^{2}J({}^{15}N,{}^{1}H)$	-1.7624
220		${}^{2}J({}^{1}H,{}^{1}H)$	-16.6857

221	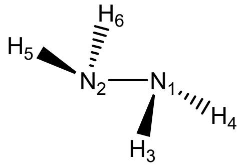	$^{3}J(^{1}H_5,^{1}H_3)$	1.3858
222	hydrazine (**32**)	$^{3}J(^{1}H_4,^{1}H_5)$	13.4227
223	N_2 molecular nitrogen (**33**)	$^{1}J(^{15}N,^{15}N)$	-2.80381
224	NH_3	$^{1}J(^{15}N,^{1}H)$	-67.3983
225	ammonia (**34**)	$^{2}J(^{1}H,^{1}H)$	-12.6291
226		$^{1}J(^{13}C_1,^{13}C_2)$	54.4454
227		$^{1}J(^{13}C_3,^{13}C_1)$	69.7182
228		$^{2}J(^{13}C_3,^{13}C_2)$	1.4797
229		$^{2}J(^{15}N_4,^{13}C_1)$	2.912682
230		$^{1}J(^{15}N_4,^{13}C_2)$	-2.42663
231		$^{2}J(^{15}N_4,^{13}C_3)$	0.966919
232		$^{2}J(^{15}N_5,^{13}C_1)$	-6.3312
233		$^{2}J(^{15}N_5,^{13}C_2)$	-0.76983
234		$^{1}J(^{15}N_5,^{13}C_3)$	-17.5492
235		$^{1}J(^{15}N,^{15}N)$	-13.595
236		$^{1}J(^{13}C_1,^{1}H_6)$	171.3601
237		$^{2}J(^{13}C_2,^{1}H_6)$	4.5181
238		$^{2}J(^{13}C3,^{1}H_6)$	6.8888
239		$^{3}J(^{15}N_4,^{1}H_6)$	-1.17397
240	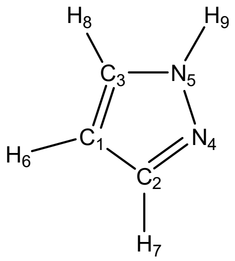	$^{3}J(^{15}N_5,^{1}H_6)$	-6.41578
241		$^{2}J(^{13}C_1,^{1}H_7)$	9.7189
242		$^{1}J(^{13}C_2,^{1}H_7)$	180.6619
243		$^{3}J(^{13}C_3,^{1}H_7)$	4.8474
244		$^{2}J(^{15}N_4,^{1}H_7)$	-12.8293
245		$^{3}J(^{15}N_5,^{1}H_7)$	-9.80119
246		$^{3}J(^{1}H_7,^{1}H_6)$	1.8425
247	1*H*-pyrazole (**35**)	$^{2}J(^{13}C_1,^{1}H_8)$	6.056
248		$^{3}J(^{13}C_2,^{1}H_8)$	8.076
249		$^{1}J(^{13}C_3,^{1}H_8)$	179.2456
250		$^{3}J(^{15}N_4,^{1}H_8)$	0.443972
251		$^{2}J(^{15}N_5,^{1}H_8)$	-4.82464
252		$^{3}J(^{1}H_8,^{1}H_6)$	3.0464
253		$^{4}J(^{1}H_8,^{1}H_7)$	-0.2914
254		$^{3}J(^{13}C_1,^{1}H_9)$	5.3713
255		$^{3}J(^{13}C_2,^{1}H_9)$	10.2248
256		$^{2}J(^{13}C_3,^{1}H_9)$	8.8113
257		$^{2}J(^{15}N_4,^{1}H_9)$	-9.47098
258		$^{1}J(^{15}N,^{1}H)$	-119.135
259		$^{4}J(^{1}H_9,^{1}H_6)$	1.6655
260		$^{4}J(^{1}H_9,^{1}H_7)$	1.5249
261		$^{3}J(^{1}H_9,^{1}H_8)$	1.5985

262		$^{1}J(^{13}C,^{13}C)$	70.9199
263		$^{2}J(^{13}C_{1},^{13}C_{3})$	-4.4285
264		$^{2}J(^{13}C_{3},^{13}C_{2})$	9.1289
265		$^{1}J(^{15}N_{4},^{13}C_{1})$	1.231058
266		$^{2}J(^{15}N_{4},^{13}C_{2})$	2.450333
267		$^{1}J(^{15}N_{4},^{13}C_{3})$	-2.79443
268		$^{2}J(^{15}N_{5},^{13}C_{1})$	-6.83507
269		$^{1}J(^{15}N_{5},^{13}C_{2})$	-19.6076
270		$^{1}J(^{15}N_{5},^{13}C_{3})$	-15.0639
271		$^{2}J(^{15}N,^{15}N)$	-1.61157
272		$^{1}J(^{13}C_{1},^{1}H_{6})$	184.3971
273		$^{2}J(^{13}C_{2},^{1}H_{6})$	14.4545
274		$^{3}J(^{13}C_{3},^{1}H_{6})$	11.5652
275		$^{2}J(^{15}N_{4},^{1}H_{6})$	-10.3739
276	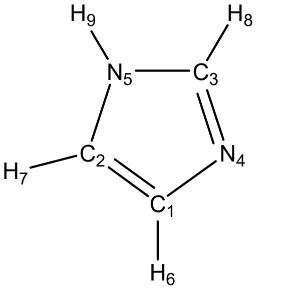	$^{3}J(^{15}N_{5},^{1}H_{6})$	-3.97415
277		$^{2}J(^{13}C_{1},^{1}H_{7})$	7.3057
278		$^{1}J(^{13}C_{2},^{1}H_{7})$	183.1321
279		$^{3}J(^{13}C_{3},^{1}H_{7})$	6.5776
280		$^{3}J(^{15}N_{4},^{1}H_{7})$	-1.21703
281		$^{2}J(^{15}N_{5},^{1}H_{7})$	-4.94612
282		$^{3}J(^{1}H_{7},^{1}H_{6})$	2.2029
283	1*H*-imidazole (**36**)	$^{3}J(^{13}C_{1},^{1}H_{8})$	11.5459
284		$^{3}J(C_{2},^{1}H_{8})$	3.1595
285		$^{1}J(C_{3},^{1}H_{8})$	200.4769
286		$^{2}J(^{15}N_{4},^{1}H_{8})$	-11.7791
287		$^{2}J(^{15}N_{5},^{1}H_{8})$	-9.60523
288		$^{4}J(^{1}H_{8},^{1}H_{6})$	-0.1768
289		$^{4}J(^{1}H8,^{1}H_{7})$	1.0754
290		$^{3}J(^{13}C_{1},^{1}H_{9})$	7.7791
291		$^{2}J(^{13}C_{2},^{1}H_{9})$	4.2316
292		$^{2}J(^{13}C_{3},^{1}H_{9})$	4.3053
293		$^{3}J(^{15}N_{4},^{1}H_{9})$	0.250953
294		$^{1}J(^{15}N,^{1}H)$	-107.568
295		$^{4}J(^{1}H_{9},^{1}H_{6})$	1.3996
296		$^{3}J(^{1}H_{9},^{1}H_{7})$	1.9791
297		$^{3}J(^{1}H_{9},^{1}H_{8})$	0.935
298	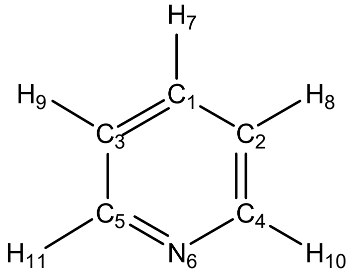	$^{1}J(^{13}C_{1},^{13}C_{2})$	56.8395
299		$^{2}J(^{13}C_{2},^{13}C_{3})$	-4.4775
300		$^{2}J(^{13}C_{1},^{13}C_{4})$	-4.0321
301		$^{1}J(^{13}C_{2},^{13}C_{4})$	57.2558
302		$^{3}J(^{13}C_{3},^{13}C_{4})$	14.9543
303		$^{2}J(^{13}C_{5},^{13}C_{4})$	-7.421
304		$^{3}J(^{15}N_{6},^{13}C_{1})$	-4.68128
305	pyridine (**37**)	$^{2}J(^{15}N_{6},^{13}C_{2})$	3.020694
306		$^{1}J(^{15}N,^{13}C)$	-2.05728

307		$^{1}J(^{13}C_{1},^{1}H_{7})$	155.6056
308		$^{2}J(^{13}C_{2},^{1}H_{7})$	-1.1409
309		$^{3}J(^{13}C_{4},^{1}H_{7})$	7.0619
310		$^{4}J(^{15}N,^{1}H)$	0.570641
311		$^{2}J(^{13}C_{1},^{1}H_{8})$	-0.9968
312		$^{1}J(^{13}C_{2},^{1}H_{8})$	158.0209
313		$^{3}J(^{13}C_{3},^{1}H_{8})$	6.8531
314		$^{2}J(^{13}C_{4},^{1}H_{8})$	1.1585
315		$^{4}J(^{13}C_{5},^{1}H_{8})$	-1.5936
316		$^{3}J(^{15}N,^{1}H)$	-1.7714
317		$^{3}J(^{1}H_{7},^{1}H_{8})$	8.1841
318		$^{4}J(^{1}H_{8},^{1}H_{9})$	0.4062
319		$^{3}J(^{13}C_{1},^{1}H_{10})$	6.8853
320		$^{2}J(^{13}C_{2},^{1}H_{10})$	6.6117
321		$^{4}J(^{13}C_{3},^{1}H_{10})$	-2.4133
322		$^{1}J(^{13}C_{4},^{1}H_{10})$	172.5818
323		$^{3}J(^{13}C_{5},^{1}H_{10})$	11.5865
324		$^{2}J(^{15}N,^{1}H)$	-10.6416
325		$^{4}J(^{1}H_{7},^{1}H_{10})$	0.9478
326		$^{3}J(^{1}H_{8},^{1}H_{10})$	5.6236
327		$^{5}J(^{1}H,^{1}H)$	1.321
328		$^{4}J(^{1}H_{10},^{1}H_{11})$	-1.0689
329		$^{2}J(^{15}N,^{13}C)$	-4.8071
330		$^{1}J(^{13}C_{2},^{13}C_{3})$	55.2434
331		$^{1}J(^{15}N,^{13}C)$	-18.4715
332		$^{1}J(^{13}C_{5},^{13}C_{3})$	70.9584
333		$^{2}J(^{13}C_{2},^{13}C_{5})$	0.6925
334		$^{2}J(^{13}C_{4},^{13}C_{5})$	7.5659
335		$^{3}J(^{15}N,^{1}H)$	-6.12752
336	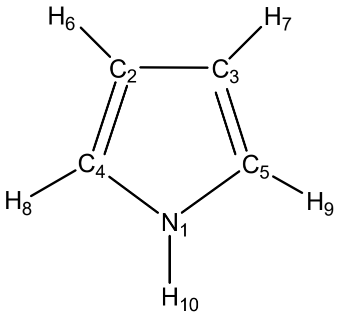	$^{1}J(^{13}C_{3},^{1}H_{7})$	165.9816
337		$^{2}J(^{13}C_{2},^{1}H_{7})$	2.6731
338		$^{2}J(^{13}C_{5},^{1}H_{7})$	6.1581
339		$^{3}J(^{13}C_{4},^{1}H_{7})$	7.4654
340		$^{3}J(^{1}H_{6},^{1}H_{7})$	3.552
341		$^{2}J(^{15}N,^{1}H)$	-4.25708
342		$^{2}J(^{13}C_{3},^{1}H_{9})$	5.3371
343		$^{3}J(^{13}C_{2},^{1}H_{9})$	7.9213
344	1*H*-pyrrole (**38**)	$^{1}J(^{13}C_{5},^{1}H_{9})$	178.4026
345		$^{3}J(^{13}C_{4},^{1}H_{9})$	5.7974
346		$^{3}J(^{1}H_{7},^{1}H_{9})$	3.6481
347		$^{4}J(^{1}H_{6},^{1}H_{9})$	0.5095
348		$^{4}J(^{1}H_{8},^{1}H_{9})$	1.8406
349		$^{1}J(^{15}N,^{1}H)$	-107.44
350		$^{3}J(^{13}C_{3},^{1}H_{10})$	7.0534
351		$^{2}J(^{13}C_{5},^{1}H_{10})$	3.3181

352		$^{4}J(^{1}H_{7},^{1}H_{10})$	2.1584
353		$^{3}J(^{1}H_{9},^{1}H_{10})$	2.6795
354		$^{1}J(^{13}C_{2},^{13}C_{1})$	73.4006
355		$^{2}J(^{13}C,^{13}C)$	-0.3931
356		$^{1}J(^{13}C_{2},^{13}C_{3})$	43.878
357		$^{1}J(^{13}C_{1},^{1}H_{4})$	155.0497
358		$^{2}J(^{13}C_{2},^{1}H_{4})$	-3.0043
359		$^{3}J(^{13}C_{3},^{1}H_{4})$	12.1355
360		$^{1}J(^{13}C_{1},^{1}H_{5})$	151.4836
361		$^{2}J(^{13}C_{2},^{1}H_{5})$	-4.3047
362		$^{3}J(^{13}C_{3},^{1}H_{5})$	7.8319
363		$^{2}J(^{1}H_{4},^{1}H_{5})$	-1.0971
364		$^{2}J(^{13}C_{1},^{1}H_{6})$	-1.6586
365		$^{1}J(^{13}C_{2},^{1}H_{6})$	148.5103
366		$^{2}J(^{13}C_{3},^{1}H_{6})$	3.6255
367		$^{3}J(^{1}H_{4},^{1}H_{6})$	11.247
368	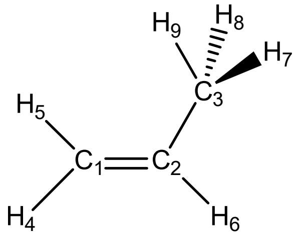	$^{3}J(^{1}H_{5},^{1}H_{6})$	16.8702
369		$^{3}J(^{13}C_{1},^{1}H_{7})$	6.3149
370		$^{2}J(^{13}C_{2},^{1}H_{7})$	-8.8155
371	prop-1-ene (**39**)	$^{1}J(^{13}C_{3},^{1}H_{7})$	122.4548
372		$^{4}J(^{1}H_{4},^{1}H_{7})$	-3.1609
373		$^{4}J(^{1}H_{5},^{1}H_{7})$	-3.4219
374		$^{3}J(^{1}H_{6},^{1}H_{7})$	4.0525
375		$^{2}J(^{1}H_{7},^{1}H_{8})$	-20.244
376		$^{3}J(^{13}C_{1},^{1}H_{9})$	6.3603
377		$^{2}J(^{13}C_{2},^{1}H_{9})$	-6.4831
378		$^{1}J(^{13}C_{3},^{1}H_{9})$	123.6607
379		$^{4}J(^{1}H_{4},^{1}H_{9})$	-0.618
380		$^{4}J(^{1}H_{5},^{1}H_{9})$	-0.5183
381		$^{3}J(^{1}H_{6},^{1}H_{9})$	12.0774
382		$^{2}J(^{1}H_{7},^{1}H_{9})$	-15.3175
383		$^{1}J(^{13}C_{1},^{13}C_{2})$	53.1061
384		$^{1}J(^{13}C_{1},^{13}C_{3})$	73.4324
385		$^{2}J(^{13}C_{2},^{13}C_{3})$	-0.215
386		$^{2}J(^{13}C_{3},^{13}C_{4})$	4.0168
387		$^{1}J(^{13}C_{1},^{1}H_{6})$	169.9864
388		$^{2}J(^{13}C_{2},^{1}H_{6})$	2.6105
389	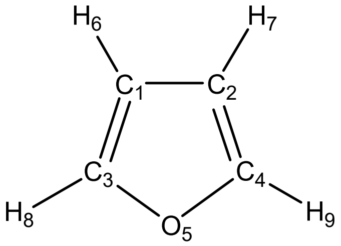	$^{2}J(^{13}C_{3},^{1}H_{6})$	7.7765
390		$^{3}J(^{13}C_{4},^{1}H_{6})$	6.9254
391		$^{3}J(^{1}H_{6},^{1}H_{7})$	3.1457
392		$^{2}J(^{13}C_{1},^{1}H_{8})$	11.225
393	furan (**40**)	$^{3}J(^{13}C_{2},^{1}H_{8})$	6.4916
394		$^{1}J(^{13}C_{3},^{1}H_{8})$	195.8161
395		$^{3}J(^{13}C_{4},^{1}H_{8})$	6.5993
396		$^{3}J(^{1}H_{6},^{1}H_{8})$	2.894

397		$^{4}J(^{1}H_{7},^{1}H_{8})$	-0.111
398		$^{4}J(^{1}H_{8},^{1}H_{9})$	1.1381
399		$^{1}J(^{19}F,^{13}C)$	-230.125
400		$^{2}J(^{19}F,^{13}C)$	20.4143
401		$^{1}J(^{13}C_{2},^{13}C_{3})$	72.7931
402		$^{2}J(^{13}C_{3},^{13}C_{4})$	2.3653
403		$^{3}J(^{19}F,^{13}C)$	4.4691
404		$^{2}J(^{13}C_{2},^{13}C_{6})$	-1.0859
405		$^{1}J(^{13}C_{3},^{13}C_{6})$	59.8373
406		$^{3}J(^{13}C_{4},^{13}C_{6})$	7.895
407		$^{2}J(^{13}C_{5},^{13}C_{6})$	-2.8679
408		$^{4}J(^{19}F,^{13}C)$	5.4833
409		$^{3}J(^{13}C_{7},^{13}C_{2})$	11.6145
410		$^{2}J(^{13}C_{3},^{13}C_{7})$	-4.3007
411		$^{1}J(^{13}C_{6},^{13}C_{7})$	59.2043
412		$^{3}J(^{19}F,^{1}H)$	5.972
413	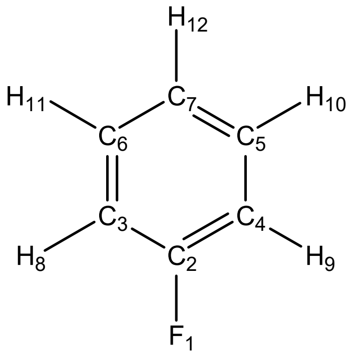	$^{2}J(^{13}C_{2},^{1}H_{8})$	-7.0576
414		$^{1}J(^{13}C_{3},^{1}H_{8})$	159.5646
415		$^{3}J(^{13}C_{4},^{1}H_{8})$	4.7319
416		$^{2}J(^{13}C_{6},^{1}H_{8})$	-2.1434
417		$^{4}J(^{13}C_{5},^{1}H_{8})$	-1.5412
418		$^{3}J(^{13}C_{7},^{1}H_{8})$	8.1316
419		$^{4}J(^{1}H_{8},^{1}H_{9})$	1.6384
420		$^{4}J(^{19}F,^{1}H)$	4.7488
421		$^{3}J(^{13}C_{2},^{1}H_{11})$	11.0597
422	1-fluorobenzene (**41**)	$^{2}J(^{13}C_{3},^{1}H_{11})$	-0.9733
423		$^{4}J(^{13}C_{4},^{1}H_{11})$	-2.2112
424		$^{1}J(^{13}C_{6},^{1}H_{11})$	156.1276
425		$^{3}J(^{13}C_{5},^{1}H_{11})$	9.298
426		$^{2}J(^{13}C_{7},^{1}H_{11})$	-1.2653
427		$^{3}J(^{1}H_{8},^{1}H_{11})$	8.9262
428		$^{5}J(^{1}H_{9},^{1}H_{11})$	0.9123
429		$^{4}J(^{1}H_{10},^{1}H_{11})$	0.7664
430		$^{5}J(^{19}F,^{1}H)$	-1.5872
431		$^{4}J(^{13}C_{2},^{1}H_{12})$	-2.6151
432		$^{3}J(^{13}C_{3},^{1}H_{12})$	8.5231
433		$^{2}J(^{13}C_{6},^{1}H_{12})$	-0.3794
434		$^{1}J(^{13}C_{7},^{1}H_{12})$	157.7466
435		$^{4}J(^{1}H_{8},^{1}H_{12})$	0.1704
436		$^{3}\mathrm{J}(^{1}H_{11},^{1}H_{12})$	8.1847

Table S7. Symmetry independent values of SSCC (in Hz) in molecules of set 1 calculated at the SOPPA(CCSD) level with the pecJ-1-new(seg) basis set.

#	Molecule	Type of SSCC [1]	SSCC value
1	$H_2C{=}C{=}CH_2$ propa-1,2-diene (**1**)	$^{1}J(^{13}C,^{13}C)$	103.2956
2		$^{2}J(^{13}C,^{13}C)$	7.7861
3		$^{2}J(^{13}C,^{1}H)$	-7.0731
4		$^{1}J(^{13}C,^{1}H)$	166.5567
5		$^{3}J(^{13}C,^{1}H)$	8.2872
6		$^{2}J(^{1}H,^{1}H)$	-15.1128
7		$^{4}J(^{1}H,^{1}H)$	-9.8249
8	$F_2C{=}CF_2$ Perfluoroethene (**2**)	$^{1}J(^{13}C,^{13}C)$	193.3061
9		$^{1}J(^{13}C,^{19}F)$	-267.3236
10		$^{2}J(^{13}C,^{19}F)$	49.0510
11		$^{2}J(^{19}F,^{19}F)$	121.2691
12		$^{3}J_{cis}(^{19}F,^{19}F)$	84.4263
13		$^{3}J_{trans}(^{19}F,^{19}F)$	-116.8412
14	$HC{\equiv}CH$ ethyne (**3**)	$^{1}J(^{13}C,^{13}C)$	190.1853
15		$^{1}J(^{13}C,^{1}H)$	251.4519
16		$^{2}J(^{13}C,^{1}H)$	49.2284
17		$^{3}J(^{1}H,^{1}H)$	11.5065
18	$H_2C{=}CH_2$ ethene (**4**)	$^{1}J(^{13}C,^{13}C)$	71.5257
19		$^{1}J(^{13}C,^{1}H)$	153.9120
20		$^{2}J(^{13}C,^{1}H)$	-4.1140
21		$^{3}J_{cis}(^{1}H,^{1}H)$	12.5021
22		$^{2}J(^{1}H,^{1}H)$	-1.0388
23		$^{3}J_{trans}(^{1}H,^{1}H)$	18.8351
24	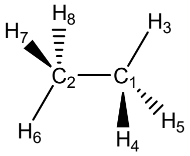 ethane (**5**)	$^{1}J(^{13}C,^{13}C)$	35.1690
25		$^{1}J(^{13}C,^{1}H)$	121.8015
26		$^{2}J(^{13}C,^{1}H)$	-5.4442
27		$^{3}J_{trans}(^{1}H_3,^{1}H_6)$	15.6798
28		$^{2}J(^{1}H,^{1}H)$	-15.3049
29		$^{3}J_{gauche}(^{1}H_4,^{1}H_6)$	3.7248
30	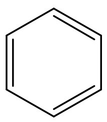 benzene (**6**)	$^{3}J(^{13}C,^{13}C)$	11.0702
31		$^{1}J(^{13}C,^{13}C)$	59.3278
32		$^{2}J(^{13}C,^{13}C)$	-3.8925
33		$^{1}J(^{13}C,^{1}H)$	154.2645
34		$^{4}J(^{13}C,^{1}H)$	-2.0264
35		$^{2}J(^{13}C,^{1}H)$	-0.8812
36		$^{3}J(^{13}C,^{1}H)$	8.0295
37		$^{3}J(^{1}H,^{1}H)$	8.2194
38		$^{4}J(^{1}H,^{1}H)$	0.3916
39		$^{5}J(^{1}H,^{1}H)$	1.0996
40	CF_4 perfluoromethane (**7**)	$^{1}J(^{19}F,^{13}C)$	-261.5640
41		$^{2}J(^{19}F,^{19}F)$	30.8581

42		$^{1}J(^{13}C,^{13}C)$	87.7607
43		$^{2}J(^{19}F,^{13}C)$	12.0266
44		$^{1}J(^{19}F,^{13}C)$	-249.3105
45		$^{1}J(^{13}C_1,^{1}H_4)$	159.0690
46		$^{2}J(^{13}C_2,^{1}H_4)$	5.6731
47	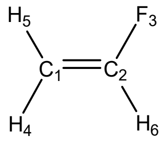	$^{3}J_{trans}(^{19}F_3,^{1}H_4)$	38.2407
48		$^{1}J(^{13}C_1,^{1}H_5)$	158.0234
49		$^{2}J(^{13}C_2,^{1}H_5)$	-11.1600
50		$^{3}J_{cis}(^{19}F_3,^{1}H_5)$	12.4919
51	fluoroethene (**8**)	$^{2}J(^{1}H,^{1}H)$	-5.9613
52		$^{2}J(^{13}C_1,^{1}H_6)$	11.6908
53		$^{1}J(^{13}C_2,^{1}H_6)$	193.3589
54		$^{2}J(^{19}F,^{1}H)$	80.6811
55		$^{3}J_{cis}(^{1}H_6,^{1}H_4)$	6.3880
56		$^{3}J_{trans}(^{1}H_6,^{1}H_5)$	13.2504
57		$^{1}J(^{19}F,^{13}C)$	-218.8895
58	CH_2F_2	$^{2}J(^{19}F,^{19}F)$	321.7389
59	difluoromethane (**9**)	$^{1}J(^{13}C,^{1}H)$	175.6008
60		$^{2}J(^{19}F,^{1}H)$	49.7892
61		$^{2}J(^{1}H,^{1}H)$	-0.6221
62		$^{1}J(^{15}N,^{13}C)$	-3.8797
63		$^{1}J(^{13}C_1,^{1}H_3)$	171.4889
64		$^{2}J(^{15}N_2,^{1}H_3)$	-9.6427
65	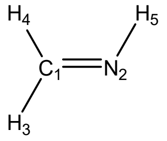	$^{1}J(^{13}C_1,^{1}H_4)$	154.3282
66		$^{2}J(^{15}N_2,^{1}H_4)$	3.7280
67		$^{2}J(^{1}H,^{1}H)$	15.6421
68	methanimine (**10**)	$^{2}J(^{13}C,^{1}H)$	-13.9518
69		$^{1}J(^{15}N,^{1}H)$	-50.2178
70		$^{3}J_{trans}(^{1}H_5,^{1}H_3)$	24.8118
71		$^{3}J_{cis}(^{1}H_5,^{1}H_4)$	18.7004
72		$^{1}J(^{13}C_1,^{13}C_2)$	190.3060
73		$^{2}J(^{13}C,^{13}C)$	12.7027
74		$^{1}J(^{13}C_2,^{13}C_3)$	70.9952
75		$^{1}J(^{13}C_1,^{1}H_4)$	250.2732
76	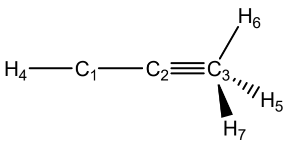	$^{2}J(^{13}C_2,^{1}H_4)$	48.7208
77		$^{3}J(^{13}C_3,^{1}H_4)$	4.4749
78		$^{3}J(^{13}C_1,^{1}H_6)$	3.7499
79	prop-1-yne (**11**)	$^{2}J(^{13}C_2,^{1}H_6)$	-12.5225
80		$^{1}J(^{13}C_3,^{1}H_6)$	127.7119
81		$^{4}J(^{1}H,^{1}H)$	-4.1382
82		$^{2}J(^{1}H,^{1}H)$	-18.5508
83		$^{1}J(^{13}C,^{13}C)$	42.2622
84		$^{1}J(^{13}C_1,^{1}H_4)$	163.3158
85		$^{2}J(^{13}C_2,^{1}H_4)$	26.6274
86		$^{2}J(^{13}C_1,^{1}H_5)$	-8.6630

87	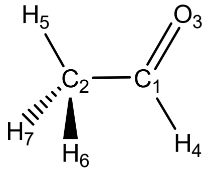 acetaldehyde (**12**)	$^{1}J(^{13}C_{2},^{1}H_{5})$	131.3272
88		$^{3}J_{trans}(^{1}H_{5},^{1}H_{4})$	7.9989
89		$^{2}J(^{13}C_{1},^{1}H_{6})$	-6.9196
90		$^{1}J(^{13}C_{2},^{1}H_{6})$	119.7317
91		$^{3}J_{gauche}(^{1}H_{6},^{1}H_{4})$	0.3001
92		$^{2}J(^{1}H_{6},^{1}H_{5})$	-14.6607
93		$^{2}J(^{1}H_{6},^{1}H_{7})$	-21.1412
94	$N{\equiv}C{-}CH_3$ acetonitrile (**13**)	$^{1}J(^{13}C,^{13}C)$	62.1972
95		$^{2}J(^{15}N,^{13}C)$	2.4635
96		$^{1}J(^{15}N,^{13}C)$	-15.6866
97		$^{1}J(^{13}C,^{1}H)$	131.1163
98		$^{2}J(^{13}C,^{1}H)$	-11.5270
99		$^{3}J(^{15}N,^{1}H)$	-1.2823
100		$^{2}J(^{1}H,^{1}H)$	-18.7062
101	$H_3C{-}F$ fluoromethane (**14**)	$^{1}J(^{19}F,^{13}C)$	-156.4206
102		$^{1}J(^{13}C,^{1}H)$	144.0860
103		$^{2}J(^{19}F,^{1}H)$	48.2577
104		$^{2}J(^{1}H,^{1}H)$	-12.0415
105	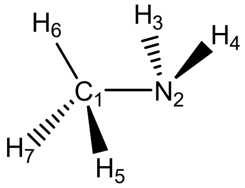 methanamine (**15**)	$^{1}J(^{15}N,^{13}C)$	-6.2468
106		$^{2}J(^{13}C,^{1}H)$	-4.0376
107		$^{1}J(^{15}N,^{1}H)$	-66.6839
108		$^{2}J(^{1}H_{3},^{1}H_{4})$	-12.0631
109		$^{1}J(^{13}C_{1},^{1}H_{5})$	129.4773
110		$^{2}J(^{15}N_{2},^{1}H_{5})$	-1.4398
111		$^{3}J(^{1}H_{5},^{1}H_{4})$	2.6362
112		$^{3}J(^{1}H_{5},^{1}H_{3})$	15.5260
113		$^{2}J(^{1}H_{7},^{1}H_{5})$	-16.8334
114		$^{1}J(^{13}C_{1},^{1}H_{6})$	125.7486
115		$^{2}J(^{15}N_{2},^{1}H_{6})$	1.2149
116		$^{3}J(^{1}H_{6},^{1}H_{4})$	2.0718
117		$^{2}J(^{1}H_{6},^{1}H_{5})$	-12.4821
118	CH_4 methane (**16**)	$^{1}J(^{13}C,^{1}H)$	122.1742
119		$^{2}J(^{1}H,^{1}H)$	-15.1678
120	CHF_3 fluoroform (**17**)	$^{1}J(^{13}C,^{1}H)$	225.0272
121		$^{1}J(^{19}F,^{13}C)$	-256.5136
122		$^{2}J(^{19}F,^{1}H)$	70.6170
123		$^{2}J(^{19}F,^{19}F)$	131.1506
124	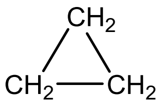 cyclopropane (**18**)	$^{1}J(^{13}C,^{13}C)$	13.9526
125		$^{1}J(^{13}C,^{1}H)$	156.0467
126		$^{2}J(^{13}C,^{1}H)$	-3.3457
127		$^{2}J(^{1}H,^{1}H)$	-7.0852
128		$^{3}J_{cis}(^{1}H,^{1}H)$	9.0951
129		$^{3}J_{trans}(^{1}H,^{1}H)$	4.9244
130	$F{-}C{\equiv}C{-}F$ 1,2-difluoroethyne (**19**)	$^{1}J(^{13}C,^{13}C)$	404.4087
131		$^{1}J(^{19}F,^{13}C)$	-274.9143

132		$^{2}J(^{19}F,^{13}C)$	42.4083
133		$^{3}J(^{19}F,^{19}F)$	-11.8623
134		$^{1}J(^{19}F,^{13}C)$	-391.1444
135	F−C≡N<br>fluoroformonitrile (**20**)	$^{1}J(^{15}N,^{13}C)$	-2.5704
136		$^{2}J(^{19}F,^{15}N)$	49.9862
137		$^{1}J(^{13}C,^{13}C)$	116.2963
138		$^{2}J(^{19}F,^{13}C)$	26.5576
139		$^{1}J(^{19}F,^{13}C)$	-277.8671
140		$^{2}J(^{19}F,^{19}F)$	33.3315
141	$H_2C{=}CF_2$<br>1,1-difluoroethene (**21**)	$^{1}J(^{13}C,^{1}H)$	165.1423
142		$^{2}J(^{13}C,^{1}H)$	-3.3065
143		$^{3}J_{cis}(^{19}F,^{1}H)$	-1.6135
144		$^{3}J_{trans}(^{19}F,^{1}H)$	25.4807
145		$^{2}J(^{1}H,^{1}H)$	-7.8086
146		$^{1}J(^{15}N,^{13}C)$	-2.6955
147		$^{1}J(^{13}C_1,^{1}H_4)$	183.6081
148		$^{2}J(^{15}N_2,^{1}H_4)$	-11.8871
149		$^{1}J(^{13}C_1,^{1}H_5)$	152.8621
150	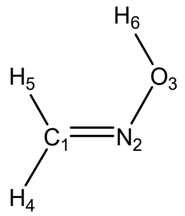	$^{2}J(^{15}N_2,^{1}H_5)$	2.9278
151		$^{2}J(^{1}H,^{1}H)$	5.4998
152		$^{3}J(^{13}C,^{1}H)$	4.2058
153	formaldehyde oxime (*Z*) (**22**)	$^{2}J(^{15}N_2,^{1}H_6)$	1.4207
154		$^{4}J(^{1}H_6,^{1}H_4)$	-1.7018
155		$^{4}J(^{1}H_6,^{1}H_5)$	1.8698
156		$^{1}J(^{15}N,^{13}C)$	-6.0297
157		$^{1}J(^{13}C_1,^{1}H_4)$	176.6109
158		$^{2}J(^{15}N_2,^{1}H_4)$	-12.7575
159		$^{1}J(^{13}C_1,^{1}H_5)$	162.2363
160	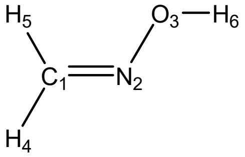	$^{2}J(^{15}N_2,^{1}H_5)$	2.9132
161		$^{2}J(^{1}H,^{1}H)$	7.1886
162	formaldehyde oxime (*E*) (**23**)	$^{3}J(^{13}C,^{1}H)$	10.8879
163		$^{2}J(^{15}N_2,^{1}H_6)$	-2.2340
164		$^{4}J(^{1}H_6,^{1}H_4)$	0.7507
165		$^{4}J(^{1}H_6,^{1}H_5)$	-1.5354
166		$^{1}J(^{13}C,^{13}C)$	273.2115
167		$^{2}J(^{19}F,^{13}C)$	25.9777
168	H−C≡C−F	$^{1}J(^{19}F,^{13}C)$	-280.9775
169	fluoroethyne (**24**)	$^{1}J(^{13}C,^{1}H)$	281.4847
170		$^{2}J(^{13}C,^{1}H)$	63.1987
171		$^{3}J(^{19}F,^{1}H)$	9.2416
172		$^{1}J(^{15}N,^{13}C)$	-16.9015
173	H−C≡N<br>hydrogen cyanide (**25**)	$^{1}J(^{13}C,^{1}H)$	260.7235
174		$^{2}J(^{15}N,^{1}H)$	-8.6843
175		$^{1}J(^{13}C,^{13}C)$	143.1338
176		$^{2}J(^{19}F_3,^{13}C_1)$	62.4453

177	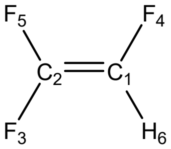 1,1,2-trifluoroethene (**26**)	$^{1}J(^{19}F_3,^{13}C_2)$	-267.6524
178		$^{1}J(^{19}F_4,^{13}C_1)$	-231.3522
179		$^{2}J(^{19}F_4,^{13}C_2)$	37.3407
180		$^{3}J_{trans}(^{19}F_3,^{19}F_4)$	-122.3994
181		$^{2}J(^{19}F_5,^{13}C_1)$	19.6408
182		$^{1}J(^{19}F_5,^{13}C_2)$	-280.4599
183		$^{2}J(^{19}F,^{19}F)$	80.9012
184		$^{3}J_{cis}(^{19}F_4,^{19}F_5)$	44.3331
185		$^{1}J(^{13}C,^{1}H)$	206.1739
186		$^{2}J(^{13}C,^{1}H)$	12.5156
187		$^{3}J_{cis}(^{19}F_3,^{1}H_6)$	-5.4922
188		$^{2}J(^{19}F,^{1}H)$	70.2473
189		$^{3}J_{trans}(^{19}F_5,^{1}H_6)$	7.4898
190	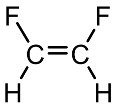 (*Z*)-1,2-difluoroethene (**27**)	$^{1}J(^{13}C,^{13}C)$	100.8834
191		$^{1}J(^{19}F,^{13}C)$	-246.2684
192		$^{2}J(^{19}F,^{13}C)$	10.4042
193		$^{3}J(^{19}F,^{19}F)$	-6.5097
194		$^{1}J(^{13}C,^{1}H)$	197.8190
195		$^{2}J(^{13}C,^{1}H)$	21.2016
196		$^{2}J(^{19}F,^{1}H)$	70.5407
197		$^{3}J(^{19}F,^{1}H)$	13.2695
198		$^{3}J(^{1}H,^{1}H)$	3.9476
199	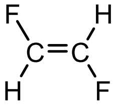 (*E*)-1,2-difluoroethene (**28**)	$^{1}J(^{13}C,^{13}C)$	114.1384
200		$^{1}J(^{19}F,^{13}C)$	-234.2693
201		$^{2}J(^{19}F,^{13}C)$	48.2068
202		$^{3}J(^{19}F,^{19}F)$	-134.0890
203		$^{2}J(^{13}C,^{1}H)$	2.9699
204		$^{1}J(^{13}C,^{1}H)$	197.2186
205		$^{3}J(^{19}F,^{1}H)$	0.0700
206		$^{2}J(^{19}F,^{1}H)$	74.3817
207		$^{3}J(^{1}H,^{1}H)$	10.5278
208	HF hydrogen fluoride (**29**)	$^{1}J(^{19}F,^{1}H)$	515.8182
209	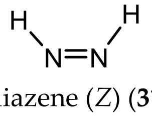 diazene (*Z*) (**31**)	$^{1}J(^{15}N,^{15}N)$	-21.4067
210		$^{1}J(^{15}N,^{1}H)$	-34.2126
211		$^{2}J(^{15}N,^{1}H)$	0.9422
212		$^{3}J(^{1}H,^{1}H)$	39.7112
213	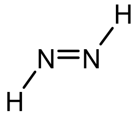 diazene (*E*) (**30**)	$^{1}J(^{15}N,^{15}N)$	-21.6430
214		$^{2}J(^{15}N,^{1}H)$	-0.8728
215		$^{1}J(^{15}N,^{1}H)$	-45.1156
216		$^{3}J(^{1}H,^{1}H)$	38.9397
217		$^{1}J(^{15}N,^{15}N)$	1.6588
218		$^{1}J(^{15}N,^{1}H)$	-59.8814
219		$^{2}J(^{15}N,^{1}H)$	-1.6900
220		$^{2}J(^{1}H,^{1}H)$	-16.7328

221	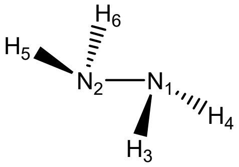	$^{3}J(^{1}H_{5},^{1}H_{3})$	1.3889
222	hydrazine (**32**)	$^{3}J(^{1}H_{4},^{1}H_{5})$	13.3531
223	N_2 molecular nitrogen (**33**)	$^{1}J(^{15}N,^{15}N)$	-3.32762
224	NH_3	$^{1}J(^{15}N,^{1}H)$	-62.8784
225	ammonia (**34**)	$^{2}J(^{1}H,^{1}H)$	-12.6789
226		$^{1}J(^{13}C_{1},^{13}C_{2})$	54.8105
227		$^{1}J(^{13}C_{3},^{13}C_{1})$	69.9525
228		$^{2}J(^{13}C_{3},^{13}C_{2})$	1.4638
229		$^{2}J(^{15}N_{4},^{13}C_{1})$	2.767917
230		$^{1}J(^{15}N_{4},^{13}C_{2})$	-2.04648
231		$^{2}J(^{15}N_{4},^{13}C_{3})$	0.950928
232		$^{2}J(^{15}N_{5},^{13}C_{1})$	-5.89662
233		$^{2}J(^{15}N_{5},^{13}C_{2})$	-0.76296
234		$^{1}J(^{15}N_{5},^{13}C_{3})$	-16.3014
235		$^{1}J(^{15}N,^{15}N)$	-12.3632
236		$^{1}J(^{13}C_{1},^{1}H_{6})$	171.2373
237		$^{2}J(^{13}C_{2},^{1}H_{6})$	4.4881
238		$^{2}J(^{13}C3,^{1}H_{6})$	6.819
239		$^{3}J(^{15}N_{4},^{1}H_{6})$	-1.07213
240	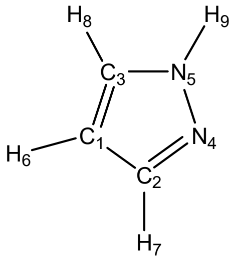	$^{3}J(^{15}N_{5},^{1}H_{6})$	-5.94628
241		$^{2}J(^{13}C_{1},^{1}H_{7})$	9.6743
242		$^{1}J(^{13}C_{2},^{1}H_{7})$	180.4903
243		$^{3}J(^{13}C_{3},^{1}H_{7})$	4.8384
244		$^{2}J(^{15}N_{4},^{1}H_{7})$	-11.838
245		$^{3}J(^{15}N_{5},^{1}H_{7})$	-9.09294
246		$^{3}J(^{1}H_{7},^{1}H_{6})$	1.8617
247	1*H*-pyrazole (**35**)	$^{2}J(^{13}C_{1},^{1}H_{8})$	5.9976
248		$^{3}J(^{13}C_{2},^{1}H_{8})$	8.0526
249		$^{1}J(^{13}C_{3},^{1}H_{8})$	179.0963
250		$^{3}J(^{15}N_{4},^{1}H_{8})$	0.431067
251		$^{2}J(^{15}N_{5},^{1}H_{8})$	-4.43832
252		$^{3}J(^{1}H_{8},^{1}H_{6})$	3.0747
253		$^{4}J(^{1}H_{8},^{1}H_{7})$	-0.2977
254		$^{3}J(^{13}C_{1},^{1}H_{9})$	5.3483
255		$^{3}J(^{13}C_{2},^{1}H_{9})$	10.1793
256		$^{2}J(^{13}C_{3},^{1}H_{9})$	8.7169
257		$^{2}J(^{15}N_{4},^{1}H_{9})$	-8.72444
258		$^{1}J(^{15}N,^{1}H)$	-110.893
259		$^{4}J(^{1}H_{9},^{1}H_{6})$	1.6419
260		$^{4}J(^{1}H_{9},^{1}H_{7})$	1.4857
261		$^{3}J(^{1}H_{9},^{1}H_{8})$	1.6276

262		$^{1}J(^{13}C,^{13}C)$	71.2163
263		$^{2}J(^{13}C_1,^{13}C_3)$	-4.4174
264		$^{2}J(^{13}C_3,^{13}C_2)$	9.0891
265		$^{1}J(^{15}N_4,^{13}C_1)$	1.280996
266		$^{2}J(^{15}N_4,^{13}C_2)$	2.337412
267		$^{1}J(^{15}N_4,^{13}C_3)$	-2.43743
268		$^{2}J(^{15}N_5,^{13}C_1)$	-6.37314
269		$^{1}J(^{15}N_5,^{13}C_2)$	-18.2176
270		$^{1}J(^{15}N_5,^{13}C_3)$	-13.9897
271		$^{2}J(^{15}N,^{15}N)$	-1.46595
272		$^{1}J(^{13}C_1,^{1}H_6)$	184.1781
273		$^{2}J(^{13}C_2,^{1}H_6)$	14.3688
274		$^{3}J(^{13}C_3,^{1}H_6)$	11.5221
275		$^{2}J(^{15}N_4,^{1}H_6)$	-9.57353
276	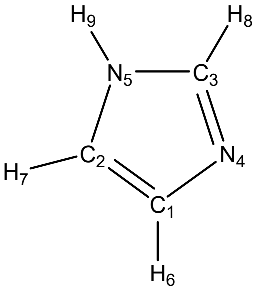	$^{3}J(^{15}N_5,^{1}H_6)$	-3.68476
277		$^{2}J(^{13}C_1,^{1}H_7)$	7.2651
278		$^{1}J(^{13}C_2,^{1}H_7)$	182.9566
279		$^{3}J(^{13}C_3,^{1}H_7)$	6.533
280		$^{3}J(^{15}N_4,^{1}H_7)$	-1.10902
281		$^{2}J(^{15}N_5,^{1}H_7)$	-4.53805
282		$^{3}J(^{1}H_7,^{1}H_6)$	2.2281
283	1*H*-imidazole (**36**)	$^{3}J(^{13}C_1,^{1}H_8)$	11.5174
284		$^{3}J(C_2,^{1}H_8)$	3.1406
285		$^{1}J(C_3,^{1}H_8)$	200.2012
286		$^{2}J(^{15}N_4,^{1}H_8)$	-10.8871
287		$^{2}J(^{15}N_5,^{1}H_8)$	-8.87004
288		$^{4}J(^{1}H_8,^{1}H_6)$	-0.1947
289		$^{4}J(^{1}H8,^{1}H_7)$	1.0648
290		$^{3}J(^{13}C_1,^{1}H_9)$	7.7344
291		$^{2}J(^{13}C_2,^{1}H_9)$	4.1495
292		$^{2}J(^{13}C_3,^{1}H_9)$	4.2473
293		$^{3}J(^{15}N_4,^{1}H_9)$	0.265401
294		$^{1}J(^{15}N,^{1}H)$	-100.193
295		$^{4}J(^{1}H_9,^{1}H_6)$	1.3797
296		$^{3}J(^{1}H_9,^{1}H_7)$	2.0075
297		$^{3}J(^{1}H_9,^{1}H_8)$	0.96
298		$^{1}J(^{13}C_1,^{13}C_2)$	57.1795
299		$^{2}J(^{13}C_2,^{13}C_3)$	-4.4836
300		$^{2}J(^{13}C_1,^{13}C_4)$	-4.0438
301	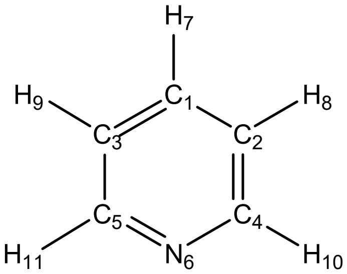	$^{1}J(^{13}C_2,^{13}C_4)$	57.659
302		$^{3}J(^{13}C_3,^{13}C_4)$	14.9352
303		$^{2}J(^{13}C_5,^{13}C_4)$	-7.4217
304		$^{3}J(^{15}N_6,^{13}C_1)$	-4.47114
305	pyridine (**37**)	$^{2}J(^{15}N_6,^{13}C_2)$	2.820801
306		$^{1}J(^{15}N,^{13}C)$	-1.71347

307		$^{1}J(^{13}C_{1},^{1}H_{7})$	155.4916
308		$^{2}J(^{13}C_{2},^{1}H_{7})$	-1.1781
309		$^{3}J(^{13}C_{4},^{1}H_{7})$	7.0525
310		$^{4}J(^{15}N,^{1}H)$	0.515653
311		$^{2}J(^{13}C_{1},^{1}H_{8})$	-1.0388
312		$^{1}J(^{13}C_{2},^{1}H_{8})$	157.9484
313		$^{3}J(^{13}C_{3},^{1}H_{8})$	6.8526
314		$^{2}J(^{13}C_{4},^{1}H_{8})$	1.1156
315		$^{4}J(^{13}C_{5},^{1}H_{8})$	-1.5972
316		$^{3}J(^{15}N,^{1}H)$	-1.6293
317		$^{3}J(^{1}H_{7},^{1}H_{8})$	8.207
318		$^{4}J(^{1}H_{8},^{1}H_{9})$	0.3967
319		$^{3}J(^{13}C_{1},^{1}H_{10})$	6.8841
320		$^{2}J(^{13}C_{2},^{1}H_{10})$	6.5525
321		$^{4}J(^{13}C_{3},^{1}H_{10})$	-2.4091
322		$^{1}J(^{13}C_{4},^{1}H_{10})$	172.3989
323		$^{3}J(^{13}C_{5},^{1}H_{10})$	11.5474
324		$^{2}J(^{15}N,^{1}H)$	-9.77356
325		$^{4}J(^{1}H_{7},^{1}H_{10})$	0.9359
326		$^{3}J(^{1}H_{8},^{1}H_{10})$	5.6541
327		$^{5}J(^{1}H,^{1}H)$	1.3202
328		$^{4}J(^{1}H_{10},^{1}H_{11})$	-1.0845
329		$^{2}J(^{15}N,^{13}C)$	-4.48896
330		$^{1}J(^{13}C_{2},^{13}C_{3})$	55.5863
331		$^{1}J(^{15}N,^{13}C)$	-17.1631
332		$^{1}J(^{13}C_{5},^{13}C_{3})$	71.235
333		$^{2}J(^{13}C_{2},^{13}C_{5})$	0.6876
334		$^{2}J(^{13}C_{4},^{13}C_{5})$	7.5509
335		$^{3}J(^{15}N,^{1}H)$	-5.69014
336	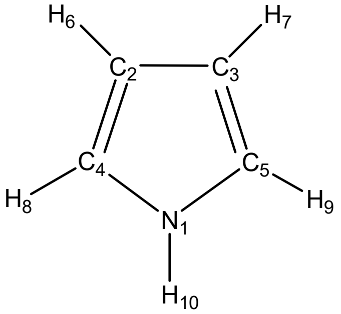	$^{1}J(^{13}C_{3},^{1}H_{7})$	165.8131
337		$^{2}J(^{13}C_{2},^{1}H_{7})$	2.6397
338		$^{2}J(^{13}C_{5},^{1}H_{7})$	6.0972
339		$^{3}J(^{13}C_{4},^{1}H_{7})$	7.4491
340		$^{3}J(^{1}H_{6},^{1}H_{7})$	3.5686
341		$^{2}J(^{15}N,^{1}H)$	-3.90134
342		$^{2}J(^{13}C_{3},^{1}H_{9})$	5.2941
343		$^{3}J(^{13}C_{2},^{1}H_{9})$	7.9135
344	1*H*-pyrrole (**38**)	$^{1}J(^{13}C_{5},^{1}H_{9})$	178.1733
345		$^{3}J(^{13}C_{4},^{1}H_{9})$	5.77
346		$^{3}J(^{1}H_{7},^{1}H_{9})$	3.6724
347		$^{4}J(^{1}H_{6},^{1}H_{9})$	0.4991
348		$^{4}J(^{1}H_{8},^{1}H_{9})$	1.8259
349		$^{1}J(^{15}N,^{1}H)$	-100.049
350		$^{3}J(^{13}C_{3},^{1}H_{10})$	7.0264
351		$^{2}J(^{13}C_{5},^{1}H_{10})$	3.2499

352		$^{4}J(^{1}H_{7},^{1}H_{10})$	2.1321
353		$^{3}J(^{1}H_{9},^{1}H_{10})$	2.7041
354		$^{1}J(^{13}C_{2},^{13}C_{1})$	73.7446
355		$^{2}J(^{13}C,^{13}C)$	-0.4015
356		$^{1}J(^{13}C_{2},^{13}C_{3})$	44.2753
357		$^{1}J(^{13}C_{1},^{1}H_{4})$	154.9528
358		$^{2}J(^{13}C_{2},^{1}H_{4})$	-3.0507
359		$^{3}J(^{13}C_{3},^{1}H_{4})$	12.1105
360		$^{1}J(^{13}C_{1},^{1}H_{5})$	151.4225
361		$^{2}J(^{13}C_{2},^{1}H_{5})$	-4.3324
362		$^{3}J(^{13}C_{3},^{1}H_{5})$	7.8438
363		$^{2}J(^{1}H_{4},^{1}H_{5})$	-1.1652
364		$^{2}J(^{13}C_{1},^{1}H_{6})$	-1.7073
365		$^{1}J(^{13}C_{2},^{1}H_{6})$	148.4333
366		$^{2}J(^{13}C_{3},^{1}H_{6})$	3.5678
367		$^{3}J(^{1}H_{4},^{1}H_{6})$	11.2759
368	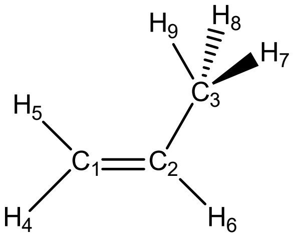	$^{3}J(^{1}H_{5},^{1}H_{6})$	16.8532
369		$^{3}J(^{13}C_{1},^{1}H_{7})$	6.31
370		$^{2}J(^{13}C_{2},^{1}H_{7})$	-8.8687
371	prop-1-ene (**39**)	$^{1}J(^{13}C_{3},^{1}H_{7})$	122.3328
372		$^{4}J(^{1}H_{4},^{1}H_{7})$	-3.1684
373		$^{4}J(^{1}H_{5},^{1}H_{7})$	-3.4246
374		$^{3}J(^{1}H_{6},^{1}H_{7})$	4.0646
375		$^{2}J(^{1}H_{7},^{1}H_{8})$	-20.2678
376		$^{3}J(^{13}C_{1},^{1}H_{9})$	6.3557
377		$^{2}J(^{13}C_{2},^{1}H_{9})$	-6.5164
378		$^{1}J(^{13}C_{3},^{1}H_{9})$	123.5561
379		$^{4}J(^{1}H_{4},^{1}H_{9})$	-0.6186
380		$^{4}J(^{1}H_{5},^{1}H_{9})$	-0.52
381		$^{3}J(^{1}H_{6},^{1}H_{9})$	12.0619
382		$^{2}J(^{1}H_{7},^{1}H_{9})$	-15.3403
383		$^{1}J(^{13}C_{1},^{13}C_{2})$	53.4486
384		$^{1}J(^{13}C_{1},^{13}C_{3})$	73.7591
385		$^{2}J(^{13}C_{2},^{13}C_{3})$	-0.2352
386		$^{2}J(^{13}C_{3},^{13}C_{4})$	4.0119
387		$^{1}J(^{13}C_{1},^{1}H_{6})$	169.8469
388		$^{2}J(^{13}C_{2},^{1}H_{6})$	2.5671
389	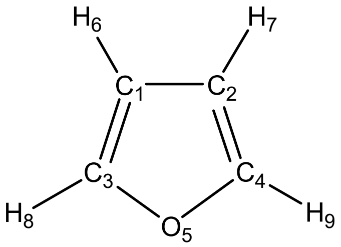	$^{2}J(^{13}C_{3},^{1}H_{6})$	7.7249
390		$^{3}J(^{13}C_{4},^{1}H_{6})$	6.8988
391		$^{3}J(^{1}H_{6},^{1}H_{7})$	3.1662
392		$^{2}J(^{13}C_{1},^{1}H_{8})$	11.1656
393	furan (**40**)	$^{3}J(^{13}C_{2},^{1}H_{8})$	6.4858
394		$^{1}J(^{13}C_{3},^{1}H_{8})$	195.5757
395		$^{3}J(^{13}C_{4},^{1}H_{8})$	6.5867
396		$^{3}J(^{1}H_{6},^{1}H_{8})$	2.9158

397		$^{4}J(^{1}H_{7},^{1}H_{8})$	-0.121
398		$^{4}J(^{1}H_{8},^{1}H_{9})$	1.1315
399		$^{1}J(^{19}F,^{13}C)$	-238.158
400		$^{2}J(^{19}F,^{13}C)$	21.1308
401		$^{1}J(^{13}C_{2},^{13}C_{3})$	73.1482
402		$^{2}J(^{13}C_{3},^{13}C_{4})$	2.2691
403		$^{3}J(^{19}F,^{13}C)$	4.6248
404		$^{2}J(^{13}C_{2},^{13}C_{6})$	-1.1511
405		$^{1}J(^{13}C_{3},^{13}C_{6})$	60.2895
406		$^{3}J(^{13}C_{4},^{13}C_{6})$	7.9455
407		$^{2}J(^{13}C_{5},^{13}C_{6})$	-2.8883
408		$^{4}J(^{19}F,^{13}C)$	5.4102
409		$^{3}J(^{13}C_{7},^{13}C_{2})$	11.6621
410		$^{2}J(^{13}C_{3},^{13}C_{7})$	-4.3043
411		$^{1}J(^{13}C_{6},^{13}C_{7})$	59.5515
412		$^{3}J(^{19}F,^{1}H)$	6.4432
413	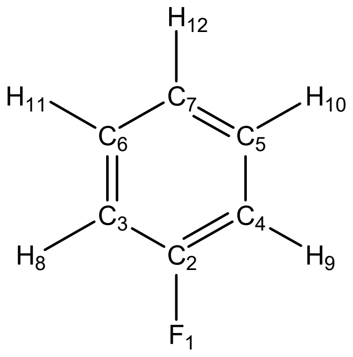	$^{2}J(^{13}C_{2},^{1}H_{8})$	-7.0958
414		$^{1}J(^{13}C_{3},^{1}H_{8})$	159.4452
415		$^{3}J(^{13}C_{4},^{1}H_{8})$	4.7347
416		$^{2}J(^{13}C_{6},^{1}H_{8})$	-2.221
417		$^{4}J(^{13}C_{5},^{1}H_{8})$	-1.541
418		$^{3}J(^{13}C_{7},^{1}H_{8})$	8.1224
419		$^{4}J(^{1}H_{8},^{1}H_{9})$	1.6337
420		$^{4}J(^{19}F,^{1}H)$	4.8594
421		$^{3}J(^{13}C_{2},^{1}H_{11})$	11.061
422	1-fluorobenzene (**41**)	$^{2}J(^{13}C_{3},^{1}H_{11})$	-0.9964
423		$^{4}J(^{13}C_{4},^{1}H_{11})$	-2.206
424		$^{1}J(^{13}C_{6},^{1}H_{11})$	156.1503
425		$^{3}J(^{13}C_{5},^{1}H_{11})$	9.2788
426		$^{2}J(^{13}C_{7},^{1}H_{11})$	-1.3075
427		$^{3}J(^{1}H_{8},^{1}H_{11})$	8.9805
428		$^{5}J(^{1}H_{9},^{1}H_{11})$	0.9214
429		$^{4}J(^{1}H_{10},^{1}H_{11})$	0.7517
430		$^{5}J(^{19}F,^{1}H)$	-1.4672
431		$^{4}J(^{13}C_{2},^{1}H_{12})$	-2.623
432		$^{3}J(^{13}C_{3},^{1}H_{12})$	8.5193
433		$^{2}J(^{13}C_{6},^{1}H_{12})$	-0.4203
434		$^{1}J(^{13}C_{7},^{1}H_{12})$	157.7166
435		$^{4}J(^{1}H_{8},^{1}H_{12})$	0.16
436		$^{3}\mathrm{J}(^{1}H_{11},^{1}H_{12})$	8.2133

Table S8. Symmetry independent values of SSCC (in Hz) in molecules of set 1 calculated at the SOPPA(CCSD) level with the pecJ-2-new(gen) basis set.

#	Molecule	Type of SSCC [1]	SSCC value
1	$H_2C{=}C{=}CH_2$ propa-1,2-diene (**1**)	$^{1}J(^{13}C,^{13}C)$	103.8268
2		$^{2}J(^{13}C,^{13}C)$	8.3927
3		$^{2}J(^{13}C,^{1}H)$	-5.7804
4		$^{1}J(^{13}C,^{1}H)$	166.7353
5		$^{3}J(^{13}C,^{1}H)$	7.8652
6		$^{2}J(^{1}H,^{1}H)$	-14.2184
7		$^{4}J(^{1}H,^{1}H)$	-8.8915
8	$F_2C{=}CF_2$ Perfluoroethene (**2**)	$^{1}J(^{13}C,^{13}C)$	196.2663
9		$^{1}J(^{13}C,^{19}F)$	-266.7213
10		$^{2}J(^{13}C,^{19}F)$	49.7446
11		$^{2}J(^{19}F,^{19}F)$	116.4673
12		$^{3}J_{cis}(^{19}F,^{19}F)$	79.3019
13		$^{3}J_{trans}(^{19}F,^{19}F)$	-116.1090
14	$HC{\equiv}CH$ ethyne (**3**)	$^{1}J(^{13}C,^{13}C)$	190.3929
15		$^{1}J(^{13}C,^{1}H)$	251.7153
16		$^{2}J(^{13}C,^{1}H)$	51.8825
17		$^{3}J(^{1}H,^{1}H)$	11.0851
18	$H_2C{=}CH_2$ ethene (**4**)	$^{1}J(^{13}C,^{13}C)$	71.2035
19		$^{1}J(^{13}C,^{1}H)$	154.1919
20		$^{2}J(^{13}C,^{1}H)$	-3.2181
21		$^{3}J_{cis}(^{1}H,^{1}H)$	12.3297
22		$^{2}J(^{1}H,^{1}H)$	0.0122
23		$^{3}J_{trans}(^{1}H,^{1}H)$	18.6166
24	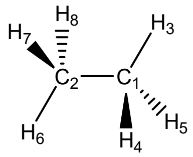 ethane (**5**)	$^{1}J(^{13}C,^{13}C)$	34.9722
25		$^{1}J(^{13}C,^{1}H)$	121.9911
26		$^{2}J(^{13}C,^{1}H)$	-5.0666
27		$^{3}J_{trans}(^{1}H_3,^{1}H_6)$	15.9111
28		$^{2}J(^{1}H,^{1}H)$	-14.4223
29		$^{3}J_{gauche}(^{1}H_4,^{1}H_6)$	3.7974
30	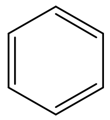 benzene (**6**)	$^{3}J(^{13}C,^{13}C)$	11.0043
31		$^{1}J(^{13}C,^{13}C)$	59.2313
32		$^{2}J(^{13}C,^{13}C)$	-3.4370
33		$^{1}J(^{13}C,^{1}H)$	155.1541
34		$^{4}J(^{13}C,^{1}H)$	-1.7422
35		$^{2}J(^{13}C,^{1}H)$	-0.1551
36		$^{3}J(^{13}C,^{1}H)$	7.8318
37		$^{3}J(^{1}H,^{1}H)$	8.1250
38		$^{4}J(^{1}H,^{1}H)$	0.7794
39		$^{5}J(^{1}H,^{1}H)$	1.1357
40	CF_4 perfluoromethane (**7**)	$^{1}J(^{19}F,^{13}C)$	-255.4745
41		$^{2}J(^{19}F,^{19}F)$	36.2598

42		$^{1}J(^{13}C,^{13}C)$	87.8624
43		$^{2}J(^{19}F,^{13}C)$	12.0368
44		$^{1}J(^{19}F,^{13}C)$	-259.6912
45		$^{1}J(^{13}C_1,^{1}H_4)$	158.9567
46		$^{2}J(^{13}C_2,^{1}H_4)$	6.6674
47	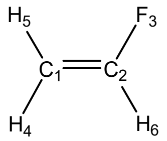	$^{3}J_{trans}(^{19}F_3,^{1}H_4)$	39.9599
48		$^{1}J(^{13}C_1,^{1}H_5)$	157.3900
49		$^{2}J(^{13}C_2,^{1}H_5)$	-10.0345
50		$^{3}J_{cis}(^{19}F_3,^{1}H_5)$	12.9486
51	fluoroethene (**8**)	$^{2}J(^{1}H,^{1}H)$	-4.8242
52		$^{2}J(^{13}C_1,^{1}H_6)$	13.1116
53		$^{1}J(^{13}C_2,^{1}H_6)$	192.6744
54		$^{2}J(^{19}F,^{1}H)$	82.6959
55		$^{3}J_{cis}(^{1}H_6,^{1}H_4)$	6.0566
56		$^{3}J_{trans}(^{1}H_6,^{1}H_5)$	12.9931
57		$^{1}J(^{19}F,^{13}C)$	-225.6415
58		$^{2}J(^{19}F,^{19}F)$	321.4617
59	CH_2F_2 difluoromethane (**9**)	$^{1}J(^{13}C,^{1}H)$	174.4776
60		$^{2}J(^{19}F,^{1}H)$	50.8196
61		$^{2}J(^{1}H,^{1}H)$	0.7425
62		$^{1}J(^{15}N,^{13}C)$	-4.8058
63		$^{1}J(^{13}C_1,^{1}H_3)$	171.5064
64		$^{2}J(^{15}N_2,^{1}H_3)$	-10.0788
65	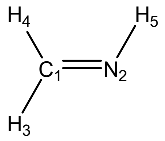	$^{1}J(^{13}C_1,^{1}H_4)$	155.6985
66		$^{2}J(^{15}N_2,^{1}H_4)$	3.9892
67		$^{2}J(^{1}H,^{1}H)$	16.5639
68	methanimine (**10**)	$^{2}J(^{13}C,^{1}H)$	-13.6081
69		$^{1}J(^{15}N,^{1}H)$	-53.7174
70		$^{3}J_{trans}(^{1}H_5,^{1}H_3)$	24.8214
71		$^{3}J_{cis}(^{1}H_5,^{1}H_4)$	18.4789
72		$^{1}J(^{13}C_1,^{13}C_2)$	191.0425
73		$^{2}J(^{13}C,^{13}C)$	13.0364
74		$^{1}J(^{13}C_2,^{13}C_3)$	70.8581
75		$^{1}J(^{13}C_1,^{1}H_4)$	250.5441
76	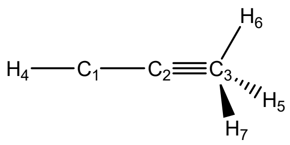	$^{2}J(^{13}C_2,^{1}H_4)$	51.7646
77		$^{3}J(^{13}C_3,^{1}H_4)$	4.2339
78		$^{3}J(^{13}C_1,^{1}H_6)$	3.7189
79	prop-1-yne (**11**)	$^{2}J(^{13}C_2,^{1}H_6)$	-11.8790
80		$^{1}J(^{13}C_3,^{1}H_6)$	128.2212
81		$^{4}J(^{1}H,^{1}H)$	-3.7390
82		$^{2}J(^{1}H,^{1}H)$	-17.7236
83		$^{1}J(^{13}C,^{13}C)$	41.9191
84		$^{1}J(^{13}C_1,^{1}H_4)$	165.4614
85		$^{2}J(^{13}C_2,^{1}H_4)$	26.7432
86		$^{2}J(^{13}C_1,^{1}H_5)$	-8.4455

87	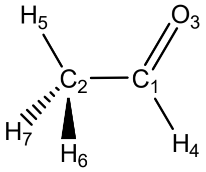 acetaldehyde (**12**)	$^{1}J(^{13}C_2,^{1}H_5)$	131.4321
88		$^{3}J_{trans}(^{1}H_5,^{1}H_4)$	8.2374
89		$^{2}J(^{13}C_1,^{1}H_6)$	-6.4977
90		$^{1}J(^{13}C_2,^{1}H_6)$	120.1341
91		$^{3}J_{gauche}(^{1}H_6,^{1}H_4)$	0.3273
92		$^{2}J(^{1}H_6,^{1}H_5)$	-13.7225
93		$^{2}J(^{1}H_6,^{1}H_7)$	-20.4786
94	$N\equiv C-CH_3$ acetonitrile (**13**)	$^{1}J(^{13}C,^{13}C)$	62.3775
95		$^{2}J(^{15}N,^{13}C)$	2.8749
96		$^{1}J(^{15}N,^{13}C)$	-18.9065
97		$^{1}J(^{13}C,^{1}H)$	131.5377
98		$^{2}J(^{13}C,^{1}H)$	-11.0707
99		$^{3}J(^{15}N,^{1}H)$	-1.6042
100		$^{2}J(^{1}H,^{1}H)$	-17.7783
101	H_3C-F fluoromethane (**14**)	$^{1}J(^{19}F,^{13}C)$	-162.7455
102		$^{1}J(^{13}C,^{1}H)$	143.4641
103		$^{2}J(^{19}F,^{1}H)$	49.3727
104		$^{2}J(^{1}H,^{1}H)$	-11.1619
105	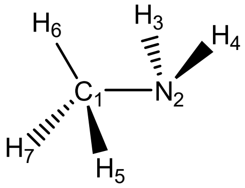 methanamine (**15**)	$^{1}J(^{15}N,^{13}C)$	-5.9968
106		$^{2}J(^{13}C,^{1}H)$	-3.7100
107		$^{1}J(^{15}N,^{1}H)$	-68.9672
108		$^{2}J(^{1}H_3,^{1}H_4)$	-11.2237
109		$^{1}J(^{13}C_1,^{1}H_5)$	129.6278
110		$^{2}J(^{15}N_2,^{1}H_5)$	-1.5753
111		$^{3}J(^{1}H_5,^{1}H_4)$	2.7448
112		$^{3}J(^{1}H_5,^{1}H_3)$	15.7803
113		$^{2}J(^{1}H_7,^{1}H_5)$	-16.0682
114		$^{1}J(^{13}C_1,^{1}H_6)$	126.6096
115		$^{2}J(^{15}N_2,^{1}H_6)$	1.0622
116		$^{3}J(^{1}H_6,^{1}H_4)$	2.1892
117		$^{2}J(^{1}H_6,^{1}H_5)$	-11.8506
118	CH_4 methane (**16**)	$^{1}J(^{13}C,^{1}H)$	122.1917
119		$^{2}J(^{1}H,^{1}H)$	-14.2113
120	CHF_3 fluoroform (**17**)	$^{1}J(^{13}C,^{1}H)$	223.8271
121		$^{1}J(^{19}F,^{13}C)$	-260.2856
122		$^{2}J(^{19}F,^{1}H)$	75.2664
123		$^{2}J(^{19}F,^{19}F)$	129.5025
124	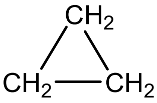 cyclopropane (**18**)	$^{1}J(^{13}C,^{13}C)$	13.4996
125		$^{1}J(^{13}C,^{1}H)$	156.9687
126		$^{2}J(^{13}C,^{1}H)$	-2.9961
127		$^{2}J(^{1}H,^{1}H)$	-6.2786
128		$^{3}J_{cis}(^{1}H,^{1}H)$	9.1466
129		$^{3}J_{trans}(^{1}H,^{1}H)$	5.1536
130	$F-C\equiv C-F$ 1,2-difluoroethyne (**19**)	$^{1}J(^{13}C,^{13}C)$	407.3621
131		$^{1}J(^{19}F,^{13}C)$	-282.7775

132		$^{2}J(^{19}\mathrm{F},^{13}\mathrm{C})$	38.5963
133		$^{3}J(^{19}\mathrm{F},^{19}\mathrm{F})$	-5.6611
134	F−C≡N<br>fluoroformonitrile (**20**)	$^{1}J(^{19}\mathrm{F},^{13}\mathrm{C})$	-406.3304
135		$^{1}J(^{15}\mathrm{N},^{13}\mathrm{C})$	-5.0620
136		$^{2}J(^{19}\mathrm{F},^{15}\mathrm{N})$	52.9014
137	$H_2C{=}CF_2$<br>1,1-difluoroethene (**21**)	$^{1}J(^{13}\mathrm{C},^{13}\mathrm{C})$	117.1183
138		$^{2}J(^{19}\mathrm{F},^{13}\mathrm{C})$	27.6701
139		$^{1}J(^{19}\mathrm{F},^{13}\mathrm{C})$	-285.9913
140		$^{2}J(^{19}\mathrm{F},^{19}\mathrm{F})$	21.3564
141		$^{1}J(^{13}\mathrm{C},^{1}\mathrm{H})$	164.1851
142		$^{2}J(^{13}\mathrm{C},^{1}\mathrm{H})$	-2.0456
143		$^{3}J_{cis}(^{19}\mathrm{F},^{1}\mathrm{H})$	-1.7299
144		$^{3}J_{trans}(^{19}\mathrm{F},^{1}\mathrm{H})$	27.1909
145		$^{2}J(^{1}\mathrm{H},^{1}\mathrm{H})$	-6.5065
146	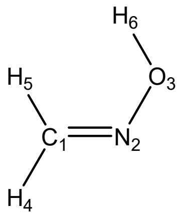<br>formaldehyde oxime (*Z*) (**22**)	$^{1}J(^{15}\mathrm{N},^{13}\mathrm{C})$	-3.4062
147		$^{1}J(^{13}\mathrm{C}_1,^{1}\mathrm{H}_4)$	183.9565
148		$^{2}J(^{15}\mathrm{N}_2,^{1}\mathrm{H}_4)$	-12.9121
149		$^{1}J(^{13}\mathrm{C}_1,^{1}\mathrm{H}_5)$	153.3398
150		$^{2}J(^{15}\mathrm{N}_2,^{1}\mathrm{H}_5)$	2.9681
151		$^{2}J(^{1}\mathrm{H},^{1}\mathrm{H})$	6.3141
152		$^{3}J(^{13}\mathrm{C},^{1}\mathrm{H})$	4.2107
153		$^{2}J(^{15}\mathrm{N}_2,^{1}\mathrm{H}_6)$	1.4291
154		$^{4}J(^{1}\mathrm{H}_6,^{1}\mathrm{H}_4)$	-1.3648
155		$^{4}J(^{1}\mathrm{H}_6,^{1}\mathrm{H}_5)$	1.3849
156	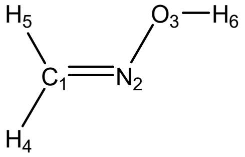<br>formaldehyde oxime (*E*) (**23**)	$^{1}J(^{15}\mathrm{N},^{13}\mathrm{C})$	-6.6429
157		$^{1}J(^{13}\mathrm{C}_1,^{1}\mathrm{H}_4)$	177.1224
158		$^{2}J(^{15}\mathrm{N}_2,^{1}\mathrm{H}_4)$	-13.7488
159		$^{1}J(^{13}\mathrm{C}_1,^{1}\mathrm{H}_5)$	162.6897
160		$^{2}J(^{15}\mathrm{N}_2,^{1}\mathrm{H}_5)$	2.9877
161		$^{2}J(^{1}\mathrm{H},^{1}\mathrm{H})$	8.0246
162		$^{3}J(^{13}\mathrm{C},^{1}\mathrm{H})$	10.9113
163		$^{2}J(^{15}\mathrm{N}_2,^{1}\mathrm{H}_6)$	-2.0619
164		$^{4}J(^{1}\mathrm{H}_6,^{1}\mathrm{H}_4)$	1.1820
165		$^{4}J(^{1}\mathrm{H}_6,^{1}\mathrm{H}_5)$	-0.8727
166	H−C≡C−F<br>fluoroethyne (**24**)	$^{1}J(^{13}\mathrm{C},^{13}\mathrm{C})$	274.3190
167		$^{2}J(^{19}\mathrm{F},^{13}\mathrm{C})$	22.7741
168		$^{1}J(^{19}\mathrm{F},^{13}\mathrm{C})$	-295.4904
169		$^{1}J(^{13}\mathrm{C},^{1}\mathrm{H})$	280.4193
170		$^{2}J(^{13}\mathrm{C},^{1}\mathrm{H})$	66.5220
171		$^{3}J(^{19}\mathrm{F},^{1}\mathrm{H})$	10.9205
172	H−C≡N<br>hydrogen cyanide (**25**)	$^{1}J(^{15}\mathrm{N},^{13}\mathrm{C})$	-19.5175
173		$^{1}J(^{13}\mathrm{C},^{1}\mathrm{H})$	262.7318
174		$^{2}J(^{15}\mathrm{N},^{1}\mathrm{H})$	-8.7531
175		$^{1}J(^{13}\mathrm{C},^{13}\mathrm{C})$	144.7616
176		$^{2}J(^{19}\mathrm{F}_3,^{13}\mathrm{C}_1)$	64.7026

177		$^{1}J(^{19}F_3,^{13}C_2)$	-272.5736
178		$^{1}J(^{19}F_4,^{13}C_1)$	-233.4617
179		$^{2}J(^{19}F_4,^{13}C_2)$	36.4966
180		$^{3}J_{trans}(^{19}F_3,^{19}F_4)$	-123.0152
181		$^{2}J(^{19}F_5,^{13}C_1)$	20.0612
182	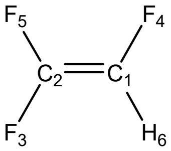	$^{1}J(^{19}F_5,^{13}C_2)$	-284.8092
183		$^{2}J(^{19}F,^{19}F)$	72.3362
184		$^{3}J_{cis}(^{19}F_4,^{19}F_5)$	41.0533
185	1,1,2-trifluoroethene (**26**)	$^{1}J(^{13}C,^{1}H)$	203.7921
186		$^{2}J(^{13}C,^{1}H)$	14.3740
187		$^{3}J_{cis}(^{19}F_3,^{1}H_6)$	-5.7806
188		$^{2}J(^{19}F,^{1}H)$	73.2840
189		$^{3}J_{trans}(^{19}F_5,^{1}H_6)$	7.9316
190		$^{1}J(^{13}C,^{13}C)$	101.3924
191		$^{1}J(^{19}F,^{13}C)$	-252.9959
192		$^{2}J(^{19}F,^{13}C)$	9.5987
193	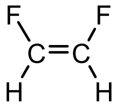	$^{3}J(^{19}F,^{19}F)$	-10.7295
194		$^{1}J(^{13}C,^{1}H)$	196.6006
195		$^{2}J(^{13}C,^{1}H)$	22.8755
196	(*Z*)-1,2-difluoroethene (**27**)	$^{2}J(^{19}F,^{1}H)$	73.3288
197		$^{3}J(^{19}F,^{1}H)$	13.5924
198		$^{3}J(^{1}H,^{1}H)$	3.5307
199		$^{1}J(^{13}C,^{13}C)$	114.9484
200		$^{1}J(^{19}F,^{13}C)$	-241.0432
201		$^{2}J(^{19}F,^{13}C)$	49.4383
202	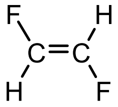	$^{3}J(^{19}F,^{19}F)$	-136.9145
203		$^{2}J(^{13}C,^{1}H)$	4.6147
204	(*E*)-1,2-difluoroethene (**28**)	$^{1}J(^{13}C,^{1}H)$	195.3913
205		$^{3}J(^{19}F,^{1}H)$	0.0947
206		$^{2}J(^{19}F,^{1}H)$	76.7571
207		$^{3}J(^{1}H,^{1}H)$	10.1425
208	HF<br>hydrogen fluoride (**29**)	$^{1}J(^{19}F,^{1}H)$	537.8127
209		$^{1}J(^{15}N,^{15}N)$	-21.3512
210	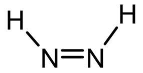	$^{1}J(^{15}N,^{1}H)$	-38.4233
211	diazene (*Z*) (**31**)	$^{2}J(^{15}N,^{1}H)$	1.2879
212		$^{3}J(^{1}H,^{1}H)$	38.7271
213		$^{1}J(^{15}N,^{15}N)$	-21.1357
214	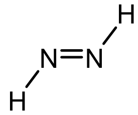	$^{2}J(^{15}N,^{1}H)$	-0.6001
215		$^{1}J(^{15}N,^{1}H)$	-49.0840
216	diazene (*E*) (**30**)	$^{3}J(^{1}H,^{1}H)$	38.2052
217		$^{1}J(^{15}N,^{15}N)$	0.7804
218		$^{1}J(^{15}N,^{1}H)$	-62.6747
219		$^{2}J(^{15}N,^{1}H)$	-1.7062
220		$^{2}J(^{1}H,^{1}H)$	-15.8593

221	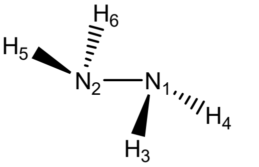	$^{3}J(^{1}H_5,^{1}H_3)$	1.5256
222	hydrazine (**32**)	$^{3}J(^{1}H_4,^{1}H_5)$	13.5625
223	N_2<br>molecular nitrogen (**33**)	$^{1}J(^{15}N,^{15}N)$	-2.80794
224	NH_3	$^{1}J(^{15}N,^{1}H)$	-65.2489
225	ammonia (**34**)	$^{2}J(^{1}H,^{1}H)$	-11.7418
226		$^{1}J(^{13}C_1,^{13}C_2)$	54.8311
227		$^{1}J(^{13}C_3,^{13}C_1)$	69.719
228		$^{2}J(^{13}C_3,^{13}C_2)$	1.861
229		$^{2}J(^{15}N_4,^{13}C_1)$	3.001616
230		$^{1}J(^{15}N_4,^{13}C_2)$	-2.6759
231		$^{2}J(^{15}N_4,^{13}C_3)$	1.039021
232		$^{2}J(^{15}N_5,^{13}C_1)$	-6.06285
233		$^{2}J(^{15}N_5,^{13}C_2)$	-0.7328
234		$^{1}J(^{15}N_5,^{13}C_3)$	-16.6234
235		$^{1}J(^{15}N,^{15}N)$	-12.68
236		$^{1}J(^{13}C_1,^{1}H_6)$	172.307
237		$^{2}J(^{13}C_2,^{1}H_6)$	5.1322
238		$^{2}J(^{13}C3,^{1}H_6)$	7.6344
239		$^{3}J(^{15}N_4,^{1}H_6)$	-1.1936
240	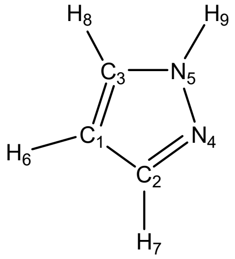	$^{3}J(^{15}N_5,^{1}H_6)$	-6.13411
241		$^{2}J(^{13}C_1,^{1}H_7)$	10.5664
242		$^{1}J(^{13}C_2,^{1}H_7)$	181.794
243		$^{3}J(^{13}C_3,^{1}H_7)$	4.771
244		$^{2}J(^{15}N_4,^{1}H_7)$	-12.6998
245		$^{3}J(^{15}N_5,^{1}H_7)$	-9.37574
246		$^{3}J(^{1}H_7,^{1}H_6)$	1.8593
247	1*H*-pyrazole (**35**)	$^{2}J(^{13}C_1,^{1}H_8)$	6.8813
248		$^{3}J(^{13}C_2,^{1}H_8)$	7.9791
249		$^{1}J(^{13}C_3,^{1}H_8)$	180.2588
250		$^{3}J(^{15}N_4,^{1}H_8)$	0.24955
251		$^{2}J(^{15}N_5,^{1}H_8)$	-4.78676
252		$^{3}J(^{1}H_8,^{1}H_6)$	3.0554
253		$^{4}J(^{1}H_8,^{1}H_7)$	0.1318
254		$^{3}J(^{13}C_1,^{1}H_9)$	5.5143
255		$^{3}J(^{13}C_2,^{1}H_9)$	10.3646
256		$^{2}J(^{13}C_3,^{1}H_9)$	9.2056
257		$^{2}J(^{15}N_4,^{1}H_9)$	-9.20362
258		$^{1}J(^{15}N,^{1}H)$	-113.366
259		$^{4}J(^{1}H_9,^{1}H_6)$	1.9994
260		$^{4}J(^{1}H_9,^{1}H_7)$	1.8875
261		$^{3}J(^{1}H_9,^{1}H_8)$	1.6766

262		$^{1}J(^{13}C,^{13}C)$	71.4399
263		$^{2}J(^{13}C_1,^{13}C_3)$	-4.3401
264		$^{2}J(^{13}C_3,^{13}C_2)$	9.104
265		$^{1}J(^{15}N_4,^{13}C_1)$	1.039021
266		$^{2}J(^{15}N_4,^{13}C_2)$	2.549087
267		$^{1}J(^{15}N_4,^{13}C_3)$	-3.18285
268		$^{2}J(^{15}N_5,^{13}C_1)$	-6.60025
269		$^{1}J(^{15}N_5,^{13}C_2)$	-18.1482
270		$^{1}J(^{15}N_5,^{13}C_3)$	-14.2349
271		$^{2}J(^{15}N,^{15}N)$	-1.62475
272		$^{1}J(^{13}C_1,^{1}H_6)$	185.5911
273		$^{2}J(^{13}C_2,^{1}H_6)$	15.5388
274		$^{3}J(^{13}C_3,^{1}H_6)$	11.625
275		$^{2}J(^{15}N_4,^{1}H_6)$	-10.4113
276	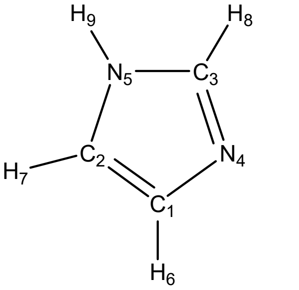	$^{3}J(^{15}N_5,^{1}H_6)$	-3.80006
277		$^{2}J(^{13}C_1,^{1}H_7)$	8.1861
278		$^{1}J(^{13}C_2,^{1}H_7)$	183.9969
279		$^{3}J(^{13}C_3,^{1}H_7)$	6.8474
280		$^{3}J(^{15}N_4,^{1}H_7)$	-1.21212
281		$^{2}J(^{15}N_5,^{1}H_7)$	-4.91385
282		$^{3}J(^{1}H_7,^{1}H_6)$	2.1373
283	1*H*-imidazole (**36**)	$^{3}J(^{13}C_1,^{1}H_8)$	11.4544
284		$^{3}J(C_2,^{1}H_8)$	3.3407
285		$^{1}J(C_3,^{1}H_8)$	201.7123
286		$^{2}J(^{15}N_4,^{1}H_8)$	-11.8916
287		$^{2}J(^{15}N_5,^{1}H_8)$	-9.45247
288		$^{4}J(^{1}H_8,^{1}H_6)$	0.2487
289		$^{4}J(^{1}H8,^{1}H_7)$	1.3605
290		$^{3}J(^{13}C_1,^{1}H_9)$	7.9306
291		$^{2}J(^{13}C_2,^{1}H_9)$	4.4841
292		$^{2}J(^{13}C_3,^{1}H_9)$	4.6616
293		$^{3}J(^{15}N_4,^{1}H_9)$	0.099596
294		$^{1}J(^{15}N,^{1}H)$	-102.48
295		$^{4}J(^{1}H_9,^{1}H_6)$	1.7313
296		$^{3}J(^{1}H_9,^{1}H_7)$	2.0821
297		$^{3}J(^{1}H_9,^{1}H_8)$	0.9832
298		$^{1}J(^{13}C_1,^{13}C_2)$	57.2064
299	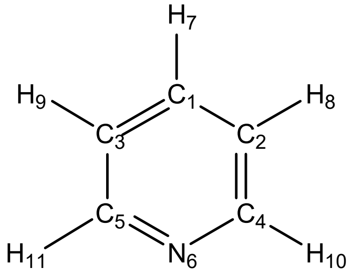	$^{2}J(^{13}C_2,^{13}C_3)$	-4.0792
300		$^{2}J(^{13}C_1,^{13}C_4)$	-3.6617
301		$^{1}J(^{13}C_2,^{13}C_4)$	57.8471
302		$^{3}J(^{13}C_3,^{13}C_4)$	15.1396
303		$^{2}J(^{13}C_5,^{13}C_4)$	-7.2092
304		$^{3}J(^{15}N_6,^{13}C_1)$	-4.642
305	pyridine (**37**)	$^{2}J(^{15}N_6,^{13}C_2)$	3.077225
306		$^{1}J(^{15}N,^{13}C)$	-2.26769

307		$^{1}J(^{13}C_1,^{1}H_7)$	156.183
308		$^{2}J(^{13}C_2,^{1}H_7)$	-0.4357
309		$^{3}J(^{13}C_4,^{1}H_7)$	6.8986
310		$^{4}J(^{15}N,^{1}H)$	0.550161
311		$^{2}J(^{13}C_1,^{1}H_8)$	-0.3833
312		$^{1}J(^{13}C_2,^{1}H_8)$	158.798
313		$^{3}J(^{13}C_3,^{1}H_8)$	6.7359
314		$^{2}J(^{13}C_4,^{1}H_8)$	1.8462
315		$^{4}J(^{13}C_5,^{1}H_8)$	-1.3111
316		$^{3}J(^{15}N,^{1}H)$	-1.74082
317		$^{3}J(^{1}H_7,^{1}H_8)$	8.1134
318		$^{4}J(^{1}H_8,^{1}H_9)$	0.7778
319		$^{3}J(^{13}C_1,^{1}H_{10})$	6.7273
320		$^{2}J(^{13}C_2,^{1}H_{10})$	7.4366
321		$^{4}J(^{13}C_3,^{1}H_{10})$	-2.1116
322		$^{1}J(^{13}C_4,^{1}H_{10})$	173.365
323		$^{3}J(^{13}C_5,^{1}H_{10})$	11.45
324		$^{2}J(^{15}N,^{1}H)$	-10.5736
325		$^{4}J(^{1}H_7,^{1}H_{10})$	1.3167
326		$^{3}J(^{1}H_8,^{1}H_{10})$	5.4834
327		$^{5}J(^{1}H,^{1}H)$	1.3788
328		$^{4}J(^{1}H_{10},^{1}H_{11})$	-0.7188
329		$^{2}J(^{15}N,^{13}C)$	-4.62937
330		$^{1}J(^{13}C_2,^{13}C_3)$	55.2009
331		$^{1}J(^{15}N,^{13}C)$	-17.2313
332		$^{1}J(^{13}C_5,^{13}C_3)$	71.1721
333		$^{2}J(^{13}C_2,^{13}C_5)$	1.1245
334		$^{2}J(^{13}C_4,^{13}C_5)$	7.5358
335		$^{3}J(^{15}N,^{1}H)$	-5.83532
336	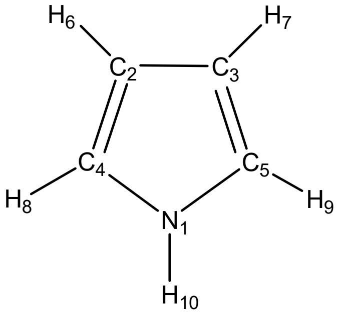	$^{1}J(^{13}C_3,^{1}H_7)$	167.0404
337		$^{2}J(^{13}C_2,^{1}H_7)$	3.3217
338		$^{2}J(^{13}C_5,^{1}H_7)$	6.995
339		$^{3}J(^{13}C_4,^{1}H_7)$	7.4139
340		$^{3}J(^{1}H_6,^{1}H_7)$	3.6289
341		$^{2}J(^{15}N,^{1}H)$	-4.26213
342		$^{2}J(^{13}C_3,^{1}H_9)$	6.2349
343		$^{3}J(^{13}C_2,^{1}H_9)$	7.7202
344	1*H*-pyrrole (**38**)	$^{1}J(^{13}C_5,^{1}H_9)$	179.404
345		$^{3}J(^{13}C_4,^{1}H_9)$	6.01
346		$^{3}J(^{1}H_7,^{1}H_9)$	3.6547
347		$^{4}J(^{1}H_6,^{1}H_9)$	0.9606
348		$^{4}J(^{1}H_8,^{1}H_9)$	2.1421
349		$^{1}J(^{15}N,^{1}H)$	-102.311
350		$^{3}J(^{13}C_3,^{1}H_{10})$	7.1653
351		$^{2}J(^{13}C_5,^{1}H_{10})$	3.6254

352		$^{4}J(^{1}H_{7},^{1}H_{10})$	2.5043
353		$^{3}J(^{1}H_{9},^{1}H_{10})$	2.7878
354		$^{1}J(^{13}C_{2},^{13}C_{1})$	73.6628
355		$^{2}J(^{13}C,^{13}C)$	-0.0726
356		$^{1}J(^{13}C_{2},^{13}C_{3})$	43.9144
357		$^{1}J(^{13}C_{1},^{1}H_{4})$	155.2445
358		$^{2}J(^{13}C_{2},^{1}H_{4})$	-2.0157
359		$^{3}J(^{13}C_{3},^{1}H_{4})$	12.0325
360		$^{1}J(^{13}C_{1},^{1}H_{5})$	151.5175
361		$^{2}J(^{13}C_{2},^{1}H_{5})$	-3.5152
362		$^{3}J(^{13}C_{3},^{1}H_{5})$	7.7576
363		$^{2}J(^{1}H_{4},^{1}H_{5})$	-0.175
364		$^{2}J(^{13}C_{1},^{1}H_{6})$	-0.8228
365		$^{1}J(^{13}C_{2},^{1}H_{6})$	148.9947
366		$^{2}J(^{13}C_{3},^{1}H_{6})$	4.0116
367	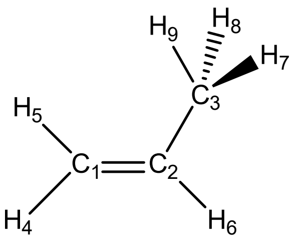	$^{3}J(^{1}H_{4},^{1}H_{6})$	10.9945
368		$^{3}J(^{1}H_{5},^{1}H_{6})$	16.5977
369		$^{3}J(^{13}C_{1},^{1}H_{7})$	6.2423
370		$^{2}J(^{13}C_{2},^{1}H_{7})$	-8.2944
371	prop-1-ene (**39**)	$^{1}J(^{13}C_{3},^{1}H_{7})$	122.6036
372		$^{4}J(^{1}H_{4},^{1}H_{7})$	-2.7761
373		$^{4}J(^{1}H_{5},^{1}H_{7})$	-3.0296
374		$^{3}J(^{1}H_{6},^{1}H_{7})$	3.9451
375		$^{2}J(^{1}H_{7},^{1}H_{8})$	-19.4684
376		$^{3}J(^{13}C_{1},^{1}H_{9})$	6.4266
377		$^{2}J(^{13}C_{2},^{1}H_{9})$	-6.1861
378		$^{1}J(^{13}C_{3},^{1}H_{9})$	123.8065
379		$^{4}J(^{1}H_{4},^{1}H_{9})$	-0.3832
380		$^{4}J(^{1}H_{5},^{1}H_{9})$	-0.5828
381		$^{3}J(^{1}H_{6},^{1}H_{9})$	12.2237
382		$^{2}J(^{1}H_{7},^{1}H_{9})$	-14.4592
383		$^{1}J(^{13}C_{1},^{13}C_{2})$	52.9638
384		$^{1}J(^{13}C_{1},^{13}C_{3})$	74.3612
385		$^{2}J(^{13}C_{2},^{13}C_{3})$	0.0824
386		$^{2}J(^{13}C_{3},^{13}C_{4})$	4.0148
387		$^{1}J(^{13}C_{1},^{1}H_{6})$	170.9151
388	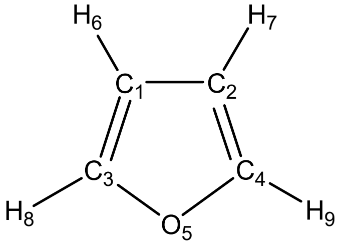	$^{2}J(^{13}C_{2},^{1}H_{6})$	3.1503
389		$^{2}J(^{13}C_{3},^{1}H_{6})$	8.8656
390		$^{3}J(^{13}C_{4},^{1}H_{6})$	6.8414
391		$^{3}J(^{1}H_{6},^{1}H_{7})$	3.2446
392		$^{2}J(^{13}C_{1},^{1}H_{8})$	12.1845
393	furan (**40**)	$^{3}J(^{13}C_{2},^{1}H_{8})$	6.3704
394		$^{1}J(^{13}C_{3},^{1}H_{8})$	196.7902
395		$^{3}J(^{13}C_{4},^{1}H_{8})$	6.725
396		$^{3}J(^{1}H_{6},^{1}H_{8})$	2.8175

397		$^{4}J(^{1}H_{7},^{1}H_{8})$	0.3245
398		$^{4}J(^{1}H_{8},^{1}H_{9})$	1.5853
399		$^{1}J(^{19}F,^{13}C)$	-248.022
400		$^{2}J(^{19}F,^{13}C)$	21.9328
401		$^{1}J(^{13}C_{2},^{13}C_{3})$	73.5479
402		$^{2}J(^{13}C_{3},^{13}C_{4})$	2.9665
403		$^{3}J(^{19}F,^{13}C)$	5.0639
404		$^{2}J(^{13}C_{2},^{13}C_{6})$	-0.5319
405		$^{1}J(^{13}C_{3},^{13}C_{6})$	60.2764
406		$^{3}J(^{13}C_{4},^{13}C_{6})$	7.7415
407		$^{2}J(^{13}C_{5},^{13}C_{6})$	-2.4025
408		$^{4}J(^{19}F,^{13}C)$	5.553
409		$^{3}J(^{13}C_{7},^{13}C_{2})$	11.4854
410		$^{2}J(^{13}C_{3},^{13}C_{7})$	-3.8881
411		$^{1}J(^{13}C_{6},^{13}C_{7})$	59.636
412		$^{3}J(^{19}F,^{1}H)$	6.2109
413	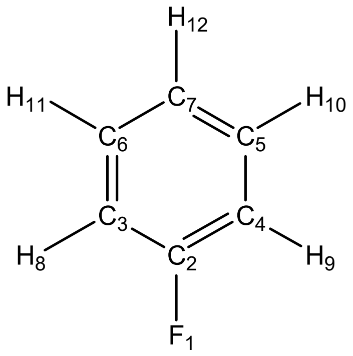	$^{2}J(^{13}C_{2},^{1}H_{8})$	-6.3524
414		$^{1}J(^{13}C_{3},^{1}H_{8})$	159.4725
415		$^{3}J(^{13}C_{4},^{1}H_{8})$	4.532
416		$^{2}J(^{13}C_{6},^{1}H_{8})$	-1.4902
417		$^{4}J(^{13}C_{5},^{1}H_{8})$	-1.2642
418		$^{3}J(^{13}C_{7},^{1}H_{8})$	7.905
419		$^{4}J(^{1}H_{8},^{1}H_{9})$	2.0481
420		$^{4}J(^{19}F,^{1}H)$	4.9431
421		$^{3}J(^{13}C_{2},^{1}H_{11})$	10.7776
422	1-fluorobenzene (**41**)	$^{2}J(^{13}C_{3},^{1}H_{11})$	-0.2932
423		$^{4}J(^{13}C_{4},^{1}H_{11})$	-1.9363
424		$^{1}J(^{13}C_{6},^{1}H_{11})$	156.4977
425		$^{3}J(^{13}C_{5},^{1}H_{11})$	9.0938
426		$^{2}J(^{13}C_{7},^{1}H_{11})$	-0.5622
427		$^{3}J(^{1}H_{8},^{1}H_{11})$	8.8459
428		$^{5}J(^{1}H_{9},^{1}H_{11})$	0.9513
429		$^{4}J(^{1}H_{10},^{1}H_{11})$	1.1427
430		$^{5}J(^{19}F,^{1}H)$	-1.3476
431		$^{4}J(^{13}C_{2},^{1}H_{12})$	-2.2951
432		$^{3}J(^{13}C_{3},^{1}H_{12})$	8.3271
433		$^{2}J(^{13}C_{6},^{1}H_{12})$	0.3301
434		$^{1}J(^{13}C_{7},^{1}H_{12})$	158.2261
435		$^{4}J(^{1}H_{8},^{1}H_{12})$	0.5434
436		$^{3}\mathrm{J}(^{1}H_{11},^{1}H_{12})$	8.0967

Table S9. Symmetry independent values of SSCC (in Hz) in molecules of set 1 calculated at the SOPPA(CCSD) level with the pecJ-2-new(seg) basis set.

#	Molecule	Type of SSCC [1]	SSCC value
1	$H_2C{=}C{=}CH_2$ propa-1,2-diene (**1**)	$^{1}J(^{13}C,^{13}C)$	103.8065
2		$^{2}J(^{13}C,^{13}C)$	8.4355
3		$^{2}J(^{13}C,^{1}H)$	-5.7134
4		$^{1}J(^{13}C,^{1}H)$	166.4475
5		$^{3}J(^{13}C,^{1}H)$	7.8462
6		$^{2}J(^{1}H,^{1}H)$	-14.1955
7		$^{4}J(^{1}H,^{1}H)$	-8.8674
8	$F_2C{=}CF_2$ Perfluoroethene (**2**)	$^{1}J(^{13}C,^{13}C)$	196.1927
9		$^{1}J(^{13}C,^{19}F)$	-264.3260
10		$^{2}J(^{13}C,^{19}F)$	49.5141
11		$^{2}J(^{19}F,^{19}F)$	116.7037
12		$^{3}J_{cis}(^{19}F,^{19}F)$	79.6018
13		$^{3}J_{trans}(^{19}F,^{19}F)$	-116.5888
14	$HC{\equiv}CH$ ethyne (**3**)	$^{1}J(^{13}C,^{13}C)$	190.3744
15		$^{1}J(^{13}C,^{1}H)$	251.3076
16		$^{2}J(^{13}C,^{1}H)$	51.9229
17		$^{3}J(^{1}H,^{1}H)$	11.0562
18	$H_2C{=}CH_2$ ethene (**4**)	$^{1}J(^{13}C,^{13}C)$	71.2056
19		$^{1}J(^{13}C,^{1}H)$	153.9244
20		$^{2}J(^{13}C,^{1}H)$	-3.1971
21		$^{3}J_{cis}(^{1}H,^{1}H)$	12.3217
22		$^{2}J(^{1}H,^{1}H)$	0.0401
23		$^{3}J_{trans}(^{1}H,^{1}H)$	18.6115
24	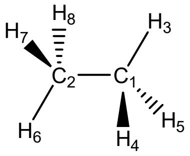 ethane (**5**)	$^{1}J(^{13}C,^{13}C)$	34.8874
25		$^{1}J(^{13}C,^{1}H)$	121.7942
26		$^{2}J(^{13}C,^{1}H)$	-5.0461
27		$^{3}J_{trans}(^{1}H_3,^{1}H_6)$	15.9151
28		$^{2}J(^{1}H,^{1}H)$	-14.4138
29		$^{3}J_{gauche}(^{1}H_4,^{1}H_6)$	3.7946
30	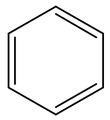 benzene (**6**)	$^{3}J(^{13}C,^{13}C)$	10.9792
31		$^{1}J(^{13}C,^{13}C)$	59.1993
32		$^{2}J(^{13}C,^{13}C)$	-3.3890
33		$^{1}J(^{13}C,^{1}H)$	154.8863
34		$^{4}J(^{13}C,^{1}H)$	-1.7284
35		$^{2}J(^{13}C,^{1}H)$	-0.1369
36		$^{3}J(^{13}C,^{1}H)$	7.8254
37		$^{3}J(^{1}H,^{1}H)$	8.1217
38		$^{4}J(^{1}H,^{1}H)$	0.7878
39		$^{5}J(^{1}H,^{1}H)$	1.1331
40	CF_4 perfluoromethane (**7**)	$^{1}J(^{19}F,^{13}C)$	-253.5120
41		$^{2}J(^{19}F,^{19}F)$	35.4309

42		$^{1}J(^{13}C,^{13}C)$	87.8726
43		$^{2}J(^{19}F,^{13}C)$	12.0029
44		$^{1}J(^{19}F,^{13}C)$	-257.6410
45		$^{1}J(^{13}C_1,^{1}H_4)$	158.6633
46		$^{2}J(^{13}C_2,^{1}H_4)$	6.6822
47		$^{3}J_{trans}(^{19}F_3,^{1}H_4)$	39.7576
48	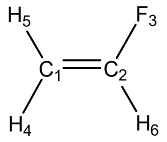	$^{1}J(^{13}C_1,^{1}H_5)$	157.1208
49		$^{2}J(^{13}C_2,^{1}H_5)$	-9.9940
50		$^{3}J_{cis}(^{19}F_3,^{1}H_5)$	12.9056
51	fluoroethene (**8**)	$^{2}J(^{1}H,^{1}H)$	-4.7986
52		$^{2}J(^{13}C_1,^{1}H_6)$	13.1249
53		$^{1}J(^{13}C_2,^{1}H_6)$	192.3714
54		$^{2}J(^{19}F,^{1}H)$	82.3323
55		$^{3}J_{cis}(^{1}H_6,^{1}H_4)$	6.0465
56		$^{3}J_{trans}(^{1}H_6,^{1}H_5)$	12.9880
57		$^{1}J(^{19}F,^{13}C)$	-224.1219
58		$^{2}J(^{19}F,^{19}F)$	321.1600
59	CH_2F_2 difluoromethane (**9**)	$^{1}J(^{13}C,^{1}H)$	174.1927
60		$^{2}J(^{19}F,^{1}H)$	50.5584
61		$^{2}J(^{1}H,^{1}H)$	0.7757
62		$^{1}J(^{15}N,^{13}C)$	-3.7468
63		$^{1}J(^{13}C_1,^{1}H_3)$	171.2921
64		$^{2}J(^{15}N_2,^{1}H_3)$	-9.4435
65		$^{1}J(^{13}C_1,^{1}H_4)$	155.4846
66	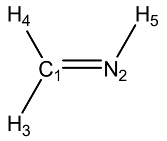	$^{2}J(^{15}N_2,^{1}H_4)$	3.8807
67		$^{2}J(^{1}H,^{1}H)$	16.6077
68	methanimine (**10**)	$^{2}J(^{13}C,^{1}H)$	-13.5958
69		$^{1}J(^{15}N,^{1}H)$	-50.3041
70		$^{3}J_{trans}(^{1}H_5,^{1}H_3)$	24.7968
71		$^{3}J_{cis}(^{1}H_5,^{1}H_4)$	18.4598
72		$^{1}J(^{13}C_1,^{13}C_2)$	191.0405
73		$^{2}J(^{13}C,^{13}C)$	13.0817
74		$^{1}J(^{13}C_2,^{13}C_3)$	70.7498
75		$^{1}J(^{13}C_1,^{1}H_4)$	250.1285
76		$^{2}J(^{13}C_2,^{1}H_4)$	51.8175
77	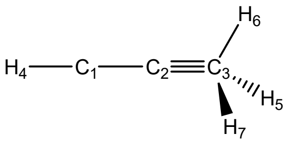	$^{3}J(^{13}C_3,^{1}H_4)$	4.2195
78		$^{3}J(^{13}C_1,^{1}H_6)$	3.6993
79	prop-1-yne (**11**)	$^{2}J(^{13}C_2,^{1}H_6)$	-11.8332
80		$^{1}J(^{13}C_3,^{1}H_6)$	128.0209
81		$^{4}J(^{1}H,^{1}H)$	-3.7280
82		$^{2}J(^{1}H,^{1}H)$	-17.7166
83		$^{1}J(^{13}C,^{13}C)$	41.8597
84		$^{1}J(^{13}C_1,^{1}H_4)$	165.2764
85		$^{2}J(^{13}C_2,^{1}H_4)$	26.7551
86		$^{2}J(^{13}C_1,^{1}H_5)$	-8.4246

87	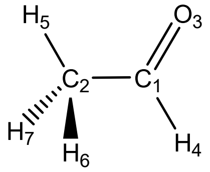 acetaldehyde (**12**)	$^{1}J(^{13}C_2,^{1}H_5)$	131.2230
88		$^{3}J_{trans}(^{1}H_5,^{1}H_4)$	8.2393
89		$^{2}J(^{13}C_1,^{1}H_6)$	-6.4814
90		$^{1}J(^{13}C_2,^{1}H_6)$	119.9374
91		$^{3}J_{gauche}(^{1}H_6,^{1}H_4)$	0.3244
92		$^{2}J(^{1}H_6,^{1}H_5)$	-13.7123
93		$^{2}J(^{1}H_6,^{1}H_7)$	-20.4721
94	N≡C−CH_3 acetonitrile (**13**)	$^{1}J(^{13}C,^{13}C)$	62.3274
95		$^{2}J(^{15}N,^{13}C)$	2.7545
96		$^{1}J(^{15}N,^{13}C)$	-17.2216
97		$^{1}J(^{13}C,^{1}H)$	131.3341
98		$^{2}J(^{13}C,^{1}H)$	-11.0423
99		$^{3}J(^{15}N,^{1}H)$	-1.4883
100		$^{2}J(^{1}H,^{1}H)$	-17.7738
101	H_3C−F fluoromethane (**14**)	$^{1}J(^{19}F,^{13}C)$	-161.3679
102		$^{1}J(^{13}C,^{1}H)$	143.2246
103		$^{2}J(^{19}F,^{1}H)$	49.1691
104		$^{2}J(^{1}H,^{1}H)$	-11.1455
105	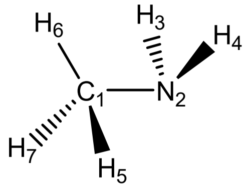 methanamine (**15**)	$^{1}J(^{15}N,^{13}C)$	-5.6575
106		$^{2}J(^{13}C,^{1}H)$	-3.7127
107		$^{1}J(^{15}N,^{1}H)$	-64.9870
108		$^{2}J(^{1}H_3,^{1}H_4)$	-11.2270
109		$^{1}J(^{13}C_1,^{1}H_5)$	129.4057
110		$^{2}J(^{15}N_2,^{1}H_5)$	-1.4980
111		$^{3}J(^{1}H_5,^{1}H_4)$	2.7452
112		$^{3}J(^{1}H_5,^{1}H_3)$	15.7736
113		$^{2}J(^{1}H_7,^{1}H_5)$	-16.0551
114		$^{1}J(^{13}C_1,^{1}H_6)$	126.4270
115		$^{2}J(^{15}N_2,^{1}H_6)$	1.0056
116		$^{3}J(^{1}H_6,^{1}H_4)$	2.1855
117		$^{2}J(^{1}H_6,^{1}H_5)$	-11.8373
118	CH_4 methane (**16**)	$^{1}J(^{13}C,^{1}H)$	121.9934
119		$^{2}J(^{1}H,^{1}H)$	-14.2006
120	CHF_3 fluoroform (**17**)	$^{1}J(^{13}C,^{1}H)$	223.4842
121		$^{1}J(^{19}F,^{13}C)$	-258.5316
122		$^{2}J(^{19}F,^{1}H)$	74.9404
123		$^{2}J(^{19}F,^{19}F)$	128.7114
124	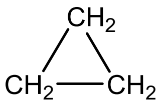 cyclopropane (**18**)	$^{1}J(^{13}C,^{13}C)$	13.4976
125		$^{1}J(^{13}C,^{1}H)$	156.7175
126		$^{2}J(^{13}C,^{1}H)$	-2.9834
127		$^{2}J(^{1}H,^{1}H)$	-6.2636
128		$^{3}J_{cis}(^{1}H,^{1}H)$	9.1494
129		$^{3}J_{trans}(^{1}H,^{1}H)$	5.1558
130	F−C≡C−F 1,2-difluoroethyne (**19**)	$^{1}J(^{13}C,^{13}C)$	407.2449
131		$^{1}J(^{19}F,^{13}C)$	-281.2369

132		$^{2}J(^{19}F,^{13}C)$	38.4985
133		$^{3}J(^{19}F,^{19}F)$	-5.1927
134	F−C≡N<br>fluoroformonitrile (**20**)	$^{1}J(^{19}F,^{13}C)$	-404.2791
135		$^{1}J(^{15}N,^{13}C)$	-3.9761
136		$^{2}J(^{19}F,^{15}N)$	51.0248
137	$H_2C{=}CF_2$<br>1,1-difluoroethene (**21**)	$^{1}J(^{13}C,^{13}C)$	117.1349
138		$^{2}J(^{19}F,^{13}C)$	27.6251
139		$^{1}J(^{19}F,^{13}C)$	-283.6514
140		$^{2}J(^{19}F,^{19}F)$	20.8558
141		$^{1}J(^{13}C,^{1}H)$	163.8866
142		$^{2}J(^{13}C,^{1}H)$	-2.0059
143		$^{3}J_{cis}(^{19}F,^{1}H)$	-1.7101
144		$^{3}J_{trans}(^{19}F,^{1}H)$	27.0386
145		$^{2}J(^{1}H,^{1}H)$	-6.4851
146	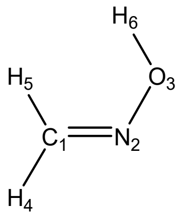<br>formaldehyde oxime (*Z*) (**22**)	$^{1}J(^{15}N,^{13}C)$	-2.4350
147		$^{1}J(^{13}C_1,^{1}H_4)$	183.7285
148		$^{2}J(^{15}N_2,^{1}H_4)$	-12.1876
149		$^{1}J(^{13}C_1,^{1}H_5)$	153.1305
150		$^{2}J(^{15}N_2,^{1}H_5)$	2.9142
151		$^{2}J(^{1}H,^{1}H)$	6.3566
152		$^{3}J(^{13}C,^{1}H)$	4.1976
153		$^{2}J(^{15}N_2,^{1}H_6)$	1.4033
154		$^{4}J(^{1}H_6,^{1}H_4)$	-1.3582
155		$^{4}J(^{1}H_6,^{1}H_5)$	1.3804
156	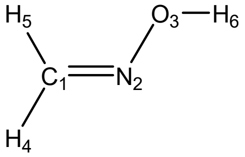<br>formaldehyde oxime (*E*) (**23**)	$^{1}J(^{15}N,^{13}C)$	-5.5260
157		$^{1}J(^{13}C_1,^{1}H_4)$	176.8900
158		$^{2}J(^{15}N_2,^{1}H_4)$	-12.9862
159		$^{1}J(^{13}C_1,^{1}H_5)$	162.4787
160		$^{2}J(^{15}N_2,^{1}H_5)$	2.9139
161		$^{2}J(^{1}H,^{1}H)$	8.0683
162		$^{3}J(^{13}C,^{1}H)$	10.9140
163		$^{2}J(^{15}N_2,^{1}H_6)$	-1.9448
164		$^{4}J(^{1}H_6,^{1}H_4)$	1.1818
165		$^{4}J(^{1}H_6,^{1}H_5)$	-0.8708
166	H−C≡C−F<br>fluoroethyne (**24**)	$^{1}J(^{13}C,^{13}C)$	274.2547
167		$^{2}J(^{19}F,^{13}C)$	22.7654
168		$^{1}J(^{19}F,^{13}C)$	-294.0680
169		$^{1}J(^{13}C,^{1}H)$	279.9613
170		$^{2}J(^{13}C,^{1}H)$	66.5397
171		$^{3}J(^{19}F,^{1}H)$	10.9732
172	H−C≡N<br>hydrogen cyanide (**25**)	$^{1}J(^{15}N,^{13}C)$	-17.8360
173		$^{1}J(^{13}C,^{1}H)$	262.4086
174		$^{2}J(^{15}N,^{1}H)$	-8.3817
175		$^{1}J(^{13}C,^{13}C)$	144.7381
176		$^{2}J(^{19}F_3,^{13}C_1)$	64.5083

177		$^{1}J(^{19}F_3,^{13}C_2)$	-270.2295
178		$^{1}J(^{19}F_4,^{13}C_1)$	-231.3712
179		$^{2}J(^{19}F_4,^{13}C_2)$	36.2879
180		$^{3}J_{trans}(^{19}F_3,^{19}F_4)$	-123.3076
181	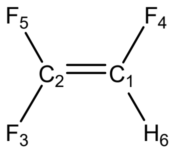	$^{2}J(^{19}F_5,^{13}C_1)$	19.9416
182		$^{1}J(^{19}F_5,^{13}C_2)$	-282.4217
183		$^{2}J(^{19}F,^{19}F)$	72.2087
184		$^{3}J_{cis}(^{19}F_4,^{19}F_5)$	41.2237
185	1,1,2-trifluoroethene (**26**)	$^{1}J(^{13}C,^{1}H)$	203.4436
186		$^{2}J(^{13}C,^{1}H)$	14.4211
187		$^{3}J_{cis}(^{19}F_3,^{1}H_6)$	-5.7218
188		$^{2}J(^{19}F,^{1}H)$	73.0027
189		$^{3}J_{trans}(^{19}F_5,^{1}H_6)$	7.8384
190		$^{1}J(^{13}C,^{13}C)$	101.4011
191		$^{1}J(^{19}F,^{13}C)$	-250.9225
192		$^{2}J(^{19}F,^{13}C)$	9.4773
193	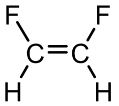	$^{3}J(^{19}F,^{19}F)$	-10.7214
194		$^{1}J(^{13}C,^{1}H)$	196.2737
195	(*Z*)-1,2-difluoroethene (**27**)	$^{2}J(^{13}C,^{1}H)$	22.8977
196		$^{2}J(^{19}F,^{1}H)$	73.0230
197		$^{3}J(^{19}F,^{1}H)$	13.4833
198		$^{3}J(^{1}H,^{1}H)$	3.5178
199		$^{1}J(^{13}C,^{13}C)$	114.9254
200		$^{1}J(^{19}F,^{13}C)$	-238.9953
201		$^{2}J(^{19}F,^{13}C)$	49.2625
202	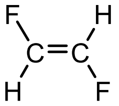	$^{3}J(^{19}F,^{19}F)$	-137.1085
203		$^{2}J(^{13}C,^{1}H)$	4.6546
204	(*E*)-1,2-difluoroethene (**28**)	$^{1}J(^{13}C,^{1}H)$	195.0810
205		$^{3}J(^{19}F,^{1}H)$	0.1181
206		$^{2}J(^{19}F,^{1}H)$	76.4393
207		$^{3}J(^{1}H,^{1}H)$	10.1369
208	HF<br>hydrogen fluoride (**29**)	$^{1}J(^{19}F,^{1}H)$	536.8453
209		$^{1}J(^{15}N,^{15}N)$	-20.6229
210	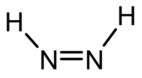	$^{1}J(^{15}N,^{1}H)$	-35.6641
211		$^{2}J(^{15}N,^{1}H)$	1.3567
212	diazene (*Z*) (**31**)	$^{3}J(^{1}H,^{1}H)$	38.6727
213	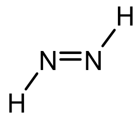	$^{1}J(^{15}N,^{15}N)$	-20.9051
214		$^{2}J(^{15}N,^{1}H)$	-0.2943
215		$^{1}J(^{15}N,^{1}H)$	-45.7176
216	diazene (*E*) (**30**)	$^{3}J(^{1}H,^{1}H)$	38.1474
217		$^{1}J(^{15}N,^{15}N)$	0.9297
218		$^{1}J(^{15}N,^{1}H)$	-58.9291
219		$^{2}J(^{15}N,^{1}H)$	-1.6437
220		$^{2}J(^{1}H,^{1}H)$	-15.8595

221	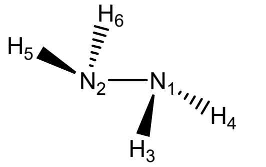	${}^{3}J({}^{1}H_5,{}^{1}H_3)$	1.5296
222	hydrazine (**32**)	${}^{3}J({}^{1}H_4,{}^{1}H_5)$	13.5511
223	N_2<br>molecular nitrogen (**33**)	${}^{1}J({}^{15}N,{}^{15}N)$	-3.03777
224	NH_3	${}^{1}J({}^{15}N,{}^{1}H)$	-61.5296
225	ammonia (**34**)	${}^{2}J({}^{1}H,{}^{1}H)$	-11.7404
226		${}^{1}J({}^{13}C_1,{}^{13}C_2)$	54.826
227		${}^{1}J({}^{13}C_3,{}^{13}C_1)$	69.7055
228		${}^{2}J({}^{13}C_3,{}^{13}C_2)$	1.894
229		${}^{2}J({}^{15}N_4,{}^{13}C_1)$	2.892061
230		${}^{1}J({}^{15}N_4,{}^{13}C_2)$	-1.95769
231		${}^{2}J({}^{15}N_4,{}^{13}C_3)$	1.030464
232		${}^{2}J({}^{15}N_5,{}^{13}C_1)$	-5.75298
233		${}^{2}J({}^{15}N_5,{}^{13}C_2)$	-0.73799
234		${}^{1}J({}^{15}N_5,{}^{13}C_3)$	-15.3773
235		${}^{1}J({}^{15}N,{}^{15}N)$	-11.873
236		${}^{1}J({}^{13}C_1,{}^{1}H_6)$	171.9922
237		${}^{2}J({}^{13}C_2,{}^{1}H_6)$	5.1473
238		${}^{2}J({}^{13}C3,{}^{1}H_6)$	7.641
239		${}^{3}J({}^{15}N_4,{}^{1}H_6)$	-1.102
240	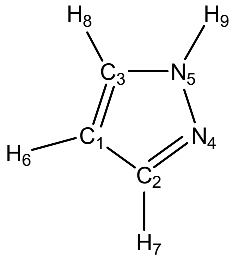	${}^{3}J({}^{15}N_5,{}^{1}H_6)$	-5.80138
241		${}^{2}J({}^{13}C_1,{}^{1}H_7)$	10.5737
242		${}^{1}J({}^{13}C_2,{}^{1}H_7)$	181.5466
243		${}^{3}J({}^{13}C_3,{}^{1}H_7)$	4.7636
244		${}^{2}J({}^{15}N_4,{}^{1}H_7)$	-11.9619
245		${}^{3}J({}^{15}N_5,{}^{1}H_7)$	-8.84662
246		${}^{3}J({}^{1}H_7,{}^{1}H_6)$	1.8581
247	1*H*-pyrazole (**35**)	${}^{2}J({}^{13}C_1,{}^{1}H_8)$	6.8886
248		${}^{3}J({}^{13}C_2,{}^{1}H_8)$	7.9671
249		${}^{1}J({}^{13}C_3,{}^{1}H_8)$	179.9861
250		${}^{3}J({}^{15}N_4,{}^{1}H_8)$	0.287845
251		${}^{2}J({}^{15}N_5,{}^{1}H_8)$	-4.49766
252		${}^{3}J({}^{1}H_8,{}^{1}H_6)$	3.0534
253		${}^{4}J({}^{1}H_8,{}^{1}H_7)$	0.1402
254		${}^{3}J({}^{13}C_1,{}^{1}H_9)$	5.5054
255		${}^{3}J({}^{13}C_2,{}^{1}H_9)$	10.3614
256		${}^{2}J({}^{13}C_3,{}^{1}H_9)$	9.1719
257		${}^{2}J({}^{15}N_4,{}^{1}H_9)$	-8.64504
258		${}^{1}J({}^{15}N,{}^{1}H)$	-106.91
259		${}^{4}J({}^{1}H_9,{}^{1}H_6)$	2.0027
260		${}^{4}J({}^{1}H_9,{}^{1}H_7)$	1.889
261		${}^{3}J({}^{1}H_9,{}^{1}H_8)$	1.6854

262		$^{1}J(^{13}C,^{13}C)$	71.4329
263		$^{2}J(^{13}C_1,^{13}C_3)$	-4.3136
264		$^{2}J(^{13}C_3,^{13}C_2)$	9.0869
265		$^{1}J(^{15}N_4,^{13}C_1)$	1.390692
266		$^{2}J(^{15}N_4,^{13}C_2)$	2.456926
267		$^{1}J(^{15}N_4,^{13}C_3)$	-2.37248
268		$^{2}J(^{15}N_5,^{13}C_1)$	-6.27986
269		$^{1}J(^{15}N_5,^{13}C_2)$	-16.9143
270		$^{1}J(^{15}N_5,^{13}C_3)$	-13.1482
271		$^{2}J(^{15}N,^{15}N)$	-1.5484
272		$^{1}J(^{13}C_1,^{1}H_6)$	185.3182
273		$^{2}J(^{13}C_2,^{1}H_6)$	15.548
274		$^{3}J(^{13}C_3,^{1}H_6)$	11.6165
275		$^{2}J(^{15}N_4,^{1}H_6)$	-9.80189
276	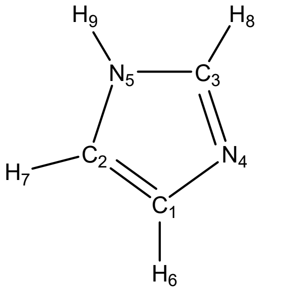	$^{3}J(^{15}N_5,^{1}H_6)$	-3.58474
277		$^{2}J(^{13}C_1,^{1}H_7)$	8.2014
278		$^{1}J(^{13}C_2,^{1}H_7)$	183.7141
279		$^{3}J(^{13}C_3,^{1}H_7)$	6.8483
280		$^{3}J(^{15}N_4,^{1}H_7)$	-1.1194
281		$^{2}J(^{15}N_5,^{1}H_7)$	-4.61969
282		$^{3}J(^{1}H_7,^{1}H_6)$	2.1324
283	1*H*-imidazole (**36**)	$^{3}J(^{13}C_1,^{1}H_8)$	11.4433
284		$^{3}J(C_2,^{1}H_8)$	3.3475
285		$^{1}J(C_3,^{1}H_8)$	201.4806
286		$^{2}J(^{15}N_4,^{1}H_8)$	-11.2013
287		$^{2}J(^{15}N_5,^{1}H_8)$	-8.89389
288		$^{4}J(^{1}H_8,^{1}H_6)$	0.2573
289		$^{4}J(^{1}H8,^{1}H_7)$	1.3642
290		$^{3}J(^{13}C_1,^{1}H_9)$	7.9177
291		$^{2}J(^{13}C_2,^{1}H_9)$	4.4538
292		$^{2}J(^{13}C_3,^{1}H_9)$	4.645
293		$^{3}J(^{15}N_4,^{1}H_9)$	0.132981
294		$^{1}J(^{15}N,^{1}H)$	-96.6187
295		$^{4}J(^{1}H_9,^{1}H_6)$	1.735
296		$^{3}J(^{1}H_9,^{1}H_7)$	2.0916
297		$^{3}J(^{1}H_9,^{1}H_8)$	0.989
298		$^{1}J(^{13}C_1,^{13}C_2)$	57.1838
299		$^{2}J(^{13}C_2,^{13}C_3)$	-4.0333
300		$^{2}J(^{13}C_1,^{13}C_4)$	-3.6176
301	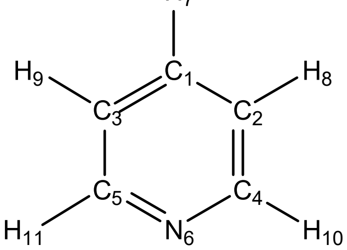	$^{1}J(^{13}C_2,^{13}C_4)$	57.8242
302		$^{3}J(^{13}C_3,^{13}C_4)$	15.1074
303		$^{2}J(^{13}C_5,^{13}C_4)$	-7.1612
304		$^{3}J(^{15}N_6,^{13}C_1)$	-4.47241
305	pyridine (**37**)	$^{2}J(^{15}N_6,^{13}C_2)$	2.921239
306		$^{1}J(^{15}N,^{13}C)$	-1.55453

307		$^{1}J(^{13}C_1,^{1}H_7)$	155.9064
308		$^{2}J(^{13}C_2,^{1}H_7)$	-0.4168
309		$^{3}J(^{13}C_4,^{1}H_7)$	6.8921
310		$^{4}J(^{15}N,^{1}H)$	0.513268
311		$^{2}J(^{13}C_1,^{1}H_8)$	-0.3635
312		$^{1}J(^{13}C_2,^{1}H_8)$	158.5221
313		$^{3}J(^{13}C_3,^{1}H_8)$	6.7305
314		$^{2}J(^{13}C_4,^{1}H_8)$	1.8739
315		$^{4}J(^{13}C_5,^{1}H_8)$	-1.2966
316		$^{3}J(^{15}N,^{1}H)$	-1.61485
317		$^{3}J(^{1}H_7,^{1}H_8)$	8.1098
318		$^{4}J(^{1}H_8,^{1}H_9)$	0.7861
319		$^{3}J(^{13}C_1,^{1}H_{10})$	6.7187
320		$^{2}J(^{13}C_2,^{1}H_{10})$	7.4537
321		$^{4}J(^{13}C_3,^{1}H_{10})$	-2.1
322		$^{1}J(^{13}C_4,^{1}H_{10})$	173.1328
323		$^{3}J(^{13}C_5,^{1}H_{10})$	11.4334
324		$^{2}J(^{15}N,^{1}H)$	-9.92997
325		$^{4}J(^{1}H_7,^{1}H_{10})$	1.3238
326		$^{3}J(^{1}H_8,^{1}H_{10})$	5.4784
327		$^{5}J(^{1}H,^{1}H)$	1.3763
328		$^{4}J(^{1}H_{10},^{1}H_{11})$	-0.7099
329		$^{2}J(^{15}N,^{13}C)$	-4.40381
330		$^{1}J(^{13}C_2,^{13}C_3)$	55.1747
331		$^{1}J(^{15}N,^{13}C)$	-16.0039
332		$^{1}J(^{13}C_5,^{13}C_3)$	71.1549
333		$^{2}J(^{13}C_2,^{13}C_5)$	1.167
334		$^{2}J(^{13}C_4,^{13}C_5)$	7.5189
335		$^{3}J(^{15}N,^{1}H)$	-5.51942
336		$^{1}J(^{13}C_3,^{1}H_7)$	166.7375
337		$^{2}J(^{13}C_2,^{1}H_7)$	3.3283
338		$^{2}J(^{13}C_5,^{1}H_7)$	7.0039
339	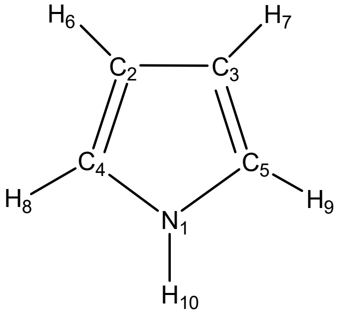	$^{3}J(^{13}C_4,^{1}H_7)$	7.4076
340		$^{3}J(^{1}H_6,^{1}H_7)$	3.6304
341		$^{2}J(^{15}N,^{1}H)$	-3.99743
342		$^{2}J(^{13}C_3,^{1}H_9)$	6.2451
343		$^{3}J(^{13}C_2,^{1}H_9)$	7.7112
344	1*H*-pyrrole (**38**)	$^{1}J(^{13}C_5,^{1}H_9)$	179.1307
345		$^{3}J(^{13}C_4,^{1}H_9)$	6.0194
346		$^{3}J(^{1}H_7,^{1}H_9)$	3.6522
347		$^{4}J(^{1}H_6,^{1}H_9)$	0.9699
348		$^{4}J(^{1}H_8,^{1}H_9)$	2.1464
349		$^{1}J(^{15}N,^{1}H)$	-96.4703
350		$^{3}J(^{13}C_3,^{1}H_{10})$	7.1589
351		$^{2}J(^{13}C_5,^{1}H_{10})$	3.5968

352		$^{4}J(^{1}H_{7},^{1}H_{10})$	2.5077
353		$^{3}J(^{1}H_{9},^{1}H_{10})$	2.7968
354		$^{1}J(^{13}C_{2},^{13}C_{1})$	73.6689
355		$^{2}J(^{13}C,^{13}C)$	-0.0394
356		$^{1}J(^{13}C_{2},^{13}C_{3})$	43.8237
357		$^{1}J(^{13}C_{1},^{1}H_{4})$	154.9785
358		$^{2}J(^{13}C_{2},^{1}H_{4})$	-1.9885
359		$^{3}J(^{13}C_{3},^{1}H_{4})$	12.0214
360		$^{1}J(^{13}C_{1},^{1}H_{5})$	151.2595
361		$^{2}J(^{13}C_{2},^{1}H_{5})$	-3.4829
362		$^{3}J(^{13}C_{3},^{1}H_{5})$	7.7534
363		$^{2}J(^{1}H_{4},^{1}H_{5})$	-0.1499
364		$^{2}J(^{13}C_{1},^{1}H_{6})$	-0.8035
365		$^{1}J(^{13}C_{2},^{1}H_{6})$	148.7295
366		$^{2}J(^{13}C_{3},^{1}H_{6})$	4.0292
367		$^{3}J(^{1}H_{4},^{1}H_{6})$	10.9873
368	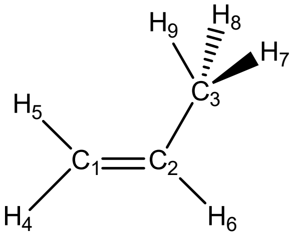	$^{3}J(^{1}H_{5},^{1}H_{6})$	16.5939
369		$^{3}J(^{13}C_{1},^{1}H_{7})$	6.2367
370		$^{2}J(^{13}C_{2},^{1}H_{7})$	-8.2642
371	prop-1-ene (**39**)	$^{1}J(^{13}C_{3},^{1}H_{7})$	122.4114
372		$^{4}J(^{1}H_{4},^{1}H_{7})$	-2.7655
373		$^{4}J(^{1}H_{5},^{1}H_{7})$	-3.0214
374		$^{3}J(^{1}H_{6},^{1}H_{7})$	3.9393
375		$^{2}J(^{1}H_{7},^{1}H_{8})$	-19.4602
376		$^{3}J(^{13}C_{1},^{1}H_{9})$	6.4263
377		$^{2}J(^{13}C_{2},^{1}H_{9})$	-6.1655
378		$^{1}J(^{13}C_{3},^{1}H_{9})$	123.597
379		$^{4}J(^{1}H_{4},^{1}H_{9})$	-0.3762
380		$^{4}J(^{1}H_{5},^{1}H_{9})$	-0.5827
381		$^{3}J(^{1}H_{6},^{1}H_{9})$	12.2287
382		$^{2}J(^{1}H_{7},^{1}H_{9})$	-14.45
383		$^{1}J(^{13}C_{1},^{13}C_{2})$	52.9292
384		$^{1}J(^{13}C_{1},^{13}C_{3})$	74.3477
385		$^{2}J(^{13}C_{2},^{13}C_{3})$	0.1224
386		$^{2}J(^{13}C_{3},^{13}C_{4})$	3.9862
387		$^{1}J(^{13}C_{1},^{1}H_{6})$	170.6103
388		$^{2}J(^{13}C_{2},^{1}H_{6})$	3.1537
389	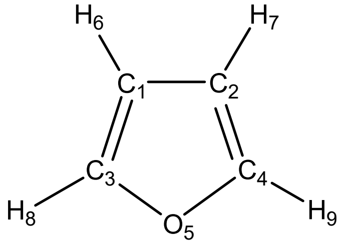	$^{2}J(^{13}C_{3},^{1}H_{6})$	8.8688
390		$^{3}J(^{13}C_{4},^{1}H_{6})$	6.8388
391		$^{3}J(^{1}H_{6},^{1}H_{7})$	3.2475
392	furan (**40**)	$^{2}J(^{13}C_{1},^{1}H_{8})$	12.2006
393		$^{3}J(^{13}C_{2},^{1}H_{8})$	6.3579
394		$^{1}J(^{13}C_{3},^{1}H_{8})$	196.4972
395		$^{3}J(^{13}C_{4},^{1}H_{8})$	6.7403
396		$^{3}J(^{1}H_{6},^{1}H_{8})$	2.8121

397		$^{4}J(^{1}H_{7},^{1}H_{8})$	0.3335
398		$^{4}J(^{1}H_{8},^{1}H_{9})$	1.5873
399		$^{1}J(^{19}F,^{13}C)$	-246.226
400		$^{2}J(^{19}F,^{13}C)$	21.8769
401		$^{1}J(^{13}C_{2},^{13}C_{3})$	73.5386
402		$^{2}J(^{13}C_{3},^{13}C_{4})$	3.0177
403		$^{3}J(^{19}F,^{13}C)$	5.0805
404		$^{2}J(^{13}C_{2},^{13}C_{6})$	-0.4713
405		$^{1}J(^{13}C_{3},^{13}C_{6})$	60.2346
406		$^{3}J(^{13}C_{4},^{13}C_{6})$	7.7155
407		$^{2}J(^{13}C_{5},^{13}C_{6})$	-2.3497
408		$^{4}J(^{19}F,^{13}C)$	5.5286
409		$^{3}J(^{13}C_{7},^{13}C_{2})$	11.4546
410		$^{2}J(^{13}C_{3},^{13}C_{7})$	-3.839
411		$^{1}J(^{13}C_{6},^{13}C_{7})$	59.598
412		$^{3}J(^{19}F,^{1}H)$	6.1854
413		$^{2}J(^{13}C_{2},^{1}H_{8})$	-6.3224
414		$^{1}J(^{13}C_{3},^{1}H_{8})$	159.193
415		$^{3}J(^{13}C_{4},^{1}H_{8})$	4.529
416	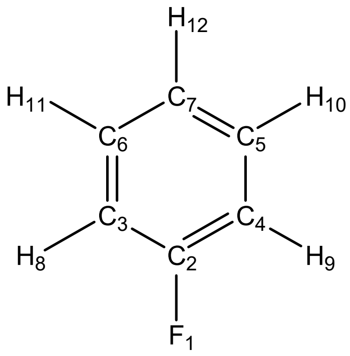	$^{2}J(^{13}C_{6},^{1}H_{8})$	-1.4736
417		$^{4}J(^{13}C_{5},^{1}H_{8})$	-1.249
418		$^{3}J(^{13}C_{7},^{1}H_{8})$	7.9
419		$^{4}J(^{1}H_{8},^{1}H_{9})$	2.0571
420		$^{4}J(^{19}F,^{1}H)$	4.9098
421		$^{3}J(^{13}C_{2},^{1}H_{11})$	10.765
422	1-fluorobenzene (**41**)	$^{2}J(^{13}C_{3},^{1}H_{11})$	-0.278
423		$^{4}J(^{13}C_{4},^{1}H_{11})$	-1.9243
424		$^{1}J(^{13}C_{6},^{1}H_{11})$	156.2285
425		$^{3}J(^{13}C_{5},^{1}H_{11})$	9.0875
426		$^{2}J(^{13}C_{7},^{1}H_{11})$	-0.5449
427		$^{3}J(^{1}H_{8},^{1}H_{11})$	8.8443
428		$^{5}J(^{1}H_{9},^{1}H_{11})$	0.9495
429		$^{4}J(^{1}H_{10},^{1}H_{11})$	1.1512
430		$^{5}J(^{19}F,^{1}H)$	-1.3525
431		$^{4}J(^{13}C_{2},^{1}H_{12})$	-2.2763
432		$^{3}J(^{13}C_{3},^{1}H_{12})$	8.3189
433		$^{2}J(^{13}C_{6},^{1}H_{12})$	0.3456
434		$^{1}J(^{13}C_{7},^{1}H_{12})$	157.9553
435		$^{4}J(^{1}H_{8},^{1}H_{12})$	0.5524
436		$^{3}\mathrm{J}(^{1}H_{11},^{1}H_{12})$	8.0937

Table S10. Symmetry independent values of SSCC (in Hz) in molecules of set 1 calculated at the SOPPA(CCSD) level with the pcJ-1 basis set.

#	Molecule	Type of SSCC [1]	SSCC value
1	$H_2C{=}C{=}CH_2$ propa-1,2-diene (**1**)	$^{1}J(^{13}C,^{13}C)$	109.5346
2		$^{2}J(^{13}C,^{13}C)$	7.0723
3		$^{2}J(^{13}C,^{1}H)$	-7.3559
4		$^{1}J(^{13}C,^{1}H)$	166.7814
5		$^{3}J(^{13}C,^{1}H)$	8.5287
6		$^{2}J(^{1}H,^{1}H)$	-15.1312
7		$^{4}J(^{1}H,^{1}H)$	-9.7362
8	$F_2C{=}CF_2$ Perfluoroethene (**2**)	$^{1}J(^{13}C,^{13}C)$	202.4252
9		$^{1}J(^{13}C,^{19}F)$	-251.7649
10		$^{2}J(^{13}C,^{19}F)$	55.1590
11		$^{2}J(^{19}F,^{19}F)$	143.7419
12		$^{3}J_{cis}(^{19}F,^{19}F)$	91.5564
13		$^{3}J_{trans}(^{19}F,^{19}F)$	-109.5294
14	$HC{\equiv}CH$ ethyne (**3**)	$^{1}J(^{13}C,^{13}C)$	196.3570
15		$^{1}J(^{13}C,^{1}H)$	248.4419
16		$^{2}J(^{13}C,^{1}H)$	48.6314
17		$^{3}J(^{1}H,^{1}H)$	11.4801
18	$H_2C{=}CH_2$ ethene (**4**)	$^{1}J(^{13}C,^{13}C)$	76.7950
19		$^{1}J(^{13}C,^{1}H)$	154.1971
20		$^{2}J(^{13}C,^{1}H)$	-4.0715
21		$^{3}J_{cis}(^{1}H,^{1}H)$	12.1983
22		$^{2}J(^{1}H,^{1}H)$	-1.5016
23		$^{3}J_{trans}(^{1}H,^{1}H)$	18.3139
24	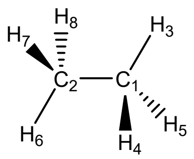 ethane (**5**)	$^{1}J(^{13}C,^{13}C)$	38.3407
25		$^{1}J(^{13}C,^{1}H)$	122.1741
26		$^{2}J(^{13}C,^{1}H)$	-5.5313
27		$^{3}J_{trans}(^{1}H_3,^{1}H_6)$	15.3689
28		$^{2}J(^{1}H,^{1}H)$	-15.3831
29		$^{3}J_{gauche}(^{1}H_4,^{1}H_6)$	3.7572
30	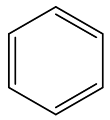 benzene (**6**)	$^{3}J(^{13}C,^{13}C)$	10.8635
31		$^{1}J(^{13}C,^{13}C)$	63.5868
32		$^{2}J(^{13}C,^{13}C)$	-3.9403
33		$^{1}J(^{13}C,^{1}H)$	154.8225
34		$^{4}J(^{13}C,^{1}H)$	-2.0271
35		$^{2}J(^{13}C,^{1}H)$	-0.8916
36		$^{3}J(^{13}C,^{1}H)$	7.8112
37		$^{3}J(^{1}H,^{1}H)$	7.9623
38		$^{4}J(^{1}H,^{1}H)$	0.3328
39		$^{5}J(^{1}H,^{1}H)$	1.2469
40	CF_4 perfluoromethane (**7**)	$^{1}J(^{19}F,^{13}C)$	-243.2309
41		$^{2}J(^{19}F,^{19}F)$	53.1273

42		$^{1}J(^{13}C,^{13}C)$	93.6814
43		$^{2}J(^{19}F,^{13}C)$	14.8945
44		$^{1}J(^{19}F,^{13}C)$	-243.7341
45		$^{1}J(^{13}C_1,^{1}H_4)$	158.8914
46		$^{2}J(^{13}C_2,^{1}H_4)$	5.5328
47	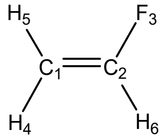	$^{3}J_{trans}(^{19}F_3,^{1}H_4)$	39.2729
48		$^{1}J(^{13}C_1,^{1}H_5)$	157.9245
49		$^{2}J(^{13}C_2,^{1}H_5)$	-10.8810
50		$^{3}J_{cis}(^{19}F_3,^{1}H_5)$	11.6960
51	fluoroethene (**8**)	$^{2}J(^{1}H,^{1}H)$	-6.0727
52		$^{2}J(^{13}C_1,^{1}H_6)$	11.8119
53		$^{1}J(^{13}C_2,^{1}H_6)$	191.1544
54		$^{2}J(^{19}F,^{1}H)$	82.9130
55		$^{3}J_{cis}(^{1}H_6,^{1}H_4)$	6.2110
56		$^{3}J_{trans}(^{1}H_6,^{1}H_5)$	12.8865
57		$^{1}J(^{19}F,^{13}C)$	-207.3635
58		$^{2}J(^{19}F,^{19}F)$	346.5272
59	CH_2F_2	$^{1}J(^{13}C,^{1}H)$	173.8644
60	difluoromethane (**9**)	$^{2}J(^{19}F,^{1}H)$	51.6097
61		$^{2}J(^{1}H,^{1}H)$	-0.5273
62		$^{1}J(^{15}N,^{13}C)$	-6.3205
63		$^{1}J(^{13}C_1,^{1}H_3)$	171.3582
64		$^{2}J(^{15}N_2,^{1}H_3)$	-8.9854
65	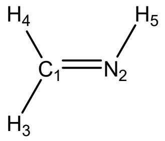	$^{1}J(^{13}C_1,^{1}H_4)$	153.9661
66		$^{2}J(^{15}N_2,^{1}H_4)$	3.9238
67		$^{2}J(^{1}H,^{1}H)$	14.6581
68	methanimine (**10**)	$^{2}J(^{13}C,^{1}H)$	-14.2041
69		$^{1}J(^{15}N,^{1}H)$	-50.3427
70		$^{3}J_{trans}(^{1}H_5,^{1}H_3)$	24.3343
71		$^{3}J_{cis}(^{1}H_5,^{1}H_4)$	17.6325
72		$^{1}J(^{13}C_1,^{13}C_2)$	196.5891
73		$^{2}J(^{13}C,^{13}C)$	12.1410
74		$^{1}J(^{13}C_2,^{13}C_3)$	74.7750
75	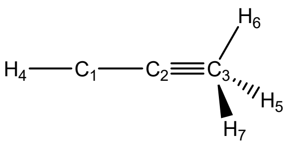	$^{1}J(^{13}C_1,^{1}H_4)$	246.7013
76		$^{2}J(^{13}C_2,^{1}H_4)$	48.0963
77		$^{3}J(^{13}C_3,^{1}H_4)$	4.5032
78		$^{3}J(^{13}C_1,^{1}H_6)$	3.9934
79	prop-1-yne (**11**)	$^{2}J(^{13}C_2,^{1}H_6)$	-12.6034
80		$^{1}J(^{13}C_3,^{1}H_6)$	128.4584
81		$^{4}J(^{1}H,^{1}H)$	-4.0702
82		$^{2}J(^{1}H,^{1}H)$	-18.6310
83		$^{1}J(^{13}C,^{13}C)$	45.9752
84		$^{1}J(^{13}C_1,^{1}H_4)$	163.2396
85		$^{2}J(^{13}C_2,^{1}H_4)$	25.9571
86		$^{2}J(^{13}C_1,^{1}H_5)$	-8.7483

87	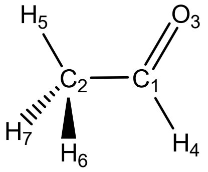 acetaldehyde (**12**)	$^{1}J(^{13}C_2,^{1}H_5)$	131.7115
88		$^{3}J_{trans}(^{1}H_5,^{1}H_4)$	7.8097
89		$^{2}J(^{13}C_1,^{1}H_6)$	-7.1100
90		$^{1}J(^{13}C_2,^{1}H_6)$	120.5394
91		$^{3}J_{gauche}(^{1}H_6,^{1}H_4)$	0.3316
92		$^{2}J(^{1}H_6,^{1}H_5)$	-14.8406
93		$^{2}J(^{1}H_6,^{1}H_7)$	-21.2463
94	$N{\equiv}C{-}CH_3$ acetonitrile (**13**)	$^{1}J(^{13}C,^{13}C)$	67.7474
95		$^{2}J(^{15}N,^{13}C)$	2.5415
96		$^{1}J(^{15}N,^{13}C)$	-20.0480
97		$^{1}J(^{13}C,^{1}H)$	131.8965
98		$^{2}J(^{13}C,^{1}H)$	-11.8021
99		$^{3}J(^{15}N,^{1}H)$	-1.5113
100		$^{2}J(^{1}H,^{1}H)$	-18.7156
101	$H_3C{-}F$ fluoromethane (**14**)	$^{1}J(^{19}F,^{13}C)$	-146.6512
102		$^{1}J(^{13}C,^{1}H)$	143.4785
103		$^{2}J(^{19}F,^{1}H)$	50.0325
104		$^{2}J(^{1}H,^{1}H)$	-11.8657
105	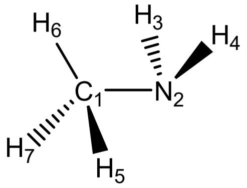 methanamine (**15**)	$^{1}J(^{15}N,^{13}C)$	-7.4910
106		$^{2}J(^{13}C,^{1}H)$	-4.1082
107		$^{1}J(^{15}N,^{1}H)$	-64.8818
108		$^{2}J(^{1}H_3,^{1}H_4)$	-12.2228
109		$^{1}J(^{13}C_1,^{1}H_5)$	129.9081
110		$^{2}J(^{15}N_2,^{1}H_5)$	-1.3119
111		$^{3}J(^{1}H_5,^{1}H_4)$	2.6159
112		$^{3}J(^{1}H_5,^{1}H_3)$	15.2958
113		$^{2}J(^{1}H_7,^{1}H_5)$	-16.9087
114		$^{1}J(^{13}C_1,^{1}H_6)$	125.5099
115		$^{2}J(^{15}N_2,^{1}H_6)$	1.0020
116		$^{3}J(^{1}H_6,^{1}H_4)$	1.9751
117		$^{2}J(^{1}H_6,^{1}H_5)$	-12.5739
118	CH_4 methane (**16**)	$^{1}J(^{13}C,^{1}H)$	122.6257
119		$^{2}J(^{1}H,^{1}H)$	-15.3748
120	CHF_3 fluoroform (**17**)	$^{1}J(^{13}C,^{1}H)$	222.2359
121		$^{1}J(^{19}F,^{13}C)$	-242.9189
122		$^{2}J(^{19}F,^{1}H)$	75.7912
123		$^{2}J(^{19}F,^{19}F)$	152.5547
124	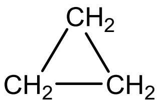 cyclopropane (**18**)	$^{1}J(^{13}C,^{13}C)$	15.9708
125		$^{1}J(^{13}C,^{1}H)$	156.0975
126		$^{2}J(^{13}C,^{1}H)$	-3.2638
127		$^{2}J(^{1}H,^{1}H)$	-6.8617
128		$^{3}J_{cis}(^{1}H,^{1}H)$	8.8091
129		$^{3}J_{trans}(^{1}H,^{1}H)$	5.0172
130	$F{-}C{\equiv}C{-}F$ 1,2-difluoroethyne (**19**)	$^{1}J(^{13}C,^{13}C)$	407.7710
131		$^{1}J(^{19}F,^{13}C)$	-248.6469

132		$^{2}J(^{19}F,^{13}C)$	48.4645
133		$^{3}J(^{19}F,^{19}F)$	6.1183
134	F−C≡N<br>fluoroformonitrile (**20**)	$^{1}J(^{19}F,^{13}C)$	-374.4662
135		$^{1}J(^{15}N,^{13}C)$	-7.4081
136		$^{2}J(^{19}F,^{15}N)$	47.4489
137	$H_2C=CF_2$<br>1,1-difluoroethene (**21**)	$^{1}J(^{13}C,^{13}C)$	122.5693
138		$^{2}J(^{19}F,^{13}C)$	29.5633
139		$^{1}J(^{19}F,^{13}C)$	-268.4922
140		$^{2}J(^{19}F,^{19}F)$	49.5775
141		$^{1}J(^{13}C,^{1}H)$	164.6329
142		$^{2}J(^{13}C,^{1}H)$	-3.0776
143		$^{3}J_{cis}(^{19}F,^{1}H)$	-2.7146
144		$^{3}J_{trans}(^{19}F,^{1}H)$	26.9736
145		$^{2}J(^{1}H,^{1}H)$	-7.7428
146	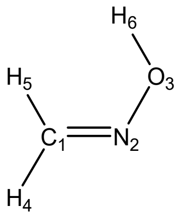<br>formaldehyde oxime (*Z*) (**22**)	$^{1}J(^{15}N,^{13}C)$	-5.2051
147		$^{1}J(^{13}C_1,^{1}H_4)$	184.1389
148		$^{2}J(^{15}N_2,^{1}H_4)$	-11.2619
149		$^{1}J(^{13}C_1,^{1}H_5)$	152.9880
150		$^{2}J(^{15}N_2,^{1}H_5)$	3.1833
151		$^{2}J(^{1}H,^{1}H)$	4.6393
152		$^{3}J(^{13}C,^{1}H)$	4.0464
153		$^{2}J(^{15}N_2,^{1}H_6)$	1.3448
154		$^{4}J(^{1}H_6,^{1}H_4)$	-1.4830
155		$^{4}J(^{1}H_6,^{1}H_5)$	1.2820
156	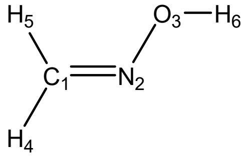<br>formaldehyde oxime (*E*) (**23**)	$^{1}J(^{15}N,^{13}C)$	-8.5128
157		$^{1}J(^{13}C_1,^{1}H_4)$	176.6103
158		$^{2}J(^{15}N_2,^{1}H_4)$	-11.9189
159		$^{1}J(^{13}C_1,^{1}H_5)$	163.0643
160		$^{2}J(^{15}N_2,^{1}H_5)$	3.2319
161		$^{2}J(^{1}H,^{1}H)$	6.3255
162		$^{3}J(^{13}C,^{1}H)$	10.6523
163		$^{2}J(^{15}N_2,^{1}H_6)$	-1.6056
164		$^{4}J(^{1}H_6,^{1}H_4)$	1.0244
165		$^{4}J(^{1}H_6,^{1}H_5)$	-0.7485
166	H−C≡C−F<br>fluoroethyne (**24**)	$^{1}J(^{13}C,^{13}C)$	278.4132
167		$^{2}J(^{19}F,^{13}C)$	30.9748
168		$^{1}J(^{19}F,^{13}C)$	-260.4308
169		$^{1}J(^{13}C,^{1}H)$	276.4899
170		$^{2}J(^{13}C,^{1}H)$	62.4989
171		$^{3}J(^{19}F,^{1}H)$	8.2804
172	H−C≡N<br>hydrogen cyanide (**25**)	$^{1}J(^{15}N,^{13}C)$	-21.1096
173		$^{1}J(^{13}C,^{1}H)$	261.4609
174		$^{2}J(^{15}N,^{1}H)$	-7.8831
175		$^{1}J(^{13}C,^{13}C)$	150.6794
176		$^{2}J(^{19}F_3,^{13}C_1)$	67.7580

177	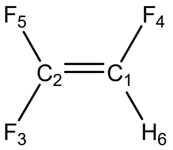<br>1,1,2-trifluoroethene (**26**)	$^{1}J(^{19}F_3,^{13}C_2)$	-255.8284
178		$^{1}J(^{19}F_4,^{13}C_1)$	-219.2429
179		$^{2}J(^{19}F_4,^{13}C_2)$	41.6997
180		$^{3}J_{trans}(^{19}F_3,^{19}F_4)$	-113.3618
181		$^{2}J(^{19}F_5,^{13}C_1)$	23.6788
182		$^{1}J(^{19}F_5,^{13}C_2)$	-267.9989
183		$^{2}J(^{19}F,^{19}F)$	100.0752
184		$^{3}J_{cis}(^{19}F_4,^{19}F_5)$	52.5229
185		$^{1}J(^{13}C,^{1}H)$	202.9107
186		$^{2}J(^{13}C,^{1}H)$	12.7446
187		$^{3}J_{cis}(^{19}F_3,^{1}H_6)$	-7.4333
188		$^{2}J(^{19}F,^{1}H)$	73.5639
189		$^{3}J_{trans}(^{19}F_5,^{1}H_6)$	7.7151
190	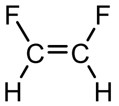<br>(*Z*)-1,2-difluoroethene (**27**)	$^{1}J(^{13}C,^{13}C)$	107.9600
191		$^{1}J(^{19}F,^{13}C)$	-237.3380
192		$^{2}J(^{19}F,^{13}C)$	13.8182
193		$^{3}J(^{19}F,^{19}F)$	1.3303
194		$^{1}J(^{13}C,^{1}H)$	195.0009
195		$^{2}J(^{13}C,^{1}H)$	21.0570
196		$^{2}J(^{19}F,^{1}H)$	73.4884
197		$^{3}J(^{19}F,^{1}H)$	13.1765
198		$^{3}J(^{1}H,^{1}H)$	3.7970
199	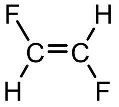<br>(*E*)-1,2-difluoroethene (**28**)	$^{1}J(^{13}C,^{13}C)$	120.6881
200		$^{1}J(^{19}F,^{13}C)$	-225.8777
201		$^{2}J(^{19}F,^{13}C)$	52.8801
202		$^{3}J(^{19}F,^{19}F)$	-123.3158
203		$^{2}J(^{13}C,^{1}H)$	3.2943
204		$^{1}J(^{13}C,^{1}H)$	194.6302
205		$^{3}J(^{19}F,^{1}H)$	-1.5636
206		$^{2}J(^{19}F,^{1}H)$	77.4811
207		$^{3}J(^{1}H,^{1}H)$	10.2675
208	HF<br>hydrogen fluoride (**29**)	$^{1}J(^{19}F,^{1}H)$	531.0778
209	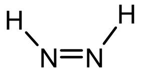<br>diazene (*Z*) (**31**)	$^{1}J(^{15}N,^{15}N)$	-17.6261
210		$^{1}J(^{15}N,^{1}H)$	-34.8366
211		$^{2}J(^{15}N,^{1}H)$	1.0742
212		$^{3}J(^{1}H,^{1}H)$	36.7539
213	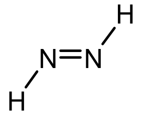<br>diazene (*E*) (**30**)	$^{1}J(^{15}N,^{15}N)$	-18.4086
214		$^{2}J(^{15}N,^{1}H)$	-0.0553
215		$^{1}J(^{15}N,^{1}H)$	-45.9481
216		$^{3}J(^{1}H,^{1}H)$	37.9640
217		$^{1}J(^{15}N,^{15}N)$	1.9746
218		$^{1}J(^{15}N,^{1}H)$	-58.9800
219		$^{2}J(^{15}N,^{1}H)$	-1.5450
220		$^{2}J(^{1}H,^{1}H)$	-17.0924

221	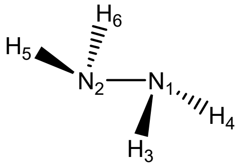	$^{3}J(^{1}H_{5},^{1}H_{3})$	1.3814
222	hydrazine (**32**)	$^{3}J(^{1}H_{4},^{1}H_{5})$	13.3786
223	N_2 molecular nitrogen (**33**)	$^{1}J(^{15}N,^{15}N)$	-1.11747
224	NH_3	$^{1}J(^{15}N,^{1}H)$	-61.0965
225	ammonia (**34**)	$^{2}J(^{1}H,^{1}H)$	-12.9053
226	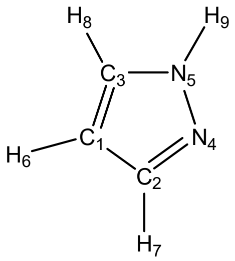 1*H*-pyrazole (**35**)	$^{1}J(^{13}C_{1},^{13}C_{2})$	59.0186
227		$^{1}J(^{13}C_{3},^{13}C_{1})$	74.0562
228		$^{2}J(^{13}C_{3},^{13}C_{2})$	1.2849
229		$^{2}J(^{15}N_{4},^{13}C_{1})$	2.946909
230		$^{1}J(^{15}N_{4},^{13}C_{2})$	-3.94343
231		$^{2}J(^{15}N_{4},^{13}C_{3})$	0.830151
232		$^{2}J(^{15}N_{5},^{13}C_{1})$	-5.48562
233		$^{2}J(^{15}N_{5},^{13}C_{2})$	-0.51074
234		$^{1}J(^{15}N_{5},^{13}C_{3})$	-16.9919
235		$^{1}J(^{15}N,^{15}N)$	-10.2564
236		$^{1}J(^{13}C_{1},^{1}H_{6})$	171.3671
237		$^{2}J(^{13}C_{2},^{1}H_{6})$	4.694
238		$^{2}J(^{13}C3,^{1}H_{6})$	6.7438
239		$^{3}J(^{15}N_{4},^{1}H_{6})$	-1.18982
240		$^{3}J(^{15}N_{5},^{1}H_{6})$	-5.55267
241		$^{2}J(^{13}C_{1},^{1}H_{7})$	9.8782
242		$^{1}J(^{13}C_{2},^{1}H_{7})$	180.5027
243		$^{3}J(^{13}C_{3},^{1}H_{7})$	4.7118
244		$^{2}J(^{15}N_{4},^{1}H_{7})$	-11.2556
245		$^{3}J(^{15}N_{5},^{1}H_{7})$	-8.63789
246		$^{3}J(^{1}H_{7},^{1}H_{6})$	1.8969
247		$^{2}J(^{13}C_{1},^{1}H_{8})$	6.0584
248		$^{3}J(^{13}C_{2},^{1}H_{8})$	7.7497
249		$^{1}J(^{13}C_{3},^{1}H_{8})$	179.2367
250		$^{3}J(^{15}N_{4},^{1}H_{8})$	0.333996
251		$^{2}J(^{15}N_{5},^{1}H_{8})$	-4.03587
252		$^{3}J(^{1}H_{8},^{1}H_{6})$	3.1441
253		$^{4}J(^{1}H_{8},^{1}H_{7})$	-0.1677
254		$^{3}J(^{13}C_{1},^{1}H_{9})$	5.2972
255		$^{3}J(^{13}C_{2},^{1}H_{9})$	9.7711
256		$^{2}J(^{13}C_{3},^{1}H_{9})$	8.8172
257		$^{2}J(^{15}N_{4},^{1}H_{9})$	-8.1531
258		$^{1}J(^{15}N,^{1}H)$	-106.705
259		$^{4}J(^{1}H_{9},^{1}H_{6})$	1.6399
260		$^{4}J(^{1}H_{9},^{1}H_{7})$	1.4973
261		$^{3}J(^{1}H_{9},^{1}H_{8})$	1.6632

262		$^{1}J(^{13}C,^{13}C)$	75.3319
263		$^{2}J(^{13}C_1,^{13}C_3)$	-4.7158
264		$^{2}J(^{13}C_3,^{13}C_2)$	8.9162
265		$^{1}J(^{15}N_4,^{13}C_1)$	-0.38814
266		$^{2}J(^{15}N_4,^{13}C_2)$	2.533657
267		$^{1}J(^{15}N_4,^{13}C_3)$	-4.4414
268		$^{2}J(^{15}N_5,^{13}C_1)$	-5.977
269		$^{1}J(^{15}N_5,^{13}C_2)$	-18.4109
270		$^{1}J(^{15}N_5,^{13}C_3)$	-14.4837
271		$^{2}J(^{15}N,^{15}N)$	-1.43132
272		$^{1}J(^{13}C_1,^{1}H_6)$	183.738
273		$^{2}J(^{13}C_2,^{1}H_6)$	14.6127
274		$^{3}J(^{13}C_3,^{1}H_6)$	11.2685
275		$^{2}J(^{15}N_4,^{1}H_6)$	-9.10865
276	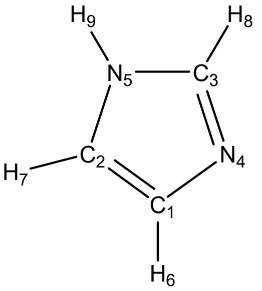	$^{3}J(^{15}N_5,^{1}H_6)$	-3.44727
277		$^{2}J(^{13}C_1,^{1}H_7)$	7.3821
278		$^{1}J(^{13}C_2,^{1}H_7)$	182.8901
279		$^{3}J(^{13}C_3,^{1}H_7)$	6.2448
280		$^{3}J(^{15}N_4,^{1}H_7)$	-1.23148
281		$^{2}J(^{15}N_5,^{1}H_7)$	-4.11078
282		$^{3}J(^{1}H_7,^{1}H_6)$	2.2871
283	1*H*-imidazole (**36**)	$^{3}J(^{13}C_1,^{1}H_8)$	11.1916
284		$^{3}J(C_2,^{1}H_8)$	3.008
285		$^{1}J(C_3,^{1}H_8)$	199.9902
286		$^{2}J(^{15}N_4,^{1}H_8)$	-10.578
287		$^{2}J(^{15}N_5,^{1}H_8)$	-8.43028
288		$^{4}J(^{1}H_8,^{1}H_6)$	-0.0507
289		$^{4}J(^{1}H8,^{1}H_7)$	1.1781
290		$^{3}J(^{13}C_1,^{1}H_9)$	7.4597
291		$^{2}J(^{13}C_2,^{1}H_9)$	4.2298
292		$^{2}J(^{13}C_3,^{1}H_9)$	4.4963
293		$^{3}J(^{15}N_4,^{1}H_9)$	0.052744
294		$^{1}J(^{15}N,^{1}H)$	-96.9498
295		$^{4}J(^{1}H_9,^{1}H_6)$	1.4397
296		$^{3}J(^{1}H_9,^{1}H_7)$	1.9956
297		$^{3}J(^{1}H_9,^{1}H_8)$	0.9696
298		$^{1}J(^{13}C_1,^{13}C_2)$	61.3972
299	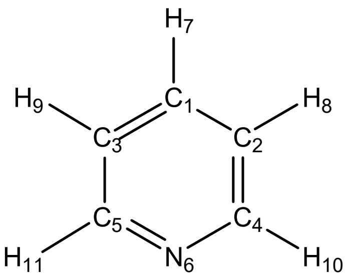	$^{2}J(^{13}C_2,^{13}C_3)$	-4.5112
300		$^{2}J(^{13}C_1,^{13}C_4)$	-4.1667
301		$^{1}J(^{13}C_2,^{13}C_4)$	61.7755
302		$^{3}J(^{13}C_3,^{13}C_4)$	14.774
303		$^{2}J(^{13}C_5,^{13}C_4)$	-7.651
304		$^{3}J(^{15}N_6,^{13}C_1)$	-4.56709
305	pyridine (**37**)	$^{2}J(^{15}N_6,^{13}C_2)$	3.077646
306		$^{1}J(^{15}N,^{13}C)$	-3.52134

307		$^{1}J(^{13}C_1,^{1}H_7)$	155.8606
308		$^{2}J(^{13}C_2,^{1}H_7)$	-1.2139
309		$^{3}J(^{13}C_4,^{1}H_7)$	6.8929
310		$^{4}J(^{15}N,^{1}H)$	0.573446
311		$^{2}J(^{13}C_1,^{1}H_8)$	-1.0724
312		$^{1}J(^{13}C_2,^{1}H_8)$	158.7291
313		$^{3}J(^{13}C_3,^{1}H_8)$	6.769
314		$^{2}J(^{13}C_4,^{1}H_8)$	1.1045
315		$^{4}J(^{13}C_5,^{1}H_8)$	-1.6764
316		$^{3}J(^{15}N,^{1}H)$	-1.77098
317		$^{3}J(^{1}H_7,^{1}H_8)$	7.9414
318		$^{4}J(^{1}H_8,^{1}H_9)$	0.3427
319		$^{3}J(^{13}C_1,^{1}H_{10})$	6.8036
320		$^{2}J(^{13}C_2,^{1}H_{10})$	6.5525
321		$^{4}J(^{13}C_3,^{1}H_{10})$	-2.3494
322		$^{1}J(^{13}C_4,^{1}H_{10})$	172.5175
323		$^{3}J(^{13}C_5,^{1}H_{10})$	11.241
324		$^{2}J(^{15}N,^{1}H)$	-9.11791
325		$^{4}J(^{1}H_7,^{1}H_{10})$	0.915
326		$^{3}J(^{1}H_8,^{1}H_{10})$	5.5716
327		$^{5}J(^{1}H,^{1}H)$	1.4852
328		$^{4}J(^{1}H_{10},^{1}H_{11})$	-0.9883
329		$^{2}J(^{15}N,^{13}C)$	-4.10488
330		$^{1}J(^{13}C_2,^{13}C_3)$	59.3832
331		$^{1}J(^{15}N,^{13}C)$	-17.5645
332		$^{1}J(^{13}C_5,^{13}C_3)$	75.4856
333		$^{2}J(^{13}C_2,^{13}C_5)$	0.5237
334		$^{2}J(^{13}C_4,^{13}C_5)$	7.3119
335		$^{3}J(^{15}N,^{1}H)$	-5.3452
336	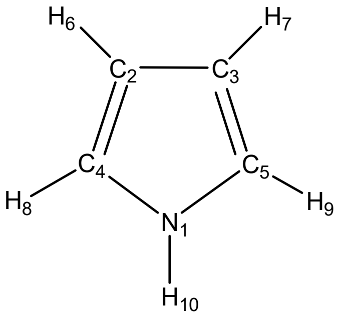	$^{1}J(^{13}C_3,^{1}H_7)$	165.8494
337		$^{2}J(^{13}C_2,^{1}H_7)$	2.7403
338		$^{2}J(^{13}C_5,^{1}H_7)$	6.1831
339		$^{3}J(^{13}C_4,^{1}H_7)$	7.2543
340		$^{3}J(^{1}H_6,^{1}H_7)$	3.5167
341		$^{2}J(^{15}N,^{1}H)$	-3.5508
342		$^{2}J(^{13}C_3,^{1}H_9)$	5.4485
343		$^{3}J(^{13}C_2,^{1}H_9)$	7.583
344	1*H*-pyrrole (**38**)	$^{1}J(^{13}C_5,^{1}H_9)$	178.1767
345		$^{3}J(^{13}C_4,^{1}H_9)$	5.517
346		$^{3}J(^{1}H_7,^{1}H_9)$	3.6903
347		$^{4}J(^{1}H_6,^{1}H_9)$	0.5818
348		$^{4}J(^{1}H_8,^{1}H_9)$	1.864
349		$^{1}J(^{15}N,^{1}H)$	-96.6633
350		$^{3}J(^{13}C_3,^{1}H_{10})$	6.829
351		$^{2}J(^{13}C_5,^{1}H_{10})$	3.4023

352		$^{4}J(^{1}H_{7},^{1}H_{10})$	2.0579
353		$^{3}J(^{1}H_{9},^{1}H_{10})$	2.6506
354		$^{1}J(^{13}C_{2},^{13}C_{1})$	78.7521
355		$^{2}J(^{13}C,^{13}C)$	-0.6523
356		$^{1}J(^{13}C_{2},^{13}C_{3})$	47.467
357		$^{1}J(^{13}C_{1},^{1}H_{4})$	155.3823
358		$^{2}J(^{13}C_{2},^{1}H_{4})$	-3.0119
359		$^{3}J(^{13}C_{3},^{1}H_{4})$	11.9636
360		$^{1}J(^{13}C_{1},^{1}H_{5})$	151.4995
361		$^{2}J(^{13}C_{2},^{1}H_{5})$	-4.1272
362		$^{3}J(^{13}C_{3},^{1}H_{5})$	7.84
363		$^{2}J(^{1}H_{4},^{1}H_{5})$	-1.7286
364		$^{2}J(^{13}C_{1},^{1}H_{6})$	-1.7218
365		$^{1}J(^{13}C_{2},^{1}H_{6})$	148.5187
366		$^{2}J(^{13}C_{3},^{1}H_{6})$	3.4173
367	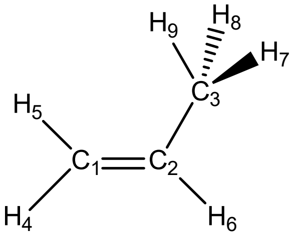	$^{3}J(^{1}H_{4},^{1}H_{6})$	10.9667
368		$^{3}J(^{1}H_{5},^{1}H_{6})$	16.2882
369		$^{3}J(^{13}C_{1},^{1}H_{7})$	6.4016
370		$^{2}J(^{13}C_{2},^{1}H_{7})$	-9.0233
371	prop-1-ene (**39**)	$^{1}J(^{13}C_{3},^{1}H_{7})$	122.9862
372		$^{4}J(^{1}H_{4},^{1}H_{7})$	-3.0822
373		$^{4}J(^{1}H_{5},^{1}H_{7})$	-3.3357
374		$^{3}J(^{1}H_{6},^{1}H_{7})$	4.0585
375		$^{2}J(^{1}H_{7},^{1}H_{8})$	-20.2683
376		$^{3}J(^{13}C_{1},^{1}H_{9})$	6.2935
377		$^{2}J(^{13}C_{2},^{1}H_{9})$	-6.4814
378		$^{1}J(^{13}C_{3},^{1}H_{9})$	123.9559
379		$^{4}J(^{1}H_{4},^{1}H_{9})$	-0.5437
380		$^{4}J(^{1}H_{5},^{1}H_{9})$	-0.6176
381		$^{3}J(^{1}H_{6},^{1}H_{9})$	11.7308
382		$^{2}J(^{1}H_{7},^{1}H_{9})$	-15.4638
383		$^{1}J(^{13}C_{1},^{13}C_{2})$	56.9703
384		$^{1}J(^{13}C_{1},^{13}C_{3})$	78.6352
385		$^{2}J(^{13}C_{2},^{13}C_{3})$	-0.5367
386		$^{2}J(^{13}C_{3},^{13}C_{4})$	3.9791
387	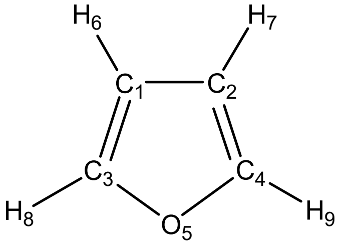	$^{1}J(^{13}C_{1},^{1}H_{6})$	170.1017
388		$^{2}J(^{13}C_{2},^{1}H_{6})$	2.5405
389		$^{2}J(^{13}C_{3},^{1}H_{6})$	7.9644
390		$^{3}J(^{13}C_{4},^{1}H_{6})$	6.6348
391		$^{3}J(^{1}H_{6},^{1}H_{7})$	3.1618
392	furan (**40**)	$^{2}J(^{13}C_{1},^{1}H_{8})$	11.1721
393		$^{3}J(^{13}C_{2},^{1}H_{8})$	6.3532
394		$^{1}J(^{13}C_{3},^{1}H_{8})$	195.1423
395		$^{3}J(^{13}C_{4},^{1}H_{8})$	6.2227
396		$^{3}J(^{1}H_{6},^{1}H_{8})$	2.9423

397		$^{4}J(^{1}H_{7},^{1}H_{8})$	-0.0046
398		$^{4}J(^{1}H_{8},^{1}H_{9})$	1.3696
399		$^{1}J(^{19}F,^{13}C)$	-229.991
400		$^{2}J(^{19}F,^{13}C)$	22.9515
401		$^{1}J(^{13}C_{2},^{13}C_{3})$	77.3804
402		$^{2}J(^{13}C_{3},^{13}C_{4})$	2.1979
403		$^{3}J(^{19}F,^{13}C)$	4.5193
404		$^{2}J(^{13}C_{2},^{13}C_{6})$	-1.263
405		$^{1}J(^{13}C_{3},^{13}C_{6})$	64.5743
406		$^{3}J(^{13}C_{4},^{13}C_{6})$	7.8273
407		$^{2}J(^{13}C_{5},^{13}C_{6})$	-2.9671
408		$^{4}J(^{19}F,^{13}C)$	6.3824
409		$^{3}J(^{13}C_{7},^{13}C_{2})$	11.3892
410		$^{2}J(^{13}C_{3},^{13}C_{7})$	-4.3816
411		$^{1}J(^{13}C_{6},^{13}C_{7})$	63.8793
412		$^{3}J(^{19}F,^{1}H)$	6.3859
413	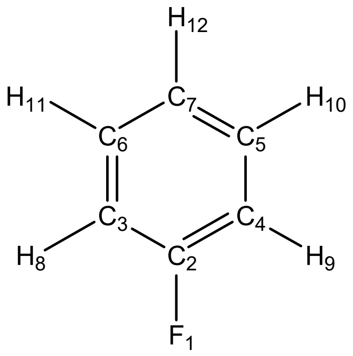	$^{2}J(^{13}C_{2},^{1}H_{8})$	-6.834
414		$^{1}J(^{13}C_{3},^{1}H_{8})$	159.6437
415		$^{3}J(^{13}C_{4},^{1}H_{8})$	4.6234
416		$^{2}J(^{13}C_{6},^{1}H_{8})$	-2.1368
417		$^{4}J(^{13}C_{5},^{1}H_{8})$	-1.5741
418		$^{3}J(^{13}C_{7},^{1}H_{8})$	7.8947
419		$^{4}J(^{1}H_{8},^{1}H_{9})$	1.543
420		$^{4}J(^{19}F,^{1}H)$	5.1453
421		$^{3}J(^{13}C_{2},^{1}H_{11})$	10.6257
422	1-fluorobenzene (**41**)	$^{2}J(^{13}C_{3},^{1}H_{11})$	-1.0677
423		$^{4}J(^{13}C_{4},^{1}H_{11})$	-2.192
424		$^{1}J(^{13}C_{6},^{1}H_{11})$	156.3234
425		$^{3}J(^{13}C_{5},^{1}H_{11})$	8.9889
426		$^{2}J(^{13}C_{7},^{1}H_{11})$	-1.2838
427		$^{3}J(^{1}H_{8},^{1}H_{11})$	8.6603
428		$^{5}J(^{1}H_{9},^{1}H_{11})$	1.0614
429		$^{4}J(^{1}H_{10},^{1}H_{11})$	0.6426
430		$^{5}J(^{19}F,^{1}H)$	-1.7883
431		$^{4}J(^{13}C_{2},^{1}H_{12})$	-2.6033
432		$^{3}J(^{13}C_{3},^{1}H_{12})$	8.3107
433		$^{2}J(^{13}C_{6},^{1}H_{12})$	-0.4215
434		$^{1}J(^{13}C_{7},^{1}H_{12})$	157.9449
435		$^{4}J(^{1}H_{8},^{1}H_{12})$	0.114
436		$^{3}\mathrm{J}(^{1}H_{11},^{1}H_{12})$	7.9393

Table S11. Symmetry independent values of SSCC (in Hz) in molecules of set 1 calculated at the SOPPA(CCSD) level with the pcJ-2 basis set.

#	Molecule	Type of SSCC [1]	SSCC value
1	$H_2C{=}C{=}CH_2$ propa-1,2-diene (**1**)	${}^{1}J({}^{13}C,{}^{13}C)$	103.0880
2		${}^{2}J({}^{13}C,{}^{13}C)$	8.3612
3		${}^{2}J({}^{13}C,{}^{1}H)$	-5.5988
4		${}^{1}J({}^{13}C,{}^{1}H)$	165.5152
5		${}^{3}J({}^{13}C,{}^{1}H)$	7.7102
6		${}^{2}J({}^{1}H,{}^{1}H)$	-13.8380
7		${}^{4}J({}^{1}H,{}^{1}H)$	-8.6965
8	$F_2C{=}CF_2$ Perfluoroethene (**2**)	${}^{1}J({}^{13}C,{}^{13}C)$	196.5684
9		${}^{1}J({}^{13}C,{}^{19}F)$	-262.0901
10		${}^{2}J({}^{13}C,{}^{19}F)$	50.1214
11		${}^{2}J({}^{19}F,{}^{19}F)$	117.2692
12		${}^{3}J_{cis}({}^{19}F,{}^{19}F)$	78.5459
13		${}^{3}J_{trans}({}^{19}F,{}^{19}F)$	-113.9957
14	$HC{\equiv}CH$ ethyne (**3**)	${}^{1}J({}^{13}C,{}^{13}C)$	188.3985
15		${}^{1}J({}^{13}C,{}^{1}H)$	249.8618
16		${}^{2}J({}^{13}C,{}^{1}H)$	51.4364
17		${}^{3}J({}^{1}H,{}^{1}H)$	10.8530
18	$H_2C{=}CH_2$ ethene (**4**)	${}^{1}J({}^{13}C,{}^{13}C)$	70.5298
19		${}^{1}J({}^{13}C,{}^{1}H)$	153.0802
20		${}^{2}J({}^{13}C,{}^{1}H)$	-3.0273
21		${}^{3}J_{cis}({}^{1}H,{}^{1}H)$	12.1869
22		${}^{2}J({}^{1}H,{}^{1}H)$	0.2156
23		${}^{3}J_{trans}({}^{1}H,{}^{1}H)$	18.2769
24	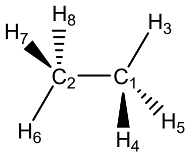 ethane (**5**)	${}^{1}J({}^{13}C,{}^{13}C)$	34.8741
25		${}^{1}J({}^{13}C,{}^{1}H)$	120.9650
26		${}^{2}J({}^{13}C,{}^{1}H)$	-4.9474
27		${}^{3}J_{trans}({}^{1}H_3,{}^{1}H_6)$	15.6904
28		${}^{2}J({}^{1}H,{}^{1}H)$	-14.1095
29		${}^{3}J_{gauche}({}^{1}H_4,{}^{1}H_6)$	3.7704
30	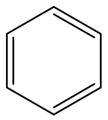 benzene (**6**)	${}^{3}J({}^{13}C,{}^{13}C)$	10.9292
31		${}^{1}J({}^{13}C,{}^{13}C)$	58.7813
32		${}^{2}J({}^{13}C,{}^{13}C)$	-3.3691
33		${}^{1}J({}^{13}C,{}^{1}H)$	154.4313
34		${}^{4}J({}^{13}C,{}^{1}H)$	-1.6901
35		${}^{2}J({}^{13}C,{}^{1}H)$	-0.0327
36		${}^{3}J({}^{13}C,{}^{1}H)$	7.6844
37		${}^{3}J({}^{1}H,{}^{1}H)$	8.0331
38		${}^{4}J({}^{1}H,{}^{1}H)$	0.7936
39		${}^{5}J({}^{1}H,{}^{1}H)$	1.1326
40	CF_4 perfluoromethane (**7**)	${}^{1}J({}^{19}F,{}^{13}C)$	-251.1481
41		${}^{2}J({}^{19}F,{}^{19}F)$	35.8271

42		$^{1}J(^{13}C,^{13}C)$	87.3091
43		$^{2}J(^{19}F,^{13}C)$	11.7657
44		$^{1}J(^{19}F,^{13}C)$	-254.9397
45		$^{1}J(^{13}C_1,^{1}H_4)$	158.1231
46		$^{2}J(^{13}C_2,^{1}H_4)$	6.9475
47		$^{3}J_{trans}(^{19}F_3,^{1}H_4)$	41.0053
48	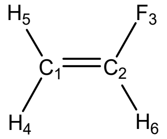	$^{1}J(^{13}C_1,^{1}H_5)$	156.1481
49		$^{2}J(^{13}C_2,^{1}H_5)$	-9.9342
50		$^{3}J_{cis}(^{19}F_3,^{1}H_5)$	13.5696
51	fluoroethene (**8**)	$^{2}J(^{1}H,^{1}H)$	-4.6898
52		$^{2}J(^{13}C_1,^{1}H_6)$	13.0473
53		$^{1}J(^{13}C_2,^{1}H_6)$	191.9210
54		$^{2}J(^{19}F,^{1}H)$	82.5209
55		$^{3}J_{cis}(^{1}H_6,^{1}H_4)$	5.9579
56		$^{3}J_{trans}(^{1}H_6,^{1}H_5)$	12.7184
57		$^{1}J(^{19}F,^{13}C)$	-221.5444
58	CH_2F_2	$^{2}J(^{19}F,^{19}F)$	318.2561
59	difluoromethane (**9**)	$^{1}J(^{13}C,^{1}H)$	173.5823
60		$^{2}J(^{19}F,^{1}H)$	50.1347
61		$^{2}J(^{1}H,^{1}H)$	0.9833
62		$^{1}J(^{15}N,^{13}C)$	-3.9384
63		$^{1}J(^{13}C_1,^{1}H_3)$	170.3417
64		$^{2}J(^{15}N_2,^{1}H_3)$	-9.3725
65		$^{1}J(^{13}C_1,^{1}H_4)$	154.6272
66	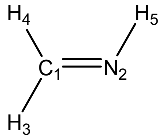	$^{2}J(^{15}N_2,^{1}H_4)$	4.0775
67		$^{2}J(^{1}H,^{1}H)$	16.6219
68	methanimine (**10**)	$^{2}J(^{13}C,^{1}H)$	-13.3395
69		$^{1}J(^{15}N,^{1}H)$	-50.5999
70		$^{3}J_{trans}(^{1}H_5,^{1}H_3)$	24.3866
71		$^{3}J_{cis}(^{1}H_5,^{1}H_4)$	18.0984
72		$^{1}J(^{13}C_1,^{13}C_2)$	189.2812
73		$^{2}J(^{13}C,^{13}C)$	12.8919
74		$^{1}J(^{13}C_2,^{13}C_3)$	70.3275
75		$^{1}J(^{13}C_1,^{1}H_4)$	248.6273
76		$^{2}J(^{13}C_2,^{1}H_4)$	51.2950
77	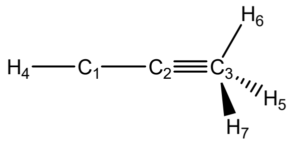	$^{3}J(^{13}C_3,^{1}H_4)$	4.1435
78		$^{3}J(^{13}C_1,^{1}H_6)$	3.6621
79	prop-1-yne (**11**)	$^{2}J(^{13}C_2,^{1}H_6)$	-11.6268
80		$^{1}J(^{13}C_3,^{1}H_6)$	127.2219
81		$^{4}J(^{1}H,^{1}H)$	-3.6537
82		$^{2}J(^{1}H,^{1}H)$	-17.3687
83		$^{1}J(^{13}C,^{13}C)$	41.6464
84		$^{1}J(^{13}C_1,^{1}H_4)$	164.5903
85		$^{2}J(^{13}C_2,^{1}H_4)$	26.3127
86		$^{2}J(^{13}C_1,^{1}H_5)$	-8.3441

87	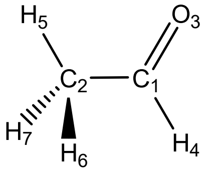 acetaldehyde (**12**)	$^{1}J(^{13}C_2,^{1}H_5)$	130.2996
88		$^{3}J_{trans}(^{1}H_5,^{1}H_4)$	8.1275
89		$^{2}J(^{13}C_1,^{1}H_6)$	-6.3620
90		$^{1}J(^{13}C_2,^{1}H_6)$	119.3152
91		$^{3}J_{gauche}(^{1}H_6,^{1}H_4)$	0.3514
92		$^{2}J(^{1}H_6,^{1}H_5)$	-13.4806
93		$^{2}J(^{1}H_6,^{1}H_7)$	-20.1211
94	$N{\equiv}C{-}CH_3$ acetonitrile (**13**)	$^{1}J(^{13}C,^{13}C)$	62.0638
95		$^{2}J(^{15}N,^{13}C)$	2.7314
96		$^{1}J(^{15}N,^{13}C)$	-17.4320
97		$^{1}J(^{13}C,^{1}H)$	130.4796
98		$^{2}J(^{13}C,^{1}H)$	-10.8544
99		$^{3}J(^{15}N,^{1}H)$	-1.5216
100		$^{2}J(^{1}H,^{1}H)$	-17.4095
101	$H_3C{-}F$ fluoromethane (**14**)	$^{1}J(^{19}F,^{13}C)$	-157.3321
102		$^{1}J(^{13}C,^{1}H)$	142.5841
103		$^{2}J(^{19}F,^{1}H)$	48.3392
104		$^{2}J(^{1}H,^{1}H)$	-10.8501
105	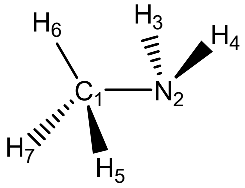 methanamine (**15**)	$^{1}J(^{15}N,^{13}C)$	-5.8533
106		$^{2}J(^{13}C,^{1}H)$	-3.5788
107		$^{1}J(^{15}N,^{1}H)$	-65.1553
108		$^{2}J(^{1}H_3,^{1}H_4)$	-10.7564
109		$^{1}J(^{13}C_1,^{1}H_5)$	128.6482
110		$^{2}J(^{15}N_2,^{1}H_5)$	-1.4721
111		$^{3}J(^{1}H_5,^{1}H_4)$	2.7225
112		$^{3}J(^{1}H_5,^{1}H_3)$	15.5486
113		$^{2}J(^{1}H_7,^{1}H_5)$	-15.7875
114		$^{1}J(^{13}C_1,^{1}H_6)$	125.7580
115		$^{2}J(^{15}N_2,^{1}H_6)$	1.0004
116		$^{3}J(^{1}H_6,^{1}H_4)$	2.1498
117		$^{2}J(^{1}H_6,^{1}H_5)$	-11.5533
118	CH_4 methane (**16**)	$^{1}J(^{13}C,^{1}H)$	121.1588
119		$^{2}J(^{1}H,^{1}H)$	-13.9091
120	CHF_3 fluoroform (**17**)	$^{1}J(^{13}C,^{1}H)$	223.0219
121		$^{1}J(^{19}F,^{13}C)$	-256.3920
122		$^{2}J(^{19}F,^{1}H)$	74.5838
123		$^{2}J(^{19}F,^{19}F)$	128.5157
124	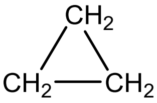 cyclopropane (**18**)	$^{1}J(^{13}C,^{13}C)$	13.4244
125		$^{1}J(^{13}C,^{1}H)$	155.6649
126		$^{2}J(^{13}C,^{1}H)$	-2.9379
127		$^{2}J(^{1}H,^{1}H)$	-5.9730
128		$^{3}J_{cis}(^{1}H,^{1}H)$	9.0198
129		$^{3}J_{trans}(^{1}H,^{1}H)$	5.1069
130	$F{-}C{\equiv}C{-}F$ 1,2-difluoroethyne (**19**)	$^{1}J(^{13}C,^{13}C)$	407.4795
131		$^{1}J(^{19}F,^{13}C)$	-274.3065

132		$^{2}J(^{19}F,^{13}C)$	40.1805
133		$^{3}J(^{19}F,^{19}F)$	-5.0857
134	F−C≡N<br>fluoroformonitrile (**20**)	$^{1}J(^{19}F,^{13}C)$	-401.8478
135		$^{1}J(^{15}N,^{13}C)$	-3.3165
136		$^{2}J(^{19}F,^{15}N)$	51.5032
137	$H_2C{=}CF_2$<br>1,1-difluoroethene (**21**)	$^{1}J(^{13}C,^{13}C)$	116.6755
138		$^{2}J(^{19}F,^{13}C)$	27.7571
139		$^{1}J(^{19}F,^{13}C)$	-281.3949
140		$^{2}J(^{19}F,^{19}F)$	25.0739
141		$^{1}J(^{13}C,^{1}H)$	163.1442
142		$^{2}J(^{13}C,^{1}H)$	-1.9402
143		$^{3}J_{cis}(^{19}F,^{1}H)$	-1.4427
144		$^{3}J_{trans}(^{19}F,^{1}H)$	27.7233
145		$^{2}J(^{1}H,^{1}H)$	-6.4062
146	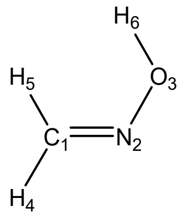<br>formaldehyde oxime (*Z*) (**22**)	$^{1}J(^{15}N,^{13}C)$	-2.7944
147		$^{1}J(^{13}C_1,^{1}H_4)$	182.8774
148		$^{2}J(^{15}N_2,^{1}H_4)$	-12.1852
149		$^{1}J(^{13}C_1,^{1}H_5)$	152.2572
150		$^{2}J(^{15}N_2,^{1}H_5)$	3.0642
151		$^{2}J(^{1}H,^{1}H)$	6.3122
152		$^{3}J(^{13}C,^{1}H)$	4.0603
153		$^{2}J(^{15}N_2,^{1}H_6)$	1.4001
154		$^{4}J(^{1}H_6,^{1}H_4)$	-1.2774
155		$^{4}J(^{1}H_6,^{1}H_5)$	1.1196
156	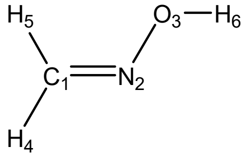<br>formaldehyde oxime (*E*) (**23**)	$^{1}J(^{15}N,^{13}C)$	-5.8827
157		$^{1}J(^{13}C_1,^{1}H_4)$	175.8535
158		$^{2}J(^{15}N_2,^{1}H_4)$	-12.9587
159		$^{1}J(^{13}C_1,^{1}H_5)$	161.8443
160		$^{2}J(^{15}N_2,^{1}H_5)$	3.0785
161		$^{2}J(^{1}H,^{1}H)$	7.9617
162		$^{3}J(^{13}C,^{1}H)$	10.7595
163		$^{2}J(^{15}N_2,^{1}H_6)$	-1.8654
164		$^{4}J(^{1}H_6,^{1}H_4)$	1.3364
165		$^{4}J(^{1}H_6,^{1}H_5)$	-0.4483
166	H−C≡C−F<br>fluoroethyne (**24**)	$^{1}J(^{13}C,^{13}C)$	273.0040
167		$^{2}J(^{19}F,^{13}C)$	23.2789
168		$^{1}J(^{19}F,^{13}C)$	-288.6317
169		$^{1}J(^{13}C,^{1}H)$	278.1154
170		$^{2}J(^{13}C,^{1}H)$	65.9845
171		$^{3}J(^{19}F,^{1}H)$	11.3875
172	H−C≡N<br>hydrogen cyanide (**25**)	$^{1}J(^{15}N,^{13}C)$	-18.1388
173		$^{1}J(^{13}C,^{1}H)$	260.9722
174		$^{2}J(^{15}N,^{1}H)$	-8.4355
175		$^{1}J(^{13}C,^{13}C)$	144.6301
176		$^{2}J(^{19}F_3,^{13}C_1)$	65.3553

177	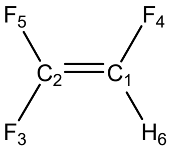 1,1,2-trifluoroethene (**26**)	$^{1}J(^{19}F_3,^{13}C_2)$	-267.7437
178		$^{1}J(^{19}F_4,^{13}C_1)$	-228.6128
179		$^{2}J(^{19}F_4,^{13}C_2)$	36.5435
180		$^{3}J_{trans}(^{19}F_3,^{19}F_4)$	-119.9465
181		$^{2}J(^{19}F_5,^{13}C_1)$	20.0977
182		$^{1}J(^{19}F_5,^{13}C_2)$	-280.4840
183		$^{2}J(^{19}F,^{19}F)$	74.6324
184		$^{3}J_{cis}(^{19}F_4,^{19}F_5)$	40.0509
185		$^{1}J(^{13}C,^{1}H)$	203.0268
186		$^{2}J(^{13}C,^{1}H)$	14.1942
187		$^{3}J_{cis}(^{19}F_3,^{1}H_6)$	-5.4724
188		$^{2}J(^{19}F,^{1}H)$	72.5096
189		$^{3}J_{trans}(^{19}F_5,^{1}H_6)$	8.2161
190	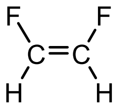 (*Z*)-1,2-difluoroethene (**27**)	$^{1}J(^{13}C,^{13}C)$	100.8699
191		$^{1}J(^{19}F,^{13}C)$	-248.5836
192		$^{2}J(^{19}F,^{13}C)$	9.3763
193		$^{3}J(^{19}F,^{19}F)$	-11.2034
194		$^{1}J(^{13}C,^{1}H)$	196.1262
195		$^{2}J(^{13}C,^{1}H)$	22.8195
196		$^{2}J(^{19}F,^{1}H)$	72.5226
197		$^{3}J(^{19}F,^{1}H)$	14.1716
198		$^{3}J(^{1}H,^{1}H)$	3.5072
199	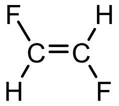 (*E*)-1,2-difluoroethene (**28**)	$^{1}J(^{13}C,^{13}C)$	114.7429
200		$^{1}J(^{19}F,^{13}C)$	-235.8600
201		$^{2}J(^{19}F,^{13}C)$	49.7363
202		$^{3}J(^{19}F,^{19}F)$	-132.4367
203		$^{2}J(^{13}C,^{1}H)$	4.4589
204		$^{1}J(^{13}C,^{1}H)$	194.5098
205		$^{3}J(^{19}F,^{1}H)$	0.4950
206		$^{2}J(^{19}F,^{1}H)$	76.3005
207		$^{3}J(^{1}H,^{1}H)$	9.9515
208	HF hydrogen fluoride (**29**)	$^{1}J(^{19}F,^{1}H)$	539.7412
209	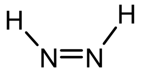 diazene (*Z*) (**31**)	$^{1}J(^{15}N,^{15}N)$	-20.7439
210		$^{1}J(^{15}N,^{1}H)$	-35.5945
211		$^{2}J(^{15}N,^{1}H)$	1.4050
212		$^{3}J(^{1}H,^{1}H)$	37.4751
213	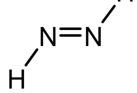 diazene (*E*) (**30**)	$^{1}J(^{15}N,^{15}N)$	-21.2719
214		$^{2}J(^{15}N,^{1}H)$	-0.0477
215		$^{1}J(^{15}N,^{1}H)$	-45.9625
216		$^{3}J(^{1}H,^{1}H)$	37.3121
217		$^{1}J(^{15}N,^{15}N)$	1.0118
218		$^{1}J(^{15}N,^{1}H)$	-59.1852
219		$^{2}J(^{15}N,^{1}H)$	-1.6212
220		$^{2}J(^{1}H,^{1}H)$	-15.3026

221	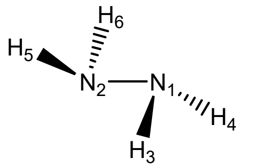	$^{3}J(^{1}H_5,^{1}H_3)$	1.5386
222	hydrazine (**32**)	$^{3}J(^{1}H_4,^{1}H_5)$	13.3884
223	N_2 molecular nitrogen (**33**)	$^{1}J(^{15}N,^{15}N)$	-3.27232
224	NH_3	$^{1}J(^{15}N,^{1}H)$	-61.6971
225	ammonia (**34**)	$^{2}J(^{1}H,^{1}H)$	-11.2174
226		$^{1}J(^{13}C_1,^{13}C_2)$	54.5631
227		$^{1}J(^{13}C_3,^{13}C_1)$	69.09
228		$^{2}J(^{13}C_3,^{13}C_2)$	1.8023
229		$^{2}J(^{15}N_4,^{13}C_1)$	3.006246
230		$^{1}J(^{15}N_4,^{13}C_2)$	-2.17259
231		$^{2}J(^{15}N_4,^{13}C_3)$	0.983191
232		$^{2}J(^{15}N_5,^{13}C_1)$	-5.71567
233		$^{2}J(^{15}N_5,^{13}C_2)$	-0.75118
234		$^{1}J(^{15}N_5,^{13}C_3)$	-15.337
235		$^{1}J(^{15}N,^{15}N)$	-11.7959
236		$^{1}J(^{13}C_1,^{1}H_6)$	171.5362
237		$^{2}J(^{13}C_2,^{1}H_6)$	5.1726
238		$^{2}J(^{13}C3,^{1}H_6)$	7.6565
239		$^{3}J(^{15}N_4,^{1}H_6)$	-1.11645
240	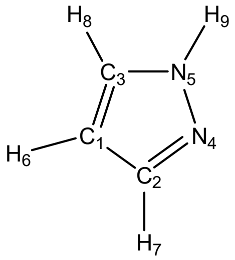	$^{3}J(^{15}N_5,^{1}H_6)$	-5.72591
241		$^{2}J(^{13}C_1,^{1}H_7)$	10.584
242		$^{1}J(^{13}C_2,^{1}H_7)$	180.8626
243		$^{3}J(^{13}C_3,^{1}H_7)$	4.6913
244		$^{2}J(^{15}N_4,^{1}H_7)$	-11.9064
245		$^{3}J(^{15}N_5,^{1}H_7)$	-8.76049
246		$^{3}J(^{1}H_7,^{1}H_6)$	1.8669
247	1*H*-pyrazole (**35**)	$^{2}J(^{13}C_1,^{1}H_8)$	6.8783
248		$^{3}J(^{13}C_2,^{1}H_8)$	7.8221
249		$^{1}J(^{13}C_3,^{1}H_8)$	179.3762
250		$^{3}J(^{15}N_4,^{1}H_8)$	0.272696
251		$^{2}J(^{15}N_5,^{1}H_8)$	-4.50467
252		$^{3}J(^{1}H_8,^{1}H_6)$	3.0461
253		$^{4}J(^{1}H_8,^{1}H_7)$	0.1591
254		$^{3}J(^{13}C_1,^{1}H_9)$	5.4502
255		$^{3}J(^{13}C_2,^{1}H_9)$	10.19
256		$^{2}J(^{13}C_3,^{1}H_9)$	9.1397
257		$^{2}J(^{15}N_4,^{1}H_9)$	-8.55779
258		$^{1}J(^{15}N,^{1}H)$	-106.483
259		$^{4}J(^{1}H_9,^{1}H_6)$	2.0146
260		$^{4}J(^{1}H_9,^{1}H_7)$	1.9107
261		$^{3}J(^{1}H_9,^{1}H_8)$	1.6933

262		$^{1}J(^{13}C,^{13}C)$	70.9612
263		$^{2}J(^{13}C_{1},^{13}C_{3})$	-4.3501
264		$^{2}J(^{13}C_{3},^{13}C_{2})$	9.0118
265		$^{1}J(^{15}N_{4},^{13}C_{1})$	1.195989
266		$^{2}J(^{15}N_{4},^{13}C_{2})$	2.536463
267		$^{1}J(^{15}N_{4},^{13}C_{3})$	-2.64237
268		$^{2}J(^{15}N_{5},^{13}C_{1})$	-6.23974
269		$^{1}J(^{15}N_{5},^{13}C_{2})$	-16.769
270		$^{1}J(^{15}N_{5},^{13}C_{3})$	-13.1543
271		$^{2}J(^{15}N,^{15}N)$	-1.57319
272		$^{1}J(^{13}C_{1},^{1}H_{6})$	184.6453
273		$^{2}J(^{13}C_{2},^{1}H_{6})$	15.5825
274		$^{3}J(^{13}C_{3},^{1}H_{6})$	11.4484
275		$^{2}J(^{15}N_{4},^{1}H_{6})$	-9.70959
276	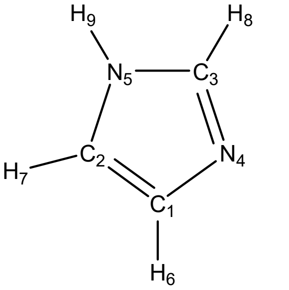	$^{3}J(^{15}N_{5},^{1}H_{6})$	-3.55458
277		$^{2}J(^{13}C_{1},^{1}H_{7})$	8.2125
278		$^{1}J(^{13}C_{2},^{1}H_{7})$	183.2173
279		$^{3}J(^{13}C_{3},^{1}H_{7})$	6.7153
280		$^{3}J(^{15}N_{4},^{1}H_{7})$	-1.14016
281		$^{2}J(^{15}N_{5},^{1}H_{7})$	-4.59332
282		$^{3}J(^{1}H_{7},^{1}H_{6})$	2.1235
283	1*H*-imidazole (**36**)	$^{3}J(^{13}C_{1},^{1}H_{8})$	11.2715
284		$^{3}J(C_{2},^{1}H_{8})$	3.301
285		$^{1}J(C_{3},^{1}H_{8})$	200.6392
286		$^{2}J(^{15}N_{4},^{1}H_{8})$	-11.1143
287		$^{2}J(^{15}N_{5},^{1}H_{8})$	-8.86724
288		$^{4}J(^{1}H_{8},^{1}H_{6})$	0.2811
289		$^{4}J(^{1}H8,^{1}H_{7})$	1.371
290		$^{3}J(^{13}C_{1},^{1}H_{9})$	7.7896
291		$^{2}J(^{13}C_{2},^{1}H_{9})$	4.4549
292		$^{2}J(^{13}C_{3},^{1}H_{9})$	4.6749
293		$^{3}J(^{15}N_{4},^{1}H_{9})$	0.118673
294		$^{1}J(^{15}N,^{1}H)$	-96.3769
295		$^{4}J(^{1}H_{9},^{1}H_{6})$	1.7492
296		$^{3}J(^{1}H_{9},^{1}H_{7})$	2.1011
297		$^{3}J(^{1}H_{9},^{1}H_{8})$	0.9989
298		$^{1}J(^{13}C_{1},^{13}C_{2})$	56.7555
299	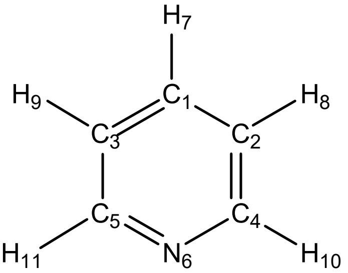	$^{2}J(^{13}C_{2},^{13}C_{3})$	-4.0482
300		$^{2}J(^{13}C_{1},^{13}C_{4})$	-3.5992
301		$^{1}J(^{13}C_{2},^{13}C_{4})$	57.4862
302		$^{3}J(^{13}C_{3},^{13}C_{4})$	15.0451
303		$^{2}J(^{13}C_{5},^{13}C_{4})$	-7.1449
304		$^{3}J(^{15}N_{6},^{13}C_{1})$	-4.57565
305	pyridine (**37**)	$^{2}J(^{15}N_{6},^{13}C_{2})$	2.996146
306		$^{1}J(^{15}N,^{13}C)$	-1.74853

307		$^{1}J(^{13}C_1,^{1}H_7)$	155.3621
308		$^{2}J(^{13}C_2,^{1}H_7)$	-0.3457
309		$^{3}J(^{13}C_4,^{1}H_7)$	6.7753
310		$^{4}J(^{15}N,^{1}H)$	0.520843
311		$^{2}J(^{13}C_1,^{1}H_8)$	-0.2844
312		$^{1}J(^{13}C_2,^{1}H_8)$	158.1305
313		$^{3}J(^{13}C_3,^{1}H_8)$	6.6312
314		$^{2}J(^{13}C_4,^{1}H_8)$	1.9521
315		$^{4}J(^{13}C_5,^{1}H_8)$	-1.2836
316		$^{3}J(^{15}N,^{1}H)$	-1.63631
317		$^{3}J(^{1}H_7,^{1}H_8)$	8.0222
318		$^{4}J(^{1}H_8,^{1}H_9)$	0.8082
319		$^{3}J(^{13}C_1,^{1}H_{10})$	6.6195
320		$^{2}J(^{13}C_2,^{1}H_{10})$	7.5044
321		$^{4}J(^{13}C_3,^{1}H_{10})$	-2.0436
322		$^{1}J(^{13}C_4,^{1}H_{10})$	172.3832
323		$^{3}J(^{13}C_5,^{1}H_{10})$	11.245
324		$^{2}J(^{15}N,^{1}H)$	-9.77763
325		$^{4}J(^{1}H_7,^{1}H_{10})$	1.319
326		$^{3}J(^{1}H_8,^{1}H_{10})$	5.4435
327		$^{5}J(^{1}H,^{1}H)$	1.376
328		$^{4}J(^{1}H_{10},^{1}H_{11})$	-0.6429
329		$^{2}J(^{15}N,^{13}C)$	-4.36748
330		$^{1}J(^{13}C_2,^{13}C_3)$	54.7613
331		$^{1}J(^{15}N,^{13}C)$	-15.923
332		$^{1}J(^{13}C_5,^{13}C_3)$	70.5819
333		$^{2}J(^{13}C_2,^{13}C_5)$	1.1024
334		$^{2}J(^{13}C_4,^{13}C_5)$	7.4517
335		$^{3}J(^{15}N,^{1}H)$	-5.45405
336	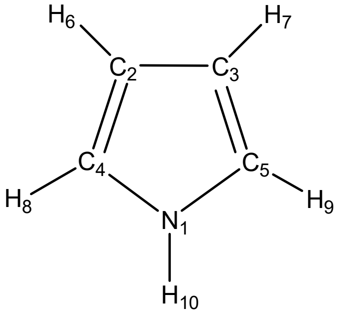	$^{1}J(^{13}C_3,^{1}H_7)$	166.3781
337		$^{2}J(^{13}C_2,^{1}H_7)$	3.3784
338		$^{2}J(^{13}C_5,^{1}H_7)$	7.077
339		$^{3}J(^{13}C_4,^{1}H_7)$	7.2851
340		$^{3}J(^{1}H_6,^{1}H_7)$	3.6287
341		$^{2}J(^{15}N,^{1}H)$	-3.99042
342		$^{2}J(^{13}C_3,^{1}H_9)$	6.2682
343		$^{3}J(^{13}C_2,^{1}H_9)$	7.5743
344	1*H*-pyrrole (**38**)	$^{1}J(^{13}C_5,^{1}H_9)$	178.6231
345		$^{3}J(^{13}C_4,^{1}H_9)$	5.9173
346		$^{3}J(^{1}H_7,^{1}H_9)$	3.6358
347		$^{4}J(^{1}H_6,^{1}H_9)$	0.9668
348		$^{4}J(^{1}H_8,^{1}H_9)$	2.1264
349		$^{1}J(^{15}N,^{1}H)$	-96.2135
350		$^{3}J(^{13}C_3,^{1}H_{10})$	7.0482
351		$^{2}J(^{13}C_5,^{1}H_{10})$	3.6175

352		${}^{4}J({}^{1}H_{7},{}^{1}H_{10})$	2.4945
353		${}^{3}J({}^{1}H_{9},{}^{1}H_{10})$	2.7869
354		${}^{1}J({}^{13}C_{2},{}^{13}C_{1})$	73.0301
355		${}^{2}J({}^{13}C,{}^{13}C)$	-0.0145
356		${}^{1}J({}^{13}C_{2},{}^{13}C_{3})$	43.6435
357		${}^{1}J({}^{13}C_{1},{}^{1}H_{4})$	154.1921
358		${}^{2}J({}^{13}C_{2},{}^{1}H_{4})$	-1.8538
359		${}^{3}J({}^{13}C_{3},{}^{1}H_{4})$	11.8379
360		${}^{1}J({}^{13}C_{1},{}^{1}H_{5})$	150.3871
361		${}^{2}J({}^{13}C_{2},{}^{1}H_{5})$	-3.3621
362		${}^{3}J({}^{13}C_{3},{}^{1}H_{5})$	7.651
363		${}^{2}J({}^{1}H_{4},{}^{1}H_{5})$	0.0202
364		${}^{2}J({}^{13}C_{1},{}^{1}H_{6})$	-0.6417
365		${}^{1}J({}^{13}C_{2},{}^{1}H_{6})$	148.0033
366		${}^{2}J({}^{13}C_{3},{}^{1}H_{6})$	4.0351
367		${}^{3}J({}^{1}H_{4},{}^{1}H_{6})$	10.8732
368	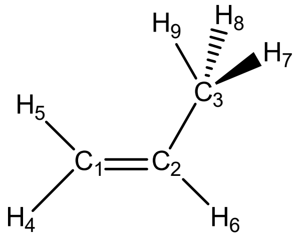	${}^{3}J({}^{1}H_{5},{}^{1}H_{6})$	16.2646
369		${}^{3}J({}^{13}C_{1},{}^{1}H_{7})$	6.1364
370		${}^{2}J({}^{13}C_{2},{}^{1}H_{7})$	-8.0933
371	prop-1-ene (**39**)	${}^{1}J({}^{13}C_{3},{}^{1}H_{7})$	121.7183
372		${}^{4}J({}^{1}H_{4},{}^{1}H_{7})$	-2.7078
373		${}^{4}J({}^{1}H_{5},{}^{1}H_{7})$	-2.9455
374		${}^{3}J({}^{1}H_{6},{}^{1}H_{7})$	3.8916
375		${}^{2}J({}^{1}H_{7},{}^{1}H_{8})$	-19.116
376		${}^{3}J({}^{13}C_{1},{}^{1}H_{9})$	6.3406
377		${}^{2}J({}^{13}C_{2},{}^{1}H_{9})$	-6.0539
378		${}^{1}J({}^{13}C_{3},{}^{1}H_{9})$	122.7732
379		${}^{4}J({}^{1}H_{4},{}^{1}H_{9})$	-0.3366
380		${}^{4}J({}^{1}H_{5},{}^{1}H_{9})$	-0.588
381		${}^{3}J({}^{1}H_{6},{}^{1}H_{9})$	11.9787
382		${}^{2}J({}^{1}H_{7},{}^{1}H_{9})$	-14.1739
383		${}^{1}J({}^{13}C_{1},{}^{13}C_{2})$	52.4983
384		${}^{1}J({}^{13}C_{1},{}^{13}C_{3})$	73.8066
385		${}^{2}J({}^{13}C_{2},{}^{13}C_{3})$	0.0531
386		${}^{2}J({}^{13}C_{3},{}^{13}C_{4})$	4.0308
387		${}^{1}J({}^{13}C_{1},{}^{1}H_{6})$	170.16
388		${}^{2}J({}^{13}C_{2},{}^{1}H_{6})$	3.1751
389	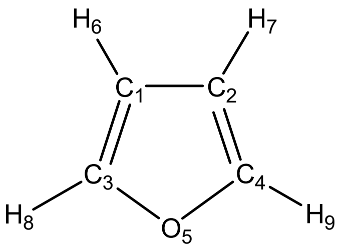	${}^{2}J({}^{13}C_{3},{}^{1}H_{6})$	8.9273
390		${}^{3}J({}^{13}C_{4},{}^{1}H_{6})$	6.6952
391		${}^{3}J({}^{1}H_{6},{}^{1}H_{7})$	3.2439
392		${}^{2}J({}^{13}C_{1},{}^{1}H_{8})$	12.1462
393	furan (**40**)	${}^{3}J({}^{13}C_{2},{}^{1}H_{8})$	6.2711
394		${}^{1}J({}^{13}C_{3},{}^{1}H_{8})$	195.6672
395		${}^{3}J({}^{13}C_{4},{}^{1}H_{8})$	6.6436
396		${}^{3}J({}^{1}H_{6},{}^{1}H_{8})$	2.7943

397		$^{4}J(^{1}H_{7},^{1}H_{8})$	0.3626
398		$^{4}J(^{1}H_{8},^{1}H_{9})$	1.682
399		$^{1}J(^{19}F,^{13}C)$	-243.096
400		$^{2}J(^{19}F,^{13}C)$	21.8139
401		$^{1}J(^{13}C_{2},^{13}C_{3})$	73.2665
402		$^{2}J(^{13}C_{3},^{13}C_{4})$	2.911
403		$^{3}J(^{19}F,^{13}C)$	5.2243
404		$^{2}J(^{13}C_{2},^{13}C_{6})$	-0.4875
405		$^{1}J(^{13}C_{3},^{13}C_{6})$	59.9698
406		$^{3}J(^{13}C_{4},^{13}C_{6})$	7.74
407		$^{2}J(^{13}C_{5},^{13}C_{6})$	-2.3651
408		$^{4}J(^{19}F,^{13}C)$	5.5104
409		$^{3}J(^{13}C_{7},^{13}C_{2})$	11.4648
410		$^{2}J(^{13}C_{3},^{13}C_{7})$	-3.8471
411		$^{1}J(^{13}C_{6},^{13}C_{7})$	59.2197
412		$^{3}J(^{19}F,^{1}H)$	6.4176
413	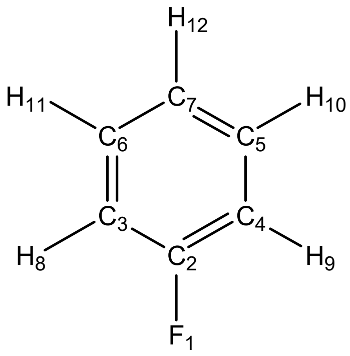	$^{2}J(^{13}C_{2},^{1}H_{8})$	-6.2964
414		$^{1}J(^{13}C_{3},^{1}H_{8})$	158.5215
415		$^{3}J(^{13}C_{4},^{1}H_{8})$	4.434
416		$^{2}J(^{13}C_{6},^{1}H_{8})$	-1.4014
417		$^{4}J(^{13}C_{5},^{1}H_{8})$	-1.2221
418		$^{3}J(^{13}C_{7},^{1}H_{8})$	7.7665
419		$^{4}J(^{1}H_{8},^{1}H_{9})$	2.0744
420		$^{4}J(^{19}F,^{1}H)$	4.938
421		$^{3}J(^{13}C_{2},^{1}H_{11})$	10.6524
422	1-fluorobenzene (**41**)	$^{2}J(^{13}C_{3},^{1}H_{11})$	-0.1461
423		$^{4}J(^{13}C_{4},^{1}H_{11})$	-1.8823
424		$^{1}J(^{13}C_{6},^{1}H_{11})$	155.7343
425		$^{3}J(^{13}C_{5},^{1}H_{11})$	8.9276
426		$^{2}J(^{13}C_{7},^{1}H_{11})$	-0.4411
427		$^{3}J(^{1}H_{8},^{1}H_{11})$	8.7653
428		$^{5}J(^{1}H_{9},^{1}H_{11})$	0.9537
429		$^{4}J(^{1}H_{10},^{1}H_{11})$	1.1464
430		$^{5}J(^{19}F,^{1}H)$	-1.2205
431		$^{4}J(^{13}C_{2},^{1}H_{12})$	-2.2456
432		$^{3}J(^{13}C_{3},^{1}H_{12})$	8.1926
433		$^{2}J(^{13}C_{6},^{1}H_{12})$	0.4519
434		$^{1}J(^{13}C_{7},^{1}H_{12})$	157.4048
435		$^{4}J(^{1}H_{8},^{1}H_{12})$	0.5568
436		$^{3}\mathrm{J}(^{1}H_{11},^{1}H_{12})$	7.9992

Table S12. Symmetry independent values of SSCC (in Hz) in molecules of set 1 calculated at the SOPPA(CCSD) level with the ccJ-pVDZ basis set.

#	Molecule	Type of SSCC [1]	SSCC value
1	$H_2C{=}C{=}CH_2$ propa-1,2-diene (**1**)	$^{1}J(^{13}C,^{13}C)$	109.8868
2		$^{2}J(^{13}C,^{13}C)$	7.7914
3		$^{2}J(^{13}C,^{1}H)$	-6.9923
4		$^{1}J(^{13}C,^{1}H)$	166.7001
5		$^{3}J(^{13}C,^{1}H)$	8.1676
6		$^{2}J(^{1}H,^{1}H)$	-15.0607
7		$^{4}J(^{1}H,^{1}H)$	-9.3468
8	$F_2C{=}CF_2$ Perfluoroethene (**2**)	$^{1}J(^{13}C,^{13}C)$	202.9879
9		$^{1}J(^{13}C,^{19}F)$	-269.9445
10		$^{2}J(^{13}C,^{19}F)$	52.9848
11		$^{2}J(^{19}F,^{19}F)$	125.3996
12		$^{3}J_{cis}(^{19}F,^{19}F)$	91.2190
13		$^{3}J_{trans}(^{19}F,^{19}F)$	-120.2192
14	$HC{\equiv}CH$ ethyne (**3**)	$^{1}J(^{13}C,^{13}C)$	197.6147
15		$^{1}J(^{13}C,^{1}H)$	250.5633
16		$^{2}J(^{13}C,^{1}H)$	49.5888
17		$^{3}J(^{1}H,^{1}H)$	10.7599
18	$H_2C{=}CH_2$ ethene (**4**)	$^{1}J(^{13}C,^{13}C)$	76.2878
19		$^{1}J(^{13}C,^{1}H)$	154.1365
20		$^{2}J(^{13}C,^{1}H)$	-3.9942
21		$^{3}J_{cis}(^{1}H,^{1}H)$	11.8881
22		$^{2}J(^{1}H,^{1}H)$	-1.3232
23		$^{3}J_{trans}(^{1}H,^{1}H)$	17.8100
24	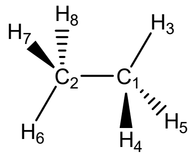 ethane (**5**)	$^{1}J(^{13}C,^{13}C)$	37.5788
25		$^{1}J(^{13}C,^{1}H)$	122.0628
26		$^{2}J(^{13}C,^{1}H)$	-5.4667
27		$^{3}J_{trans}(^{1}H_3,^{1}H_6)$	15.0693
28		$^{2}J(^{1}H,^{1}H)$	-14.8968
29		$^{3}J_{gauche}(^{1}H_4,^{1}H_6)$	3.6462
30	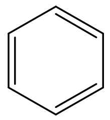 benzene (**6**)	$^{3}J(^{13}C,^{13}C)$	11.0841
31		$^{1}J(^{13}C,^{13}C)$	63.4092
32		$^{2}J(^{13}C,^{13}C)$	-3.8786
33		$^{1}J(^{13}C,^{1}H)$	154.4140
34		$^{4}J(^{13}C,^{1}H)$	-1.9742
35		$^{2}J(^{13}C,^{1}H)$	-0.7315
36		$^{3}J(^{13}C,^{1}H)$	7.7537
37		$^{3}J(^{1}H,^{1}H)$	7.8842
38		$^{4}J(^{1}H,^{1}H)$	0.3832
39		$^{5}J(^{1}H,^{1}H)$	0.9760
40	CF_4 perfluoromethane (**7**)	$^{1}J(^{19}F,^{13}C)$	-257.8896
41		$^{2}J(^{19}F,^{19}F)$	34.3935

42		$^{1}J(^{13}C,^{13}C)$	93.2076
43		$^{2}J(^{19}F,^{13}C)$	13.1302
44		$^{1}J(^{19}F,^{13}C)$	-261.8981
45		$^{1}J(^{13}C_1,^{1}H_4)$	158.5008
46		$^{2}J(^{13}C_2,^{1}H_4)$	5.6522
47		$^{3}J_{trans}(^{19}F_3,^{1}H_4)$	36.6062
48	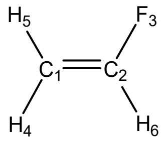	$^{1}J(^{13}C_1,^{1}H_5)$	157.7458
49		$^{2}J(^{13}C_2,^{1}H_5)$	-10.7123
50		$^{3}J_{cis}(^{19}F_3,^{1}H_5)$	11.4127
51	fluoroethene (**8**)	$^{2}J(^{1}H,^{1}H)$	-5.9241
52		$^{2}J(^{13}C_1,^{1}H_6)$	12.1316
53		$^{1}J(^{13}C_2,^{1}H_6)$	190.9219
54		$^{2}J(^{19}F,^{1}H)$	80.8458
55		$^{3}J_{cis}(^{1}H_6,^{1}H_4)$	6.0018
56		$^{3}J_{trans}(^{1}H_6,^{1}H_5)$	12.4661
57		$^{1}J(^{19}F,^{13}C)$	-222.8444
58		$^{2}J(^{19}F,^{19}F)$	334.9153
59	CH_2F_2 difluoromethane (**9**)	$^{1}J(^{13}C,^{1}H)$	173.1462
60		$^{2}J(^{19}F,^{1}H)$	50.3626
61		$^{2}J(^{1}H,^{1}H)$	-0.1541
62		$^{1}J(^{15}N,^{13}C)$	-4.7438
63		$^{1}J(^{13}C_1,^{1}H_3)$	171.0392
64		$^{2}J(^{15}N_2,^{1}H_3)$	-9.3475
65	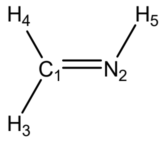	$^{1}J(^{13}C_1,^{1}H_4)$	153.8365
66		$^{2}J(^{15}N_2,^{1}H_4)$	3.7674
67		$^{2}J(^{1}H,^{1}H)$	14.9849
68	methanimine (**10**)	$^{2}J(^{13}C,^{1}H)$	-14.2155
69		$^{1}J(^{15}N,^{1}H)$	-48.4508
70		$^{3}J_{trans}(^{1}H_5,^{1}H_3)$	23.8936
71		$^{3}J_{cis}(^{1}H_5,^{1}H_4)$	17.6757
72		$^{1}J(^{13}C_1,^{13}C_2)$	198.3123
73		$^{2}J(^{13}C,^{13}C)$	12.8524
74		$^{1}J(^{13}C_2,^{13}C_3)$	75.4509
75		$^{1}J(^{13}C_1,^{1}H_4)$	249.0818
76	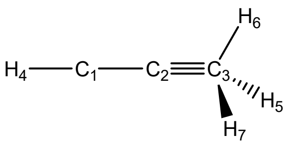	$^{2}J(^{13}C_2,^{1}H_4)$	49.3581
77		$^{3}J(^{13}C_3,^{1}H_4)$	4.3381
78		$^{3}J(^{13}C_1,^{1}H_6)$	3.8060
79	prop-1-yne (**11**)	$^{2}J(^{13}C_2,^{1}H_6)$	-12.5959
80		$^{1}J(^{13}C_3,^{1}H_6)$	128.2289
81		$^{4}J(^{1}H,^{1}H)$	-4.0486
82		$^{2}J(^{1}H,^{1}H)$	-18.0001
83		$^{1}J(^{13}C,^{13}C)$	45.4636
84		$^{1}J(^{13}C_1,^{1}H_4)$	163.3831
85		$^{2}J(^{13}C_2,^{1}H_4)$	26.4722
86		$^{2}J(^{13}C_1,^{1}H_5)$	-8.6876

87	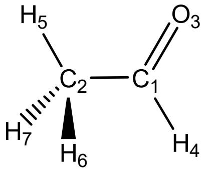 acetaldehyde (**12**)	$^{1}J(^{13}C_{2},^{1}H_{5})$	131.4762
88		$^{3}J_{trans}(^{1}H_{5},^{1}H_{4})$	7.5258
89		$^{2}J(^{13}C_{1},^{1}H_{6})$	-7.0446
90		$^{1}J(^{13}C_{2},^{1}H_{6})$	120.0699
91		$^{3}J_{gauche}(^{1}H_{6},^{1}H_{4})$	0.2725
92		$^{2}J(^{1}H_{6},^{1}H_{5})$	-14.2455
93		$^{2}J(^{1}H_{6},^{1}H_{7})$	-20.6345
94	N≡C−CH_3 acetonitrile (**13**)	$^{1}J(^{13}C,^{13}C)$	67.3902
95		$^{2}J(^{15}N,^{13}C)$	2.5492
96		$^{1}J(^{15}N,^{13}C)$	-18.1921
97		$^{1}J(^{13}C,^{1}H)$	131.5215
98		$^{2}J(^{13}C,^{1}H)$	-11.7400
99		$^{3}J(^{15}N,^{1}H)$	-1.3298
100		$^{2}J(^{1}H,^{1}H)$	-18.1320
101	H_3C−F fluoromethane (**14**)	$^{1}J(^{19}F,^{13}C)$	-162.1941
102		$^{1}J(^{13}C,^{1}H)$	142.9297
103		$^{2}J(^{19}F,^{1}H)$	49.4731
104		$^{2}J(^{1}H,^{1}H)$	-11.4740
105	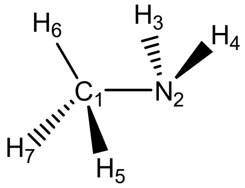 methanamine (**15**)	$^{1}J(^{15}N,^{13}C)$	-6.6450
106		$^{2}J(^{13}C,^{1}H)$	-4.1760
107		$^{1}J(^{15}N,^{1}H)$	-63.7811
108		$^{2}J(^{1}H_{3},^{1}H_{4})$	-12.0803
109		$^{1}J(^{13}C_{1},^{1}H_{5})$	129.4989
110		$^{2}J(^{15}N_{2},^{1}H_{5})$	-1.4012
111		$^{3}J(^{1}H_{5},^{1}H_{4})$	2.5642
112		$^{3}J(^{1}H_{5},^{1}H_{3})$	14.9982
113		$^{2}J(^{1}H_{7},^{1}H_{5})$	-16.3686
114		$^{1}J(^{13}C_{1},^{1}H_{6})$	125.2760
115		$^{2}J(^{15}N_{2},^{1}H_{6})$	0.9509
116		$^{3}J(^{1}H_{6},^{1}H_{4})$	1.9751
117		$^{2}J(^{1}H_{6},^{1}H_{5})$	-12.1121
118	CH_4 methane (**16**)	$^{1}J(^{13}C,^{1}H)$	122.3774
119		$^{2}J(^{1}H,^{1}H)$	-14.9015
120	CHF_3 fluoroform (**17**)	$^{1}J(^{13}C,^{1}H)$	221.2890
121		$^{1}J(^{19}F,^{13}C)$	-258.4724
122		$^{2}J(^{19}F,^{1}H)$	74.0427
123		$^{2}J(^{19}F,^{19}F)$	134.4004
124	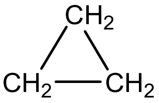 cyclopropane (**18**)	$^{1}J(^{13}C,^{13}C)$	15.3830
125		$^{1}J(^{13}C,^{1}H)$	156.4537
126		$^{2}J(^{13}C,^{1}H)$	-3.2749
127		$^{2}J(^{1}H,^{1}H)$	-6.9094
128		$^{3}J_{cis}(^{1}H,^{1}H)$	8.8085
129		$^{3}J_{trans}(^{1}H,^{1}H)$	4.7571
130	F−C≡C−F 1,2-difluoroethyne (**19**)	$^{1}J(^{13}C,^{13}C)$	411.0271
131		$^{1}J(^{19}F,^{13}C)$	-276.0366

132		$^{2}J(^{19}F,^{13}C)$	43.6022
133		$^{3}J(^{19}F,^{19}F)$	5.1885
134	F−C≡N<br>fluoroformonitrile (**20**)	$^{1}J(^{19}F,^{13}C)$	-401.9597
135		$^{1}J(^{15}N,^{13}C)$	-4.7952
136		$^{2}J(^{19}F,^{15}N)$	48.9748
137	$H_2C{=}CF_2$<br>1,1-difluoroethene (**21**)	$^{1}J(^{13}C,^{13}C)$	122.4669
138		$^{2}J(^{19}F,^{13}C)$	27.9021
139		$^{1}J(^{19}F,^{13}C)$	-287.8951
140		$^{2}J(^{19}F,^{19}F)$	20.8243
141		$^{1}J(^{13}C,^{1}H)$	164.1635
142		$^{2}J(^{13}C,^{1}H)$	-2.8232
143		$^{3}J_{cis}(^{19}F,^{1}H)$	-2.4981
144		$^{3}J_{trans}(^{19}F,^{1}H)$	25.2092
145		$^{2}J(^{1}H,^{1}H)$	-7.6521
146	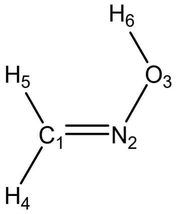<br><br>formaldehyde oxime (*Z*) (**22**)	$^{1}J(^{15}N,^{13}C)$	-3.3540
147		$^{1}J(^{13}C_1,^{1}H_4)$	183.4341
148		$^{2}J(^{15}N_2,^{1}H_4)$	-11.6454
149		$^{1}J(^{13}C_1,^{1}H_5)$	152.7111
150		$^{2}J(^{15}N_2,^{1}H_5)$	2.9030
151		$^{2}J(^{1}H,^{1}H)$	4.9947
152		$^{3}J(^{13}C,^{1}H)$	4.0644
153		$^{2}J(^{15}N_2,^{1}H_6)$	1.3716
154		$^{4}J(^{1}H_6,^{1}H_4)$	-1.6888
155		$^{4}J(^{1}H_6,^{1}H_5)$	1.4799
156	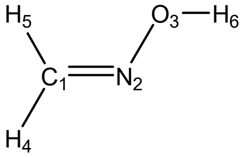<br><br>formaldehyde oxime (*E*) (**23**)	$^{1}J(^{15}N,^{13}C)$	-6.6590
157		$^{1}J(^{13}C_1,^{1}H_4)$	176.2704
158		$^{2}J(^{15}N_2,^{1}H_4)$	-12.3577
159		$^{1}J(^{13}C_1,^{1}H_5)$	162.4202
160		$^{2}J(^{15}N_2,^{1}H_5)$	2.9291
161		$^{2}J(^{1}H,^{1}H)$	6.6579
162		$^{3}J(^{13}C,^{1}H)$	10.6886
163		$^{2}J(^{15}N_2,^{1}H_6)$	-1.7983
164		$^{4}J(^{1}H_6,^{1}H_4)$	0.8192
165		$^{4}J(^{1}H_6,^{1}H_5)$	-0.9937
166	H−C≡C−F<br>fluoroethyne (**24**)	$^{1}J(^{13}C,^{13}C)$	280.7990
167		$^{2}J(^{19}F,^{13}C)$	27.4852
168		$^{1}J(^{19}F,^{13}C)$	-287.5361
169		$^{1}J(^{13}C,^{1}H)$	278.5423
170		$^{2}J(^{13}C,^{1}H)$	63.2970
171		$^{3}J(^{19}F,^{1}H)$	8.7055
172	H−C≡N<br>hydrogen cyanide (**25**)	$^{1}J(^{15}N,^{13}C)$	-19.0904
173		$^{1}J(^{13}C,^{1}H)$	261.9683
174		$^{2}J(^{15}N,^{1}H)$	-8.1427
175		$^{1}J(^{13}C,^{13}C)$	150.8125
176		$^{2}J(^{19}F_3,^{13}C_1)$	65.1835

177		$^{1}J(^{19}F_3,^{13}C_2)$	-275.2476
178		$^{1}J(^{19}F_4,^{13}C_1)$	-236.5153
179		$^{2}J(^{19}F_4,^{13}C_2)$	39.7632
180		$^{3}J_{trans}(^{19}F_3,^{19}F_4)$	-126.0988
181		$^{2}J(^{19}F_5,^{13}C_1)$	22.1833
182		$^{1}J(^{19}F_5,^{13}C_2)$	-286.7962
183	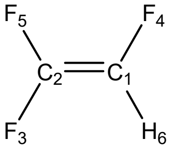	$^{2}J(^{19}F,^{19}F)$	76.1974
184		$^{3}J_{cis}(^{19}F_4,^{19}F_5)$	51.0408
185	1,1,2-trifluoroethene (**26**)	$^{1}J(^{13}C,^{1}H)$	201.9002
186		$^{2}J(^{13}C,^{1}H)$	13.3379
187		$^{3}J_{cis}(^{19}F_3,^{1}H_6)$	-6.6811
188		$^{2}J(^{19}F,^{1}H)$	72.5652
189		$^{3}J_{trans}(^{19}F_5,^{1}H_6)$	6.9398
190		$^{1}J(^{13}C,^{13}C)$	107.5151
191		$^{1}J(^{19}F,^{13}C)$	-255.1072
192		$^{2}J(^{19}F,^{13}C)$	12.3760
193	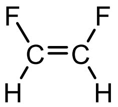	$^{3}J(^{19}F,^{19}F)$	-1.8944
194		$^{1}J(^{13}C,^{1}H)$	194.2992
195	(*Z*)-1,2-difluoroethene (**27**)	$^{2}J(^{13}C,^{1}H)$	21.6115
196		$^{2}J(^{19}F,^{1}H)$	72.2827
197		$^{3}J(^{19}F,^{1}H)$	11.9171
198		$^{3}J(^{1}H,^{1}H)$	3.5900
199		$^{1}J(^{13}C,^{13}C)$	120.4648
200		$^{1}J(^{19}F,^{13}C)$	-244.0228
201		$^{2}J(^{19}F,^{13}C)$	50.2030
202	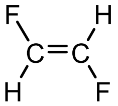	$^{3}J(^{19}F,^{19}F)$	-139.4489
203		$^{2}J(^{13}C,^{1}H)$	3.6765
204	(*E*)-1,2-difluoroethene (**28**)	$^{1}J(^{13}C,^{1}H)$	194.0004
205		$^{3}J(^{19}F,^{1}H)$	-0.8513
206		$^{2}J(^{19}F,^{1}H)$	75.8313
207		$^{3}J(^{1}H,^{1}H)$	9.8598
208	HF<br>hydrogen fluoride (**29**)	$^{1}J(^{19}F,^{1}H)$	502.5214
209		$^{1}J(^{15}N,^{15}N)$	-19.1951
210	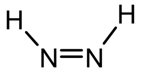	$^{1}J(^{15}N,^{1}H)$	-32.5220
211	diazene (*Z*) (**31**)	$^{2}J(^{15}N,^{1}H)$	0.7087
212		$^{3}J(^{1}H,^{1}H)$	37.4548
213		$^{1}J(^{15}N,^{15}N)$	-19.7168
214	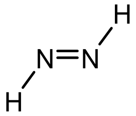	$^{2}J(^{15}N,^{1}H)$	-0.5694
215		$^{1}J(^{15}N,^{1}H)$	-43.3139
216	diazene (*E*) (**30**)	$^{3}J(^{1}H,^{1}H)$	37.8560
217		$^{1}J(^{15}N,^{15}N)$	1.7700
218		$^{1}J(^{15}N,^{1}H)$	-57.3232
219		$^{2}J(^{15}N,^{1}H)$	-1.6171
220		$^{2}J(^{1}H,^{1}H)$	-16.7562

221	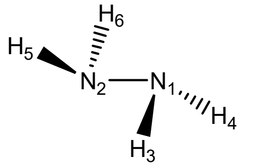	$^{3}J(^{1}H_{5},^{1}H_{3})$	1.3585
222	hydrazine (**32**)	$^{3}J(^{1}H_{4},^{1}H_{5})$	13.0708
223	N_2 molecular nitrogen (**33**)	$^{1}J(^{15}N,^{15}N)$	-2.9506
224	NH_3	$^{1}J(^{15}N,^{1}H)$	-59.7571
225	ammonia (**34**)	$^{2}J(^{1}H,^{1}H)$	-12.801
226		$^{1}J(^{13}C_{1},^{13}C_{2})$	58.7478
227		$^{1}J(^{13}C_{3},^{13}C_{1})$	74.1752
228		$^{2}J(^{13}C_{3},^{13}C_{2})$	1.4028
229		$^{2}J(^{15}N_{4},^{13}C_{1})$	2.824589
230		$^{1}J(^{15}N_{4},^{13}C_{2})$	-2.74631
231		$^{2}J(^{15}N_{4},^{13}C_{3})$	0.94672
232		$^{2}J(^{15}N_{5},^{13}C_{1})$	-5.66335
233		$^{2}J(^{15}N_{5},^{13}C_{2})$	-0.57639
234		$^{1}J(^{15}N_{5},^{13}C_{3})$	-16.5952
235		$^{1}J(^{15}N,^{15}N)$	-11.1993
236		$^{1}J(^{13}C_{1},^{1}H_{6})$	170.8716
237		$^{2}J(^{13}C_{2},^{1}H_{6})$	4.7273
238		$^{2}J(^{13}C3,^{1}H_{6})$	7.0158
239		$^{3}J(^{15}N_{4},^{1}H_{6})$	-1.02387
240		$^{3}J(^{15}N_{5},^{1}H_{6})$	-5.63487
241	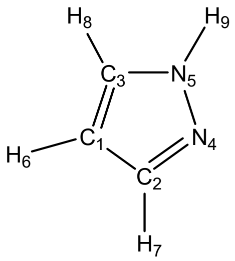	$^{2}J(^{13}C_{1},^{1}H_{7})$	10.1479
242		$^{1}J(^{13}C_{2},^{1}H_{7})$	180.2654
243		$^{3}J(^{13}C_{3},^{1}H_{7})$	4.6259
244		$^{2}J(^{15}N_{4},^{1}H_{7})$	-11.6495
245		$^{3}J(^{15}N_{5},^{1}H_{7})$	-8.70676
246		$^{3}J(^{1}H_{7},^{1}H_{6})$	1.6976
247	1*H*-pyrazole (**35**)	$^{2}J(^{13}C_{1},^{1}H_{8})$	6.3091
248		$^{3}J(^{13}C_{2},^{1}H_{8})$	7.8013
249		$^{1}J(^{13}C_{3},^{1}H_{8})$	178.7253
250		$^{3}J(^{15}N_{4},^{1}H_{8})$	0.413112
251		$^{2}J(^{15}N_{5},^{1}H_{8})$	-4.15342
252		$^{3}J(^{1}H_{8},^{1}H_{6})$	2.8786
253		$^{4}J(^{1}H_{8},^{1}H_{7})$	-0.2628
254		$^{3}J(^{13}C_{1},^{1}H_{9})$	5.2512
255		$^{3}J(^{13}C_{2},^{1}H_{9})$	9.9525
256		$^{2}J(^{13}C_{3},^{1}H_{9})$	8.8688
257		$^{2}J(^{15}N_{4},^{1}H_{9})$	-8.38385
258		$^{1}J(^{15}N,^{1}H)$	-106.319
259		$^{4}J(^{1}H_{9},^{1}H_{6})$	1.5425
260		$^{4}J(^{1}H_{9},^{1}H_{7})$	1.3758
261		$^{3}J(^{1}H_{9},^{1}H_{8})$	1.5517

262		${}^{1}J({}^{13}C,{}^{13}C)$	75.4604
263		${}^{2}J({}^{13}C_{1},{}^{13}C_{3})$	-4.6351
264		${}^{2}J({}^{13}C_{3},{}^{13}C_{2})$	9.0434
265		${}^{1}J({}^{15}N_{4},{}^{13}C_{1})$	0.684685
266		${}^{2}J({}^{15}N_{4},{}^{13}C_{2})$	2.46871
267		${}^{1}J({}^{15}N_{4},{}^{13}C_{3})$	-3.10388
268		${}^{2}J({}^{15}N_{5},{}^{13}C_{1})$	-6.14056
269		${}^{1}J({}^{15}N_{5},{}^{13}C_{2})$	-18.3273
270		${}^{1}J({}^{15}N_{5},{}^{13}C_{3})$	-14.231
271		${}^{2}J({}^{15}N,{}^{15}N)$	-1.4388
272		${}^{1}J({}^{13}C_{1},{}^{1}H_{6})$	183.7042
273		${}^{2}J({}^{13}C_{2},{}^{1}H_{6})$	14.9017
274		${}^{3}J({}^{13}C_{3},{}^{1}H_{6})$	11.3448
275		${}^{2}J({}^{15}N_{4},{}^{1}H_{6})$	-9.50409
276	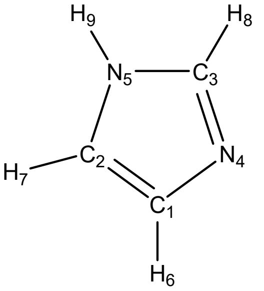	${}^{3}J({}^{15}N_{5},{}^{1}H_{6})$	-3.41304
277		${}^{2}J({}^{13}C_{1},{}^{1}H_{7})$	7.5203
278		${}^{1}J({}^{13}C_{2},{}^{1}H_{7})$	182.3225
279		${}^{3}J({}^{13}C_{3},{}^{1}H_{7})$	6.3879
280		${}^{3}J({}^{15}N_{4},{}^{1}H_{7})$	-1.0574
281		${}^{2}J({}^{15}N_{5},{}^{1}H_{7})$	-4.29524
282		${}^{3}J({}^{1}H_{7},{}^{1}H_{6})$	1.9973
283	1*H*-imidazole (**36**)	${}^{3}J({}^{13}C_{1},{}^{1}H_{8})$	11.2462
284		${}^{3}J(C_{2},{}^{1}H_{8})$	3.0013
285		${}^{1}J(C_{3},{}^{1}H_{8})$	199.7768
286		${}^{2}J({}^{15}N_{4},{}^{1}H_{8})$	-10.9071
287		${}^{2}J({}^{15}N_{5},{}^{1}H_{8})$	-8.62863
288		${}^{4}J({}^{1}H_{8},{}^{1}H_{6})$	-0.1548
289		${}^{4}J({}^{1}H8,{}^{1}H_{7})$	1.0108
290		${}^{3}J({}^{13}C_{1},{}^{1}H_{9})$	7.5844
291		${}^{2}J({}^{13}C_{2},{}^{1}H_{9})$	4.229
292		${}^{2}J({}^{13}C_{3},{}^{1}H_{9})$	4.4272
293		${}^{3}J({}^{15}N_{4},{}^{1}H_{9})$	0.192037
294		${}^{1}J({}^{15}N,{}^{1}H)$	-96.1952
295		${}^{4}J({}^{1}H_{9},{}^{1}H_{6})$	1.3083
296		${}^{3}J({}^{1}H_{9},{}^{1}H_{7})$	1.9264
297		${}^{3}J({}^{1}H_{9},{}^{1}H_{8})$	0.8706
298		${}^{1}J({}^{13}C_{1},{}^{13}C_{2})$	61.2643
299		${}^{2}J({}^{13}C_{2},{}^{13}C_{3})$	-4.4793
300		${}^{2}J({}^{13}C_{1},{}^{13}C_{4})$	-4.1201
301	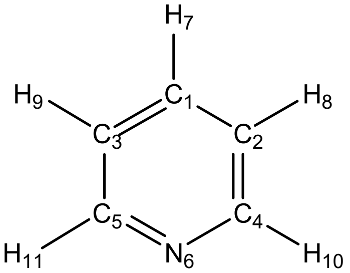	${}^{1}J({}^{13}C_{2},{}^{13}C_{4})$	61.6792
302		${}^{3}J({}^{13}C_{3},{}^{13}C_{4})$	15.0733
303		${}^{2}J({}^{13}C_{5},{}^{13}C_{4})$	-7.5115
304		${}^{3}J({}^{15}N_{6},{}^{13}C_{1})$	-4.50271
305	pyridine (**37**)	${}^{2}J({}^{15}N_{6},{}^{13}C_{2})$	2.924605
306		${}^{1}J({}^{15}N,{}^{13}C)$	-2.39016

307		$^{1}J(^{13}C_{1},^{1}H_{7})$	155.42
308		$^{2}J(^{13}C_{2},^{1}H_{7})$	-1.0247
309		$^{3}J(^{13}C_{4},^{1}H_{7})$	6.823
310		$^{4}J(^{15}N,^{1}H)$	0.525051
311		$^{2}J(^{13}C_{1},^{1}H_{8})$	-0.9053
312		$^{1}J(^{13}C_{2},^{1}H_{8})$	157.9782
313		$^{3}J(^{13}C_{3},^{1}H_{8})$	6.6564
314		$^{2}J(^{13}C_{4},^{1}H_{8})$	1.2941
315		$^{4}J(^{13}C_{5},^{1}H_{8})$	-1.5726
316		$^{3}J(^{15}N,^{1}H)$	-1.59676
317		$^{3}J(^{1}H_{7},^{1}H_{8})$	7.8642
318		$^{4}J(^{1}H_{8},^{1}H_{9})$	0.3623
319		$^{3}J(^{13}C_{1},^{1}H_{10})$	6.6731
320		$^{2}J(^{13}C_{2},^{1}H_{10})$	6.9065
321		$^{4}J(^{13}C_{3},^{1}H_{10})$	-2.3386
322		$^{1}J(^{13}C_{4},^{1}H_{10})$	172.2317
323		$^{3}J(^{13}C_{5},^{1}H_{10})$	11.2576
324		$^{2}J(^{15}N,^{1}H)$	-9.66457
325		$^{4}J(^{1}H_{7},^{1}H_{10})$	0.9322
326		$^{3}J(^{1}H_{8},^{1}H_{10})$	5.3622
327		$^{5}J(^{1}H,^{1}H)$	1.2054
328		$^{4}J(^{1}H_{10},^{1}H_{11})$	-1.0663
329	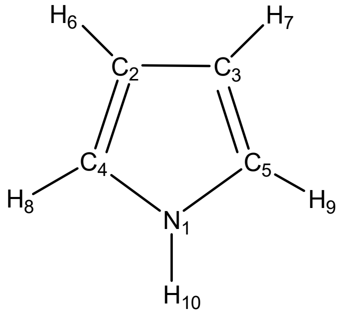 1*H*-pyrrole (**38**)	$^{2}J(^{15}N,^{13}C)$	-4.24291
330		$^{1}J(^{13}C_{2},^{13}C_{3})$	59.1731
331		$^{1}J(^{15}N,^{13}C)$	-17.3331
332		$^{1}J(^{13}C_{5},^{13}C_{3})$	75.5227
333		$^{2}J(^{13}C_{2},^{13}C_{5})$	0.6583
334		$^{2}J(^{13}C_{4},^{13}C_{5})$	7.4546
335		$^{3}J(^{15}N,^{1}H)$	-5.37929
336		$^{1}J(^{13}C_{3},^{1}H_{7})$	165.7257
337		$^{2}J(^{13}C_{2},^{1}H_{7})$	2.8691
338		$^{2}J(^{13}C_{5},^{1}H_{7})$	6.3536
339		$^{3}J(^{13}C_{4},^{1}H_{7})$	7.2529
340		$^{3}J(^{1}H_{6},^{1}H_{7})$	3.3977
341		$^{2}J(^{15}N,^{1}H)$	-3.67606
342		$^{2}J(^{13}C_{3},^{1}H_{9})$	5.6131
343		$^{3}J(^{13}C_{2},^{1}H_{9})$	7.5731
344		$^{1}J(^{13}C_{5},^{1}H_{9})$	177.9727
345		$^{3}J(^{13}C_{4},^{1}H_{9})$	5.6065
346		$^{3}J(^{1}H_{7},^{1}H_{9})$	3.4551
347		$^{4}J(^{1}H_{6},^{1}H_{9})$	0.5531
348		$^{4}J(^{1}H_{8},^{1}H_{9})$	1.7484
349		$^{1}J(^{15}N,^{1}H)$	-96.0962
350		$^{3}J(^{13}C_{3},^{1}H_{10})$	6.8882
351		$^{2}J(^{13}C_{5},^{1}H_{10})$	3.3483

352		$^{4}J(^{1}H_{7},^{1}H_{10})$	2.0043
353		$^{3}J(^{1}H_{9},^{1}H_{10})$	2.6212
354		$^{1}J(^{13}C_{2},^{13}C_{1})$	78.3995
355		$^{2}J(^{13}C,^{13}C)$	-0.5116
356		$^{1}J(^{13}C_{2},^{13}C_{3})$	46.9792
357		$^{1}J(^{13}C_{1},^{1}H_{4})$	155.1895
358		$^{2}J(^{13}C_{2},^{1}H_{4})$	-2.8811
359		$^{3}J(^{13}C_{3},^{1}H_{4})$	11.9012
360		$^{1}J(^{13}C_{1},^{1}H_{5})$	151.5836
361		$^{2}J(^{13}C_{2},^{1}H_{5})$	-4.1478
362		$^{3}J(^{13}C_{3},^{1}H_{5})$	7.7989
363		$^{2}J(^{1}H_{4},^{1}H_{5})$	-1.5605
364		$^{2}J(^{13}C_{1},^{1}H_{6})$	-1.593
365		$^{1}J(^{13}C_{2},^{1}H_{6})$	148.5895
366		$^{2}J(^{13}C_{3},^{1}H_{6})$	3.5745
367	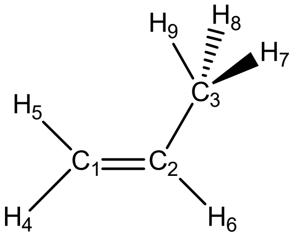	$^{3}J(^{1}H_{4},^{1}H_{6})$	10.692
368		$^{3}J(^{1}H_{5},^{1}H_{6})$	15.8382
369		$^{3}J(^{13}C_{1},^{1}H_{7})$	6.269
370		$^{2}J(^{13}C_{2},^{1}H_{7})$	-8.9092
371	prop-1-ene (**39**)	$^{1}J(^{13}C_{3},^{1}H_{7})$	122.6292
372		$^{4}J(^{1}H_{4},^{1}H_{7})$	-3.0576
373		$^{4}J(^{1}H_{5},^{1}H_{7})$	-3.286
374		$^{3}J(^{1}H_{6},^{1}H_{7})$	3.9096
375		$^{2}J(^{1}H_{7},^{1}H_{8})$	-19.7099
376		$^{3}J(^{13}C_{1},^{1}H_{9})$	6.3349
377		$^{2}J(^{13}C_{2},^{1}H_{9})$	-6.4543
378		$^{1}J(^{13}C_{3},^{1}H_{9})$	123.91
379		$^{4}J(^{1}H_{4},^{1}H_{9})$	-0.6324
380		$^{4}J(^{1}H_{5},^{1}H_{9})$	-0.4733
381		$^{3}J(^{1}H_{6},^{1}H_{9})$	11.5055
382		$^{2}J(^{1}H_{7},^{1}H_{9})$	-14.9038
383		$^{1}J(^{13}C_{1},^{13}C_{2})$	56.8736
384		$^{1}J(^{13}C_{1},^{13}C_{3})$	78.7389
385		$^{2}J(^{13}C_{2},^{13}C_{3})$	-0.4429
386		$^{2}J(^{13}C_{3},^{13}C_{4})$	4.0304
387		$^{1}J(^{13}C_{1},^{1}H_{6})$	169.817
388		$^{2}J(^{13}C_{2},^{1}H_{6})$	2.7239
389	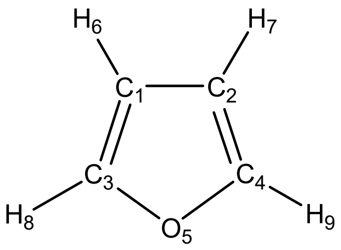	$^{2}J(^{13}C_{3},^{1}H_{6})$	8.1622
390		$^{3}J(^{13}C_{4},^{1}H_{6})$	6.6328
391		$^{3}J(^{1}H_{6},^{1}H_{7})$	3.0303
392		$^{2}J(^{13}C_{1},^{1}H_{8})$	11.4216
393	furan (**40**)	$^{3}J(^{13}C_{2},^{1}H_{8})$	6.284
394		$^{1}J(^{13}C_{3},^{1}H_{8})$	195.169
395		$^{3}J(^{13}C_{4},^{1}H_{8})$	6.3352
396		$^{3}J(^{1}H_{6},^{1}H_{8})$	2.6697

397		$^{4}J(^{1}H_{7},^{1}H_{8})$	-0.0522
398		$^{4}J(^{1}H_{8},^{1}H_{9})$	1.2198
399		$^{1}J(^{19}F,^{13}C)$	-249.034
400		$^{2}J(^{19}F,^{13}C)$	21.8441
401		$^{1}J(^{13}C_{2},^{13}C_{3})$	77.6188
402		$^{2}J(^{13}C_{3},^{13}C_{4})$	2.5978
403		$^{3}J(^{19}F,^{13}C)$	4.2779
404		$^{2}J(^{13}C_{2},^{13}C_{6})$	-1.0467
405		$^{1}J(^{13}C_{3},^{13}C_{6})$	64.2889
406		$^{3}J(^{13}C_{4},^{13}C_{6})$	7.8883
407		$^{2}J(^{13}C_{5},^{13}C_{6})$	-2.8502
408		$^{4}J(^{19}F,^{13}C)$	6.1705
409		$^{3}J(^{13}C_{7},^{13}C_{2})$	11.5687
410		$^{2}J(^{13}C_{3},^{13}C_{7})$	-4.285
411		$^{1}J(^{13}C_{6},^{13}C_{7})$	63.7309
412		$^{3}J(^{19}F,^{1}H)$	5.9327
413	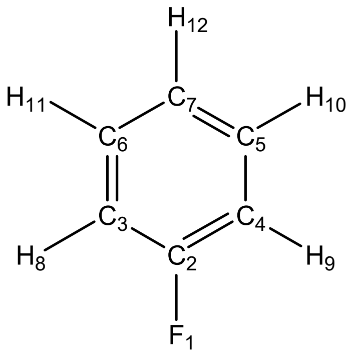	$^{2}J(^{13}C_{2},^{1}H_{8})$	-6.7069
414		$^{1}J(^{13}C_{3},^{1}H_{8})$	159.0112
415		$^{3}J(^{13}C_{4},^{1}H_{8})$	4.5415
416		$^{2}J(^{13}C_{6},^{1}H_{8})$	-1.9919
417		$^{4}J(^{13}C_{5},^{1}H_{8})$	-1.5037
418		$^{3}J(^{13}C_{7},^{1}H_{8})$	7.8284
419		$^{4}J(^{1}H_{8},^{1}H_{9})$	1.602
420		$^{4}J(^{19}F,^{1}H)$	4.6978
421		$^{3}J(^{13}C_{2},^{1}H_{11})$	10.6054
422	1-fluorobenzene (**41**)	$^{2}J(^{13}C_{3},^{1}H_{11})$	-0.9236
423		$^{4}J(^{13}C_{4},^{1}H_{11})$	-2.1476
424		$^{1}J(^{13}C_{6},^{1}H_{11})$	155.7721
425		$^{3}J(^{13}C_{5},^{1}H_{11})$	8.9681
426		$^{2}J(^{13}C_{7},^{1}H_{11})$	-1.1245
427		$^{3}J(^{1}H_{8},^{1}H_{11})$	8.6002
428		$^{5}J(^{1}H_{9},^{1}H_{11})$	0.8009
429		$^{4}J(^{1}H_{10},^{1}H_{11})$	0.7315
430		$^{5}J(^{19}F,^{1}H)$	-1.9607
431		$^{4}J(^{13}C_{2},^{1}H_{12})$	-2.5214
432		$^{3}J(^{13}C_{3},^{1}H_{12})$	8.2255
433		$^{2}J(^{13}C_{6},^{1}H_{12})$	-0.2644
434		$^{1}J(^{13}C_{7},^{1}H_{12})$	157.5209
435		$^{4}J(^{1}H_{8},^{1}H_{12})$	0.1783
436		$^{3}\mathrm{J}(^{1}H_{11},^{1}H_{12})$	7.8717

Table S13. Symmetry independent values of SSCC (in Hz) in molecules of set 1 calculated at the SOPPA(CCSD) level with the ccJ-pVTZ basis set.

#	Molecule	Type of SSCC [1]	SSCC value
1	$H_2C{=}C{=}CH_2$ propa-1,2-diene (**1**)	$^{1}J(^{13}C,^{13}C)$	104.6831
2		$^{2}J(^{13}C,^{13}C)$	8.3843
3		$^{2}J(^{13}C,^{1}H)$	-5.8322
4		$^{1}J(^{13}C,^{1}H)$	165.3324
5		$^{3}J(^{13}C,^{1}H)$	7.8539
6		$^{2}J(^{1}H,^{1}H)$	-13.6958
7		$^{4}J(^{1}H,^{1}H)$	-8.6390
8	$F_2C{=}CF_2$ Perfluoroethene (**2**)	$^{1}J(^{13}C,^{13}C)$	198.7685
9		$^{1}J(^{13}C,^{19}F)$	-260.9235
10		$^{2}J(^{13}C,^{19}F)$	51.1152
11		$^{2}J(^{19}F,^{19}F)$	122.6079
12		$^{3}J_{cis}(^{19}F,^{19}F)$	81.7882
13		$^{3}J_{trans}(^{19}F,^{19}F)$	-115.7916
14	$HC{\equiv}CH$ ethyne (**3**)	$^{1}J(^{13}C,^{13}C)$	191.1630
15		$^{1}J(^{13}C,^{1}H)$	249.4234
16		$^{2}J(^{13}C,^{1}H)$	51.3456
17		$^{3}J(^{1}H,^{1}H)$	10.7458
18	$H_2C{=}CH_2$ ethene (**4**)	$^{1}J(^{13}C,^{13}C)$	71.9032
19		$^{1}J(^{13}C,^{1}H)$	152.9832
20		$^{2}J(^{13}C,^{1}H)$	-3.2014
21		$^{3}J_{cis}(^{1}H,^{1}H)$	12.0964
22		$^{2}J(^{1}H,^{1}H)$	0.2838
23		$^{3}J_{trans}(^{1}H,^{1}H)$	18.1081
24	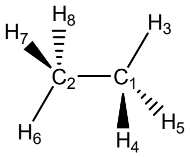 ethane (**5**)	$^{1}J(^{13}C,^{13}C)$	35.3574
25		$^{1}J(^{13}C,^{1}H)$	121.2488
26		$^{2}J(^{13}C,^{1}H)$	-4.9724
27		$^{3}J_{trans}(^{1}H_3,^{1}H_6)$	15.5407
28		$^{2}J(^{1}H,^{1}H)$	-13.9471
29		$^{3}J_{gauche}(^{1}H_4,^{1}H_6)$	3.7308
30	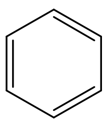 benzene (**6**)	$^{3}J(^{13}C,^{13}C)$	11.1011
31		$^{1}J(^{13}C,^{13}C)$	59.8536
32		$^{2}J(^{13}C,^{13}C)$	-3.4724
33		$^{1}J(^{13}C,^{1}H)$	154.0581
34		$^{4}J(^{13}C,^{1}H)$	-1.7344
35		$^{2}J(^{13}C,^{1}H)$	-0.1277
36		$^{3}J(^{13}C,^{1}H)$	7.7354
37		$^{3}J(^{1}H,^{1}H)$	7.9688
38		$^{4}J(^{1}H,^{1}H)$	0.7660
39		$^{5}J(^{1}H,^{1}H)$	1.0971
40	CF_4 perfluoromethane (**7**)	$^{1}J(^{19}F,^{13}C)$	-251.0024
41		$^{2}J(^{19}F,^{19}F)$	36.8564

42		$^{1}J(^{13}C,^{13}C)$	88.8025
43		$^{2}J(^{19}F,^{13}C)$	12.3158
44		$^{1}J(^{19}F,^{13}C)$	-255.4654
45		$^{1}J(^{13}C_1,^{1}H_4)$	157.6502
46		$^{2}J(^{13}C_2,^{1}H_4)$	6.6628
47		$^{3}J_{trans}(^{19}F_3,^{1}H_4)$	39.6761
48	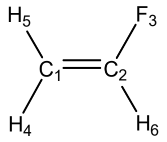	$^{1}J(^{13}C_1,^{1}H_5)$	156.0426
49		$^{2}J(^{13}C_2,^{1}H_5)$	-9.9980
50		$^{3}J_{cis}(^{19}F_3,^{1}H_5)$	12.8748
51	fluoroethene (**8**)	$^{2}J(^{1}H,^{1}H)$	-4.5166
52		$^{2}J(^{13}C_1,^{1}H_6)$	12.9732
53		$^{1}J(^{13}C_2,^{1}H_6)$	191.3182
54		$^{2}J(^{19}F,^{1}H)$	81.5955
55		$^{3}J_{cis}(^{1}H_6,^{1}H_4)$	5.9022
56		$^{3}J_{trans}(^{1}H_6,^{1}H_5)$	12.5981
57		$^{1}J(^{19}F,^{13}C)$	-221.4825
58		$^{2}J(^{19}F,^{19}F)$	327.6088
59	CH_2F_2	$^{1}J(^{13}C,^{1}H)$	173.6207
60	difluoromethane (**9**)	$^{2}J(^{19}F,^{1}H)$	50.6957
61		$^{2}J(^{1}H,^{1}H)$	1.1564
62		$^{1}J(^{15}N,^{13}C)$	-4.1719
63		$^{1}J(^{13}C_1,^{1}H_3)$	170.2402
64		$^{2}J(^{15}N_2,^{1}H_3)$	-9.5178
65	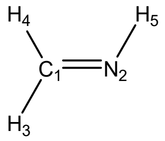	$^{1}J(^{13}C_1,^{1}H_4)$	154.2019
66		$^{2}J(^{15}N_2,^{1}H_4)$	3.8510
67		$^{2}J(^{1}H,^{1}H)$	16.6594
68	methanimine (**10**)	$^{2}J(^{13}C,^{1}H)$	-13.4376
69		$^{1}J(^{15}N,^{1}H)$	-49.7601
70		$^{3}J_{trans}(^{1}H_5,^{1}H_3)$	24.1368
71		$^{3}J_{cis}(^{1}H_5,^{1}H_4)$	18.0294
72		$^{1}J(^{13}C_1,^{13}C_2)$	191.8911
73		$^{2}J(^{13}C,^{13}C)$	13.0480
74		$^{1}J(^{13}C_2,^{13}C_3)$	71.3913
75		$^{1}J(^{13}C_1,^{1}H_4)$	248.1966
76	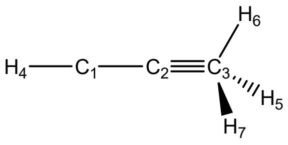	$^{2}J(^{13}C_2,^{1}H_4)$	51.2036
77		$^{3}J(^{13}C_3,^{1}H_4)$	4.1629
78		$^{3}J(^{13}C_1,^{1}H_6)$	3.7583
79	prop-1-yne (**11**)	$^{2}J(^{13}C_2,^{1}H_6)$	-11.7224
80		$^{1}J(^{13}C_3,^{1}H_6)$	127.4374
81		$^{4}J(^{1}H,^{1}H)$	-3.6381
82		$^{2}J(^{1}H,^{1}H)$	-17.1954
83		$^{1}J(^{13}C,^{13}C)$	42.3921
84		$^{1}J(^{13}C_1,^{1}H_4)$	164.2808
85		$^{2}J(^{13}C_2,^{1}H_4)$	26.4884
86		$^{2}J(^{13}C_1,^{1}H_5)$	-8.3519

87	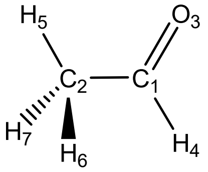	$^{1}J(^{13}C_{2},^{1}H_{5})$	130.4553
88	acetaldehyde (**12**)	$^{3}J_{trans}(^{1}H_{5},^{1}H_{4})$	8.0148
89		$^{2}J(^{13}C_{1},^{1}H_{6})$	-6.4419
90		$^{1}J(^{13}C_{2},^{1}H_{6})$	119.4058
91		$^{3}J_{gauche}(^{1}H_{6},^{1}H_{4})$	0.3163
92		$^{2}J(^{1}H_{6},^{1}H_{5})$	-13.3025
93		$^{2}J(^{1}H_{6},^{1}H_{7})$	-19.8649
94	N≡C−CH_3	$^{1}J(^{13}C,^{13}C)$	63.0658
95	acetonitrile (**13**)	$^{2}J(^{15}N,^{13}C)$	2.7431
96		$^{1}J(^{15}N,^{13}C)$	-18.1514
97		$^{1}J(^{13}C,^{1}H)$	130.6705
98		$^{2}J(^{13}C,^{1}H)$	-10.9361
99		$^{3}J(^{15}N,^{1}H)$	-1.5193
100		$^{2}J(^{1}H,^{1}H)$	-17.2328
101	H_3C−F	$^{1}J(^{19}F,^{13}C)$	-158.0545
102	fluoromethane (**14**)	$^{1}J(^{13}C,^{1}H)$	142.6285
103		$^{2}J(^{19}F,^{1}H)$	49.2210
104		$^{2}J(^{1}H,^{1}H)$	-10.6409
105	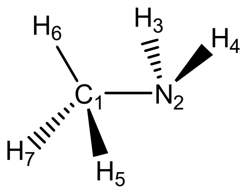	$^{1}J(^{15}N,^{13}C)$	-5.9097
106	methanamine (**15**)	$^{2}J(^{13}C,^{1}H)$	-3.6370
107		$^{1}J(^{15}N,^{1}H)$	-64.6097
108		$^{2}J(^{1}H_{3},^{1}H_{4})$	-10.5643
109		$^{1}J(^{13}C_{1},^{1}H_{5})$	128.8105
110		$^{2}J(^{15}N_{2},^{1}H_{5})$	-1.5690
111		$^{3}J(^{1}H_{5},^{1}H_{4})$	2.6874
112		$^{3}J(^{1}H_{5},^{1}H_{3})$	15.4222
113		$^{2}J(^{1}H_{7},^{1}H_{5})$	-15.5433
114		$^{1}J(^{13}C_{1},^{1}H_{6})$	125.5791
115		$^{2}J(^{15}N_{2},^{1}H_{6})$	0.8839
116		$^{3}J(^{1}H_{6},^{1}H_{4})$	2.1206
117		$^{2}J(^{1}H_{6},^{1}H_{5})$	-11.3649
118	CH_4	$^{1}J(^{13}C,^{1}H)$	121.4321
119	methane (**16**)	$^{2}J(^{1}H,^{1}H)$	-13.7215
120	CHF_3	$^{1}J(^{13}C,^{1}H)$	223.1599
121	fluoroform (**17**)	$^{1}J(^{19}F,^{13}C)$	-256.1089
122		$^{2}J(^{19}F,^{1}H)$	75.2130
123		$^{2}J(^{19}F,^{19}F)$	132.3052
124	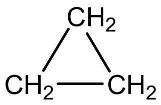	$^{1}J(^{13}C,^{13}C)$	13.8195
125	cyclopropane (**18**)	$^{1}J(^{13}C,^{1}H)$	155.7277
126		$^{2}J(^{13}C,^{1}H)$	-2.9489
127		$^{2}J(^{1}H,^{1}H)$	-5.9766
128		$^{3}J_{cis}(^{1}H,^{1}H)$	8.9769
129		$^{3}J_{trans}(^{1}H,^{1}H)$	5.0223
130	F−C≡C−F	$^{1}J(^{13}C,^{13}C)$	410.7169
131	1,2-difluoroethyne (**19**)	$^{1}J(^{19}F,^{13}C)$	-273.5037

132		$^{2}J(^{19}F,^{13}C)$	41.3398
133		$^{3}J(^{19}F,^{19}F)$	-1.9865
134	F−C≡N<br>fluoroformonitrile (**20**)	$^{1}J(^{19}F,^{13}C)$	-399.8769
135		$^{1}J(^{15}N,^{13}C)$	-4.3323
136		$^{2}J(^{19}F,^{15}N)$	50.4599
137	$H_2C{=}CF_2$<br>1,1-difluoroethene (**21**)	$^{1}J(^{13}C,^{13}C)$	118.3892
138		$^{2}J(^{19}F,^{13}C)$	28.2284
139		$^{1}J(^{19}F,^{13}C)$	-281.0367
140		$^{2}J(^{19}F,^{19}F)$	25.8521
141		$^{1}J(^{13}C,^{1}H)$	162.7652
142		$^{2}J(^{13}C,^{1}H)$	-2.0553
143		$^{3}J_{cis}(^{19}F,^{1}H)$	-1.7406
144		$^{3}J_{trans}(^{19}F,^{1}H)$	26.9477
145		$^{2}J(^{1}H,^{1}H)$	-6.2097
146	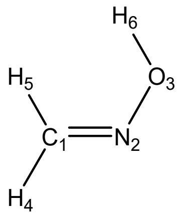<br>formaldehyde oxime (*Z*) (**22**)	$^{1}J(^{15}N,^{13}C)$	-3.0849
147		$^{1}J(^{13}C_1,^{1}H_4)$	182.4252
148		$^{2}J(^{15}N_2,^{1}H_4)$	-12.1383
149		$^{1}J(^{13}C_1,^{1}H_5)$	152.1885
150		$^{2}J(^{15}N_2,^{1}H_5)$	2.9109
151		$^{2}J(^{1}H,^{1}H)$	6.5008
152		$^{3}J(^{13}C,^{1}H)$	4.0628
153		$^{2}J(^{15}N_2,^{1}H_6)$	1.3833
154		$^{4}J(^{1}H_6,^{1}H_4)$	-1.3360
155		$^{4}J(^{1}H_6,^{1}H_5)$	1.1600
156	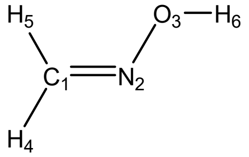<br>formaldehyde oxime (*E*) (**23**)	$^{1}J(^{15}N,^{13}C)$	-6.1407
157		$^{1}J(^{13}C_1,^{1}H_4)$	175.5759
158		$^{2}J(^{15}N_2,^{1}H_4)$	-12.9455
159		$^{1}J(^{13}C_1,^{1}H_5)$	161.4583
160		$^{2}J(^{15}N_2,^{1}H_5)$	2.8998
161		$^{2}J(^{1}H,^{1}H)$	8.1253
162		$^{3}J(^{13}C,^{1}H)$	10.8096
163		$^{2}J(^{15}N_2,^{1}H_6)$	-1.9404
164		$^{4}J(^{1}H_6,^{1}H_4)$	1.2769
165		$^{4}J(^{1}H_6,^{1}H_5)$	-0.5034
166	H−C≡C−F<br>fluoroethyne (**24**)	$^{1}J(^{13}C,^{13}C)$	276.0499
167		$^{2}J(^{19}F,^{13}C)$	24.6779
168		$^{1}J(^{19}F,^{13}C)$	-287.9205
169		$^{1}J(^{13}C,^{1}H)$	277.6149
170		$^{2}J(^{13}C,^{1}H)$	65.8139
171		$^{3}J(^{19}F,^{1}H)$	11.2650
172	H−C≡N<br>hydrogen cyanide (**25**)	$^{1}J(^{15}N,^{13}C)$	-18.7324
173		$^{1}J(^{13}C,^{1}H)$	260.3511
174		$^{2}J(^{15}N,^{1}H)$	-8.2827
175		$^{1}J(^{13}C,^{13}C)$	146.5077
176		$^{2}J(^{19}F_3,^{13}C_1)$	66.0070

177	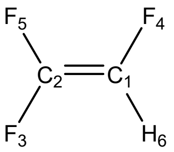 1,1,2-trifluoroethene (**26**)	$^{1}J(^{19}F_3,^{13}C_2)$	-267.0785
178		$^{1}J(^{19}F_4,^{13}C_1)$	-228.0661
179		$^{2}J(^{19}F_4,^{13}C_2)$	37.4695
180		$^{3}J_{trans}(^{19}F_3,^{19}F_4)$	-121.9796
181		$^{2}J(^{19}F_5,^{13}C_1)$	20.7253
182		$^{1}J(^{19}F_5,^{13}C_2)$	-279.5201
183		$^{2}J(^{19}F,^{19}F)$	77.7767
184		$^{3}J_{cis}(^{19}F_4,^{19}F_5)$	42.6290
185		$^{1}J(^{13}C,^{1}H)$	202.1788
186		$^{2}J(^{13}C,^{1}H)$	14.1874
187		$^{3}J_{cis}(^{19}F_3,^{1}H_6)$	-5.7149
188		$^{2}J(^{19}F,^{1}H)$	72.2396
189		$^{3}J_{trans}(^{19}F_5,^{1}H_6)$	7.8473
190	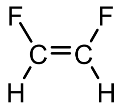 (*Z*)-1,2-difluoroethene (**27**)	$^{1}J(^{13}C,^{13}C)$	102.6085
191		$^{1}J(^{19}F,^{13}C)$	-248.2288
192		$^{2}J(^{19}F,^{13}C)$	10.0448
193		$^{3}J(^{19}F,^{19}F)$	-9.4817
194		$^{1}J(^{13}C,^{1}H)$	195.1243
195		$^{2}J(^{13}C,^{1}H)$	22.6742
196		$^{2}J(^{19}F,^{1}H)$	72.2092
197		$^{3}J(^{19}F,^{1}H)$	13.5317
198		$^{3}J(^{1}H,^{1}H)$	3.4236
199	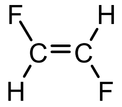 (*E*)-1,2-difluoroethene (**28**)	$^{1}J(^{13}C,^{13}C)$	116.2749
200		$^{1}J(^{19}F,^{13}C)$	-236.0069
201		$^{2}J(^{19}F,^{13}C)$	50.2783
202		$^{3}J(^{19}F,^{19}F)$	-135.1999
203		$^{2}J(^{13}C,^{1}H)$	4.4778
204		$^{1}J(^{13}C,^{1}H)$	193.9082
205		$^{3}J(^{19}F,^{1}H)$	0.1160
206		$^{2}J(^{19}F,^{1}H)$	75.7426
207		$^{3}J(^{1}H,^{1}H)$	9.8359
208	HF hydrogen fluoride (**29**)	$^{1}J(^{19}F,^{1}H)$	532.6543
209	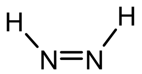 diazene (*Z*) (**31**)	$^{1}J(^{15}N,^{15}N)$	-20.8152
210		$^{1}J(^{15}N,^{1}H)$	-34.8405
211		$^{2}J(^{15}N,^{1}H)$	1.0749
212		$^{3}J(^{1}H,^{1}H)$	37.5112
213	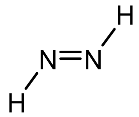 diazene (*E*) (**30**)	$^{1}J(^{15}N,^{15}N)$	-21.1741
214		$^{2}J(^{15}N,^{1}H)$	-0.5175
215		$^{1}J(^{15}N,^{1}H)$	-44.9037
216		$^{3}J(^{1}H,^{1}H)$	37.2388
217		$^{1}J(^{15}N,^{15}N)$	1.1019
218		$^{1}J(^{15}N,^{1}H)$	-58.5291
219		$^{2}J(^{15}N,^{1}H)$	-1.7116
220		$^{2}J(^{1}H,^{1}H)$	-15.0740

221	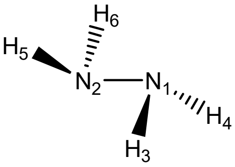	$^{3}J(^{1}H_{5},^{1}H_{3})$	1.5114
222	hydrazine (**32**)	$^{3}J(^{1}H_{4},^{1}H_{5})$	13.3175
223	N_2<br>molecular nitrogen (**33**)	$^{1}J(^{15}N,^{15}N)$	-3.38704
224	NH_3	$^{1}J(^{15}N,^{1}H)$	-60.9886
225	ammonia (**34**)	$^{2}J(^{1}H,^{1}H)$	-11.0651
226		$^{1}J(^{13}C_{1},^{13}C_{2})$	55.4893
227		$^{1}J(^{13}C_{3},^{13}C_{1})$	70.3339
228		$^{2}J(^{13}C_{3},^{13}C_{2})$	1.7854
229		$^{2}J(^{15}N_{4},^{13}C_{1})$	2.917311
230		$^{1}J(^{15}N_{4},^{13}C_{2})$	-2.41372
231		$^{2}J(^{15}N_{4},^{13}C_{3})$	0.966499
232		$^{2}J(^{15}N_{5},^{13}C_{1})$	-5.79633
233		$^{2}J(^{15}N_{5},^{13}C_{2})$	-0.7436
234		$^{1}J(^{15}N_{5},^{13}C_{3})$	-15.7555
235		$^{1}J(^{15}N,^{15}N)$	-11.8233
236		$^{1}J(^{13}C_{1},^{1}H_{6})$	170.8594
237		$^{2}J(^{13}C_{2},^{1}H_{6})$	5.099
238		$^{2}J(^{13}C3,^{1}H_{6})$	7.5855
239		$^{3}J(^{15}N_{4},^{1}H_{6})$	-1.08152
240	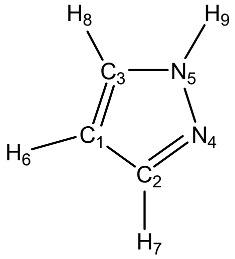	$^{3}J(^{15}N_{5},^{1}H_{6})$	-5.72647
241		$^{2}J(^{13}C_{1},^{1}H_{7})$	10.5449
242		$^{1}J(^{13}C_{2},^{1}H_{7})$	180.3428
243		$^{3}J(^{13}C_{3},^{1}H_{7})$	4.6865
244		$^{2}J(^{15}N_{4},^{1}H_{7})$	-11.8934
245		$^{3}J(^{15}N_{5},^{1}H_{7})$	-8.77185
246		$^{3}J(^{1}H_{7},^{1}H_{6})$	1.8081
247	1*H*-pyrazole (**35**)	$^{2}J(^{13}C_{1},^{1}H_{8})$	6.8308
248		$^{3}J(^{13}C_{2},^{1}H_{8})$	7.8572
249		$^{1}J(^{13}C_{3},^{1}H_{8})$	178.7672
250		$^{3}J(^{15}N_{4},^{1}H_{8})$	0.295
251		$^{2}J(^{15}N_{5},^{1}H_{8})$	-4.43425
252		$^{3}J(^{1}H_{8},^{1}H_{6})$	2.9728
253		$^{4}J(^{1}H_{8},^{1}H_{7})$	0.1228
254		$^{3}J(^{13}C_{1},^{1}H_{9})$	5.4213
255		$^{3}J(^{13}C_{2},^{1}H_{9})$	10.2178
256		$^{2}J(^{13}C_{3},^{1}H_{9})$	9.1265
257		$^{2}J(^{15}N_{4},^{1}H_{9})$	-8.55779
258		$^{1}J(^{15}N,^{1}H)$	-106.303
259		$^{4}J(^{1}H_{9},^{1}H_{6})$	1.9679
260		$^{4}J(^{1}H_{9},^{1}H_{7})$	1.8522
261		$^{3}J(^{1}H_{9},^{1}H_{8})$	1.6624

262		$^{1}J(^{13}C,^{13}C)$	72.1588
263		$^{2}J(^{13}C_{1},^{13}C_{3})$	-4.4118
264		$^{2}J(^{13}C_{3},^{13}C_{2})$	9.1319
265		$^{1}J(^{15}N_{4},^{13}C_{1})$	1.108317
266		$^{2}J(^{15}N_{4},^{13}C_{2})$	2.485683
267		$^{1}J(^{15}N_{4},^{13}C_{3})$	-2.84282
268		$^{2}J(^{15}N_{5},^{13}C_{1})$	-6.31913
269		$^{1}J(^{15}N_{5},^{13}C_{2})$	-17.1964
270		$^{1}J(^{15}N_{5},^{13}C_{3})$	-13.5193
271		$^{2}J(^{15}N,^{15}N)$	-1.51515
272		$^{1}J(^{13}C_{1},^{1}H_{6})$	184.0946
273		$^{2}J(^{13}C_{2},^{1}H_{6})$	15.4785
274		$^{3}J(^{13}C_{3},^{1}H_{6})$	11.4975
275		$^{2}J(^{15}N_{4},^{1}H_{6})$	-9.75476
276	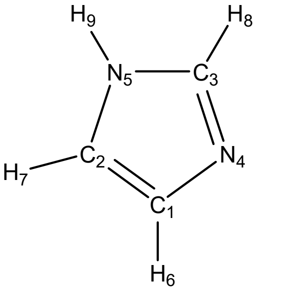	$^{3}J(^{15}N_{5},^{1}H_{6})$	-3.52092
277		$^{2}J(^{13}C_{1},^{1}H_{7})$	8.1215
278		$^{1}J(^{13}C_{2},^{1}H_{7})$	182.4805
279		$^{3}J(^{13}C_{3},^{1}H_{7})$	6.7311
280		$^{3}J(^{15}N_{4},^{1}H_{7})$	-1.10215
281		$^{2}J(^{15}N_{5},^{1}H_{7})$	-4.56302
282		$^{3}J(^{1}H_{7},^{1}H_{6})$	2.0552
283	1*H*-imidazole (**36**)	$^{3}J(^{13}C_{1},^{1}H_{8})$	11.3168
284		$^{3}J(C_{2},^{1}H_{8})$	3.2718
285		$^{1}J(C_{3},^{1}H_{8})$	200.0291
286		$^{2}J(^{15}N_{4},^{1}H_{8})$	-11.1233
287		$^{2}J(^{15}N_{5},^{1}H_{8})$	-8.83301
288		$^{4}J(^{1}H_{8},^{1}H_{6})$	0.2368
289		$^{4}J(^{1}H8,^{1}H_{7})$	1.3224
290		$^{3}J(^{13}C_{1},^{1}H_{9})$	7.8001
291		$^{2}J(^{13}C_{2},^{1}H_{9})$	4.4379
292		$^{2}J(^{13}C_{3},^{1}H_{9})$	4.6179
293		$^{3}J(^{15}N_{4},^{1}H_{9})$	0.134945
294		$^{1}J(^{15}N,^{1}H)$	-96.1209
295		$^{4}J(^{1}H_{9},^{1}H_{6})$	1.6998
296		$^{3}J(^{1}H_{9},^{1}H_{7})$	2.072
297		$^{3}J(^{1}H_{9},^{1}H_{8})$	0.9732
298	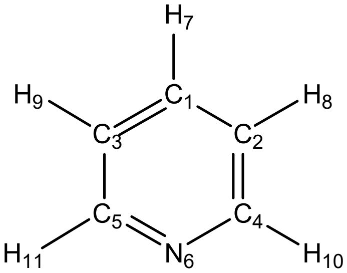	$^{1}J(^{13}C_{1},^{13}C_{2})$	57.8068
299		$^{2}J(^{13}C_{2},^{13}C_{3})$	-4.1278
300		$^{2}J(^{13}C_{1},^{13}C_{4})$	-3.7038
301		$^{1}J(^{13}C_{2},^{13}C_{4})$	58.5478
302		$^{3}J(^{13}C_{3},^{13}C_{4})$	15.238
303		$^{2}J(^{13}C_{5},^{13}C_{4})$	-7.2685
304		$^{3}J(^{15}N_{6},^{13}C_{1})$	-4.58519
305	pyridine (**37**)	$^{2}J(^{15}N_{6},^{13}C_{2})$	2.955325
306		$^{1}J(^{15}N,^{13}C)$	-1.95516

307		$^{1}J(^{13}C_1,^{1}H_7)$	155.0262
308		$^{2}J(^{13}C_2,^{1}H_7)$	-0.4159
309		$^{3}J(^{13}C_4,^{1}H_7)$	6.8164
310		$^{4}J(^{15}N,^{1}H)$	0.520001
311		$^{2}J(^{13}C_1,^{1}H_8)$	-0.3583
312		$^{1}J(^{13}C_2,^{1}H_8)$	157.6135
313		$^{3}J(^{13}C_3,^{1}H_8)$	6.6518
314		$^{2}J(^{13}C_4,^{1}H_8)$	1.854
315		$^{4}J(^{13}C_5,^{1}H_8)$	-1.3098
316		$^{3}J(^{15}N,^{1}H)$	-1.61163
317		$^{3}J(^{1}H_7,^{1}H_8)$	7.9533
318		$^{4}J(^{1}H_8,^{1}H_9)$	0.7675
319		$^{3}J(^{13}C_1,^{1}H_{10})$	6.642
320		$^{2}J(^{13}C_2,^{1}H_{10})$	7.4361
321		$^{4}J(^{13}C_3,^{1}H_{10})$	-2.1001
322		$^{1}J(^{13}C_4,^{1}H_{10})$	172.0339
323		$^{3}J(^{13}C_5,^{1}H_{10})$	11.3081
324		$^{2}J(^{15}N,^{1}H)$	-9.85702
325		$^{4}J(^{1}H_7,^{1}H_{10})$	1.2863
326		$^{3}J(^{1}H_8,^{1}H_{10})$	5.3684
327		$^{5}J(^{1}H,^{1}H)$	1.3375
328		$^{4}J(^{1}H_{10},^{1}H_{11})$	-0.6909
329		$^{2}J(^{15}N,^{13}C)$	-4.42625
330		$^{1}J(^{13}C_2,^{13}C_3)$	55.7283
331		$^{1}J(^{15}N,^{13}C)$	-16.327
332		$^{1}J(^{13}C_5,^{13}C_3)$	71.8211
333		$^{2}J(^{13}C_2,^{13}C_5)$	1.0859
334		$^{2}J(^{13}C_4,^{13}C_5)$	7.5677
335		$^{3}J(^{15}N,^{1}H)$	-5.44985
336	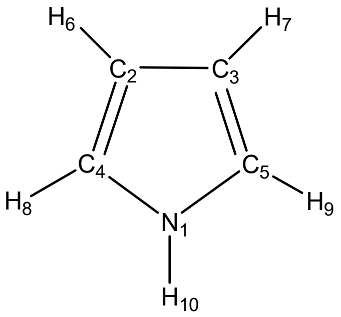	$^{1}J(^{13}C_3,^{1}H_7)$	165.7967
337		$^{2}J(^{13}C_2,^{1}H_7)$	3.317
338		$^{2}J(^{13}C_5,^{1}H_7)$	6.955
339		$^{3}J(^{13}C_4,^{1}H_7)$	7.3116
340		$^{3}J(^{1}H_6,^{1}H_7)$	3.5652
341		$^{2}J(^{15}N,^{1}H)$	-3.94497
342		$^{2}J(^{13}C_3,^{1}H_9)$	6.1907
343		$^{3}J(^{13}C_2,^{1}H_9)$	7.5969
344	1*H*-pyrrole (**38**)	$^{1}J(^{13}C_5,^{1}H_9)$	177.9989
345		$^{3}J(^{13}C_4,^{1}H_9)$	5.9177
346		$^{3}J(^{1}H_7,^{1}H_9)$	3.5599
347		$^{4}J(^{1}H_6,^{1}H_9)$	0.9363
348		$^{4}J(^{1}H_8,^{1}H_9)$	2.0832
349		$^{1}J(^{15}N,^{1}H)$	-95.9928
350		$^{3}J(^{13}C_3,^{1}H_{10})$	7.0542
351		$^{2}J(^{13}C_5,^{1}H_{10})$	3.5894

352		$^{4}J(^{1}H_{7},^{1}H_{10})$	2.4553
353		$^{3}J(^{1}H_{9},^{1}H_{10})$	2.7603
354		$^{1}J(^{13}C_{2},^{13}C_{1})$	74.3666
355		$^{2}J(^{13}C,^{13}C)$	-0.0664
356		$^{1}J(^{13}C_{2},^{13}C_{3})$	44.3357
357		$^{1}J(^{13}C_{1},^{1}H_{4})$	154.0234
358		$^{2}J(^{13}C_{2},^{1}H_{4})$	-2.0124
359		$^{3}J(^{13}C_{3},^{1}H_{4})$	11.8665
360		$^{1}J(^{13}C_{1},^{1}H_{5})$	150.3169
361		$^{2}J(^{13}C_{2},^{1}H_{5})$	-3.4889
362		$^{3}J(^{13}C_{3},^{1}H_{5})$	7.6808
363		$^{2}J(^{1}H_{4},^{1}H_{5})$	0.1
364		$^{2}J(^{13}C_{1},^{1}H_{6})$	-0.7934
365		$^{1}J(^{13}C_{2},^{1}H_{6})$	147.9189
366		$^{2}J(^{13}C_{3},^{1}H_{6})$	3.9985
367	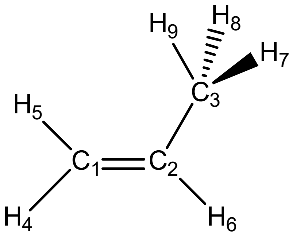	$^{3}J(^{1}H_{4},^{1}H_{6})$	10.7844
368		$^{3}J(^{1}H_{5},^{1}H_{6})$	16.1168
369		$^{3}J(^{13}C_{1},^{1}H_{7})$	6.231
370		$^{2}J(^{13}C_{2},^{1}H_{7})$	-8.1954
371	prop-1-ene (**39**)	$^{1}J(^{13}C_{3},^{1}H_{7})$	121.8979
372		$^{4}J(^{1}H_{4},^{1}H_{7})$	-2.7055
373		$^{4}J(^{1}H_{5},^{1}H_{7})$	-2.9405
374		$^{3}J(^{1}H_{6},^{1}H_{7})$	3.8564
375		$^{2}J(^{1}H_{7},^{1}H_{8})$	-18.8948
376		$^{3}J(^{13}C_{1},^{1}H_{9})$	6.3688
377		$^{2}J(^{13}C_{2},^{1}H_{9})$	-6.0717
378		$^{1}J(^{13}C_{3},^{1}H_{9})$	123.027
379		$^{4}J(^{1}H_{4},^{1}H_{9})$	-0.3749
380		$^{4}J(^{1}H_{5},^{1}H_{9})$	-0.5571
381		$^{3}J(^{1}H_{6},^{1}H_{9})$	11.8918
382		$^{2}J(^{1}H_{7},^{1}H_{9})$	-14.002
383		$^{1}J(^{13}C_{1},^{13}C_{2})$	53.4304
384		$^{1}J(^{13}C_{1},^{13}C_{3})$	75.1138
385		$^{2}J(^{13}C_{2},^{13}C_{3})$	-0.003
386		$^{2}J(^{13}C_{3},^{13}C_{4})$	4.0984
387		$^{1}J(^{13}C_{1},^{1}H_{6})$	169.6279
388		$^{2}J(^{13}C_{2},^{1}H_{6})$	3.1354
389	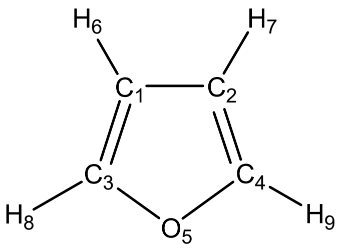	$^{2}J(^{13}C_{3},^{1}H_{6})$	8.7714
390		$^{3}J(^{13}C_{4},^{1}H_{6})$	6.7291
391		$^{3}J(^{1}H_{6},^{1}H_{7})$	3.1827
392		$^{2}J(^{13}C_{1},^{1}H_{8})$	12.0548
393	furan (**40**)	$^{3}J(^{13}C_{2},^{1}H_{8})$	6.2789
394		$^{1}J(^{13}C_{3},^{1}H_{8})$	195.2082
395		$^{3}J(^{13}C_{4},^{1}H_{8})$	6.6498
396		$^{3}J(^{1}H_{6},^{1}H_{8})$	2.7252

397		$^{4}J(^{1}H_{7},^{1}H_{8})$	0.327
398		$^{4}J(^{1}H_{8},^{1}H_{9})$	1.6384
399		$^{1}J(^{19}F,^{13}C)$	-243.466
400		$^{2}J(^{19}F,^{13}C)$	22.1536
401		$^{1}J(^{13}C_{2},^{13}C_{3})$	74.3841
402		$^{2}J(^{13}C_{3},^{13}C_{4})$	2.9395
403		$^{3}J(^{19}F,^{13}C)$	5.1177
404		$^{2}J(^{13}C_{2},^{13}C_{6})$	-0.5413
405		$^{1}J(^{13}C_{3},^{13}C_{6})$	60.9424
406		$^{3}J(^{13}C_{4},^{13}C_{6})$	7.8443
407		$^{2}J(^{13}C_{5},^{13}C_{6})$	-2.4383
408		$^{4}J(^{19}F,^{13}C)$	5.6984
409		$^{3}J(^{13}C_{7},^{13}C_{2})$	11.6122
410		$^{2}J(^{13}C_{3},^{13}C_{7})$	-3.9339
411		$^{1}J(^{13}C_{6},^{13}C_{7})$	60.2687
412		$^{3}J(^{19}F,^{1}H)$	6.0487
413	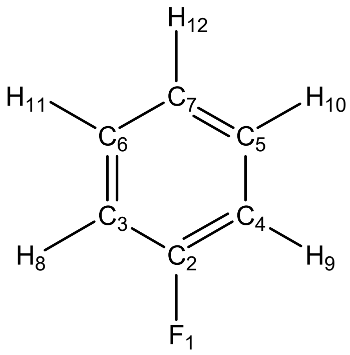 1-fluorobenzene (**41**)	$^{2}J(^{13}C_{2},^{1}H_{8})$	-6.3229
414		$^{1}J(^{13}C_{3},^{1}H_{8})$	158.2196
415		$^{3}J(^{13}C_{4},^{1}H_{8})$	4.4724
416		$^{2}J(^{13}C_{6},^{1}H_{8})$	-1.4721
417		$^{4}J(^{13}C_{5},^{1}H_{8})$	-1.2597
418		$^{3}J(^{13}C_{7},^{1}H_{8})$	7.8135
419		$^{4}J(^{1}H_{8},^{1}H_{9})$	2.0244
420		$^{4}J(^{19}F,^{1}H)$	4.8583
421		$^{3}J(^{13}C_{2},^{1}H_{11})$	10.666
422		$^{2}J(^{13}C_{3},^{1}H_{11})$	-0.2574
423		$^{4}J(^{13}C_{4},^{1}H_{11})$	-1.9275
424		$^{1}J(^{13}C_{6},^{1}H_{11})$	155.3539
425		$^{3}J(^{13}C_{5},^{1}H_{11})$	8.9821
426		$^{2}J(^{13}C_{7},^{1}H_{11})$	-0.5337
427		$^{3}J(^{1}H_{8},^{1}H_{11})$	8.6859
428		$^{5}J(^{1}H_{9},^{1}H_{11})$	0.9187
429		$^{4}J(^{1}H_{10},^{1}H_{11})$	1.1235
430		$^{5}J(^{19}F,^{1}H)$	-1.4049
431		$^{4}J(^{13}C_{2},^{1}H_{12})$	-2.2825
432		$^{3}J(^{13}C_{3},^{1}H_{12})$	8.2286
433		$^{2}J(^{13}C_{6},^{1}H_{12})$	0.3493
434		$^{1}J(^{13}C_{7},^{1}H_{12})$	157.0329
435		$^{4}J(^{1}H_{8},^{1}H_{12})$	0.5348
436		$^{3}\mathrm{J}(^{1}H_{11},^{1}H_{12})$	7.9387

Table S14. Symmetry independent values of SSCC (in Hz) in molecules of set 1 calculated at the SOPPA(CCSD) level with the aug-cc-pVTZ-J basis set.

#	Molecule	Type of SSCC [1]	SSCC value
1	$H_2C{=}C{=}CH_2$ propa-1,2-diene (**1**)	$^1J(^{13}C,^{13}C)$	104.8846
2		$^2J(^{13}C,^{13}C)$	8.6327
3		$^2J(^{13}C,^{1}H)$	-5.5284
4		$^1J(^{13}C,^{1}H)$	166.9188
5		$^3J(^{13}C,^{1}H)$	7.7690
6		$^2J(^{1}H,^{1}H)$	-14.1479
7		$^4J(^{1}H,^{1}H)$	-8.7251
8	$F_2C{=}CF_2$ Perfluoroethene (**2**)	$^1J(^{13}C,^{13}C)$	199.8182
9		$^1J(^{13}C,^{19}F)$	-265.7612
10		$^2J(^{13}C,^{19}F)$	49.6448
11		$^2J(^{19}F,^{19}F)$	109.5507
12		$^3J_{cis}(^{19}F,^{19}F)$	71.1629
13		$^3J_{trans}(^{19}F,^{19}F)$	-109.1139
14	$HC{\equiv}CH$ ethyne (**3**)	$^1J(^{13}C,^{13}C)$	190.3570
15		$^1J(^{13}C,^{1}H)$	252.0702
16		$^2J(^{13}C,^{1}H)$	51.8948
17		$^3J(^{1}H,^{1}H)$	10.8586
18	$H_2C{=}CH_2$ ethene (**4**)	$^1J(^{13}C,^{13}C)$	72.0138
19		$^1J(^{13}C,^{1}H)$	154.3658
20		$^2J(^{13}C,^{1}H)$	-2.9704
21		$^3J_{cis}(^{1}H,^{1}H)$	12.2656
22		$^2J(^{1}H,^{1}H)$	0.0895
23		$^3J_{trans}(^{1}H,^{1}H)$	18.3753
24	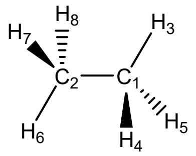 ethane (**5**)	$^1J(^{13}C,^{13}C)$	35.2113
25		$^1J(^{13}C,^{1}H)$	121.8838
26		$^2J(^{13}C,^{1}H)$	-5.0254
27		$^3J_{trans}(^{1}H_3,^{1}H_6)$	15.7632
28		$^2J(^{1}H,^{1}H)$	-14.3650
29		$^3J_{gauche}(^{1}H_4,^{1}H_6)$	3.7931
30	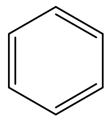 benzene (**6**)	$^3J(^{13}C,^{13}C)$	10.8052
31		$^1J(^{13}C,^{13}C)$	59.8885
32		$^2J(^{13}C,^{13}C)$	-3.2477
33		$^1J(^{13}C,^{1}H)$	155.5402
34		$^4J(^{13}C,^{1}H)$	-1.7048
35		$^2J(^{13}C,^{1}H)$	0.0289
36		$^3J(^{13}C,^{1}H)$	7.8108
37		$^3J(^{1}H,^{1}H)$	8.0882
38		$^4J(^{1}H,^{1}H)$	0.7934
39		$^5J(^{1}H,^{1}H)$	1.0896
40	CF_4 perfluoromethane (**7**)	$^1J(^{19}F,^{13}C)$	-254.1922
41		$^2J(^{19}F,^{19}F)$	36.6075

42		$^{1}J(^{13}C,^{13}C)$	88.9263
43		$^{2}J(^{19}F,^{13}C)$	11.8083
44		$^{1}J(^{19}F,^{13}C)$	-259.1238
45		$^{1}J(^{13}C_1,^{1}H_4)$	159.6062
46		$^{2}J(^{13}C_2,^{1}H_4)$	7.1687
47	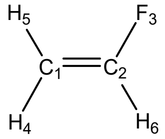	$^{3}J_{trans}(^{19}F_3,^{1}H_4)$	41.9139
48		$^{1}J(^{13}C_1,^{1}H_5)$	157.2977
49		$^{2}J(^{13}C_2,^{1}H_5)$	-9.9038
50		$^{3}J_{cis}(^{19}F_3,^{1}H_5)$	14.2397
51	fluoroethene (**8**)	$^{2}J(^{1}H,^{1}H)$	-4.8837
52		$^{2}J(^{13}C_1,^{1}H_6)$	13.4082
53		$^{1}J(^{13}C_2,^{1}H_6)$	193.8449
54		$^{2}J(^{19}F,^{1}H)$	83.1991
55		$^{3}J_{cis}(^{1}H_6,^{1}H_4)$	5.9543
56		$^{3}J_{trans}(^{1}H_6,^{1}H_5)$	12.7059
57		$^{1}J(^{19}F,^{13}C)$	-227.0071
58	CH_2F_2	$^{2}J(^{19}F,^{19}F)$	307.8282
59	difluoromethane (**9**)	$^{1}J(^{13}C,^{1}H)$	175.1049
60		$^{2}J(^{19}F,^{1}H)$	50.1873
61		$^{2}J(^{1}H,^{1}H)$	0.7456
62		$^{1}J(^{15}N,^{13}C)$	-4.5597
63		$^{1}J(^{13}C_1,^{1}H_3)$	171.6694
64		$^{2}J(^{15}N_2,^{1}H_3)$	-9.4347
65	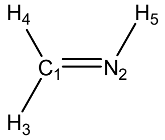	$^{1}J(^{13}C_1,^{1}H_4)$	156.3684
66		$^{2}J(^{15}N_2,^{1}H_4)$	4.2853
67		$^{2}J(^{1}H,^{1}H)$	16.6227
68	methanimine (**10**)	$^{2}J(^{13}C,^{1}H)$	-13.4422
69		$^{1}J(^{15}N,^{1}H)$	-51.3424
70		$^{3}J_{trans}(^{1}H_5,^{1}H_3)$	24.5132
71		$^{3}J_{cis}(^{1}H_5,^{1}H_4)$	18.3212
72		$^{1}J(^{13}C_1,^{13}C_2)$	191.0650
73		$^{2}J(^{13}C,^{13}C)$	13.1976
74		$^{1}J(^{13}C_2,^{13}C_3)$	71.2965
75		$^{1}J(^{13}C_1,^{1}H_4)$	250.8041
76	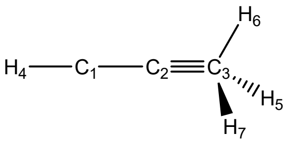	$^{2}J(^{13}C_2,^{1}H_4)$	51.7490
77		$^{3}J(^{13}C_3,^{1}H_4)$	4.1951
78		$^{3}J(^{13}C_1,^{1}H_6)$	3.6970
79	prop-1-yne (**11**)	$^{2}J(^{13}C_2,^{1}H_6)$	-11.8003
80		$^{1}J(^{13}C_3,^{1}H_6)$	128.2197
81		$^{4}J(^{1}H,^{1}H)$	-3.6905
82		$^{2}J(^{1}H,^{1}H)$	-17.6837
83		$^{1}J(^{13}C,^{13}C)$	42.2273
84		$^{1}J(^{13}C_1,^{1}H_4)$	165.9628
85		$^{2}J(^{13}C_2,^{1}H_4)$	26.7950
86		$^{2}J(^{13}C_1,^{1}H_5)$	-8.4591

87	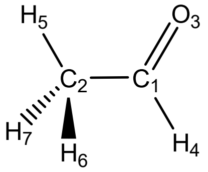 acetaldehyde (**12**)	$^{1}J(^{13}C_2,^{1}H_5)$	131.4008
88		$^{3}J_{trans}(^{1}H_5,^{1}H_4)$	8.0905
89		$^{2}J(^{13}C_1,^{1}H_6)$	-6.4629
90		$^{1}J(^{13}C_2,^{1}H_6)$	120.1644
91		$^{3}J_{gauche}(^{1}H_6,^{1}H_4)$	0.3242
92		$^{2}J(^{1}H_6,^{1}H_5)$	-13.7032
93		$^{2}J(^{1}H_6,^{1}H_7)$	-20.4521
94	N≡C−CH_3 acetonitrile (**13**)	$^{1}J(^{13}C,^{13}C)$	62.8846
95		$^{2}J(^{15}N,^{13}C)$	2.8045
96		$^{1}J(^{15}N,^{13}C)$	-17.3778
97		$^{1}J(^{13}C,^{1}H)$	131.5193
98		$^{2}J(^{13}C,^{1}H)$	-11.0254
99		$^{3}J(^{15}N,^{1}H)$	-1.5482
100		$^{2}J(^{1}H,^{1}H)$	-17.7066
101	H_3C−F fluoromethane (**14**)	$^{1}J(^{19}F,^{13}C)$	-165.2342
102		$^{1}J(^{13}C,^{1}H)$	143.7854
103		$^{2}J(^{19}F,^{1}H)$	47.9860
104		$^{2}J(^{1}H,^{1}H)$	-11.2874
105	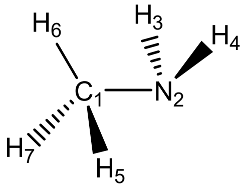 methanamine (**15**)	$^{1}J(^{15}N,^{13}C)$	-5.8119
106		$^{2}J(^{13}C,^{1}H)$	-3.6746
107		$^{1}J(^{15}N,^{1}H)$	-65.7351
108		$^{2}J(^{1}H_3,^{1}H_4)$	-11.1361
109		$^{1}J(^{13}C_1,^{1}H_5)$	129.6351
110		$^{2}J(^{15}N_2,^{1}H_5)$	-1.4357
111		$^{3}J(^{1}H_5,^{1}H_4)$	2.7424
112		$^{3}J(^{1}H_5,^{1}H_3)$	15.6038
113		$^{2}J(^{1}H_7,^{1}H_5)$	-16.0914
114		$^{1}J(^{13}C_1,^{1}H_6)$	126.9516
115		$^{2}J(^{15}N_2,^{1}H_6)$	1.1054
116		$^{3}J(^{1}H_6,^{1}H_4)$	2.1812
117		$^{2}J(^{1}H_6,^{1}H_5)$	-11.8860
118	CH_4 methane (**16**)	$^{1}J(^{13}C,^{1}H)$	122.1501
119		$^{2}J(^{1}H,^{1}H)$	-14.1498
120	CHF_3 fluoroform (**17**)	$^{1}J(^{13}C,^{1}H)$	225.2834
121		$^{1}J(^{19}F,^{13}C)$	-260.6073
122		$^{2}J(^{19}F,^{1}H)$	75.2116
123		$^{2}J(^{19}F,^{19}F)$	125.5124
124	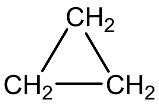 cyclopropane (**18**)	$^{1}J(^{13}C,^{13}C)$	13.6591
125		$^{1}J(^{13}C,^{1}H)$	156.9629
126		$^{2}J(^{13}C,^{1}H)$	-2.9634
127		$^{2}J(^{1}H,^{1}H)$	-6.1939
128		$^{3}J_{cis}(^{1}H,^{1}H)$	9.1106
129		$^{3}J_{trans}(^{1}H,^{1}H)$	5.0762
130	F−C≡C−F 1,2-difluoroethyne (**19**)	$^{1}J(^{13}C,^{13}C)$	412.7773
131		$^{1}J(^{19}F,^{13}C)$	-277.5413

132		$^{2}J(^{19}F,^{13}C)$	37.5908
133		$^{3}J(^{19}F,^{19}F)$	-10.5625
134		$^{1}J(^{19}F,^{13}C)$	-404.4762
135	F−C≡N<br>fluoroformonitrile (**20**)	$^{1}J(^{15}N,^{13}C)$	-3.0504
136		$^{2}J(^{19}F,^{15}N)$	52.9227
137		$^{1}J(^{13}C,^{13}C)$	118.8602
138		$^{2}J(^{19}F,^{13}C)$	28.2594
139		$^{1}J(^{19}F,^{13}C)$	-284.5852
140		$^{2}J(^{19}F,^{19}F)$	24.6770
141	$H_2C{=}CF_2$<br>1,1-difluoroethene (**21**)	$^{1}J(^{13}C,^{1}H)$	164.4983
142		$^{2}J(^{13}C,^{1}H)$	-1.8225
143		$^{3}J_{cis}(^{19}F,^{1}H)$	-1.2003
144		$^{3}J_{trans}(^{19}F,^{1}H)$	28.1320
145		$^{2}J(^{1}H,^{1}H)$	-6.5744
146		$^{1}J(^{15}N,^{13}C)$	-3.1960
147		$^{1}J(^{13}C_1,^{1}H_4)$	184.4193
148		$^{2}J(^{15}N_2,^{1}H_4)$	-12.3238
149		$^{1}J(^{13}C_1,^{1}H_5)$	153.7321
150	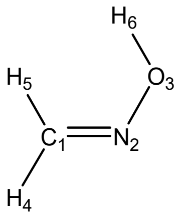	$^{2}J(^{15}N_2,^{1}H_5)$	3.1625
151		$^{2}J(^{1}H,^{1}H)$	6.1949
152		$^{3}J(^{13}C,^{1}H)$	4.3182
153	formaldehyde oxime (*Z*) (**22**)	$^{2}J(^{15}N_2,^{1}H_6)$	1.4837
154		$^{4}J(^{1}H_6,^{1}H_4)$	-1.3627
155		$^{4}J(^{1}H_6,^{1}H_5)$	1.1561
156		$^{1}J(^{15}N,^{13}C)$	-6.2232
157		$^{1}J(^{13}C_1,^{1}H_4)$	177.5910
158		$^{2}J(^{15}N_2,^{1}H_4)$	-13.1302
159		$^{1}J(^{13}C_1,^{1}H_5)$	163.1605
160	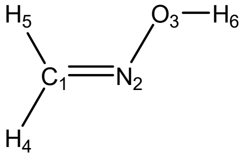	$^{2}J(^{15}N_2,^{1}H_5)$	3.1743
161		$^{2}J(^{1}H,^{1}H)$	7.8732
162	formaldehyde oxime (*E*) (**23**)	$^{3}J(^{13}C,^{1}H)$	11.0051
163		$^{2}J(^{15}N_2,^{1}H_6)$	-1.8824
164		$^{4}J(^{1}H_6,^{1}H_4)$	1.3052
165		$^{4}J(^{1}H_6,^{1}H_5)$	-0.5078
166		$^{1}J(^{13}C,^{13}C)$	276.0899
167		$^{2}J(^{19}F,^{13}C)$	21.3793
168	H−C≡C−F<br>fluoroethyne (**24**)	$^{1}J(^{19}F,^{13}C)$	-291.1342
169		$^{1}J(^{13}C,^{1}H)$	280.8715
170		$^{2}J(^{13}C,^{1}H)$	66.7345
171		$^{3}J(^{19}F,^{1}H)$	10.9325
172		$^{1}J(^{15}N,^{13}C)$	-18.1167
173	H−C≡N<br>hydrogen cyanide (**25**)	$^{1}J(^{13}C,^{1}H)$	263.2082
174		$^{2}J(^{15}N,^{1}H)$	-8.2471
175		$^{1}J(^{13}C,^{13}C)$	147.0491
176		$^{2}J(^{19}F_3,^{13}C_1)$	65.2346

177		$^{1}J(^{19}F_3,^{13}C_2)$	-271.3420
178		$^{1}J(^{19}F_4,^{13}C_1)$	-233.8991
179		$^{2}J(^{19}F_4,^{13}C_2)$	35.5425
180		$^{3}J_{trans}(^{19}F_3,^{19}F_4)$	-115.4958
181		$^{2}J(^{19}F_5,^{13}C_1)$	20.1725
182	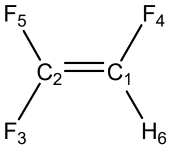	$^{1}J(^{19}F_5,^{13}C_2)$	-283.7175
183		$^{2}J(^{19}F,^{19}F)$	70.5478
184		$^{3}J_{cis}(^{19}F_4,^{19}F_5)$	34.4611
185	1,1,2-trifluoroethene (**26**)	$^{1}J(^{13}C,^{1}H)$	205.1008
186		$^{2}J(^{13}C,^{1}H)$	14.6368
187		$^{3}J_{cis}(^{19}F_3,^{1}H_6)$	-5.3955
188		$^{2}J(^{19}F,^{1}H)$	72.7854
189		$^{3}J_{trans}(^{19}F_5,^{1}H_6)$	8.4253
190		$^{1}J(^{13}C,^{13}C)$	102.4200
191		$^{1}J(^{19}F,^{13}C)$	-253.2892
192		$^{2}J(^{19}F,^{13}C)$	8.8904
193	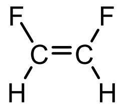	$^{3}J(^{19}F,^{19}F)$	-14.3738
194		$^{1}J(^{13}C,^{1}H)$	198.2660
195	(*Z*)-1,2-difluoroethene (**27**)	$^{2}J(^{13}C,^{1}H)$	23.3808
196		$^{2}J(^{19}F,^{1}H)$	72.9645
197		$^{3}J(^{19}F,^{1}H)$	14.6398
198		$^{3}J(^{1}H,^{1}H)$	3.4914
199		$^{1}J(^{13}C,^{13}C)$	116.7271
200		$^{1}J(^{19}F,^{13}C)$	-240.5358
201		$^{2}J(^{19}F,^{13}C)$	49.1852
202	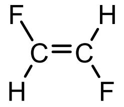	$^{3}J(^{19}F,^{19}F)$	-128.2132
203		$^{2}J(^{13}C,^{1}H)$	4.7677
204	(*E*)-1,2-difluoroethene (**28**)	$^{1}J(^{13}C,^{1}H)$	196.3149
205		$^{3}J(^{19}F,^{1}H)$	0.6823
206		$^{2}J(^{19}F,^{1}H)$	76.6598
207		$^{3}J(^{1}H,^{1}H)$	9.8992
208	HF<br>hydrogen fluoride (**29**)	$^{1}J(^{19}F,^{1}H)$	539.4070
209		$^{1}J(^{15}N,^{15}N)$	-19.8461
210	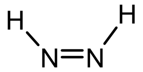	$^{1}J(^{15}N,^{1}H)$	-36.7251
211	diazene (*Z*) (**31**)	$^{2}J(^{15}N,^{1}H)$	1.8885
212		$^{3}J(^{1}H,^{1}H)$	38.2179
213		$^{1}J(^{15}N,^{15}N)$	-20.2660
214	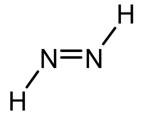	$^{2}J(^{15}N,^{1}H)$	-0.0188
215		$^{1}J(^{15}N,^{1}H)$	-46.7935
216	diazene (*E*) (**30**)	$^{3}J(^{1}H,^{1}H)$	37.6630
217		$^{1}J(^{15}N,^{15}N)$	0.6865
218		$^{1}J(^{15}N,^{1}H)$	-59.7595
219		$^{2}J(^{15}N,^{1}H)$	-1.5352
220		$^{2}J(^{1}H,^{1}H)$	-15.7127

221	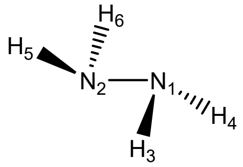	$^{3}J(^{1}H_{5},^{1}H_{3})$	1.5455
222	hydrazine (**32**)	$^{3}J(^{1}H_{4},^{1}H_{5})$	13.3959
223	N_2<br>molecular nitrogen (**33**)	$^{1}J(^{15}N,^{15}N)$	-2.62022
224	NH_3	$^{1}J(^{15}N,^{1}H)$	-62.3281
225	ammonia (**34**)	$^{2}J(^{1}H,^{1}H)$	-11.5946
226		$^{1}J(^{13}C_{1},^{13}C_{2})$	55.5918
227		$^{1}J(^{13}C_{3},^{13}C_{1})$	70.415
228		$^{2}J(^{13}C_{3},^{13}C_{2})$	2.0157
229		$^{2}J(^{15}N_{4},^{13}C_{1})$	3.038649
230		$^{1}J(^{15}N_{4},^{13}C_{2})$	-2.48133
231		$^{2}J(^{15}N_{4},^{13}C_{3})$	1.097797
232		$^{2}J(^{15}N_{5},^{13}C_{1})$	-5.77683
233		$^{2}J(^{15}N_{5},^{13}C_{2})$	-0.66406
234		$^{1}J(^{15}N_{5},^{13}C_{3})$	-15.729
235		$^{1}J(^{15}N,^{15}N)$	-11.7715
236		$^{1}J(^{13}C_{1},^{1}H_{6})$	172.749
237		$^{2}J(^{13}C_{2},^{1}H_{6})$	5.3171
238		$^{2}J(^{13}C3,^{1}H_{6})$	7.8622
239		$^{3}J(^{15}N_{4},^{1}H_{6})$	-1.141
240	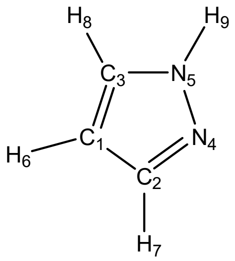	$^{3}J(^{15}N_{5},^{1}H_{6})$	-5.8255
241		$^{2}J(^{13}C_{1},^{1}H_{7})$	10.7767
242		$^{1}J(^{13}C_{2},^{1}H_{7})$	182.2203
243		$^{3}J(^{13}C_{3},^{1}H_{7})$	4.7663
244		$^{2}J(^{15}N_{4},^{1}H_{7})$	-12.0859
245		$^{3}J(^{15}N_{5},^{1}H_{7})$	-8.88968
246		$^{3}J(^{1}H_{7},^{1}H_{6})$	1.8277
247	1*H*-pyrazole (**35**)	$^{2}J(^{13}C_{1},^{1}H_{8})$	7.0658
248		$^{3}J(^{13}C_{2},^{1}H_{8})$	7.9344
249		$^{1}J(^{13}C_{3},^{1}H_{8})$	180.8319
250		$^{3}J(^{15}N_{4},^{1}H_{8})$	0.255863
251		$^{2}J(^{15}N_{5},^{1}H_{8})$	-4.58757
252		$^{3}J(^{1}H_{8},^{1}H_{6})$	3.0128
253		$^{4}J(^{1}H_{8},^{1}H_{7})$	0.1271
254		$^{3}J(^{13}C_{1},^{1}H_{9})$	5.5379
255		$^{3}J(^{13}C_{2},^{1}H_{9})$	10.3554
256		$^{2}J(^{13}C_{3},^{1}H_{9})$	9.2928
257		$^{2}J(^{15}N_{4},^{1}H_{9})$	-8.70312
258		$^{1}J(^{15}N,^{1}H)$	-107.415
259		$^{4}J(^{1}H_{9},^{1}H_{6})$	1.9894
260		$^{4}J(^{1}H_{9},^{1}H_{7})$	1.8907
261		$^{3}J(^{1}H_{9},^{1}H_{8})$	1.689

262		$^{1}J(^{13}C,^{13}C)$	72.3823
263		$^{2}J(^{13}C_{1},^{13}C_{3})$	-4.3828
264		$^{2}J(^{13}C_{3},^{13}C_{2})$	9.0166
265		$^{1}J(^{15}N_{4},^{13}C_{1})$	1.016717
266		$^{2}J(^{15}N_{4},^{13}C_{2})$	2.566482
267		$^{1}J(^{15}N_{4},^{13}C_{3})$	-2.93849
268		$^{2}J(^{15}N_{5},^{13}C_{1})$	-6.30511
269		$^{1}J(^{15}N_{5},^{13}C_{2})$	-17.1187
270		$^{1}J(^{15}N_{5},^{13}C_{3})$	-13.5188
271		$^{2}J(^{15}N,^{15}N)$	-1.61727
272		$^{1}J(^{13}C_{1},^{1}H_{6})$	186.094
273		$^{2}J(^{13}C_{2},^{1}H_{6})$	15.8719
274		$^{3}J(^{13}C_{3},^{1}H_{6})$	11.6142
275		$^{2}J(^{15}N_{4},^{1}H_{6})$	-9.87049
276	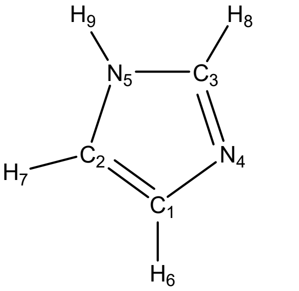	$^{3}J(^{15}N_{5},^{1}H_{6})$	-3.62584
277		$^{2}J(^{13}C_{1},^{1}H_{7})$	8.4218
278		$^{1}J(^{13}C_{2},^{1}H_{7})$	184.6627
279		$^{3}J(^{13}C_{3},^{1}H_{7})$	6.8473
280		$^{3}J(^{15}N_{4},^{1}H_{7})$	-1.15994
281		$^{2}J(^{15}N_{5},^{1}H_{7})$	-4.67903
282		$^{3}J(^{1}H_{7},^{1}H_{6})$	2.083
283	1*H*-imidazole (**36**)	$^{3}J(^{13}C_{1},^{1}H_{8})$	11.4213
284		$^{3}J(C_{2},^{1}H_{8})$	3.38
285		$^{1}J(C_{3},^{1}H_{8})$	202.3116
286		$^{2}J(^{15}N_{4},^{1}H_{8})$	-11.2892
287		$^{2}J(^{15}N_{5},^{1}H_{8})$	-8.99896
288		$^{4}J(^{1}H_{8},^{1}H_{6})$	0.2527
289		$^{4}J(^{1}H8,^{1}H_{7})$	1.3424
290		$^{3}J(^{13}C_{1},^{1}H_{9})$	7.9148
291		$^{2}J(^{13}C_{2},^{1}H_{9})$	4.5208
292		$^{2}J(^{13}C_{3},^{1}H_{9})$	4.7788
293		$^{3}J(^{15}N_{4},^{1}H_{9})$	0.11208
294		$^{1}J(^{15}N,^{1}H)$	-97.1588
295		$^{4}J(^{1}H_{9},^{1}H_{6})$	1.7199
296		$^{3}J(^{1}H_{9},^{1}H_{7})$	2.101
297		$^{3}J(^{1}H_{9},^{1}H_{8})$	0.9929
298		$^{1}J(^{13}C_{1},^{13}C_{2})$	57.8942
299		$^{2}J(^{13}C_{2},^{13}C_{3})$	-3.9801
300		$^{2}J(^{13}C_{1},^{13}C_{4})$	-3.5282
301	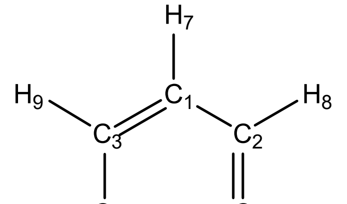	$^{1}J(^{13}C_{2},^{13}C_{4})$	58.6905
302		$^{3}J(^{13}C_{3},^{13}C_{4})$	15.0162
303		$^{2}J(^{13}C_{5},^{13}C_{4})$	-7.1321
304		$^{3}J(^{15}N_{6},^{13}C_{1})$	-4.46455
305	pyridine (**37**)	$^{2}J(^{15}N_{6},^{13}C_{2})$	3.058709
306		$^{1}J(^{15}N,^{13}C)$	-2.12307

307		${}^{1}J({}^{13}C_{1},{}^{1}H_{7})$	156.4461
308		${}^{2}J({}^{13}C_{2},{}^{1}H_{7})$	-0.285
309		${}^{3}J({}^{13}C_{4},{}^{1}H_{7})$	6.8939
310		${}^{4}J({}^{15}N,{}^{1}H)$	0.544409
311		${}^{2}J({}^{13}C_{1},{}^{1}H_{8})$	-0.2265
312		${}^{1}J({}^{13}C_{2},{}^{1}H_{8})$	159.2298
313		${}^{3}J({}^{13}C_{3},{}^{1}H_{8})$	6.7428
314		${}^{2}J({}^{13}C_{4},{}^{1}H_{8})$	2.0487
315		${}^{4}J({}^{13}C_{5},{}^{1}H_{8})$	-1.2969
316		${}^{3}J({}^{15}N,{}^{1}H)$	-1.65609
317		${}^{3}J({}^{1}H_{7},{}^{1}H_{8})$	8.0691
318		${}^{4}J({}^{1}H_{8},{}^{1}H_{9})$	0.8026
319		${}^{3}J({}^{13}C_{1},{}^{1}H_{10})$	6.7298
320		${}^{2}J({}^{13}C_{2},{}^{1}H_{10})$	7.6787
321		${}^{4}J({}^{13}C_{3},{}^{1}H_{10})$	-2.0622
322		${}^{1}J({}^{13}C_{4},{}^{1}H_{10})$	173.6921
323		${}^{3}J({}^{13}C_{5},{}^{1}H_{10})$	11.4202
324		${}^{2}J({}^{15}N,{}^{1}H)$	-9.92169
325		${}^{4}J({}^{1}H_{7},{}^{1}H_{10})$	1.3155
326		${}^{3}J({}^{1}H_{8},{}^{1}H_{10})$	5.4483
327		${}^{5}J({}^{1}H,{}^{1}H)$	1.3336
328		${}^{4}J({}^{1}H_{10},{}^{1}H_{11})$	-0.68
329		${}^{2}J({}^{15}N,{}^{13}C)$	-4.3996
330		${}^{1}J({}^{13}C_{2},{}^{13}C_{3})$	55.6887
331		${}^{1}J({}^{15}N,{}^{13}C)$	-16.2725
332		${}^{1}J({}^{13}C_{5},{}^{13}C_{3})$	71.8971
333		${}^{2}J({}^{13}C_{2},{}^{13}C_{5})$	1.251
334		${}^{2}J({}^{13}C_{4},{}^{13}C_{5})$	7.3764
335		${}^{3}J({}^{15}N,{}^{1}H)$	-5.54972
336	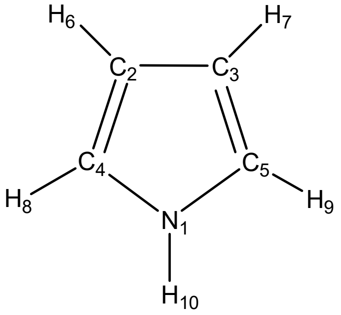	${}^{1}J({}^{13}C_{3},{}^{1}H_{7})$	167.5539
337		${}^{2}J({}^{13}C_{2},{}^{1}H_{7})$	3.4862
338		${}^{2}J({}^{13}C_{5},{}^{1}H_{7})$	7.2697
339		${}^{3}J({}^{13}C_{4},{}^{1}H_{7})$	7.3935
340		${}^{3}J({}^{1}H_{6},{}^{1}H_{7})$	3.6187
341		${}^{2}J({}^{15}N,{}^{1}H)$	-4.06841
342		${}^{2}J({}^{13}C_{3},{}^{1}H_{9})$	6.4495
343		${}^{3}J({}^{13}C_{2},{}^{1}H_{9})$	7.6804
344	1*H*-pyrrole (**38**)	${}^{1}J({}^{13}C_{5},{}^{1}H_{9})$	180.0386
345		${}^{3}J({}^{13}C_{4},{}^{1}H_{9})$	6.0373
346		${}^{3}J({}^{1}H_{7},{}^{1}H_{9})$	3.618
347		${}^{4}J({}^{1}H_{6},{}^{1}H_{9})$	0.9557
348		${}^{4}J({}^{1}H_{8},{}^{1}H_{9})$	2.1144
349		${}^{1}J({}^{15}N,{}^{1}H)$	-97.0007
350		${}^{3}J({}^{13}C_{3},{}^{1}H_{10})$	7.1622
351		${}^{2}J({}^{13}C_{5},{}^{1}H_{10})$	3.692

352		$^{4}J(^{1}H_{7},^{1}H_{10})$	2.4789
353		$^{3}J(^{1}H_{9},^{1}H_{10})$	2.7997
354		$^{1}J(^{13}C_{2},^{13}C_{1})$	74.4628
355		$^{2}J(^{13}C,^{13}C)$	0.024
356		$^{1}J(^{13}C_{2},^{13}C_{3})$	44.1467
357		$^{1}J(^{13}C_{1},^{1}H_{4})$	155.4083
358		$^{2}J(^{13}C_{2},^{1}H_{4})$	-1.7794
359		$^{3}J(^{13}C_{3},^{1}H_{4})$	12.0232
360		$^{1}J(^{13}C_{1},^{1}H_{5})$	151.6081
361		$^{2}J(^{13}C_{2},^{1}H_{5})$	-3.2511
362		$^{3}J(^{13}C_{3},^{1}H_{5})$	7.7735
363		$^{2}J(^{1}H_{4},^{1}H_{5})$	-0.0995
364		$^{2}J(^{13}C_{1},^{1}H_{6})$	-0.5197
365		$^{1}J(^{13}C_{2},^{1}H_{6})$	149.1235
366		$^{2}J(^{13}C_{3},^{1}H_{6})$	4.1209
367		$^{3}J(^{1}H_{4},^{1}H_{6})$	10.9241
368	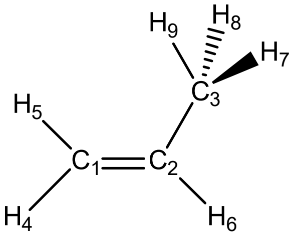	$^{3}J(^{1}H_{5},^{1}H_{6})$	16.295
369		$^{3}J(^{13}C_{1},^{1}H_{7})$	6.219
370		$^{2}J(^{13}C_{2},^{1}H_{7})$	-8.1934
371	prop-1-ene (**39**)	$^{1}J(^{13}C_{3},^{1}H_{7})$	122.5923
372		$^{4}J(^{1}H_{4},^{1}H_{7})$	-2.744
373		$^{4}J(^{1}H_{5},^{1}H_{7})$	-2.9619
374		$^{3}J(^{1}H_{6},^{1}H_{7})$	3.8893
375		$^{2}J(^{1}H_{7},^{1}H_{8})$	-19.4158
376		$^{3}J(^{13}C_{1},^{1}H_{9})$	6.5108
377		$^{2}J(^{13}C_{2},^{1}H_{9})$	-6.1258
378		$^{1}J(^{13}C_{3},^{1}H_{9})$	123.7368
379		$^{4}J(^{1}H_{4},^{1}H_{9})$	-0.3676
380		$^{4}J(^{1}H_{5},^{1}H_{9})$	-0.5527
381		$^{3}J(^{1}H_{6},^{1}H_{9})$	12.024
382		$^{2}J(^{1}H_{7},^{1}H_{9})$	-14.4192
383		$^{1}J(^{13}C_{1},^{13}C_{2})$	53.3786
384		$^{1}J(^{13}C_{1},^{13}C_{3})$	75.2337
385		$^{2}J(^{13}C_{2},^{13}C_{3})$	0.2157
386		$^{2}J(^{13}C_{3},^{13}C_{4})$	3.8388
387		$^{1}J(^{13}C_{1},^{1}H_{6})$	171.3705
388		$^{2}J(^{13}C_{2},^{1}H_{6})$	3.2652
389	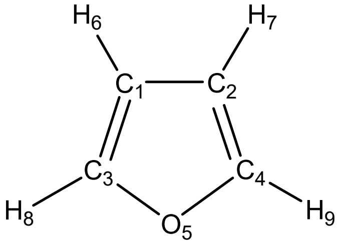	$^{2}J(^{13}C_{3},^{1}H_{6})$	9.1503
390		$^{3}J(^{13}C_{4},^{1}H_{6})$	6.8052
391		$^{3}J(^{1}H_{6},^{1}H_{7})$	3.2276
392		$^{2}J(^{13}C_{1},^{1}H_{8})$	12.4011
393	furan (**40**)	$^{3}J(^{13}C_{2},^{1}H_{8})$	6.3568
394		$^{1}J(^{13}C_{3},^{1}H_{8})$	197.3038
395		$^{3}J(^{13}C_{4},^{1}H_{8})$	6.7618
396		$^{3}J(^{1}H_{6},^{1}H_{8})$	2.7641

397		$^{4}J(^{1}H_{7},^{1}H_{8})$	0.337
398		$^{4}J(^{1}H_{8},^{1}H_{9})$	1.6686
399		$^{1}J(^{19}F,^{13}C)$	-247.654
400		$^{2}J(^{19}F,^{13}C)$	22.4162
401		$^{1}J(^{13}C_{2},^{13}C_{3})$	74.5657
402		$^{2}J(^{13}C_{3},^{13}C_{4})$	3.1879
403		$^{3}J(^{19}F,^{13}C)$	5.6552
404		$^{2}J(^{13}C_{2},^{13}C_{6})$	-0.263
405		$^{1}J(^{13}C_{3},^{13}C_{6})$	61.1019
406		$^{3}J(^{13}C_{4},^{13}C_{6})$	7.5558
407		$^{2}J(^{13}C_{5},^{13}C_{6})$	-2.2094
408		$^{4}J(^{19}F,^{13}C)$	4.8362
409		$^{3}J(^{13}C_{7},^{13}C_{2})$	11.3139
410		$^{2}J(^{13}C_{3},^{13}C_{7})$	-3.7233
411		$^{1}J(^{13}C_{6},^{13}C_{7})$	60.3641
412		$^{3}J(^{19}F,^{1}H)$	6.7011
413		$^{2}J(^{13}C_{2},^{1}H_{8})$	-6.2911
414		$^{1}J(^{13}C_{3},^{1}H_{8})$	159.6454
415	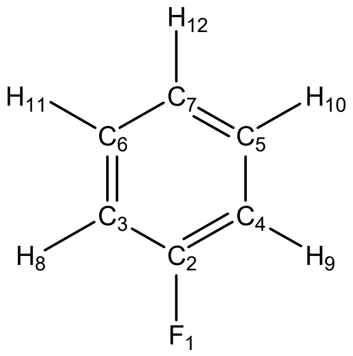	$^{3}J(^{13}C_{4},^{1}H_{8})$	4.48
416		$^{2}J(^{13}C_{6},^{1}H_{8})$	-1.3689
417		$^{4}J(^{13}C_{5},^{1}H_{8})$	-1.2311
418		$^{3}J(^{13}C_{7},^{1}H_{8})$	7.8951
419		$^{4}J(^{1}H_{8},^{1}H_{9})$	2.0903
420		$^{4}J(^{19}F,^{1}H)$	4.9471
421		$^{3}J(^{13}C_{2},^{1}H_{11})$	10.8239
422	1-fluorobenzene (**41**)	$^{2}J(^{13}C_{3},^{1}H_{11})$	-0.0824
423		$^{4}J(^{13}C_{4},^{1}H_{11})$	-1.9009
424		$^{1}J(^{13}C_{6},^{1}H_{11})$	156.8481
425		$^{3}J(^{13}C_{5},^{1}H_{11})$	9.0912
426		$^{2}J(^{13}C_{7},^{1}H_{11})$	-0.3907
427		$^{3}J(^{1}H_{8},^{1}H_{11})$	8.8248
428		$^{5}J(^{1}H_{9},^{1}H_{11})$	0.904
429		$^{4}J(^{1}H_{10},^{1}H_{11})$	1.1549
430		$^{5}J(^{19}F,^{1}H)$	-1.1575
431		$^{4}J(^{13}C_{2},^{1}H_{12})$	-2.2701
432		$^{3}J(^{13}C_{3},^{1}H_{12})$	8.3327
433		$^{2}J(^{13}C_{6},^{1}H_{12})$	0.5275
434		$^{1}J(^{13}C_{7},^{1}H_{12})$	158.5151
435		$^{4}J(^{1}H_{8},^{1}H_{12})$	0.5461
436		$^{3}\mathrm{J}(^{1}H_{11},^{1}H_{12})$	8.0529
